# Design and synthesis of doublecortin-like kinase 1 inhibitors and their bioactivity evaluation

**DOI:** 10.1080/14756366.2023.2287990

**Published:** 2023-12-07

**Authors:** Pengming Pan, Dengbo Ji, Zhongjun Li, Xiangbao Meng

**Affiliations:** a State Key Laboratory of Natural and Biomimetic Drugs, School of Pharmaceutical Sciences, Peking University, Beijing, China; b Key Laboratory of Carcinogenesis and Translational Research (Ministry of Education), Department of Gastrointestinal Surgery III, Peking University Cancer Hospital & Institute, Beijing, China

**Keywords:** Doublecortin-like kinase 1 (DCLK1), kinase inhibitor, antitumor, colorectal cancer (CRC)

## Abstract

Doublecortin-like kinase 1 (DCLK) is a microtubule-associated serine/threonine kinase that is upregulated in a wide range of cancers and is believed to be related to tumour growth and development. Upregulated DCLK1 has been used to identify patients at high risk of cancer progression and tumours with chemotherapy-resistance. Moreover, DCLK1 has been identified as a cancer stem cell (CSC) biomarker in various cancers, which has received considerable attention recently. Herein, a series of DCLK1 inhibitors were prepared based on the previously reported XMD8-92 structure. Among all the synthesised compounds, **D1**, **D2**, **D6**, **D7**, **D8**, **D12**, **D14**, and **D15** showed higher DCLK1 inhibitory activities (IC_50_ 40–74 nM) than XMD8-92 (IC_50_ 161 nM). Compounds **D1** and **D2** were selective DCLK1 inhibitors as they showed a rather weak inhibitory effect on LRRK2. The antiproliferative activities of these compounds were also preliminarily evaluated. The structure-activity relationship revealed by our compounds provides useful guidance for the further development of DCLK1 inhibitors.

## Introduction

Doublecortin-like kinase 1 (DCLK1) was first identified in the brain as a microtubule-associated protein (MAP) and it was found to play a crucial role in early neurogenesis^1^. Accumulating studies indicated that DCLK1 is related to tumour development. DCLK1 isoforms are overexpressed in many types of cancer cells, especially in colorectal cancer, which worsens the survival outcomes of colorectal cancer (CRC) patients^2^^,^^3^. Epithelial-mesenchymal transition (EMT) plays a key role in cancer invasion and metastasis, especially in pancreatic cancer. It was shown that small-interfering RNA (siRNA)-mediated knockdown of DCLK-1 in human pancreatic cancer cells induced the downregulation of EMT-associated transcription factors^4^. Moreover, pharmacological inhibition of DCLK1 prevented the development of DCLK1^+^ pancreatic ductal adenocarcinoma (PDAC) in clinically relevant patient-derived PDAC organoid models^5^.

Additionally, DCLK1 is recognised as a regulator of type II immune response and linked with functional regulation of the tumour microenvironment. DCLK1 is strongly linked to the infiltration of multiple immune cell types, especially tumour-associated macrophages (TAMs) and regulatory T-cells (Treg). And DCLK1 might contribute to TAM-mediated inhibition of CD8^+^ T-cells to enhance tumour growth in the tumour microenvironment^6^. In addition, it was reported that the overexpression of miR-539 enhanced the sensitivity of cisplatin (DDP)-resistant NSCLC cells to DDP by directly targeting DCLK1^7^. Considering DCLK1 played key roles in tumour growth, EMT, tumour immunity, and drug resistance, targeting DCLK1 is a potential strategy for treating cancers.

**Figure 1.**
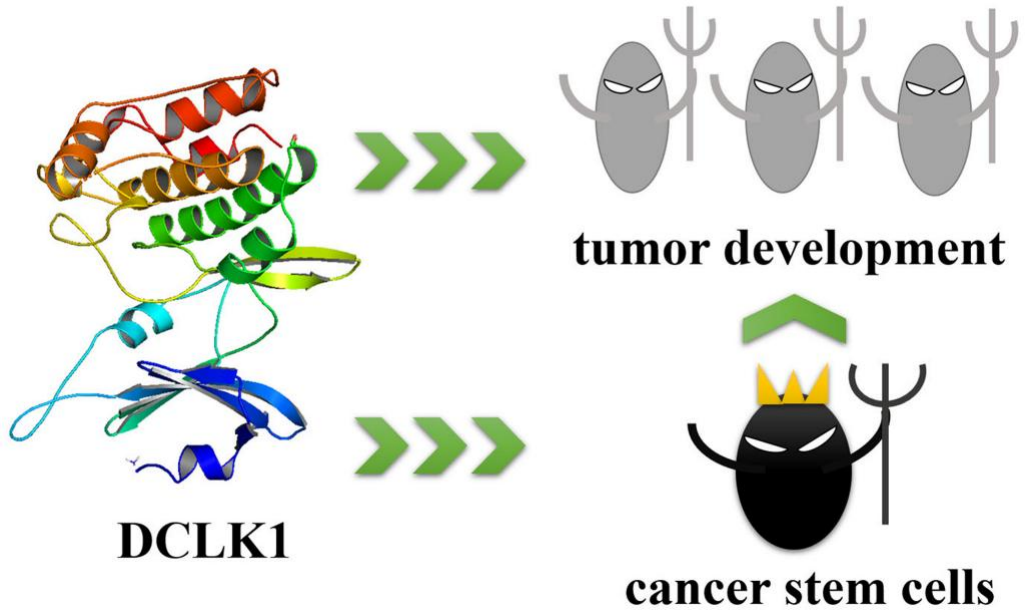
The role of DCLK1 in tumours.

Cancer stem cells (CSCs) exhibit unique self-renewal properties and are regarded as the key initiator in tumour formation, development, metastasis, and recurrence^8^^,^^9^. Because CSCs have strong resistance to traditional drug treatment, new strategies targeting CSCs have been sought^10^^,^^11^. DCLK1 is regarded as a marker of various cancer stem cells and it regulates pro-survival signalling and self-renewal of tumour cells^12–14^ (Figure 1). In the adenomatous polyposis coli (APC) mouse model, the expression of DCLK1 is positively correlated with other stem cell markers, and the enteroids formed from the intestinal DCLK^+^ cells display higher pluripotency and pro-survival signalling. DCLK1 knockdown in APC^Min/+^ mice reduced the ability of colon cancer cells to self-renew and survive^14^. Furthermore, DCLK1 marks a population of tumour stem-like cells in renal cell carcinoma (RCC). A novel monoclonal antibody targeting DCLK1 alternative splice variants-positive cells blocked RCC tumorigenesis *in vivo* and siRNA mediated knockdown of DCLK1 resulted in decreased expression of EMT and pluripotency factors in RCC^15^^,^^16^. In addition, small extracellular vesicle (sEV/exosome) biogenesis and EMT are inhibited in gastric cancer (GC)^17^ by a specific small molecule inhibitor of DCLK1.

**Figure 2.**
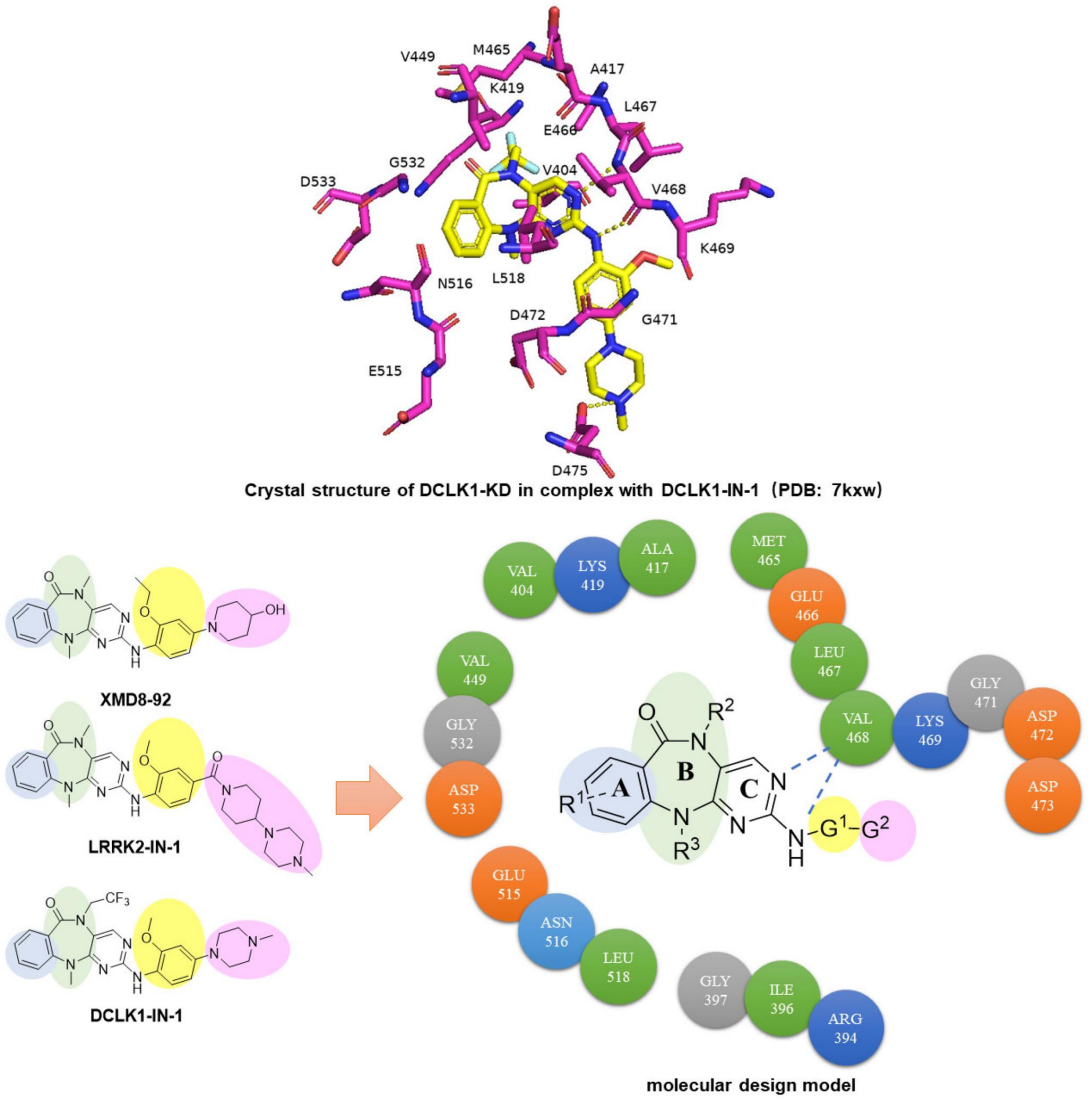
Crystal structure of DCLK1-KD in complex with DCLK1-IN-1 (PDB: 7kxw) and molecular design.

The crystal structure of DCLK1 has been reported, which provides the necessary structure information for the design of DCLK1 inhibitors^18^. However, DCLK1 and the Leucine-rich repeat kinase 2 (LRRK2) share a similar inhibitor profile. And XMD8-92 and LRRK-IN-1, which are widely used DCLK1 inhibitors, do not have good selectivity^19^^,^^20^. Therefore, we conducted chemical modifications of the structures of XMD8-92 and LRRK2-IN-1 (Figure 2), and various effective DCLK1 inhibitors with higher selectivity profiles were synthesised. Their structure–activity relationship was also investigated.

## Results and discussion

### The molecular design

DCLK1-IN-1 was an efficient DCLK1 selective inhibitor developed by Gray et al^5^. XMD8-92, LRRK-IN-1, and DCLK1-IN-1 share similar scaffold structures, namely the benzopyrimido diazepine moiety. As shown in Figure 2, we plan to introduce substitution groups on the A-ring, such as electron withdrawing or electron donating groups, to enhance the interactions with surrounding amino acid residues. On the B-ring, we plan to replace the R^2^ and R^3^ with more bulky groups because there is still space in the kinase pocket adjacent to them. The C-ring has a strong interaction with VAL468, and we intend to keep it. G^1^ and G^2^ are located at the interface between protein and solvent, and we hope to introduce hydrophilic groups to improve the physicochemical properties of the resulting compounds and their selectivity to DCLK1.

### Chemistry

**Scheme 1.**
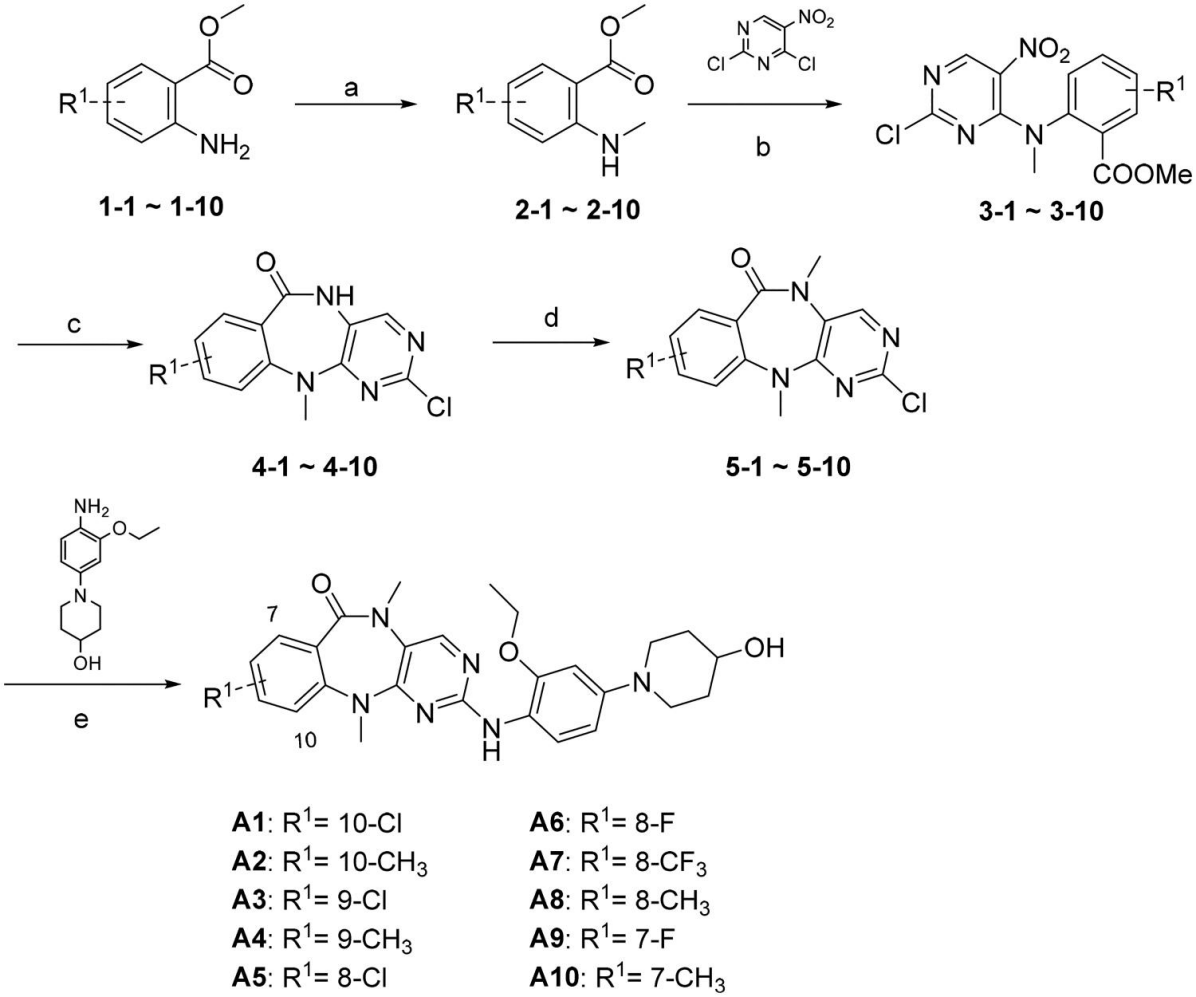
Synthesis of compounds **A1**–**A10**. Reagents and conditions: (a) NaY, 150 °C, dimethyl carbonate, 23–55%; (b) DIEA, 1,4-dioxane, 50 °C, 33–89%; (c) SnCl_2_·2H_2_O, EtOAc, 70 °C, 41–89%; (d) CH_3_I, NaH, THF, 0 °C, 48–100%; (e) Pd(OAc)_2_, XPhos, Cs_2_CO_3_, *t*-BuOH, 110 °C, 15–41%.

**Scheme 2.**
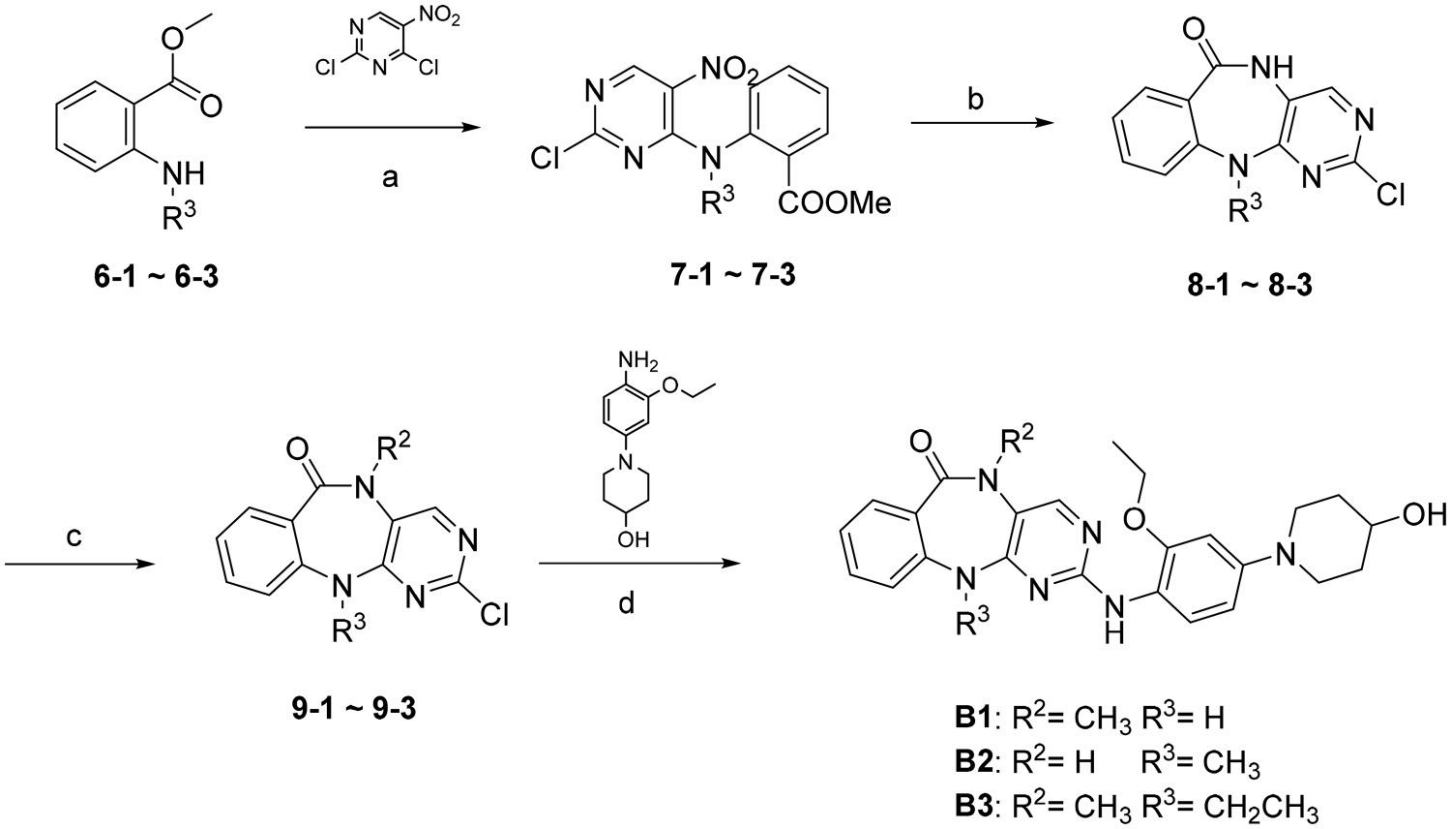
Synthesis of compounds **B1**–**B3**. (a) DIEA, 1,4-dioxane, 50 °C, 70–85%; (c) SnCl_2_·2H_2_O, EtOAc, 70 °C, 43–95%; (d) R^2^I, NaH, THF, 0 °C, 57–100%; (e) Pd(OAc)_2_, XPhos, Cs_2_CO_3_, *t*-BuOH, 110 °C, 15–41%.

The synthesis method for XMD8-92 analogues reported by Gray’s et al.^20^^,^^21^ was adopted and modified in this study. As shown in Scheme 1, substituted anthranilates **1-1–1-10** were transformed into *N*-methylated products **2-1–2-10** in the presence of dimethyl carbonate and NaY molecular sieves^22^. Compounds **3-1–3-10** were synthesised through the nucleophilic substitution of 2,4-dichloro-5-nitropyrimidine by the secondary amine of compounds **2-1–2-10**. The benzopyrimido diazepines **4-1–4-10** were achieved by reduction of the nitro group of compounds **3-1–3-10** followed by the spontaneous closure of the seven membered ring. Next, compounds **5-1–5-10** were obtained via methylation of compounds **4-1–4-10** by methyl iodide and NaH in anhydrous THF. Finally, **A1–10** was obtained via Pd-catalysed Buchwald-Hartwig cross coupling reaction in low to moderate yields. As shown in Scheme 2, the synthesis of the **B** series is similar to that of the A series. The series **C** and **D** compounds were modified at the G^1^ and G^2^ positions (Figure 2).

**Scheme 3.**
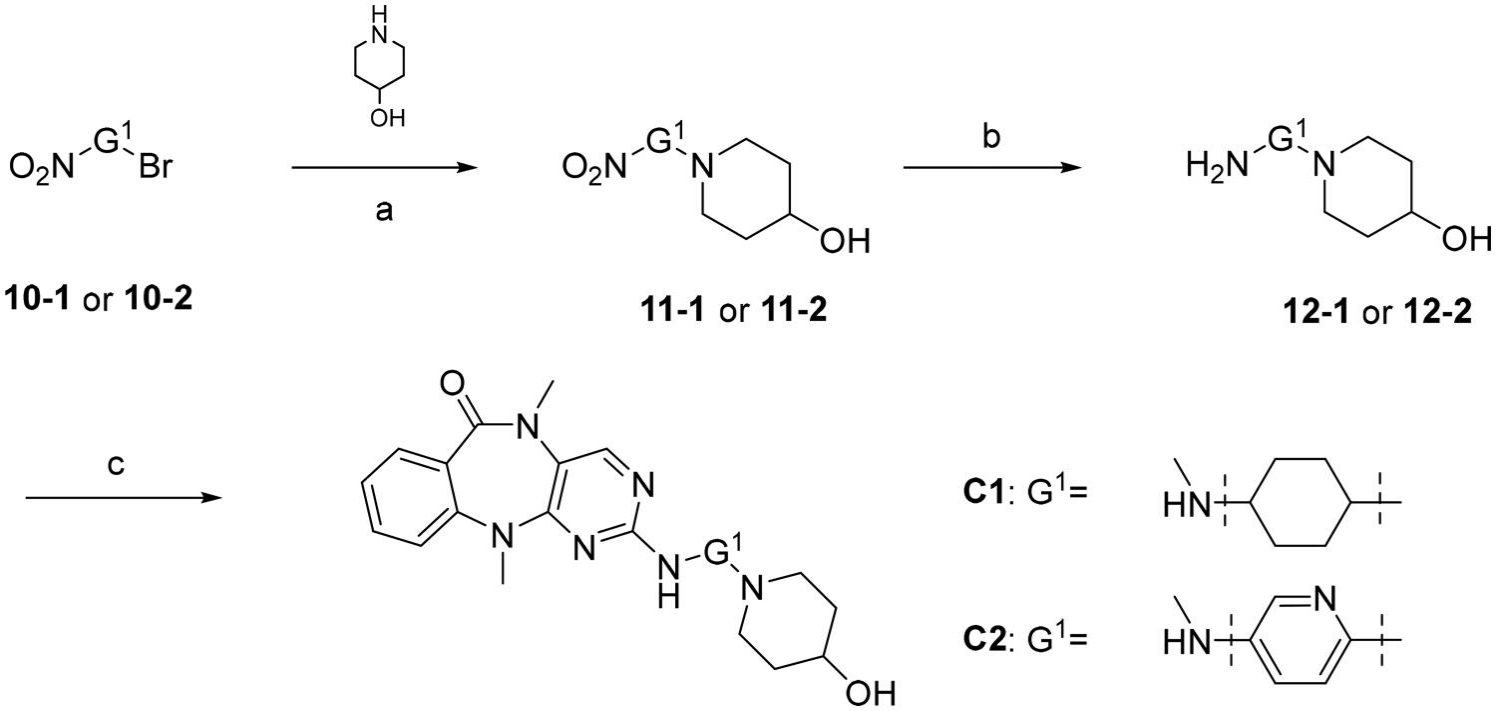
Synthesis of compounds **C1**, **C2**. (a) K_2_CO_3_, DMF, 50 °C, 80–96%; (b) SnCl_2_·2H_2_O, EtOAc, 70 °C, 52–78%; (c) Pd(OAc)_2_, XPhos, Cs_2_CO_3_, *t*-BuOH, 110 °C, 35–46%.

**Scheme 4.**
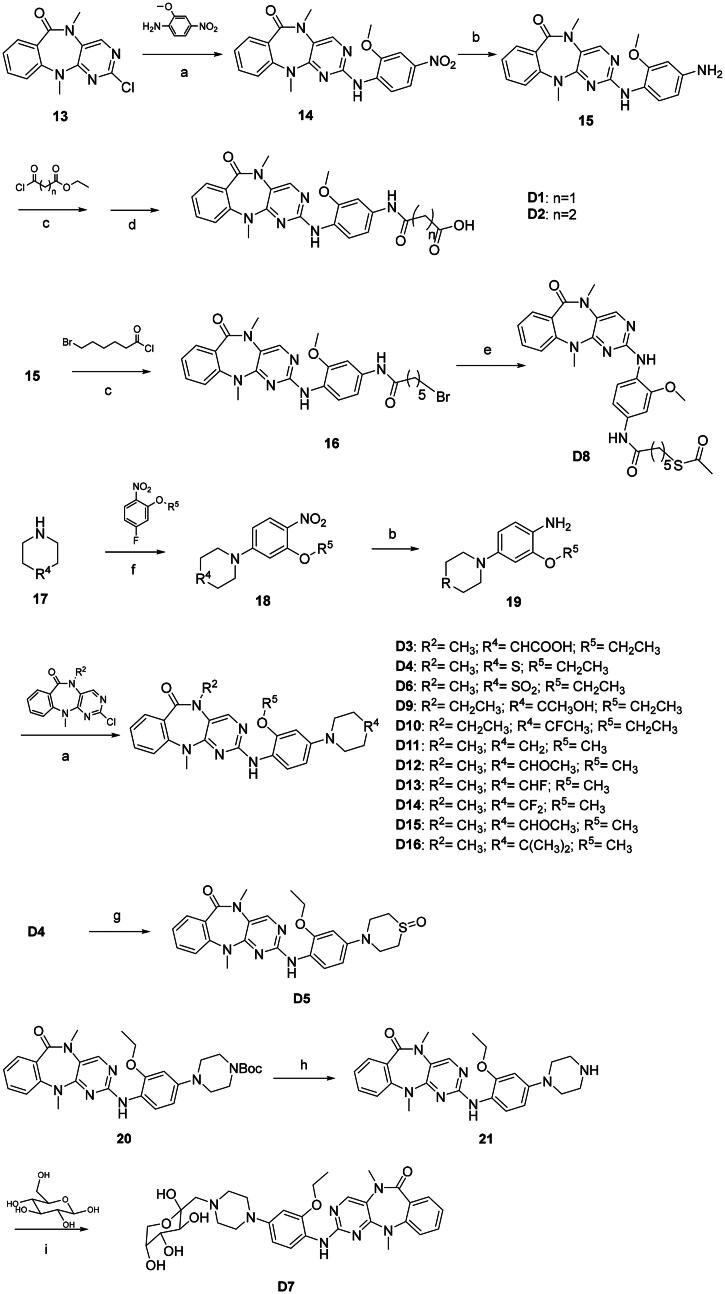
Synthesis of compounds **D1**–**D16**. (a) Pd(OAc)_2_, XPhos, Cs_2_CO_3_, *t*-BuOH, 110 °C, 30–48%; (b)H_2_, Pd/C, r.t., 100%; (c) Et_3_N, CH_2_Cl_2_, r.t.; (d) LiOH (2 M), THF, 90% over two steps; (e) CH_3_COSK, DMF, 100 °C, 38%; (f) K_2_CO_3_, DMF, 90 °C, 91%; (g) H_2_O_2_, AcOH, r.t., 95%; (h) TFA, CH_2_Cl_2_, r.t., 100%; (i) AcOH, EtOH, reflux, 82%.

The synthesis of series **C** compounds proceeded from the synthesis of G^1^ and G^2^ groups before carrying out coupling reaction with compound **13** (Scheme 3). Most of the compounds of series **D** were also prepared via similar procedure. Alternatively, compounds **D1**, **D2**, **D5**, **D7**, and **D8** were obtained by coupling reaction of G^1^/G^2^ moiety, followed by further modification (Scheme 4). It is worth mentioning that compound **D8** was synthesised with a longer linker, which could be used as a probe for DCLK1. Furthermore, some compounds were introduced with hydrophilic groups, such as compounds **D5** and **D6** with hydrophilic sulfoxide or sulphone groups, respectively, and compound **D7** with a fructose group via the Maillard reaction^23^. In addition, compounds with lipophilic G^2^ groups were also synthesised, such as **D10**–**D14**. In total, more than 30 compounds were synthesised to evaluate their DCLK1 inhibitory activity.

### In vitro *evaluation of DCLK1 inhibitory activity*

All compounds were tested for their kinase inhibitory activity at the concentration of 1 μM. As shown in Table 1, the kinase activity of DCLK1 and LRRK2 after drug treatment was presented as a percentage, with 100% indicating no inhibitory activity and 0% indicating complete inhibition. Compounds **D1**, **D2**, **D5**-**D8**, **D11**, **D12**, **D14**, and **D15** exhibited higher DCLK1 kinase inhibitory activity than XMD8-92. In addition, at the same concentration, compounds **A6**, **A9**, **B3**, **D1**, **D2**, and **D7** exhibited higher selectivity towards DCLK1 comparing with LRRK2. Compounds with high activity or good selectivity were selected for further IC_50_ determination (Table 2). The IC_50_ values of XMD8-92 for DCLK1 and LRRK2 kinases are similar, which indicates poor selectivity. Most compounds exhibited good DCLK1 inhibitory activity, and nine compounds exhibited higher DCLK1 inhibitory activity than XDM8-92. It is worth noting that compounds **D1**, **D2**, and **D8** had significantly increased DCLK1 kinase selectivity with enhanced kinase inhibitory activity.

**Table 1.** DCLK1 and LRRK2 inhibitory activities of the synthesised compounds at the concentration of 1 uM.

Entry	DCLK1 activity (%)	LRRK2 activity (%)	Selectivity (DCLK1 activity/LRRK2 activity)
XMD8-92	14	18	0.78
**A1**	25	8	3.13
**A2**	103	100	1.03
**A3**	85	88	0.97
**A4**	89	83	1.07
**A5**	87	89	0.98
**A6**	19	51	0.37
**A7**	109	109	1.00
**A8**	102	93	1.10
**A9**	17	50	0.34
**A10**	96	98	0.98
**B1**	102	76	1.34
**B2**	59	72	0.82
**B3**	28	70	0.40
**C1**	56	99	0.57
**C2**	15	7	2.14
**D1**	7	56	0.13
**D2**	10	49	0.20
**D3**	20	34	0.59
**D4**	18	33	0.55
**D5**	12	21	0.57
**D6**	9	19	0.47
**D7**	6	21	0.29
**D8**	8	22	0.36
**D9**	26	51	0.51
**D10**	51	81	0.63
**D11**	11	18	0.61
**D12**	5	10	0.50
**D13**	59	72	0.82
**D14**	7	16	0.44
**D15**	4	9	0.44
**D16**	15	33	0.45

**Table 2.** DCLK1 and LRRK2 inhibitory IC_50_ values of the synthesised compounds.

Entry	IC_50_ of DCLK1 (nM)	IC_50_ of LRRK2 (nM)	Selectivity (IC_50_ of DCLK1/IC_50_ of LRRK2)
XMD8-92	161	219	0.74
**A6**	365	>1000	<0.37
**A9**	338	>1000	<0.34
**B3**	324	>1000	<0.32
**D1**	47	>1000	<0.05
**D2**	70	>1000	<0.07
**D3**	340	>1000	<0.34
**D4**	271	425	0.64
**D5**	146	320	0.46
**D6**	74	195	0.38
**D7**	46	154	0.30
**D8**	40	228	0.18
**D11**	100	266	0.38
**D12**	47	99	0.47
**D14**	53	132	0.40
**D15**	52	89	0.58

### Structure–activity relationships

**Figure 3.**
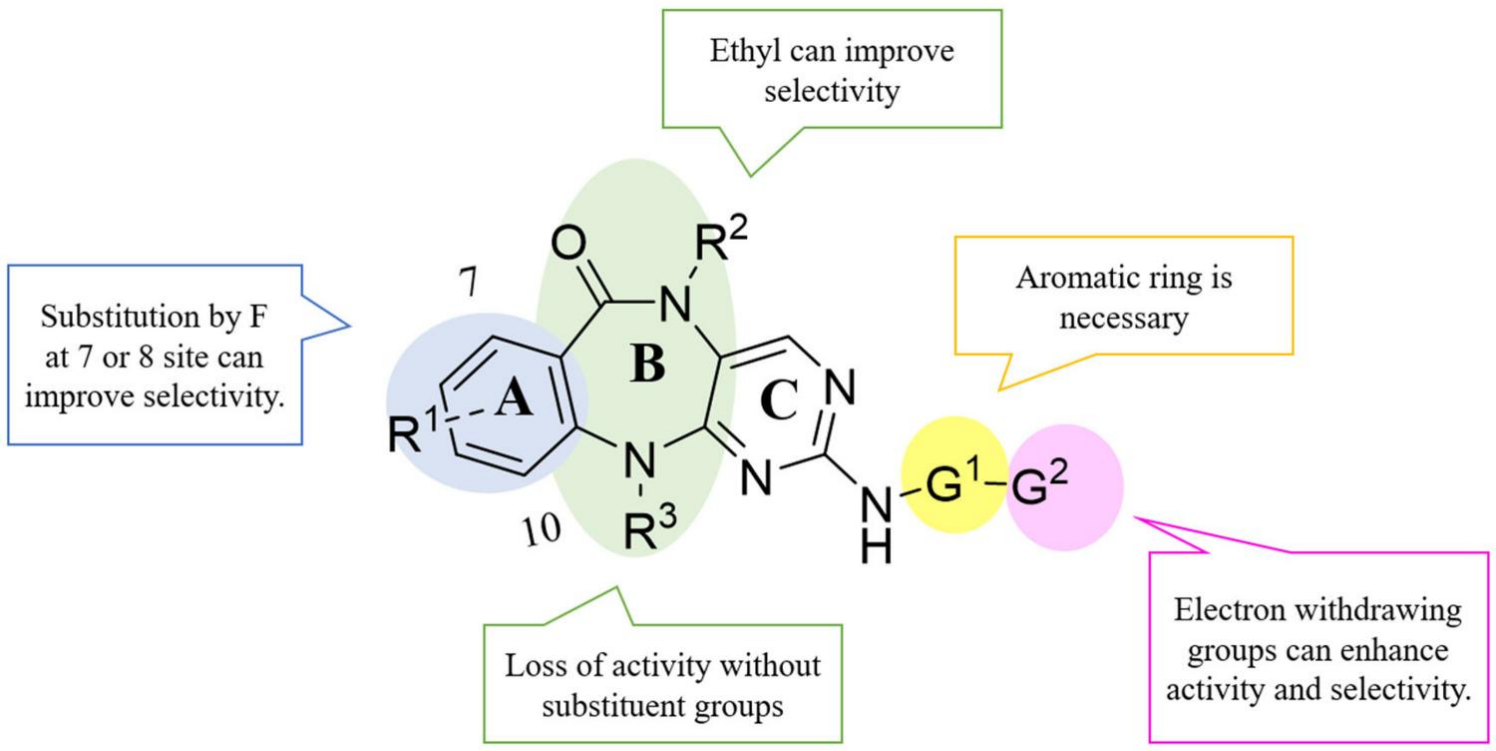
Structure–activity relationship analysis of DCLK1 inhibitors.

We analysed the structure–activity relationship based on the kinase inhibitory data of the synthesised compounds (Figure 3). Firstly, any substituents on the A-ring led to decreased or lost activity, even the smallest F atom. Secondly, when the R^2^ substituent was ethyl, the DCLK1 selectivity of compound **B3** was improved, indicating that this position may accommodate larger groups, which was also mentioned in Gray’s reports^5^^,^^24^. The DCLK1 inhibitory activity of compound **B2** decreased, indicating that the R^3^ cannot be H atom. Compared with compound **C2** with a pyridine ring structure, the DCLK1 inhibitory activity of compound **C1** with an aliphatic ring was weakened, indicating the necessity of G^1^ being an aromatic ring. The introduction of carboxyl groups at G2 increased the DCLK1 selectivity for compounds **D1** and **D2**. The introduction of difluorinated compound **D14** also has higher DCLK1 selectivity than monofluorinated compounds **D10** and **D13**. Therefore, we believe that the G2 group is more important for the selectivity of DCLK1. In addition, compared with **D10–D14** with hydrophobic groups, **D5–D7** carrying hydrophilic G2 groups exhibited little effects on the activity and selectivity of DCLK1. Interestingly, when the G2 moiety was glucose (**D7**) or long-chain group (**D8**), enhanced DCLK1 inhibitory activity and selectivity were observed, indicating the potential in developing more potent and selective DCLK1 inhibitors with this class of compounds.

### Anti-proliferation activity assay

**Figure 4.**
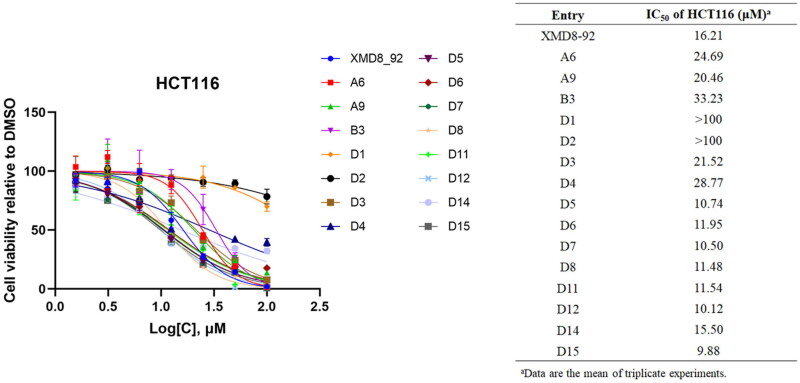
The antiproliferative effects of most compounds on HCT116.

To determine whether DCLK1 was required for tumour cell proliferation, the cytotoxicity of compounds was evaluated in colorectal cancer cell line HCT116. HCT116 was treated with compounds for 72 h, and the cell viability was determined using CCK8 assay (Figure 4). The antiproliferative effects of most compounds on HCT116 were correlated with their DCLK1 inhibitory activities, except for compounds **D1** and **D2**. The poor cytotoxicity of compounds **D1** and **D2** may be attributed to their poor cell membrane permeability due to the negatively charged carboxylates. Compounds **D5–8**, **D11–12**, and **D14–15** showed higher anti-proliferate activity than the positive control XMD8-92. Overall, targeting DCLK1 inhibited the proliferation of HCT116, indicating the importance of DCLK1 in tumour growth.

### Molecular docking study

**Figure 5.**
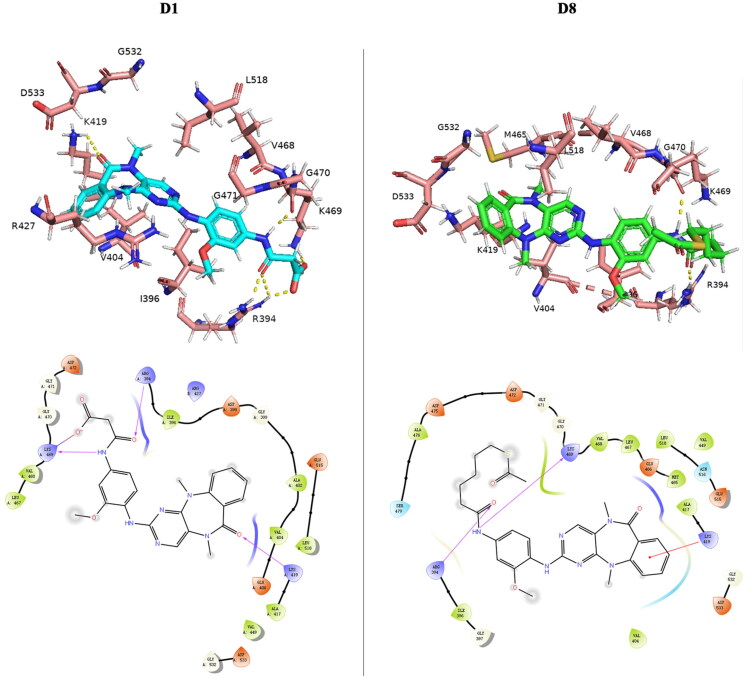
Docking of compounds **D1** and **D8** to the residues of DCLK1 subunit (PDB ID: 5JZN).

Due to the excellent kinase inhibitory activity exhibited by compounds **D1** and **D8**, the molecular docking study was performed to predict the possible binding mode with DCLK1 subunit^18^ (PDB ID: 5JZN). Through docking software, compounds **D1** and **D8** were successfully localised in the binding pocket of DCLK1 subunit. Similar to the crystal structures of DCLK1-KD in complex with reported inhibitors^25^, the benzodiazepine scaffolds are located on the inner side of the hydrophobic pocket. As shown in Figure 5, compound **D1** exhibited three hydrogen bonds. The carbonyl group on the diazepine ring formed a hydrogen bond with LYS419. Notably, two hydrogen bonds were formed by the carboxyl group with residues LYS469 and ARG394. In addition, the carboxyl group formed a salt bridge with LYS469 which enhanced the interaction between **D1** and DCLK1. The docking results of compounds **D8** and **D1** were similar. Compound **D8** formed two hydrogen bonds with residues LYS469 and ARG394. The hydrophobic interaction of benzodiazepine rings matched the surrounding hydrophobic amino acids. And the benzene ring had a *p*–*π* conjugation effect with LYS419. These interactions maintained the activity of compound **D8** against DCLK1 even with a long “tail”. Compared to the crystal structure of DCLK1-IN-1 in Figure 2, compounds **D1** and **D8** gave up hydrogen bonding with residue VAL468 and chose to form more hydrogen bonds with residues LYS469 and ARG394, which may be more advantageous in enhancing the binding force and thus enhancing the inhibitory activity against DCLK1. Interestingly, compound **D8** with a long-chain group replacing the carboxylic group did not undermine the binding to DCLK1, which also suggests that compound **D8** may be suitable for developing a probe targeting DCLK1, such as proteolysis-targeting chimaeras (PROTACs)^26^. The docking results somewhat explain the elevation of DCLK1 kinase activity by compounds **D1** and **D8** as well as validate the discussion in the structure–activity relationship analysis.

## Conclusion

DCLK1 is an important marker of cancer stem cell and an important anti-tumour target. In this study, we synthesised a series of derivatives of XMD8-92 which is widely used as a non-selective DCLK1 inhibitor. We found that compounds **D1**, **D2**, **D5**, **D6**, **D7**, **D8**, **D12**, **D14**, and **D15** exhibited higher activity than XMD8-92, among which compounds **D1**, **D2**, and **D8** exhibited good DCLK1 selectivity. The cytotoxicity assay demonstrated that this series of compounds are suitable leads for developing new anti-tumour drugs.

## Experimental section

### Chemistry

All reagents and solvents were obtained from commercial suppliers (Bide Pharmatech, Energy Chemical, Alfa, etc.) without further purification unless noted. Reactions were monitored by TLC. Thin layer chromatography was carried out using TLC silica gel 60 F254 plates. Flash column chromatography was performed with 200–300 mesh silica gel. The NMR spectrum was recorded on a Bruker-400 NMR spectrometer, with TMS as an internal standard and chemical shifts reported in ppm (*δ*). Coupling constants (*J*) are reported in Hz. The melting point was measured by an X-5 micro melting point metre. High-resolution mass spectra (HRMS) were obtained on a Shimadzu LCMS-IT-TOF mass spectrometer.

#### Preparation of intermediate 2-1–2-10

A sealed tube (100 ml of internal volume) was charged with a solution of compound **1-1–1-10** (0.12 M) and NaY (1:1 w/w) in dimethyl carbonate (30 ml). Air in the reaction mixture was replaced by N_2_ at room temperature. The sealed tube was then heated at 150 °C for 15 h. After the reaction was completed, the sealed tube was cooled to room temperature and opened slowly. The NaY was filtered out and the filtrate was concentrated *in vacuo*. Purification by silica gel column chromatography (petroleum ether/EtOAc, 20:1 v/v) gave intermediate **2-1–2-10** (23–55%).

##### Methyl 4-methyl-2-(methylamino)benzoate (2–4)

White solid; yield 51%; m.p. 49–50 °C; ^1^H NMR (400 MHz, CDCl_3_) *δ* 7.80 (d, *J* = 8.1 Hz, 1H), 7.62 (s, 1H), 6.49 (s, 1H), 6.44 (d, *J* = 8.3 Hz, 1H), 3.86 (s, 3H), 2.93 (d, *J* = 5.1 Hz, 3H), 2.35 (s, 3H).^13^C NMR (101 MHz, CDCl_3_) *δ* 169.06, 152.04, 145.41, 131.49, 115.76, 110.95, 107.45, 51.28, 29.54, 22.19.

#### Preparation of intermediate 3-1–3-10 or 7-1–7-3

**2** or **6** (1 eq), 2,4-dichloro-5-nitropyrimidine (1.5 eq), and DIEA (1.5 eq) were dissolved in 1,4-dioxane and heated at 50 °C for 6–10 h. The solvent was concentrated, and the residue was diluted with water (50 ml) and extracted with DCM (50 ml) × 3. The organics were combined and washed with brine (20 ml), dried over Na_2_SO_4_, filtered, and concentrated *in vacuo*. Purification by silica gel column chromatography (eluant: petroleum ether/EtOAc, 10:1 v/v) gave intermediate **3-1–3-10** or **7-1–7-3** (33–89%).

##### Methyl 3-chloro-2-((2-chloro-5-nitropyrimidin-4-yl)amino)benzoate (3-1)

Yellow solid; yield 33%; m.p. 115–116 °C; ^1^H NMR (400 MHz, DMSO-d6) *δ* 10.71 (s, 1H), 9.24 (s, 1H), 7.91 (t, *J* = 8.2 Hz, 2H), 7.56 (t, *J* = 8.0 Hz, 1H), 3.73 (s, 3H). ^13^C NMR (101 MHz, DMSO) *δ* 165.27, 162.65, 158.52, 154.90, 134.15, 134.04, 133.60, 130.23, 129.88, 129.58, 127.83, 53.00. HRMS-ESI (m/z): [M + H]^+^ calcd for C_12_H_8_Cl_2_N_4_O_4_: 342.9995, found: 342.9993.

##### Methyl 2-((2-chloro-5-nitropyrimidin-4-yl)amino)-3-methylbenzoate (3-2)

Yellow solid; yield 62%; m.p. 167–168 °C; ^1^H NMR (400 MHz, DMSO-d6) *δ* 10.57 (s, 1H), 9.19 (s, 1H), 7.79 (d, *J* = 7.7 Hz, 1H), 7.62 (d, *J* = 7.6 Hz, 1H), 7.43 (t, *J* = 7.7 Hz, 1H), 3.70 (s, 3H), 2.24 (s, 3H). ^13^C NMR (101 MHz, DMSO) *δ* 166.32, 162.67, 158.19, 154.98, 137.50, 135.15, 135.07, 128.60, 128.02, 127.99, 52.63, 18.45. HRMS-ESI (m/z): [M + H]^+^ calcd for C_13_H_11_ClN_4_O_4_: 323.0542, found: 323.0543.

##### Methyl 4-chloro-2-((2-chloro-5-nitropyrimidin-4-yl)(methyl)amino)benzoate (3-3)

White solid; yield 51%; m.p. 135–136 °C; ^1^H NMR (400 MHz, CDCl_3_) *δ* 8.55 (s, 1H), 8.00 (d, *J* = 8.5 Hz, 1H), 7.43 (dd, *J* = 8.5, 2.1 Hz, 1H), 7.24 (d, *J* = 2.1 Hz, 1H), 3.81 (s, 3H), 3.57 (s, 3H). ^13^C NMR (101 MHz, CDCl_3_) *δ* 163.99, 161.00, 155.55, 154.64, 144.11, 140.10, 133.67, 131.71, 129.11, 127.99, 52.68, 41.97. HRMS-ESI (m/z): [M + H]^+^ calcd for C_13_H_10_Cl_2_N_4_O_4_: 357.0152, found: 357.0151.

##### Methyl 2-((2-chloro-5-nitropyrimidin-4-yl)(methyl)amino)-4-methylbenzoate (3-4)

Yellow solid; yield 85%; m.p. 143–144 °C; ^1^H NMR (400 MHz, CDCl_3_) *δ* 8.45 (s, 1H), 7.96 (d, *J* = 8.0 Hz, 1H), 7.24 (d, *J* = 8.1 Hz, 1H), 6.98 (s, 1H), 3.81 (s, 3H), 3.57 (s, 3H), 2.40 (s, 3H). ^13^C NMR (101 MHz, CDCl_3_) *δ* 164.69, 160.80, 155.06, 154.54, 145.54, 142.95, 132.70, 131.94, 129.60, 127.78, 122.77, 52.35, 41.91, 21.39. HRMS-ESI (m/z): [M + H]^+^ calcd for C_14_H_13_ClN_4_O_4_: 337.0698, found: 337.0696.

##### Methyl 5-chloro-2-((2-chloro-5-nitropyrimidin-4-yl)(methyl)amino)benzoate (3-5)

Brown solid; yield 73%; m.p. 198–199 °C; ^1^H NMR (400 MHz, CDCl_3_) *δ* 8.49 (s, 1H), 7.99 (d, *J* = 2.5 Hz, 1H), 7.54 (dd, *J* = 8.5, 2.5 Hz, 1H), 7.16 (d, *J* = 8.5 Hz, 1H), 3.80 (s, 3H), 3.52 (s, 3H). ^13^C NMR (101 MHz, CDCl_3_) *δ* 163.60, 160.89, 155.49, 154.63, 141.47, 134.59, 134.23, 132.51, 131.69, 129.07, 127.36, 52.81, 42.02. HRMS-ESI (m/z): [M + H]^+^ calcd for C_13_H_10_Cl_2_N_4_O_4_: 357.0152, found: 357.0161.

##### Methyl 2-((2-chloro-5-nitropyrimidin-4-yl)(methyl)amino)-5-fluorobenzoate (3-6)

White solid; yield 72%; m.p. 118–119 °C; ^1^H NMR (400 MHz, CDCl_3_) *δ* 8.48 (s, 1H), 7.75 (dd, *J* = 8.7, 2.9 Hz, 1H), 7.36–7.29 (m, 1H), 7.23 (dd, *J* = 8.8, 4.9 Hz, 1H), 3.84 (d, *J* = 1.0 Hz, 3H), 3.56 (s, 3H). ^13^C NMR (101 MHz, CDCl_3_) *δ* 163.60, 162.68, 160.86, 160.18, 155.28, 154.76, 138.96, 129.90, 129.82, 121.44, 119.61, 52.82, 42.10. HRMS-ESI (m/z): [M + H]^+^ calcd for C_13_H_10_ClFN_4_O_4_: 341.0447, found: 341.0446.

##### Methyl 2-((2-chloro-5-nitropyrimidin-4-yl)amino)-5-(trifluoromethyl)benzoate (3-7)

Yellow solid; yield 46%; m.p. = 149–150 °C; ^1^H NMR (400 MHz, CDCl_3_) *δ* 12.89 (s, 1H), 9.28 (s, 1H), 8.82 (d, *J* = 8.8 Hz, 1H), 8.41 (d, *J* = 2.3 Hz, 1H), 7.91 (dd, *J* = 8.9, 2.3 Hz, 1H), 4.07 (s, 3H). ^13^C NMR (101 MHz, CDCl_3_) *δ* 166.49, 163.63, 157.80, 153.13, 141.08, 130.45, 128.64, 127.94, 126.87, 124.68, 123.51, 119.06, 53.21. HRMS-ESI (m/z): [M + H]^+^ calcd for C_13_H_8_ClF_3_N_4_O_4_: 377.0259, found: 377.0258.

##### Methyl 2-((2-chloro-5-nitropyrimidin-4-yl)(methyl)amino)-5-methylbenzoate (3-8)

White solid; yield 89%; m.p. 96–97 °C; ^1^H NMR (400 MHz, CDCl_3_) *δ* 8.46 (s, 1H), 7.86 (s, 1H), 7.40 (d, *J* = 8.1 Hz, 1H), 7.09 (d, *J* = 8.1 Hz, 1H), 3.82 (s, 3H), 3.57 (s, 3H), 2.42 (s, 3H). ^13^C NMR (101 MHz, CDCl_3_) *δ* 164.90, 160.79, 155.11, 154.73, 140.45, 139.16, 134.83, 133.07, 131.90, 127.32, 125.55, 52.44, 42.03, 21.07. HRMS-ESI (m/z): [M + H]^+^ calcd for C_14_H_13_ClN_4_O_4_: 337.0698, found: 337.0695.

##### Methyl 2-((2-chloro-5-nitropyrimidin-4-yl)(methyl)amino)-6-fluorobenzoate (3-9)

Yellow solid; yield 73%; m.p. 91–92 °C; ^1^H NMR (400 MHz, CDCl_3_) *δ* 8.60 (s, 1H), 7.52 (q, *J* = 7.8 Hz, 1H), 7.17 (t, *J* = 8.9 Hz, 1H), 7.03 (d, *J* = 8.0 Hz, 1H), 3.81 (s, 3H), 3.56 (s, 3H). ^13^C NMR (101 MHz, CDCl_3_) *δ* 163.21, 162.26, 161.07, 159.70, 155.82, 154.72, 143.11, 133.48, 131.71, 122.47, 116.95, 52.89, 42.31. HRMS-ESI (m/z): [M + H]^+^ calcd for C_13_H_10_ClFN_4_O_4_: 341.0447, found: 341.0445.

##### Methyl 2-((2-chloro-5-nitropyrimidin-4-yl)(methyl)amino)-6-methylbenzoate (3-10)

Yellow solid; yield 85%; m.p. 122–123 °C; ^1^H NMR (400 MHz, CDCl_3_) *δ* 8.55 (s, 1H), 7.41 (t, *J* = 7.8 Hz, 1H), 7.24 (d, *J* = 7.7 Hz, 1H), 7.08 (d, *J* = 7.9 Hz, 1H), 3.74 (s, 3H), 3.55 (s, 3H), 2.37 (s, 3H). ^13^C NMR (101 MHz, CDCl_3_) *δ* 167.13, 160.79, 155.50, 154.74, 141.01, 138.72, 131.97, 131.50, 130.87, 129.77, 124.26, 52.30, 42.25, 20.17. HRMS-ESI (m/z): [M + H]^+^ calcd for C_14_H_13_ClN_4_O_4_: 337.0698, found: 337.0695.

#### Preparation of intermediate 4-1–4-10 or 8-1–8-3

**3-1–3-10** or **7-1–7-10** (1 eq) and SnCl_2_·2H_2_O (2 eq) in EtOAc (10 ml) was heated at 70 °C for 5–8 h. The solvent was concentrated, and the residue diluted with water (50 ml) and extracted with EtOAc (50 ml) × 3. The organics were combined and washed with brine (20 ml), dried over Na_2_SO_4_, filtered, and concentrated *in vacuo* to give the white or yellow powder, washed with MeOH and air dried to give intermediate **4-1–4-10** or **8-1–8-3** (43–95%).

##### 2,10-Dichloro-5,11-dihydro-6*H*-benzo[*e*]pyrimido[5,4-*b*][1,4]diazepin-6-one (4-1)

White solid; yield 52%; m.p. 246–247 °C; ^1^H NMR (400 MHz, DMSO-d6) *δ* 10.40 (s, 1H), 8.24 (s, 1H), 8.12 (s, 1H), 7.78 (dd, *J* = 8.0, 1.6 Hz, 1H), 7.68 (dd, *J* = 7.9, 1.6 Hz, 1H), 7.09 (t, *J* = 7.9 Hz, 1H). ^13^C NMR (101 MHz, DMSO) *δ* 165.54, 157.00, 153.20, 149.80, 139.13, 134.75, 132.36, 124.51, 124.12, 123.16, 122.61. HRMS-ESI (m/z): [M + H]^+^ calcd for C_11_H_6_Cl_2_N_4_O: 280.9991, found: 280.9992.

##### 2-Chloro-10-methyl-5,11-dihydro-6*H*-benzo[*e*]pyrimido[5,4-*b*][1,4]diazepin-6-one (4-2)

White solid; yield 58%; m.p. 210–211 °C; ^1^H NMR (400 MHz, DMSO-d6) *δ* 10.23 (s, 1H), 8.30 (s, 1H), 8.06 (s, 1H), 7.62 (d, *J* = 7.8 Hz, 1H), 7.36 (d, *J* = 7.3 Hz, 1H), 6.98 (t, *J* = 7.6 Hz, 1H), 2.34 (s, 3H). ^13^C NMR (101 MHz, DMSO) *δ* 167.15, 158.41, 153.17, 149.23, 141.40, 136.08, 130.99, 128.54, 123.70, 123.29, 122.77, 18.45. HRMS-ESI (m/z): [M + H]^+^ calcd for C_12_H_9_ClN_4_O: 261.0532, found: 261.0537.

##### 2,9-Dichloro-11-methyl-5,11-dihydro-6*H*-benzo[*e*]pyrimido[5,4-*b*][1,4]diazepin-6-one (4-3)

White solid; yield 71%; m.p. 241–242 °C; ^1^H NMR (400 MHz, DMSO-d6) *δ* 10.52 (s, 1H), 8.18 (s, 1H), 7.74 (d, *J* = 8.4 Hz, 1H), 7.34 (s, 1H), 7.28 (d, *J* = 8.4 Hz, 1H), 3.35 (s, 3H). ^13^C NMR (101 MHz, DMSO) *δ* 166.66, 160.88, 153.48, 150.13, 148.97, 138.89, 133.65, 124.62, 124.56, 124.31, 120.13, 37.35. HRMS-ESI (m/z): [M + H]^+^ calcd for C_12_H_8_Cl_2_N_4_O: 295.0148, found: 295.0150.

##### 2-Chloro-9,11-dimethyl-5,11-dihydro-6*H*-benzo[*e*]pyrimido[5,4-*b*][1,4]diazepin-6-one (4-4)

White solid; yield 82%; m.p. 238–239 °C; ^1^H NMR (400 MHz, DMSO-d6) *δ* 10.36 (s, 1H), 8.13 (s, 1H), 7.62 (d, *J* = 7.9 Hz, 1H), 7.09 (s, 1H), 7.02 (d, *J* = 8.0 Hz, 1H), 3.33 (s, 3H), 2.34 (s, 3H). ^13^C NMR (101 MHz, DMSO) *δ* 167.52, 161.41, 153.35, 149.61, 147.69, 144.76, 131.96, 125.27, 124.42, 123.01, 120.46, 37.25, 21.51. HRMS-ESI (m/z): [M + H]^+^ calcd for C_13_H_11_ClN_4_O: 275.0694, found: 275.0692.

##### 2,8-Dichloro-11-methyl-5,11-dihydro-6*H*-benzo[*e*]pyrimido[5,4-*b*][1,4]diazepin-6-one (4-5)

White solid; yield 68%; m.p. 230–231 °C; ^1^H NMR (400 MHz, DMSO-d6) *δ* 10.60 (s, 1H), 8.16 (s, 1H), 7.66 (s, 1H), 7.63 (s, 1H), 7.30 (s, 1H), 3.32 (s, 3H). ^13^C NMR (101 MHz, DMSO) *δ* 166.27, 161.11, 153.57, 150.12, 146.76, 133.70, 131.10, 128.58, 127.49, 124.16, 122.28, 37.35. HRMS-ESI (m/z): [M + H]^+^ calcd for C_12_H_8_Cl_2_N_4_O: 295.0148, found: 295.0148.

##### 2-Chloro-8-fluoro-11-methyl-5,11-dihydro-6*H*-benzo[*e*]pyrimido[5,4-*b*][1,4]diazepin-6-one (4-6)

White solid; yield 70%; m.p. 216–217 °C; ^1^H NMR (400 MHz, DMSO-d6) *δ* 10.60 (s, 1H), 8.16 (s, 1H), 7.50–7.43 (m, 2H), 7.33 (dd, *J* = 9.9, 4.6 Hz, 1H), 2.09 (s, 3H). ^13^C NMR (101 MHz, DMSO) *δ* 166.32, 161.40, 159.74, 153.58, 149.93, 144.15, 127.71, 124.19, 122.41, 121.02, 117.88, 37.42. HRMS-ESI (m/z): [M + H]^+^ calcd for C_12_H_8_ClFN_4_O: 279.0443, found: 279.0442.

##### 2-Chloro-8-(trifluoromethyl)-5,11-dihydro-6*H*-benzo[*e*]pyrimido[5,4-*b*][1,4]diazepin-6-one (4-7)

Yellow solid; yield 41%; m.p. = 241–242 °C; ^1^H NMR (400 MHz, DMSO-d6) *δ* 10.26 (s, 1H), 10.25 (s, 1H), 8.09 (d, *J* = 2.4 Hz, 1H), 7.98 (s, 1H), 7.73 (dd, *J* = 8.7, 2.4 Hz, 1H), 7.26 (d, *J* = 8.5 Hz, 1H). ^13^C NMR (101 MHz, DMSO) *δ* 164.36, 155.50, 153.01, 148.59, 146.06, 131.27, 130.53, 125.68, 122.64, 121.53, 121.06, 120.18. HRMS-ESI (m/z): [M + H]^+^ calcd for C_12_H_6_ClF_3_N_4_O: 315.0225, found: 315.0225.

##### 2-Chloro-8,11-dimethyl-5,11-dihydro-6*H*-benzo[*e*]pyrimido[5,4-*b*][1,4]diazepin-6-one (4-8)

White solid; yield 89%; m.p. 218–219 °C; ^1^H NMR (400 MHz, DMSO-d6) *δ* 10.40 (s, 1H), 8.12 (s, 1H), 7.53 (s, 1H), 7.39 (d, *J* = 8.3 Hz, 1H), 7.16 (d, *J* = 8.5 Hz, 1H), 3.33 (s, 3H), 2.28 (s, 3H). ^13^C NMR (101 MHz, DMSO) *δ* 167.64, 161.56, 153.39, 149.56, 145.42, 134.71, 133.87, 132.02, 125.61, 124.30, 120.03, 37.18, 20.33. HRMS-ESI (m/z): [M + H]^+^ calcd for C_13_H_11_ClN_4_O: 257.0694, found: 257.0693.

##### 2-Chloro-7-fluoro-11-methyl-5,11-dihydro-6*H*-benzo[*e*]pyrimido[5,4-*b*][1,4]diazepin-6-one (4-9)

White solid; yield 43%; m.p. 215–216 °C; ^1^H NMR (400 MHz, DMSO-d6) *δ* 10.58 (s, 1H), 8.20 (s, 1H), 7.55 (q, *J* = 7.8, 7.4 Hz, 1H), 7.15 (d, *J* = 8.4 Hz, 1H), 7.08 (t, *J* = 9.4 Hz, 1H), 3.33 (s, 3H). ^13^C NMR (101 MHz, DMSO) *δ* 163.77, 162.34, 153.59, 150.77, 150.24, 133.89, 133.78, 124.31, 115.99, 113.10, 112.88, 37.13. HRMS-ESI (m/z): [M + H]^+^ calcd for C_12_H_8_ClFN_4_O: 279.0443, found: 279.0444.

##### 2-Chloro-7,11-dimethyl-5,11-dihydro-6*H*-benzo[*e*]pyrimido[5,4-*b*][1,4]diazepin-6-one (4-10)

Yellow solid; yield 49%; m.p. 145–146 °C; ^1^H NMR (400 MHz, CDCl_3_) *δ* 7.63 (s, 1H), 7.32 (t, *J* = 7.8 Hz, 1H), 7.21 (d, *J* = 7.6 Hz, 1H), 6.93 (d, *J* = 7.8 Hz, 1H), 3.89 (s, 3H), 2.38 (s, 3H). ^13^C NMR (101 MHz, CDCl_3_) *δ* 167.91, 165.01, 154.59, 151.69, 149.62, 140.86, 130.81, 128.96, 127.81, 126.28, 115.68, 36.00, 21.08, 0.01. HRMS-ESI (m/z): [M + H]^+^ calcd for C_13_H_11_ClN_4_O: 275.0694, found: 275.0699.

#### Preparation of intermediate 5-1–5-10 or 9-1–9-3

**4-1–4-10** or **8-1–8-3** (1 eq), MeI (6 eq) and NaH (2 eq, 60% suspension in mineral oil) was added in THF (10 ml) at −5 °C. After the reaction was complete as monitored by TLC, the solvent was diluted with water (30 ml) and extracted with EtOAc (30 ml) × 3. The organics were combined, dried over Na_2_SO_4_, filtered, and concentrated *in vacuo*. Purification by silica gel column chromatography (eluant: petroleum ether/EtOAc, 4:1 v/v) gave intermediate **5-1–5-10** or **9-1–9-3** (48–100%).

##### 2,10-Dichloro-5,11-dimethyl-5,11-dihydro-6*H*-benzo[*e*]pyrimido[5,4-*b*][1,4]diazepin-6-one (5-1)

Yellow solid; yield 48%; m.p. 178–179 °C; ^1^H NMR (400 MHz, CDCl_3_) *δ* 7.94 (d, *J* = 8.0 Hz, 1H), 7.53 (d, *J* = 7.8 Hz, 1H), 7.13 (s, 1H), 6.99 (t, *J* = 8.2 Hz, 1H), 3.86 (s, 3H), 3.38 (s, 3H). ^13^C NMR (101 MHz, CDCl_3_) *δ* 166.93, 151.53, 145.55, 142.63, 136.51, 134.35, 132.95, 132.03, 128.90, 127.22, 124.22, 39.63, 36.79. HRMS-ESI (m/z): [M + H]^+^ calcd for C_13_H_10_Cl_2_N_4_O: 309.0304, found: 309.0303.

##### 2-Chloro-5,10,11-trimethyl-5,11-dihydro-6*H*-benzo[*e*]pyrimido[5,4-*b*][1,4]diazepin-6-one (5-2)

Yellow solid; yield 68%; m.p. 188–189 °C; ^1^H NMR (400 MHz, CDCl_3_) *δ* 7.88 (d, *J* = 8.0 Hz, 1H), 7.32 (d, *J* = 7.4 Hz, 1H), 7.04 (s, 1H), 6.99 (t, *J* = 7.6 Hz, 1H), 3.74 (s, 3H), 3.38 (s, 3H), 2.34 (s, 3H). ^13^C NMR (101 MHz, CDCl_3_) *δ* 168.07, 149.81, 145.19, 143.70, 136.49, 135.37, 134.93, 131.26, 128.80, 125.75, 124.16, 39.56, 36.36, 19.77, 0.03. HRMS-ESI (m/z): [M + H]^+^ calcd for C_14_H_13_ClN_4_O: 289.0851, found: 289.0847.

##### 2,9-Dichloro-5,11-dimethyl-5,11-dihydro-6*H*-benzo[*e*]pyrimido[5,4-*b*][1,4]diazepin-6-one (5-3)

White solid; yield 100%; m.p. 165–166 °C; ^1^H NMR (400 MHz, CDCl_3_) *δ* 8.28 (s, 1H), 7.81 (d, *J* = 8.4 Hz, 1H), 7.17 (dd, *J* = 8.4, 1.9 Hz, 1H), 7.11 (d, *J* = 1.9 Hz, 1H), 3.52 (s, 3H), 3.43 (s, 3H). ^13^C NMR (101 MHz, CDCl_3_) *δ* 166.74, 163.33, 154.90, 151.60, 149.27, 139.30, 133.89, 128.32, 124.62, 124.13, 118.35, 38.29, 36.35. HRMS-ESI (m/z): [M + H]^+^ calcd for C_13_H_10_Cl_2_N_4_O: 309.0304, found: 309.0302.

##### 2-Chloro-5,9,11-trimethyl-5,11-dihydro-6*H*-benzo[*e*]pyrimido[5,4-*b*][1,4]diazepin-6-one (5-4)

White solid; yield 100%; m.p. 162–163 °C; ^1^H NMR (400 MHz, CDCl_3_) *δ* 8.23 (s, 1H), 7.76 (d, *J* = 7.9 Hz, 1H), 7.00 (d, *J* = 8.0 Hz, 0H), 6.90 (s, 1H), 3.51 (s, 3H), 3.43 (s, 3H), 2.39 (s, 3H). ^13^C NMR (101 MHz, CDCl_3_) *δ* 167.69, 163.94, 154.60, 151.17, 148.26, 143.94, 132.57, 128.55, 125.25, 123.00, 118.47, 38.27, 36.26, 21.67. HRMS-ESI (m/z): [M + H]^+^ calcd for C_14_H_13_ClN_4_O: 289.0851, found: 289.0849.

##### 2,8-Dichloro-5,11-dimethyl-5,11-dihydro-6*H*-benzo[*e*]pyrimido[5,4-*b*][1,4]diazepin-6-one (5-5)

White solid; yield 96%; m.p. 179–180 °C; ^1^H NMR (400 MHz, CDCl_3_) *δ* 8.27 (s, 1H), 7.82 (d, *J* = 2.6 Hz, 1H), 7.42 (dd, *J* = 8.9, 2.6 Hz, 1H), 7.05 (d, *J* = 8.8 Hz, 1H), 3.52 (s, 3H), 3.41 (s, 3H). ^13^C NMR (101 MHz, CDCl_3_) *δ* 166.37, 163.54, 154.88, 151.62, 146.88, 132.81, 132.18, 129.96, 128.16, 127.17, 119.40, 38.47, 36.36. HRMS-ESI (m/z): [M + H]^+^ calcd for C_13_H_10_Cl_2_N_4_O: 309.0304, found: 309.0305.

##### 2-Chloro-8-fluoro-5,11-dimethyl-5,11-dihydro-6*H*-benzo[*e*]pyrimido[5,4-*b*][1,4]diazepin-6-one (5-6)

White solid; yield 92%; m.p. 168–169 °C; ^1^H NMR (400 MHz, CDCl_3_) *δ* 8.25 (s, 1H), 7.53 (dd, *J* = 8.7, 3.1 Hz, 1H), 7.17 (m, 1H), 7.07 (dd, *J* = 9.1, 4.4 Hz, 1H), 3.51 (s, 3H), 3.39 (s, 3H).^13^C NMR (101 MHz, CDCl_3_) *δ* 166.41, 163.84, 160.19, 157.76, 154.84, 151.53, 144.44, 128.23, 127.48, 120.03, 118.86, 38.50, 36.44. HRMS-ESI (m/z): [M + H]^+^ calcd for C_13_H_10_ClFN_4_O: 293.0600, found: 293.0596.

##### 2-Chloro-5,11-dimethyl-8-(trifluoromethyl)-5,11-dihydro-6*H*-benzo[*e*]pyrimido[5,4-*b*][1,4]diazepin-6-one(5-7)

White solid; yield 90%; m.p. = 167–168 °C; ^1^H NMR (400 MHz, CDCl_3_) *δ* 8.31 (s, 1H), 8.15 (d, *J* = 2.3 Hz, 1H), 7.72 (dd, *J* = 8.7, 2.3 Hz, 1H), 7.23 (d, *J* = 8.7 Hz, 1H), 3.55 (s, 3H), 3.48 (s, 3H). ^13^C NMR (101 MHz, CDCl_3_) *δ* 166.39, 163.10, 155.01, 151.87, 151.14, 130.25, 129.71, 128.12, 126.80, 126.08, 124.78, 118.47, 38.36, 36.47. HRMS-ESI (m/z): [M + H]^+^ calcd for C_14_H_10_ClF_3_N_4_O: 343.0568, found: 343.0565.

##### 2-Chloro-5,8,11-trimethyl-5,11-dihydro-6*H*-benzo[*e*]pyrimido[5,4-*b*][1,4]diazepin-6-one (5-8)

White solid; yield 100%; m.p. 173–174 °C; ^1^H NMR (400 MHz, CDCl_3_) *δ* 8.22 (s, 1H), 7.65 (d, *J* = 2.2 Hz, 1H), 7.27 (dd, *J* = 8.3, 2.8 Hz, 1H), 6.99 (d, *J* = 8.4 Hz, 1H), 3.51 (s, 3H), 3.40 (s, 3H), 2.33 (s, 3H). ^13^C NMR (101 MHz, CDCl_3_) *δ* 167.85, 164.09, 154.62, 151.20, 145.98, 134.15, 133.72, 132.65, 128.42, 125.54, 117.87, 38.45, 36.17, 20.38. HRMS-ESI (m/z): [M + H]^+^ calcd for C_14_H_13_ClN_4_O: 289.0851, found: 289.0848.

##### 2-Chloro-7-fluoro-5,11-dimethyl-5,11-dihydro-6*H*-benzo[*e*]pyrimido[5,4-*b*][1,4]diazepin-6-one (5-9)

White solid; yield 97%; m.p. 172–173 °C; ^1^H NMR (400 MHz, CDCl_3_) *δ* 8.33 (s, 1H), 7.40 (m, 1H), 6.98–6.88 (m, 2H), 3.53 (s, 3H), 3.41 (s, 3H). ^13^C NMR (101 MHz, CDCl_3_) *δ* 164.28, 163.50, 160.78, 154.81, 152.27, 150.56, 132.60, 128.49, 115.63, 113.67, 112.81, 37.96, 36.32. HRMS-ESI (m/z): [M + H]^+^ calcd for C_13_H_10_ClFN_4_O: 293.0600, found: 293.0597.

##### 2-Chloro-5,7,11-trimethyl-5,11-dihydro-6*H*-benzo[*e*]pyrimido[5,4-*b*][1,4]diazepin-6-one (5-10)

White solid; yield 81%; m.p. 132–133 °C; ^1^H NMR (400 MHz, CDCl_3_) *δ* 8.29 (s, 1H), 7.29 (t, *J* = 8.1 Hz, 1H), 7.05 (d, *J* = 7.5 Hz, 1H), 6.99 (d, *J* = 8.3 Hz, 1H), 3.53 (s, 3H), 3.39 (s, 3H), 2.45 (s, 3H). ^13^C NMR (101 MHz, CDCl_3_) *δ* 167.91, 165.01, 154.59, 151.69, 149.62, 140.86, 130.81, 128.96, 127.81, 126.28, 115.68, 38.29, 36.00, 21.08. HRMS-ESI (m/z): [M + H]^+^ calcd for C_14_H_13_ClN_4_O: 289.0851, found: 289.0848.

#### General procedure for synthesis of compound A1–A10 and B1–B3

A sealed tube with a mixture of **5-1–5-10** or **9-1–9-3** (1 eq), 1–(4-amino-3-ethoxyphenyl)piperidin-4-ol (1 eq), Pd(OAc)_2_ (0.05 eq), XPhos (0.1 eq), and Cs_2_CO_3_ (2 eq) in *t*-BuOH (5.0 ml) was heated at 110 °C for 8–16 h. The reaction was then filtered through celite and eluted with dichloromethane. The dichloromethane was removed *in vacuo* and the resulting crude product was purified by silica gel column chromatography (eluant: DCM/MeOH, 40:1 v/v) to afford the target product (15–41%).

##### 10-Chloro-2-((2-ethoxy-4-(4-hydroxypiperidin-1-yl)phenyl)amino)-5,11-dimethyl-5,11-dihydro-6*H*-benzo[*e*]pyrimido[5,4-*b*][1,4]diazepin-6-one(A1)

Yellow solid; yield 21%; m.p. 130–131 °C; ^1^H NMR (400 MHz, CDCl_3_) *δ* 8.32 (d, *J* = 8.6 Hz, 1H), 8.15 (s, 1H), 7.68 (dd, *J* = 7.7, 1.6 Hz, 1H), 7.52 (s, 1H), 7.45 (dd, *J* = 8.0, 1.7 Hz, 1H), 7.15 (t, *J* = 7.8 Hz, 1H), 6.65–6.56 (m, 2H), 4.13 (q, *J* = 6.9 Hz, 2H), 3.90–3.83 (m, 1H), 3.65 (s, 3H), 3.54–3.47 (m, 5H), 2.95–2.87 (m, 2H), 2.10–2.00 (m, 2H), 1.81–1.72 (m, 2H), 1.49 (t, *J* = 7.0 Hz, 3H). ^13^C NMR (101 MHz, CDCl_3_) *δ* 167.62, 163.36, 156.20, 151.67, 148.04, 147.11, 144.56, 134.46, 132.83, 129.92, 128.70, 126.04, 122.38, 121.63, 119.04, 108.72, 102.09, 67.85, 64.23, 48.46, 38.56, 38.45, 34.43, 14.99. HRMS-ESI (m/z): [M + H]^+^ calcd for C_26_H_29_ClN_6_O_3_: 509.2062, found: 509.2059.

##### 2-((2-Ethoxy-4-(4-hydroxypiperidin-1-yl)phenyl)amino)-5,10,11-trimethyl-5,11-dihydro-6*H*-benzo[*e*]pyrimido[5,4-*b*][1,4]diazepin-6-one(A2)

Brown solid; yield 28%; m.p. 177–178 °C; ^1^H NMR (400 MHz, CDCl_3_) *δ* 7.98 (d, *J* = 8.5 Hz, 1H), 7.86 (d, *J* = 7.9 Hz, 1H), 7.28 (s, 1H), 7.22 (s, 1H), 6.95–6.87 (m, 2H), 6.60–6.55 (m, 2H), 4.13 (q, *J* = 7.0 Hz, 2H), 3.89–3.82 (m, 1H), 3.73 (s, 3H), 3.53–3.47 (s, 2H), 3.37 (s, 3H), 2.96–2.85 (m, 2H), 2.36 (s, 3H), 2.08–1.99 (m, 2H), 1.77–1.67 (m, 2H), 1.49 (t, *J* = 7.0 Hz, 3H). ^13^C NMR (101 MHz, CDCl_3_) *δ* 168.97, 152.28, 149.03, 148.59, 148.50, 146.33, 139.66, 135.64, 134.21, 130.99, 126.07, 122.61, 121.94, 121.77, 120.19, 108.75, 101.62, 67.74, 64.22, 48.11, 39.93, 34.25, 29.70, 20.05, 15.00. HRMS-ESI (m/z): [M + H]^+^ calcd for C_27_H_32_N_6_O_3_: 489.2609, found: 489.2608.

##### 9-Chloro-2-((2-ethoxy-4-(4-hydroxypiperidin-1-yl)phenyl)amino)-5,11-dimethyl-5,11-dihydro-6*H*-benzo[*e*]pyrimido[5,4-*b*][1,4]diazepin-6-one(A3)

Brown solid; yield 31%; m.p. 162–163 °C; ^1^H NMR (400 MHz, CDCl_3_) *δ* 8.22 (d, *J* = 8.5 Hz, 1H), 8.14 (s, 1H), 7.80 (d, *J* = 8.4 Hz, 1H), 7.50 (s, 1H), 7.14–7.07 (m, 2H), 6.63–6.58 (m, 2H), 4.12 (q, *J* = 6.9 Hz, 2H), 3.91–3.82 (m, 1H), 3.54–3.47 (m, 5H), 3.41 (s, 3H), 2.95–2.87 (m, 2H), 2.06–2.01 (m, 2H), 1.81–1.70 (m, 2H), 1.48 (t, *J* = 6.9 Hz, 3H). ^13^C NMR (101 MHz, CDCl_3_) *δ* 167.42, 163.13, 156.15, 151.75, 150.41, 148.18, 147.20, 138.41, 133.53, 124.95, 123.81, 122.19, 120.51, 119.20, 117.75, 108.59, 102.15, 67.75, 64.25, 48.43, 38.11, 35.97, 34.36, 14.97. HRMS-ESI (m/z): [M + H]^+^ calcd for C_26_H_29_ClN_6_O_3_: 509.2062, found: 509.2063.

##### 2-((2-Ethoxy-4-(4-hydroxypiperidin-1-yl)phenyl)amino)-5,9,11-trimethyl-5,11-dihydro-6*H*-benzo[*e*]pyrimido[5,4-*b*][1,4]diazepin-6-one(A4)

White solid; yield 40%; m.p. 171–172 °C; ^1^H NMR (400 MHz, CDCl_3_) *δ* 8.26 (d, *J* = 8.6 Hz, 1H), 8.11 (s, 1H), 7.75 (d, *J* = 7.9 Hz, 1H), 7.48 (s, 1H), 6.95 (d, *J* = 7.9 Hz, 1H), 6.89 (s, 1H), 6.64–6.55 (m, 2H), 4.12 (q, *J* = 7.0 Hz, 2H), 3.90–3.83 (m, 1H), 3.55–3.44 (m, 5H), 3.42 (s, 3H), 2.94–2.86 (m, 2H), 2.36 (s, 3H), 2.08–2.01 (m, 2H), 1.80–1.70 (m, 2H), 1.48 (t, *J* = 7.0 Hz, 3H). ^13^C NMR (101 MHz, CDCl_3_) *δ* 168.38, 163.75, 156.04, 151.33, 149.39, 148.07, 147.09, 142.98, 132.21, 124.50, 123.75, 122.44, 120.87, 119.05, 117.91, 108.61, 102.17, 67.78, 64.23, 48.48, 38.11, 35.91, 34.40, 21.64, 14.98. HRMS-ESI (m/z): [M + H]^+^ calcd for C_27_H_32_N_6_O_3_: 489.2609, found: 489.2602.

##### 8-Chloro-2-((2-ethoxy-4-(4-hydroxypiperidin-1-yl)phenyl)amino)-5,11-dimethyl-5,11-dihydro-6*H*-benzo[*e*]pyrimido[5,4-*b*][1,4]diazepin-6-one(A5)

Yellow solid; yield 28%; m.p. 173–174 °C; ^1^H NMR (400 MHz, CDCl_3_) *δ* 8.21 (d, *J* = 8.6 Hz, 1H), 8.12 (s, 1H), 7.81 (s, 1H), 7.50 (s, 1H), 7.35 (d, *J* = 8.9 Hz, 1H), 7.00 (d, *J* = 9.1 Hz, 1H), 6.60 (s, 1H), 6.58 (s, 1H), 4.11 (q, *J* = 7.0 Hz, 2H), 3.89–3.80 (m, 1H), 3.52–3.44 (m, 5H), 3.38 (s, 3H), 2.93–2.83 (m, 2H), 2.07–2.00 (m, 2H), 1.79–1.68 (m, 2H), 1.47 (t, *J* = 7.0 Hz, 3H). ^13^C NMR (101 MHz, CDCl_3_) *δ* 167.05, 163.40, 156.12, 151.75, 148.17, 148.05, 147.28, 132.05, 131.82, 129.06, 127.92, 122.10, 120.31, 119.19, 118.82, 108.55, 102.09, 67.67, 64.24, 48.41, 38.31, 35.98, 34.35, 14.97. HRMS-ESI (m/z): [M + H]^+^ calcd for C_26_H_29_ClN_6_O_3_: 509.2062, found: 509.2061.

##### 2-((2-Ethoxy-4-(4-hydroxypiperidin-1-yl)phenyl)amino)-8-fluoro-5,11-dimethyl-5,11-dihydro-6*H*-benzo[*e*]pyrimido[5,4-*b*][1,4]diazepin-6-one(A6)

Yellow solid; yield 23%; m.p. 166–167 °C; ^1^H NMR (400 MHz, CDCl_3_) *δ* 8.21 (d, *J* = 8.5 Hz, 1H), 8.11 (s, 1H), 7.57–7.49 (m, 2H), 7.14–7.06 (m, 1H), 7.02 (dd, *J* = 9.2, 4.4 Hz, 1H), 6.61–6.56 (m, 2H), 4.10 (q, *J* = 6.9 Hz, 2H), 3.87–3.78 (m, 1H), 3.53–3.42 (m, 5H), 3.37 (s, 3H), 2.92–2.83 (m, 2H), 2.06–1.98 (m, 2H), 1.78–1.68 (m, 2H), 1.46 (t, *J* = 7.0 Hz, 3H). ^13^C NMR (101 MHz, CDCl_3_) *δ* 167.13, 163.75, 159.87, 157.45, 156.13, 151.63, 148.16, 147.27, 145.62, 145.59, 128.06, 122.12, 120.39, 119.20, 118.21, 108.55, 102.09, 67.58, 64.24, 48.44, 38.35, 36.06, 34.34, 14.97. HRMS-ESI (m/z): [M + H]^+^ calcd for C_26_H_29_FN_6_O_3_: 493.2358, found: 493.2353.

##### 2-((2-Ethoxy-4-(4-hydroxypiperidin-1-yl)phenyl)amino)-5,11-dimethyl-8-(trifluoromethyl)-5,11-dihydro-6*H*-benzo[*e*]pyrimido[5,4-*b*][1,4]diazepin-6-one(A7)

Yellow solid; yield 15%; m.p. 119–120 °C; ^1^H NMR (400 MHz, CDCl_3_) *δ* 8.27–8.12 (m, 3H), 7.66 (d, *J* = 8.7 Hz, 1H), 7.52 (s, 1H), 7.19 (d, *J* = 8.7 Hz, 1H), 6.61 (s, 1H), 6.59 (s, 1H), 4.12 (q, *J* = 6.9 Hz, 2H), 3.90–3.83 (m, 1H), 3.60–3.44 (m, 8H), 2.96–2.87 (m, 2H), 2.10–1.99 (m, 2H), 1.79–1.74 (m, 2H), 1.48 (t, *J* = 6.9 Hz, 3H). ^13^C NMR (101 MHz, CDCl_3_) *δ* 167.08, 162.91, 156.18, 152.26, 152.00, 148.22, 147.34, 129.88, 129.56, 128.95, 126.79, 125.95, 122.02, 120.21, 119.22, 117.82, 108.53, 102.09, 67.76, 64.25, 48.37, 38.21, 36.09, 34.35, 14.96. HRMS-ESI (m/z): [M + H]^+^ calcd for C_27_H_29_F_3_N_6_O_3_: 543.2326, found: 543.2322.

##### 2-((2-Ethoxy-4-(4-hydroxypiperidin-1-yl)phenyl)amino)-5,8,11-trimethyl-5,11-dihydro-6*H*-benzo[*e*]pyrimido[5,4-*b*][1,4]diazepin-6-one(A8)

Yellow solid; yield 41%; m.p. 170–171 °C; ^1^H NMR (400 MHz, CDCl_3_) *δ* 8.23 (d, *J* = 8.5 Hz, 1H), 8.08 (s, 1H), 7.62 (d, *J* = 2.2 Hz, 1H), 7.48 (s, 1H), 7.20 (dd, *J* = 8.5, 2.2 Hz, 1H), 6.95 (d, *J* = 8.4 Hz, 1H), 6.60–6.56 (m, 2H), 4.09 (q, *J* = 7.0 Hz, 2H), 3.86–3.77 (m, 1H), 3.49–3.44 (m, 5H), 3.37 (s, 3H), 2.90–2.82 (m, 2H), 2.30 (s, 3H), 2.04–1.97 (m, 2H), 1.78–1.67 (m, 2H), 1.45 (t, *J* = 7.0 Hz, 3H). ^13^C NMR (101 MHz, CDCl_3_) *δ* 168.52, 163.95, 156.01, 151.33, 148.08, 147.15, 147.13, 133.21, 133.01, 132.29, 126.22, 122.34, 120.68, 119.09, 117.28, 108.60, 102.14, 67.57, 64.23, 48.49, 38.29, 35.82, 34.36, 20.38, 14.97. HRMS-ESI (m/z): [M + H]^+^ calcd for C_27_H_32_N_6_O_3_: 489.2609, found: 489.2602.

##### 2-((2-Ethoxy-4-(4-hydroxypiperidin-1-yl)phenyl)amino)-7-fluoro-5,11-dimethyl-5,11-dihydro-6*H*-benzo[*e*]pyrimido[5,4-*b*][1,4]diazepin-6-one(A9)

Yellow solid; yield 23%; m.p. 174–175 °C; ^1^H NMR (400 MHz, CDCl_3_) *δ* 8.21 (d, *J* = 8.9 Hz, 1H), 8.18 (s, 1H), 7.51 (s, 1H), 7.37–7.29 (m, 1H), 6.91–6.83 (m, 2H), 6.59 (s, 1H), 6.57 (s, 1H), 4.11 (q, *J* = 6.9 Hz, 2H), 3.89–3.80 (m, 1H), 3.54–3.44 (m, 5H), 3.38 (s, 3H), 2.93–2.85 (m, 2H), 2.10–2.00 (m, 2H), 1.79–1.68 (m, 2H), 1.47 (t, *J* = 7.0 Hz, 3H). ^13^C NMR (101 MHz, CDCl_3_) *δ* 164.20, 163.31, 160.77, 156.05, 152.42, 151.85, 148.17, 147.30, 131.93, 122.05, 120.52, 119.21, 116.01, 113.10, 111.99, 108.53, 102.05, 67.67, 64.23, 48.39, 37.80, 35.98, 34.34, 14.96. HRMS-ESI (m/z): [M + H]^+^ calcd for C_26_H_29_FN_6_O_3_: 493.2358, found: 493.2354.

##### 2-((2-Ethoxy-4-(4-hydroxypiperidin-1-yl)phenyl)amino)-5,7,11-trimethyl-5,11-dihydro-6*H*-benzo[*e*]pyrimido[5,4-*b*][1,4]diazepin-6-one(A10)

Yellow solid; yield 25%; m.p. = 184–185 °C; ^1^H NMR (400 MHz, CDCl_3_) *δ* 8.25 (d, *J* = 8.6 Hz, 1H), 8.16 (s, 1H), 7.48 (s, 1H), 7.22 (t, *J* = 7.9 Hz, 1H), 6.98 (dd, *J* = 12.0, 7.9 Hz, 2H), 6.64–6.55 (m, 2H), 4.12 (q, *J* = 7.0 Hz, 2H), 3.89–3.81 (m, 1H), 3.53–3.46 (m, 5H), 3.37 (s, 3H), 2.94–2.86 (m, 2H), 2.47 (s, 3H), 2.10–1.99 (m, 2H), 1.80–1.69 (m, 2H), 1.48 (t, *J* = 7.0 Hz, 3H). ^13^C NMR (101 MHz, CDCl_3_) *δ* 168.46, 164.94, 155.99, 151.90, 150.75, 148.09, 147.13, 140.40, 130.12, 127.03, 126.90, 122.38, 121.12, 119.11, 115.04, 108.63, 102.15, 67.77, 64.24, 48.45, 38.12, 35.67, 34.40, 21.06, 14.97. HRMS-ESI (m/z): [M + H]^+^ calcd for C_27_H_32_N_6_O_3_: 489.2609, found: 489.2606.

##### 2-((2-Ethoxy-4-(4-hydroxypiperidin-1-yl)phenyl)amino)-11-methyl-5,11-dihydro-6*H*-benzo[*e*]pyrimido[5,4-*b*][1,4]diazepin-6-one(B1)

Yellow solid; yield 29%; m.p. 165–166 °C; ^1^H NMR (400 MHz, CDCl_3_) *δ* 8.83 (s, 1H), 8.26 (d, *J* = 8.6 Hz, 1H), 8.01 (s, 1H), 7.91 (dd, *J* = 7.8, 1.7 Hz, 1H), 7.54–7.48 (m, 1H), 7.47 (s, 1H), 7.20–7.09 (m, 2H), 6.63–6.57 (m, 2H), 4.12 (q, *J* = 7.0 Hz, 2H), 3.90–3.83 (m, 1H), 3.53–3.47 (m, 2H), 3.46 (s, 3H), 2.94–2.85 (m, 2H), 2.10–2.01 (m, 2H), 1.79–1.73 (m, 2H), 1.48 (t, *J* = 7.0 Hz, 3H). ^13^C NMR (101 MHz, CDCl_3_) *δ* 169.51, 161.75, 156.61, 149.25, 149.08, 148.02, 146.96, 133.33, 132.05, 125.53, 123.65, 122.60, 118.93, 118.71, 115.15, 108.64, 102.22, 67.84, 64.22, 48.56, 36.67, 34.41, 14.99. HRMS-ESI (m/z): [M + H]^+^ calcd for C_25_H_28_N_6_O_3_: 461.2296, found: 461.2293.

##### 2-((2-Ethoxy-4-(4-hydroxypiperidin-1-yl)phenyl)amino)-5-methyl-5,11-dihydro-6*H*-benzo[*e*]pyrimido[5,4-*b*][1,4]diazepin-6-one(B2)

Yellow solid; yield 20%; m.p. 194–195 °C; ^1^H NMR (400 MHz, CDCl_3_) *δ* 8.16–8.09 (m, 2H), 7.95 (dd, *J* = 7.9, 1.6 Hz, 1H), 7.39–7.30 (m, 2H), 7.07 (t, *J* = 7.6 Hz, 1H), 6.81 (d, *J* = 8.0 Hz, 1H), 6.57 (s, 1H), 6.55 (s, 1H), 6.39 (s, 1H), 4.08 (q, *J* = 7.0 Hz, 2H), 3.88–3.81 (m, 1H), 3.49–3.45 (m, 5H), 2.91–2.83 (m, 2H), 2.06–1.99 (m, 2H), 1.74–1.70 (m, 2H), 1.44 (t, *J* = 7.0 Hz, 3H). ^13^C NMR (101 MHz, CDCl_3_) *δ* 167.61, 160.63, 156.30, 151.81, 148.40, 147.45, 145.07, 133.27, 132.86, 123.46, 123.18, 121.96, 119.63, 119.10, 118.89, 108.57, 102.15, 67.79, 64.19, 48.41, 38.51, 34.37, 14.97. HRMS-ESI (m/z): [M + H]^+^ calcd for C_25_H_28_N_6_O_3_: 461.2296, found: 461.2292.

##### 2-((2-Ethoxy-4-(4-hydroxypiperidin-1-yl)phenyl)amino)-5-ethyl-11-methyl-5,11-dihydro-6*H*-benzo[*e*]pyrimido[5,4-*b*][1,4]diazepin-6-one(B3)

Brown solid; yield 25%; m.p. 162–163 °C; ^1^H NMR (400 MHz, CDCl_3_) *δ* 8.25 (d, *J* = 8.7 Hz, 1H), 8.19 (s, 1H), 7.80 (dd, *J* = 7.8, 1.7 Hz, 1H), 7.50 (s, 1H), 7.40 (t, *J* = 7.9 Hz, 1H), 7.13 (t, *J* = 7.7 Hz, 1H), 7.07 (d, *J* = 8.4 Hz, 1H), 6.62–6.57 (m, 2H), 4.12 (q, *J* = 6.9 Hz, 2H), 3.99 (q, *J* = 6.9 Hz, 2H), 3.87–3.81 (m, 1H), 3.52–3.46 (m, 2H), 3.41 (s, 3H), 2.93–2.84 (m, 2H), 2.06–2.01 (m, 2H), 1.78–1.70 (m, 2H), 1.47 (t, *J* = 7.0 Hz, 3H), 1.30 (t, *J* = 6.9 Hz, 3H). ^13^C NMR (101 MHz, CDCl_3_) *δ* 168.26, 164.80, 156.05, 151.89, 149.62, 148.08, 147.15, 132.02, 131.89, 127.29, 123.72, 122.30, 119.27, 119.05, 117.27, 108.59, 102.12, 67.73, 64.20, 48.52, 46.19, 35.79, 34.37, 14.99, 13.87. HRMS-ESI (m/z): [M + H]^+^ calcd for C_27_H_32_N_6_O_3_: 489.2609, found: 489.2603.

#### General procedure for synthesis of compounds C1 and C2

The compounds **C1** and **C2** are synthesised by a similar method using the above-described procedure for the reaction of compound **A1**.

##### 2-((4-(4-Hydroxypiperidin-1-yl)cyclohexyl)amino)-5,11-dimethyl-5,11-dihydro-6*H*-benzo[*e*]pyrimido[5,4-*b*][1,4]diazepin-6-one(C1)

Yellow solid; yield 45%; m.p. 203–204 °C; ^1^H NMR (400 MHz, CDCl_3_) *δ* 7.99 (s, 1H), 7.82 (dd, *J* = 7.8, 1.6 Hz, 1H), 7.40 (td, *J* = 7.9, 1.7 Hz, 1H), 7.11 (t, *J* = 7.5 Hz, 1H), 7.04 (d, *J* = 8.3 Hz, 1H), 4.08–4.03 (m, 1H), 3.82–3.62 (m, 3H), 3.45 (s, 3H), 3.29 (s, 3H), 2.96–2.87 (m, 2H), 2.46–2.37 (m, 2H), 2.00–1.93 (m, 4H), 1.78–1.73 (m, 2H), 1.64–1.58 (m, 4H). ^13^C NMR (101 MHz, CDCl_3_) *δ* 168.40, 164.19, 158.13, 151.46, 149.54, 132.20, 132.17, 126.59, 123.53, 117.20, 67.28, 62.30, 46.54, 46.22, 38.22, 35.42, 34.19, 29.18, 23.62. HRMS-ESI (m/z): [M + H]^+^ calcd for C_24_H_32_N_6_O_2_: 437.2660, found: 437.2657.

##### 2-((6-(4-Hydroxypiperidin-1-yl)pyridin-3-yl)amino)-5,11-dimethyl-5,11-dihydro-6*H*-benzo[*e*]pyrimido[5,4-*b*][1,4]diazepin-6-one(C2)

Yellow solid; yield 62%; m.p. 189–190 °C; ^1^H NMR (400 MHz, Methanol-d4) *δ* 8.55 (d, *J* = 2.3 Hz, 1H), 8.32 (s, 1H), 8.18 (dd, *J* = 9.9, 2.4 Hz, 1H), 7.83 (dd, *J* = 7.9, 1.5 Hz, 1H), 7.65–7.57 (m, 1H), 7.54 (d, *J* = 9.8 Hz, 1H), 7.34 (d, *J* = 8.3 Hz, 1H), 7.30 (t, *J* = 7.5 Hz, 1H), 4.09–4.01 (m, 3H), 3.68–3.60 (m, 2H), 3.56 (s, 3H), 3.52 (s, 3H), 2.12–2.04 (m, 2H), 1.79–1.68 (m, 2H). ^13^C NMR (101 MHz, CD_3_OD) *δ* 167.73, 166.34, 150.90, 149.47, 146.33, 142.22, 139.36, 133.09, 131.78, 127.68, 125.59, 125.04, 124.80, 121.19, 118.90, 113.44, 64.77, 43.93, 38.04, 36.46, 32.72. HRMS-ESI (m/z): [M + H]^+^ calcd for C_23_H_25_N_7_O_2_: 432.2142, found: 432.2137.

#### Preparation of intermediate 14

A sealed tube with a mixture of **13** (1 eq), 2-Methoxy-4-nitroaniline (1 eq), Pd(OAc)_2_ (0.05 eq), XPhos (0.1 eq), and Cs_2_CO_3_ (2 eq) in *t*-BuOH was heated at 110 °C for 10 h. The reaction was then filtered through celite and eluted with dichloromethane. The dichloromethane was removed *in vacuo* and the resulting crude product was purified by silica gel column chromatography (eluant: DCM/MeOH, 40:1 v/v) to afford the title intermediate **14** (36%).

##### 2-((2-Methoxy-4-nitrophenyl)amino)-5,11-dimethyl-5,11-dihydro-6*H*-benzo[*e*]pyrimido[5,4-*b*][1,4]diazepin-6-one(14)

Yellow solid; yield 36%; m.p. 215–216 °C; ^1^H NMR (400 MHz, CDCl_3_) *δ* 8.71 (d, *J* = 9.1 Hz, 1H), 8.23 (s, 1H), 8.01 (dd, *J* = 9.0, 2.3 Hz, 2H), 7.88 (dd, *J* = 7.8, 1.8 Hz, 1H), 7.78 (d, *J* = 2.5 Hz, 1H), 7.51–7.44 (m, 1H), 7.18 (td, *J* = 7.5, 1.0 Hz, 1H), 7.13 (dd, *J* = 8.4, 1.0 Hz, 1H), 4.05 (s, 3H), 3.54 (s, 3H), 3.48 (s, 3H). ^13^C NMR (101 MHz, CDCl_3_) *δ* 168.07, 163.65, 154.71, 151.14, 149.03, 146.73, 141.17, 135.67, 132.60, 132.44, 126.37, 124.01, 123.04, 118.07, 117.50, 115.56, 105.09, 56.33, 38.30, 36.07. HRMS-ESI (m/z): [M + H]^+^ calcd for C_20_H_18_N_6_O_4_: 407.1462, found: 407.1461.

#### Preparation of intermediate 15

A mixture of **14** (1 eq), Pd/C (0.05 eq), and methanol was stirred at room temperature for 12 h under a hydrogen atmosphere. The reaction mixture was filtered and the residue obtained by evaporation of the solvent of the filtrate under reduced pressure. This residue is prepared as the hydrochloride salt to afford the intermediate **15** (100%).

##### 2-((4-Amino-2-methoxyphenyl)amino)-5,11-dimethyl-5,11-dihydro-6*H*-benzo[*e*]pyrimido[5,4-*b*][1,4]diazepin-6-one(15)

Brown solid; yield 100%; m.p. 199–200 °C; ^1^H NMR (400 MHz, Methanol-d4) *δ* 8.28 (s, 1H), 8.14–8.07 (m, 1H), 7.85 (dd, *J* = 7.8, 1.6 Hz, 1H), 7.66–7.60 (m, 1H), 7.37 (d, *J* = 8.3 Hz, 1H), 7.33 (t, *J* = 7.7 Hz, 1H), 7.21 (s, 1H), 7.16 (dd, *J* = 8.5, 2.3 Hz, 1H), 4.02 (s, 3H), 3.58 (s, 3H), 3.51 (s, 3H). ^13^C NMR (101 MHz, CD_3_OD) *δ* 167.49, 167.19, 152.44, 149.74, 145.43, 138.96, 133.23, 131.85, 128.83, 125.51, 125.43, 125.08, 120.65, 119.19, 114.98, 106.56, 55.73, 38.19, 36.51. HRMS-ESI (m/z): [M + H]^+^ calcd for C_20_H_20_N_6_O_2_: 377.1721, found: 377.1715.

#### General procedure for synthesis of compounds D1 and D2

To a solution of **18** (1 eq) and triethylamine (2 eq) in DCM ethyl 3-chloro-3-oxopropanoate/ethyl 4-chloro-4-oxobutanoate (1.1 eq) in DCM was added dropwise. The reaction mixture was stirred at RT for 1.5 h. After the reaction was complete as monitored by TLC, water was added to the solution. The organic phase is washed with saturated NaHCO_3_ and dried over anhydrous Na_2_SO_4_. After filtration, the combined organic solution is concentrated to dryness under reduced pressure. The crude product was dissolved in THF and treated with 2 N LiOH. The reaction was stirred for 1 h. The solution was added with 1 N HCl to make the pH = 6. This mixture was then washed with EtOAc (three times), and the combined extracts were washed with brine, dried with Na_2_SO_4_, and evaporated under reduced pressure to afford the crude product. The resulting crude product was purified by silica gel column chromatography (eluant: DCM/MeOH, 40:1 v/v) to afford the compound **D1**, **D2** (70%).

##### 3-((4-((5,11-Dimethyl-6-oxo-6,11-dihydro-5*H*-benzo[*e*]pyrimido[5,4-*b*][1,4]diazepin-2-yl)amino)-3-methoxyphenyl)amino)-3-oxopropanoic acid(D1)

White solid; yield 70%; m.p. 187–188 °C; ^1^H NMR (400 MHz, DMSO-d6) *δ* 12.59 (br, 1H), 10.11 (s, 1H), 8.35 (s, 1H), 8.05 (s, 1H), 7.94 (d, *J* = 8.7 Hz, 1H), 7.68 (dd, *J* = 7.7, 1.7 Hz, 1H), 7.52–7.45 (m, 1H), 7.40 (d, *J* = 2.2 Hz, 1H), 7.23 (d, *J* = 8.4, 1.0 Hz, 1H), 7.19–7.12 (m, 2H), 3.81 (s, 3H), 3.39 (s, 3H), 3.35 (s, 2H), 3.29 (s, 3H). ^13^C NMR (101 MHz, DMSO) *δ* 169.75, 167.52, 164.73, 163.50, 152.76, 150.03, 149.70, 135.15, 132.90, 132.11, 126.65, 124.43, 123.89, 121.50, 120.89, 118.21, 111.17, 103.05, 56.05, 44.45, 37.90, 35.95. HRMS-ESI (m/z): [M + H]^+^ calcd for C_23_H_22_N_6_O_5_: 463.1724, found: 463.1726.

##### 4-((4-((5,11-Dimethyl-6-oxo-6,11-dihydro-5*H*-benzo[*e*]pyrimido[5,4-*b*][1,4]diazepin-2-yl)amino)-3-methoxyphenyl)amino)-4-oxobutanoic acid(D2)

White solid; yield 70%; m.p. 237–238 °C; 1H NMR (400 MHz, DMSO-d6) *δ* 12.12 (s, 1H), 9.94 (s, 1H), 8.34 (s, 1H), 8.03 (s, 1H), 7.90 (d, *J* = 8.6 Hz, 1H), 7.68 (dd, *J* = 7.8, 1.7 Hz, 1H), 7.53–7.45 (m, 1H), 7.45 (d, *J* = 2.2 Hz, 1H), 7.23 (dd, *J* = 8.4, 1.0 Hz, 1H), 7.19–7.14 (m, 1H), 7.12 (dd, *J* = 8.7, 2.2 Hz, 1H), 3.80 (s, 3H), 3.39 (s, 3H), 3.28 (s, 3H), 2.60–2.52 (m, 4H). ^13^C NMR (101 MHz, DMSO) *δ* 174.30, 170.25, 167.52, 163.50, 156.53, 152.76, 150.08, 149.71, 135.63, 132.90, 132.10, 126.66, 123.95, 123.89, 121.58, 120.82, 118.20, 110.96, 102.97, 56.01, 37.89, 35.93, 31.48, 29.28. HRMS-ESI (m/z): [M + H]^+^ calcd for C_24_H_24_N_6_O_5_: 477.1881, found: 477.1875.

#### Preparation of intermediate 16

The intermediate **16** are synthesised by a similar method using the above-described procedure for the reaction of compounds **D1** and **D2**.

##### 6-Bromo-*N*-(4-((5,11-dimethyl-6-oxo-6,11-dihydro-5*H*-benzo[*e*]pyrimido[5,4-*b*][1,4]diazepin-2-yl)amino)-3-methoxyphenyl)hexanamide(16)

White solid; yield 87%; m.p. 137–138 °C; ^1^H NMR (400 MHz, CDCl_3_) *δ* 8.30 (d, *J* = 8.4 Hz, 2H), 8.11 (s, 1H), 7.82 (d, *J* = 7.8 Hz, 1H), 7.59 (s, 1H), 7.51 (s, 1H), 7.40 (t, *J* = 7.8 Hz, 1H), 7.10 (t, *J* = 7.5 Hz, 1H), 7.05 (d, *J* = 8.3 Hz, 1H), 6.97 (d, *J* = 8.7 Hz, 1H), 3.84 (s, 3H), 3.47 (s, 3H), 3.40–3.34 (m, 5H), 2.35 (t, *J* = 7.5 Hz, 2H), 1.90–1.80 (m, 2H), 1.73 (m, 2H), 1.52–1.43 (m, 2H). ^13^C NMR (101 MHz, CDCl_3_) *δ* 171.37, 168.45, 163.71, 155.85, 151.39, 149.43, 147.94, 132.95, 132.46, 132.17, 126.41, 125.29, 123.69, 121.11, 118.12, 117.44, 111.71, 103.32, 55.78, 38.28, 37.14, 35.87, 33.68, 32.46, 27.77, 24.71. HRMS-ESI (m/z): [M + H]^+^ calcd for C_26_H_29_BrN_6_O_3_: 553.1557, found: 553.1553.

#### General procedure for the synthesis of compound D8

A mixture of **16** (1 eq) and potassium ethanethioate (6 eq) in DMF was heated to 100 °C for 6 h. After the reaction was complete as monitored by TLC, the solvent was diluted with water (20 ml) and extracted with EtOAc (20 ml) × 3. The organics were combined, dried over Na_2_SO_4_, filtered, and concentrated *in vacuo*. Purification by silica gel column chromatography (eluant: DCM/MeOH, 50:1 v/v) gave compound **D8** (72%).

##### *S*-(6-((4-((5,11-dimethyl-6-oxo-6,11-dihydro-5*H*-benzo[*e*]pyrimido[5,4-*b*][1,4]diazepin-2-yl)amino)-3-methoxyphenyl)amino)-6-oxohexyl) ethanethioate(D8)

Brown solid; yield 72%; m.p. 159–160 °C; ^1^H NMR (400 MHz, CDCl_3_) *δ* 8.35 (d, *J* = 8.7 Hz, 1H), 8.14 (s, 1H), 7.86 (dd, *J* = 7.8, 1.7 Hz, 1H), 7.60 (s, 1H), 7.56 (d, *J* = 2.3 Hz, 1H), 7.46–7.40 (m, 1H), 7.38 (s, 1H), 7.15 (t, *J* = 7.5 Hz, 1H), 7.09 (d, *J* = 8.3 Hz, 1H), 6.87 (dd, *J* = 8.7, 2.3 Hz, 1H), 4.10 (t, *J* = 6.7 Hz, 2H), 3.92 (s, 3H), 3.51 (s, 3H), 3.42 (s, 3H), 2.39 (t, *J* = 7.4 Hz, 2H), 2.06 (s, 3H), 1.84–1.76 (m, 2H), 1.75–1.66 (m, 2H), 1.53–1.40 (m, 2H). ^13^C NMR (101 MHz, CDCl_3_) *δ* 171.23, 170.91, 168.34, 163.75, 155.82, 151.35, 149.40, 148.01, 132.53, 132.37, 132.26, 126.53, 125.51, 123.71, 121.26, 118.08, 117.38, 111.36, 103.18, 64.26, 55.84, 38.25, 37.46, 35.88, 28.42, 25.61, 25.14, 21.02. HRMS-ESI (m/z): [M + H]^+^ calcd for C_28_H_32_N_6_O_4_S: 549.2279, found: 549.2286.

#### Preparation of intermediate 18

A mixture of **17** (1.5 eq), 5-fluoro-2-nitrophenyl ethyl ether (1 eq), and K_2_CO_3_ in DMF was heated to 90 °C for 8–10 h. After the reaction was complete as monitored by TLC, the solution was diluted with water (50 ml) and extracted with EtOAc (50 ml) × 3. The organics were combined, dried over Na_2_SO_4_, filtered, and concentrated *in vacuo*. Purification by silica gel column chromatography (eluant: petroleum ether/EtOAc, 5:1 v/v) gave intermediate **18** (62–91%).

##### 4-(3-Ethoxy-4-nitrophenyl)thiomorpholine (18-4)

Yellow solid; yield 90%; m.p. 118–119 °C; ^1^H NMR (400 MHz, CDCl_3_) *δ* 7.98 (d, *J* = 9.3 Hz, 1H), 6.38 (dd, *J* = 9.4, 2.5 Hz, 1H), 6.29 (d, *J* = 2.5 Hz, 1H), 4.15 (q, *J* = 6.9 Hz, 2H), 3.85–3.78 (m, 4H), 2.76–2.68 (m, 4H), 1.51 (t, *J* = 6.9 Hz, 3H). ^13^C NMR (101 MHz, CDCl_3_) *δ* 155.84, 154.33, 129.86, 128.89, 105.77, 98.80, 65.35, 50.44, 25.93, 14.65. HRMS-ESI (m/z): [M + H]^+^ calcd for C_12_H_16_N_2_O_3_S: 269.0954, found: 269.0953.

#### Preparation of intermediate 19

The intermediate **19** are synthesised by similar method using the above-described procedure for the reaction of intermediate **15**.

##### 1-(4-Amino-3-ethoxyphenyl)piperidine-4-carboxylic acid (19-3)

White solid; yield 100%; m.p. 146–147 °C; ^1^H NMR (400 MHz, DMSO-d6) *δ* 7.64 (d, *J* = 14.6 Hz, 1H), 7.51–7.46 (m, 1H), 7.41–7.33 (m, 1H), 4.18 (q, *J* = 7.0 Hz, 2H), 3.66 (s, 2H), 3.56 (s, 4H), 2.24–2.08 (m, 4H), 1.38 (t, *J* = 6.9 Hz, 3H). ^13^C NMR (101 MHz, DMSO) *δ* 175.25, 174.12, 151.96, 123.95, 113.07, 109.98, 106.59, 65.40, 52.27, 37.99, 26.13, 14.79. HRMS-ESI (m/z): [M + H]^+^ calcd for C_14_H_20_N_2_O_3_: 265.1547, found: 265.1544.

#### General procedure for synthesis of compounds D3, D4, D6, D9–D16

The compounds **D3**, **D4**, **D6**, **D9**–**D16** are synthesised by a similar method using the above-described procedure for the reaction of compound **A1**.

##### 1-(4-((5,11-Dimethyl-6-oxo-6,11-dihydro-5*H*-benzo[*e*]pyrimido[5,4-*b*][1,4]diazepin-2-yl)amino)-3-ethoxyphenyl)piperidine-4-carboxylic acid(D3)

White solid; yield 48%; m.p. 159–160 °C; ^1^H NMR (400 MHz, Methanol-d4) *δ* 8.38 (d, *J* = 8.8 Hz, 1H), 8.32 (s, 1H), 7.80 (dd, *J* = 7.8, 1.7 Hz, 1H), 7.63–7.57 (m, 1H), 7.46 (s, 1H), 7.41 (dd, *J* = 8.8, 2.5 Hz, 1H), 7.32 (d, *J* = 8.5 Hz, 1H), 7.28 (t, *J* = 7.5 Hz, 1H), 4.30 (q, *J* = 6.9 Hz, 2H), 3.88–3.72 (m, 5H), 3.53 (s, 3H), 3.50 (s, 3H), 2.45–2.38 (m, 2H), 2.33–2.26 (m, 2H), 1.51 (t, *J* = 7.0 Hz, 3H). ^13^C NMR (101 MHz, CD_3_OD) *δ* 175.52, 174.12, 168.40, 165.84, 152.07, 149.96, 147.16, 144.57, 137.80, 133.26, 131.70, 128.29, 125.47, 124.65, 121.05, 118.59, 112.76, 104.89, 65.34, 55.50, 55.38, 38.07, 37.14, 36.06, 25.84, 25.74, 13.61. HRMS-ESI (m/z): [M + H]^+^ calcd for C_27_H_30_N_6_O_4_: 503.2401, found: 503.2397.

##### 2-((2-Ethoxy-4-thiomorpholinophenyl)amino)-5,11-dimethyl-5,11-dihydro-6*H*-benzo[*e*]pyrimido[5,4-*b*][1,4]diazepin-6-one(D4)

Yellow solid; yield 38%; m.p. 190–191 °C; ^1^H NMR (400 MHz, CDCl_3_) *δ* 8.28 (d, *J* = 8.8 Hz, 1H), 8.13 (s, 1H), 7.86 (dd, *J* = 7.8, 1.7 Hz, 1H), 7.50 (s, 1H), 7.46–7.40 (m, 1H), 7.15 (t, *J* = 7.5 Hz, 1H), 7.10 (d, *J* = 8.3 Hz, 1H), 6.58 (dd, *J* = 8.8, 2.6 Hz, 1H), 6.54 (d, *J* = 2.5 Hz, 1H), 4.12 (q, *J* = 7.0 Hz, 2H), 3.51 (s, 3H), 3.49–3.44 (m, 4H), 3.43 (s, 3H), 2.85–2.77 (m, 4H), 1.49 (t, *J* = 7.0 Hz, 3H). 13 C NMR (101 MHz, CDCl_3_) *δ* 168.35, 163.77, 156.01, 151.47, 149.48, 148.15, 147.49, 132.29, 132.25, 126.60, 123.66, 122.97, 120.86, 119.06, 117.36, 109.55, 102.99, 64.29, 53.41, 38.23, 35.90, 27.45, 14.96. HRMS-ESI (m/z): [M + H]^+^ calcd for C_25_H_28_N_6_O_2_S: 477.2067, found: 477.2065.

##### 2-((4-(1,1-Dioxidothiomorpholino)-2-ethoxyphenyl)amino)-5,11-dimethyl-5,11-dihydro-6*H*-benzo[*e*]pyrimido[5,4-*b*][1,4]diazepin-6-one(D6)

White solid; yield 32%; m.p. 157–158 °C; ^1^H NMR (400 MHz, Methanol-d4) *δ* 8.12 (s, 1H), 7.83 (dd, *J* = 7.8, 1.7 Hz, 1H), 7.69–7.62 (m, 1H), 7.52 (s, 1H), 7.40–7.31 (m, 2H), 6.80–6.70 (m, 2H), 4.16 (q, *J* = 7.0 Hz, 2H), 3.99–3.89 (m, 4H), 3.55 (s, 3H), 3.49 (s, 3H), 3.25–3.20 (m, 4H), 1.35 (t, *J* = 6.9 Hz, 3H). ^13^C NMR (101 MHz, CD_3_OD) *δ* 168.01, 167.57, 145.24, 133.56, 131.79, 125.25, 125.07, 119.95, 119.23, 107.95, 101.15, 64.33, 49.99, 47.17, 38.65, 36.48, 13.81. HRMS-ESI (m/z): [M + H]^+^ calcd for C_25_H_28_N_6_O_4_S: 509.1966, found: 509.1962.

##### 2-((2-Ethoxy-4-(4-hydroxy-4-methylpiperidin-1-yl)phenyl)amino)-5-ethyl-11-methyl-5,11-dihydro-6*H*-benzo[*e*]pyrimido[5,4-*b*][1,4]diazepin-6-one(D9)

White solid; yield 30%; m.p. 178–179 °C; ^1^H NMR (400 MHz, CDCl_3_) *δ* 8.25 (d, *J* = 8.7 Hz, 1H), 8.18 (s, 1H), 7.80 (d, *J* = 7.8 Hz, 1H), 7.50 (s, 1H), 7.40 (t, *J* = 7.8 Hz, 1H), 7.12 (t, *J* = 7.5 Hz, 1H), 7.06 (d, *J* = 8.3 Hz, 1H), 6.66–6.56 (m, 2H), 4.26–3.71 (m, 4H), 3.40 (s, 3H), 3.32–3.22 (m, 2H), 3.21–3.09 (m, 2H), 1.85–1.70 (m, 4H), 1.46 (t, *J* = 7.0 Hz, 3H), 1.38–1.22 (m, 7H). ^13^C NMR (101 MHz, CDCl_3_) *δ* 168.24, 164.80, 156.10, 151.89, 149.63, 148.14, 147.36, 132.00, 131.89, 127.30, 123.69, 122.17, 119.23, 119.17, 117.26, 108.66, 102.06, 77.45, 77.13, 76.81, 67.65, 64.23, 47.16, 46.16, 38.59, 35.76, 29.87, 14.98, 13.85. HRMS-ESI (m/z): [M + H]^+^ calcd for C_28_H_34_N_6_O_3_: 503.2771, found: 503.2767.

##### 2-((2-Ethoxy-4-(4-fluoro-4-methylpiperidin-1-yl)phenyl)amino)-5-ethyl-11-methyl-5,11-dihydro-6*H*-benzo[*e*]pyrimido[5,4-*b*][1,4]diazepin-6-one(D10)

White solid; yield 33%; m.p. 189–190 °C; ^1^H NMR (400 MHz, CDCl_3_) *δ* 8.28 (d, *J* = 8.7 Hz, 1H), 8.20 (s, 1H), 7.82 (d, *J* = 7.7 Hz, 1H), 7.51 (s, 1H), 7.40 (t, *J* = 7.8 Hz, 1H), 7.14 (t, *J* = 7.6 Hz, 1H), 7.07 (d, *J* = 8.3 Hz, 1H), 6.67–6.57 (m, 2H), 4.21–3.75 (m, 4H), 3.47–3.33 (m, 5H), 3.11 (t, *J* = 11.9 Hz, 2H), 2.01–1.77 (m, 4H), 1.53–1.37 (m, 6H), 1.31 (t, *J* = 7.1 Hz, 3H). ^13^C NMR (101 MHz, CDCl_3_) *δ* 168.20, 164.78, 156.09, 151.90, 149.63, 148.14, 147.10, 131.98, 131.90, 127.35, 123.69, 122.44, 119.34, 119.15, 117.25, 108.83, 102.18, 92.92, 91.25, 77.41, 77.09, 76.78, 64.24, 46.97, 46.94, 46.14, 36.45, 36.24, 35.76, 26.88, 14.97, 13.85. HRMS-ESI (m/z): [M + H]^+^ calcd for C_28_H_33_FN_6_O_2_: 505.2702, found: 505.2719.

##### 2-((2-Methoxy-4-(piperidin-1-yl)phenyl)amino)-5,11-dimethyl-5,11-dihydro-6*H*-benzo[*e*]pyrimido[5,4-*b*][1,4]diazepin-6-one(D11)

White solid; yield 45%; m.p. 166–167 °C; ^1^H NMR (400 MHz, CDCl_3_) *δ* 8.22 (d, *J* = 9.3 Hz, 1H), 8.13 (s, 1H), 7.86 (dd, *J* = 7.8, 1.7 Hz, 1H), 7.48–7.38 (m, 2H), 7.19–7.05 (m, 2H), 6.60 (d, *J* = 7.4 Hz, 2H), 3.90 (s, 3H), 3.51 (s, 3H), 3.42 (s, 3H), 3.17–3.08 (m, 4H), 1.81–1.72 (m, 4H), 1.65–1.55 (m, 2H). ^13^C NMR (101 MHz, CDCl_3_) *δ* 168.36, 163.77, 156.19, 151.47, 149.52, 148.92, 148.34, 132.23, 126.64, 123.60, 121.95, 120.76, 119.32, 117.33, 108.56, 101.21, 77.33, 77.02, 76.70, 55.65, 51.83, 38.19, 35.84, 26.06, 24.28. HRMS-ESI (m/z): [M + H]^+^ calcd for C_25_H_28_N_6_O_2_: 445.2352, found: 445.2346.

##### 2-((2-Methoxy-4-(4-methoxypiperidin-1-yl)phenyl)amino)-5,11-dimethyl-5,11-dihydro-6*H*-benzo[*e*]pyrimido[5,4-*b*][1,4]diazepin-6-one(D12)

White solid; yield 47%; m.p. 179–180 °C; ^1^H NMR (400 MHz, CDCl_3_) *δ* 8.22 (d, *J* = 8.5 Hz, 1H), 8.12 (s, 1H), 7.86 (dd, *J* = 7.8, 1.8 Hz, 1H), 7.47–7.38 (m, 2H), 7.18–7.05 (m, 2H), 6.64–6.56 (m, 2H), 3.90 (s, 3H), 3.54–3.44 (m, 5H), 3.43–3.34 (m, 7H), 2.97–2.86 (m, 2H), 2.12–2.00 (m, 2H), 1.81–1.70 (m, 2H). ^13^C NMR (101 MHz, CDCl_3_) *δ* 168.34, 163.76, 156.15, 151.46, 149.50, 148.91, 147.42, 132.25, 132.23, 126.62, 123.60, 122.17, 120.81, 119.32, 117.33, 108.64, 101.19, 77.36, 77.04, 76.73, 76.06, 55.67, 55.59, 48.40, 38.19, 35.84, 30.84. HRMS-ESI (m/z): [M + H]^+^ calcd for C_26_H_30_N_6_O_3_: 475.2458, found: 475.2450.

##### 2-((4-(4-Fluoropiperidin-1-yl)-2-methoxyphenyl)amino)-5,11-dimethyl-5,11-dihydro-6*H*-benzo[*e*]pyrimido[5,4-*b*][1,4]diazepin-6-one(D13)

White solid; yield 33%; m.p. 160–161 °C; ^1^H NMR (400 MHz, CDCl_3_) *δ* 8.17 (s, 1H), 8.15 (s, 1H), 7.87 (dd, *J* = 7.8, 1.7 Hz, 1H), 7.49–7.39 (m, 2H), 7.20–7.06 (m, 2H), 6.56 (s, 1H), 5.86–5.71 (m, 1H), 5.08–4.97 (m, 2H), 4.40 (s, 2H), 3.90 (s, 3H), 3.52 (s, 3H), 3.45 (s, 3H), 3.24 (t, *J* = 7.4 Hz, 2H), 2.29 (q, *J* = 7.2 Hz, 2H). ^13^C NMR (101 MHz, CDCl_3_) *δ* 168.34, 163.76, 156.20, 151.56, 149.47, 148.14, 136.49, 132.27, 132.26, 126.66, 123.67, 122.44, 120.71, 120.55, 117.27, 115.93, 101.15, 77.35, 77.03, 76.71, 55.76, 51.76, 38.22, 35.85, 32.64. HRMS-ESI (m/z): [M + H]^+^ calcd for C_25_H_27_FN_6_O_2_: 463.2252, found: 463.2261.

##### 2-((4-(4,4-Difluoropiperidin-1-yl)-2-methoxyphenyl)amino)-5,11-dimethyl-5,11-dihydro-6*H*-benzo[*e*]pyrimido[5,4-*b*][1,4]diazepin-6-one(D14)

White solid; yield 32%; m.p. 171–172 °C; ^1^H NMR (400 MHz, CDCl_3_) *δ* 8.27 (d, *J* = 8.5 Hz, 1H), 8.14 (s, 1H), 7.87 (d, *J* = 7.5 Hz, 1H), 7.51–7.39 (m, 2H), 7.20–7.06 (m, 2H), 6.67–6.56 (m, 2H), 3.91 (s, 3H), 3.55–3.36 (m, 6H), 3.35–3.23 (m, 4H), 2.23–2.05 (m, 4H). ^13^C NMR (101 MHz, CDCl_3_) *δ* 168.30, 163.77, 156.07, 151.44, 149.46, 148.93, 146.07, 132.27, 126.62, 123.65, 122.96, 121.01, 119.26, 117.34, 109.13, 101.51, 77.37, 77.05, 76.74, 55.73, 47.99, 47.94, 47.89, 38.19, 35.85, 34.12, 33.89, 33.67. HRMS-ESI (m/z): [M + H]^+^ calcd for C_25_H_26_F_2_N_6_O_2_: 481.2164, found: 481.2155.

##### 2-((4-(4-Hydroxy-4-methylpiperidin-1-yl)-2-methoxyphenyl)amino)-5,11-dimethyl-5,11-dihydro-6*H*-benzo[*e*]pyrimido[5,4-*b*][1,4]diazepin-6-one(D15)

White solid; yield 37%; m.p. 162–163 °C; ^1^H NMR (400 MHz, CDCl_3_) *δ* 8.22 (d, *J* = 8.6 Hz, 1H), 8.12 (s, 1H), 7.89–7.82 (m, 1H), 7.47–7.38 (m, 2H), 7.19–7.05 (m, 2H), 6.67–6.58 (m, 2H), 3.90 (s, 3H), 3.50 (s, 3H), 3.42 (s, 3H), 3.35–3.25 (m, 2H), 3.23–3.11 (m, 2H), 1.91–1.80 (m, 2H), 1.76–1.71 (m, 2H), 1.33 (s, 3H). ^13^C NMR (101 MHz, CDCl_3_) *δ* 168.40, 163.80, 156.16, 151.46, 149.51, 148.95, 147.48, 132.28, 132.23, 126.59, 123.63, 122.12, 120.79, 119.38, 117.36, 108.72, 101.15, 77.35, 77.03, 76.72, 67.82, 55.69, 47.18, 38.66, 38.23, 35.85, 29.88. HRMS-ESI (m/z): [M + H]^+^ calcd for C_26_H_30_N_6_O_3_: 475.2458, found: 475.2451.

##### 2-((4-(4,4-Dimethylpiperidin-1-yl)-2-methoxyphenyl)amino)-5,11-dimethyl-5,11-dihydro-6*H*-benzo[*e*]pyrimido[5,4-*b*][1,4]diazepin-6-one(D16)

White solid; yield 40%; m.p. 181–182 °C; ^1^H NMR (400 MHz, CDCl_3_) *δ* 8.22 (d, *J* = 8.5 Hz, 1H), 8.12 (s, 1H), 7.90–7.82 (m, 1H), 7.47–7.39 (m, 2H), 7.18–7.04 (m, 2H), 6.66–6.57 (m, 2H), 3.90 (s, 3H), 3.50 (s, 3H), 3.41 (s, 3H), 3.19–3.11 (m, 4H), 1.61–1.53 (m, 4H), 1.02 (s, 6H). ^13^C NMR (101 MHz, CDCl_3_) *δ* 168.35, 163.76, 156.20, 151.48, 149.52, 148.96, 147.94, 132.25, 132.22, 126.63, 123.59, 121.75, 120.73, 119.40, 117.33, 108.22, 100.77, 77.39, 77.07, 76.75, 55.65, 47.03, 38.67, 38.19, 35.84, 28.43, 27.94. HRMS-ESI (m/z): [M + H]^+^ calcd for C_27_H_32_N_6_O_2_: 473.2665, found: 473.2658.

#### General procedure for the synthesis of compound D5

**D4** (1 eq) was dissolved in acetic acid, and hydrogen peroxide (1 eq) was added dropwise. After the reaction was complete as monitored by TLC, the residue was obtained by evaporation of solvent under reduced pressure. Purification by silica gel column chromatography (eluant: DCM/MeOH, 60:1 v/v) gave compound **D5** (95%).

##### 2-((2-ethoxy-4-(1-Oxidothiomorpholino)phenyl)amino)-5,11-dimethyl-5,11-dihydro-6*H*-benzo[*e*]pyrimido[5,4-*b*][1,4]diazepin-6-one(D5)

White solid; yield 95%; m.p. 163–164 °C; ^1^H NMR (400 MHz, Methanol-d4) *δ* 8.19 (s, 1H), 7.83 (dd, *J* = 7.8, 1.7 Hz, 1H), 7.76 (s, 1H), 7.68–7.61 (m, 1H), 7.40–7.29 (m, 2H), 7.08–6.94 (m, 2H), 4.22 (q, *J* = 7.0 Hz, 2H), 4.16–4.05 (m, 2H), 3.83–3.76 (m, 2H), 3.56 (s, 3H), 3.50 (s, 3H), 3.31–3.24 (m, 2H), 3.14–3.04 (m, 2H), 1.40 (t, *J* = 6.9 Hz, 3H). ^13^C NMR (101 MHz, CD_3_OD) *δ* 167.89, 167.31, 152.63, 150.25, 145.43, 138.51, 133.48, 131.80, 125.17, 120.20, 119.19, 109.55, 102.47, 64.61, 43.36, 42.38, 38.53, 36.51, 13.75. HRMS-ESI (m/z): [M + H]^+^ calcd for C_25_H_28_N_6_O_3_S: 493.2016, found: 493.2011.

#### Preparation of intermediate 20

The intermediate **20** are synthesised by a similar method using the above-described procedure for the reaction of compound **A1**.

##### Tert-butyl 4-(4-((5,11-dimethyl-6-oxo-6,11-dihydro-5*H*-benzo[*e*]pyrimido[5,4-*b*][1,4] diazepin-2-yl)amino)-3-ethoxyphenyl)piperazine-1-carboxylate(20)

White solid; yield 29%; m.p. 237–238 °C; ^1^H NMR (400 MHz, CDCl_3_) *δ* 8.29 (d, *J* = 8.5 Hz, 1H), 8.13 (s, 1H), 7.86 (dd, *J* = 7.8, 1.7 Hz, 1H), 7.49 (s, 1H), 7.46–7.40 (m, 1H), 7.14 (t, *J* = 7.5 Hz, 1H), 7.10 (d, *J* = 8.4 Hz, 1H), 6.63–6.54 (m, 2H), 4.12 (q, *J* = 6.9 Hz, 2H), 3.61 (t, *J* = 5.1 Hz, 4H), 3.51 (s, 3H), 3.43 (s, 3H), 3.09 (t, *J* = 5.1 Hz, 4H), 1.52–1.46 (m, 12H). ^13^C NMR (101 MHz, CDCl_3_) *δ* 168.32, 163.75, 156.01, 154.72, 151.46, 149.48, 148.10, 146.92, 132.27, 132.25, 126.61, 123.65, 123.06, 120.87, 119.02, 117.34, 108.68, 102.15, 79.89, 64.27, 50.59, 38.21, 35.89, 28.45, 14.95. HRMS-ESI (m/z): [M + H]^+^ calcd for C_30_H_37_N_7_O_4_: 560.2980, found: 560.2978.

#### Preparation of intermediate 21

A mixture of **21** (1 eq), trifluoroacetic acid (10 eq), and DCM was stirred at room temperature for 1.5 h. After the reaction was complete as monitored by TLC, the residue was obtained by evaporation of solvent under reduced pressure. This residue is prepared as the hydrochloride salt to afford the title intermediate **21** (100%).

##### 2-((2-Ethoxy-4-(piperazin-1-yl)phenyl)amino)-5,11-dimethyl-5,11-dihydro-6*H*-benzo[*e*]pyrimido[5,4-*b*][1,4]diazepin-6-one(21)

Yellow solid; yield 100%; m.p. 185–186 °C; ^1^H NMR (400 MHz, Methanol-d4) *δ* 8.15 (s, 1H), 7.85 (dd, *J* = 7.8, 1.7 Hz, 1H), 7.67–7.56 (m, 1.7 Hz, 2H), 7.38 (d, *J* = 8.0 Hz, 1H), 7.34 (t, *J* = 7.6 Hz, 1H), 6.92 (d, *J* = 2.5 Hz, 1H), 6.84 (dd, *J* = 8.7, 2.5 Hz, 1H), 4.20 (q, *J* = 7.0 Hz, 2H), 3.64–3.59 (m, 4H), 3.57 (s, 3H), 3.50 (s, 3H), 3.49–4.46 (m, 4H), 1.38 (t, *J* = 7.0 Hz, 3H). ^13^C NMR (101 MHz, CD_3_OD) *δ* 167.59, 167.46, 145.13, 137.19, 133.26, 131.81, 125.34, 125.09, 120.16, 119.20, 108.85, 102.33, 64.31, 46.87, 43.07, 38.30, 36.44, 13.68. HRMS-ESI (m/z): [M + H]^+^ calcd for C_25_H_29_N_7_O_2_: 460.2455, found: 460.2449.

#### Preparation of compound D7

A mixture of **21** (1 eq), glucose (1.5 eq), and acetic acid (1 eq) in ethanol was heated to reflux for 8 h. After the reaction was complete as monitored by TLC, the residue was obtained by evaporation of solvent under reduced pressure. Purification by silica gel column chromatography (eluant: DCM/MeOH, 30:1 v/v) gave compound **D7** (82%).

##### 2-((2-Ethoxy-4-(4-(((2*R*,3*S*,4*R*,5*R*)-2,3,4,5-tetrahydroxytetrahydro-2*H*-pyran-2-yl)methyl)piperazin-1-yl)phenyl)amino)-5,11-dimethyl-5,11-dihydro-6*H*-benzo[*e*]pyrimido[5,4-*b*][1,4]diazepin-6-one(D7)

Yellow solid; yield 82%; m.p. 126–127 °C; ^1^H NMR (400 MHz, CDCl_3_) *δ* 8.25 (d, *J* = 8.5 Hz, 1H), 8.12 (s, 1H), 7.85 (dd, *J* = 7.8, 1.7 Hz, 1H), 7.54 (s, 1H), 7.46–7.40 (m, 1H), 7.14 (t, *J* = 7.3 Hz, 1H), 7.09 (d, *J* = 8.3 Hz, 1H), 6.59–5.64 (m, 2H), 4.11 (q, *J* = 7.0 Hz, 2H), 4.03 (d, *J* = 12.4 Hz, 2H), 3.92–3.69 (m, 7H), 3.57 (d, *J* = 9.4 Hz, 1H), 3.50 (s, 3H), 3.42 (s, 3H), 3.19–3.10 (m, 5H), 2.97 (d, *J* = 13.3 Hz, 1H), 2.76–2.66 (m, 2H), 2.08 (s, 1H), 1.47 (t, *J* = 7.0 Hz, 3H). ^13^C NMR (101 MHz, CDCl_3_) *δ* 168.42, 163.82, 155.99, 151.37, 149.45, 148.21, 146.76, 132.34, 132.24, 126.52, 123.69, 122.70, 120.76, 119.22, 117.38, 108.17, 101.68, 96.71, 71.56, 70.89, 69.27, 64.28, 62.79, 61.16, 54.32, 50.42, 38.29, 35.90, 14.95. HRMS-ESI (m/z): [M + H]^+^ calcd for C_31_H_39_N_7_O_7_: 622.2984, found: 622.2996.

### Biological evaluation

#### In vitro *kinase inhibition assay*

Eurofins (Belgium) conducted *in vitro* DCLK1 and LRRK2 kinase activity assays. Compounds were sent as dry powders to Eurofins.

#### In vitro *cytotoxicity assay*

A 96-well plate (3599, Corning, USA) was inoculated with 100 μL of complete medium containing 10 000 cells, and the experimental, control, and blank groups were established. Three parallel wells were used per concentration. After 10 h, media from the experimental and control groups were replaced with a complete medium containing the indicated concentration of the compound. Cells were incubated for 72 h before 10 μL of CCK8 reagent (CA1210, Solarbio, China) was added to each well and incubated for 1 h. The absorbance at 450 nm was measured with a microplate reader (Tecan, Switzerland). The IC_50_ was determined using GraphPad Prism 8 and nonlinear regression curve fitting.

### Molecular modelling

Docking results were carried out by Schrödinger Maestro and Pymol. All parameters follow the default values. The DCLK1 crystal structure was acquired from RCSB Protein Data Bank (http://www.rcsb.org, PDB ID: 5JZN). The compound was prepared with ChemDraw and Schrödinger Maestro.

## Consent form

All authors agree with the submission of the final version for publication in the journal.

## Supplementary Material

Supplemental Material
