# Supplementary material for: Design and synthesis of doublecortin-like kinase 1 inhibitors and their bioactivity evaluation
Source: J Enzyme Inhib Med Chem. 2023 Dec 7;39(1):2287990. doi: 10.1080/14756366.2023.2287990 (PMC11792831; doi:10.1080/14756366.2023.2287990)

SUPPORTING INFORMATION

Design and synthesis of doublecortin-like kinase 1 inhibitors and their bioactivity evaluation

Pengming Pan[a], Dengbo Ji[b], Zhongjun Li[a], and Xiangbao Meng[a*]

[a] *State Key Laboratory of Natural and Biomimetic Drugs, School of Pharmaceutical Sciences, Peking University, Beijing 100191, China;*

[b] *Key Laboratory of Carcinogenesis and Translational Research (Ministry of Education), Department of Gastrointestinal Surgery III, Peking University Cancer Hospital & Institute, Beijing 100142, China*

*Corresponding author Prof. Meng. Tel: 86-010-82801714, E-mail address: xbmeng@bjmu.edu.cn.

Content

1. General Information……………………………………………………S2

2. ^{1}H-NMR, ^{13}C-NMR of target compounds…………………………..S2-S73

1. General Information

All reagents and solvents used were obtained from commercial suppliers without further purification, except for special cases. Reactions were monitored by TLC. Thin layer chromatography was carried out using TLC silica gel 60 F254 plates. Flash column chromatography was performed with 200-300 mesh silica gel. The NMR spectrum was measured by a Bruker-400 NMR spectrometer, with TMS as an internal standard and chemical shifts reported in ppm (δ). Coupling constants (J)are reported in Hz. Spin multiplicities are described as s (singlet), br (broad singlet), d (doublet), t (triplet), q (quartet), and m (multiplet). Melting point was measured by a X-5 micro melting point meter.

2. 1H-NMR, 13C-NMR of target compounds

The $^{1}H/^{13}C$ NMR spectrum of intermediate **2-4**

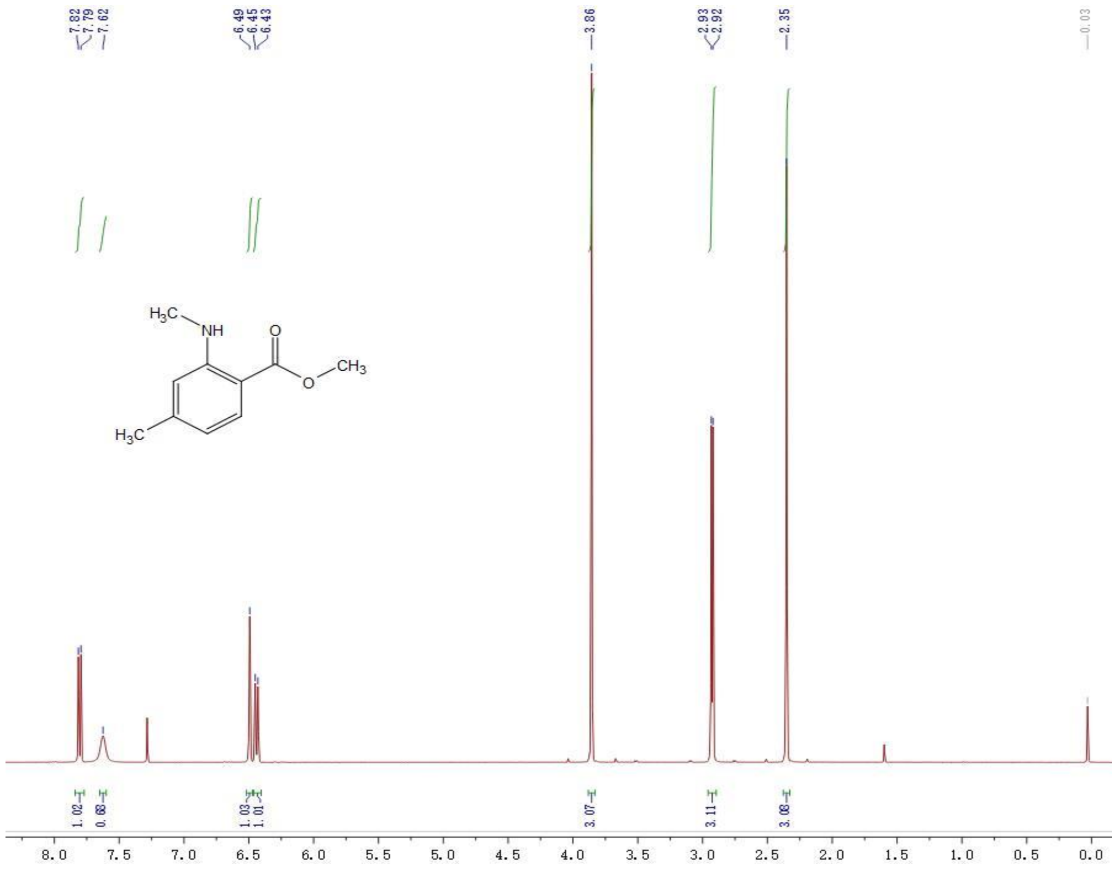

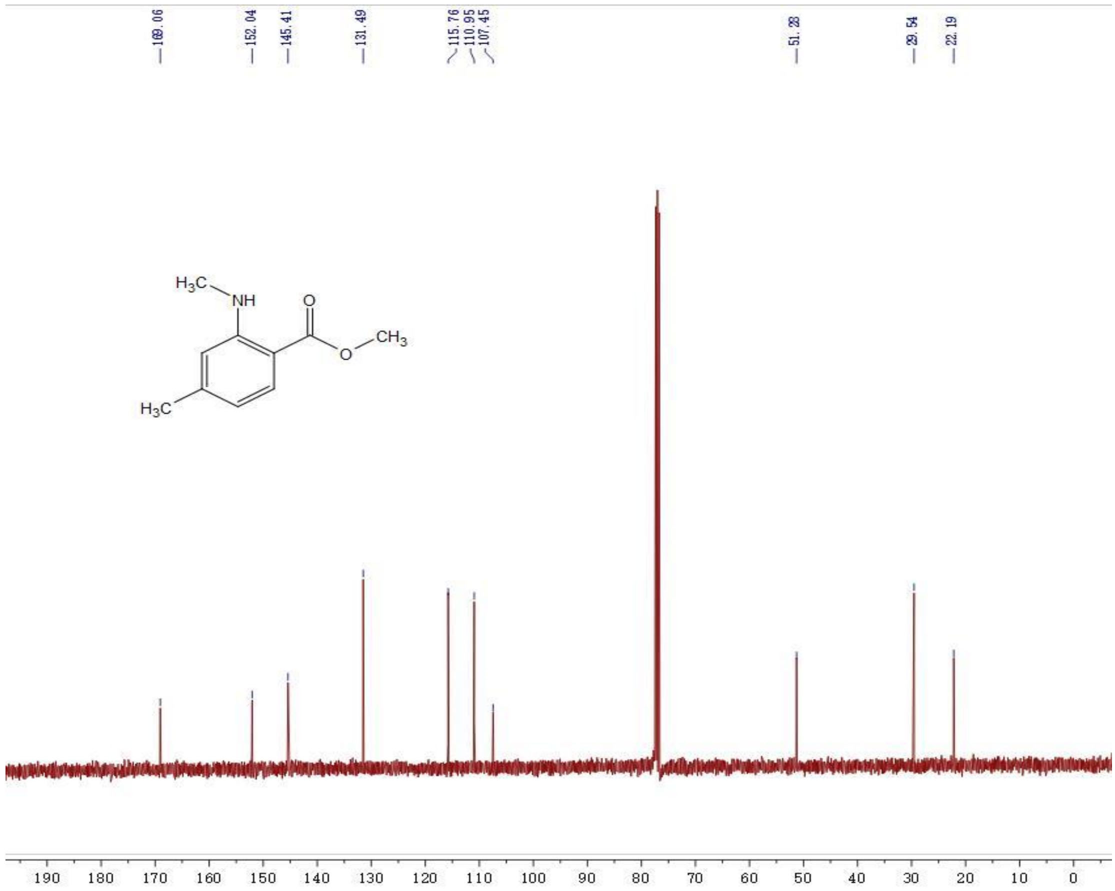

The $^{1}H/^{13}C$ NMR spectrum of intermediate **3-1**

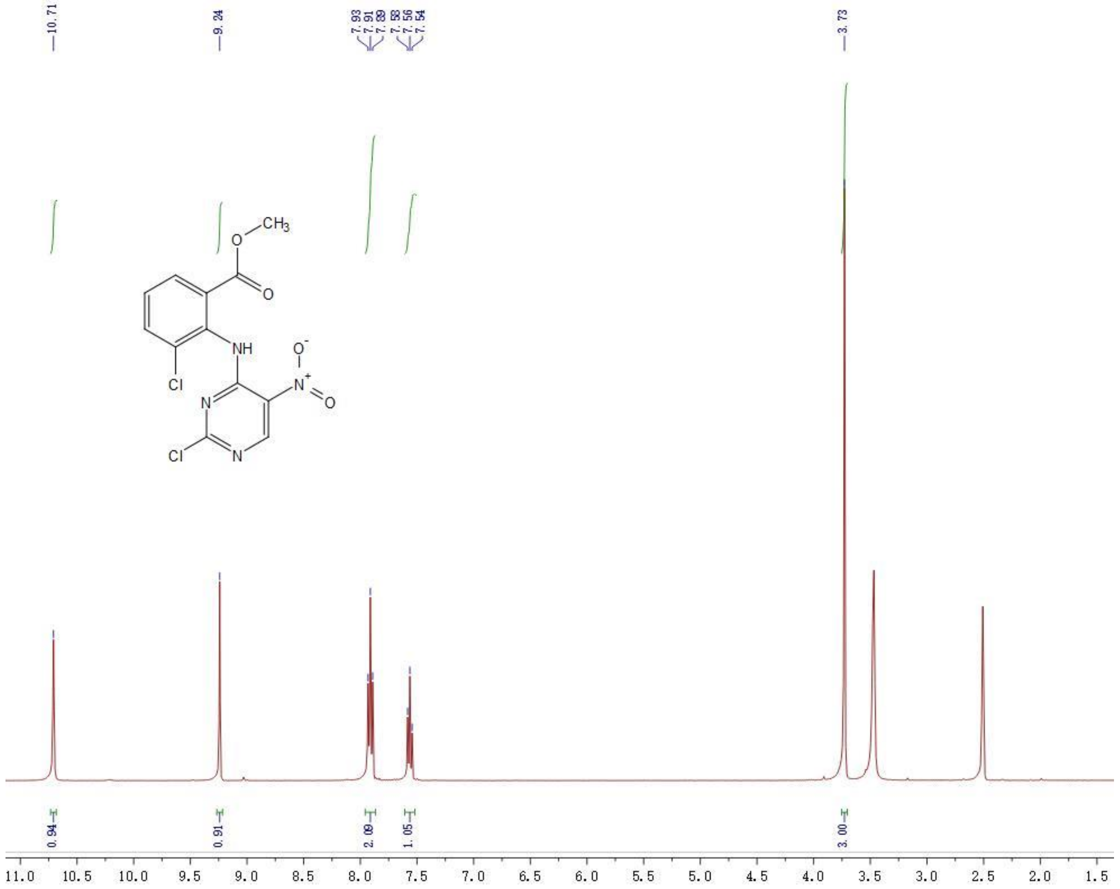

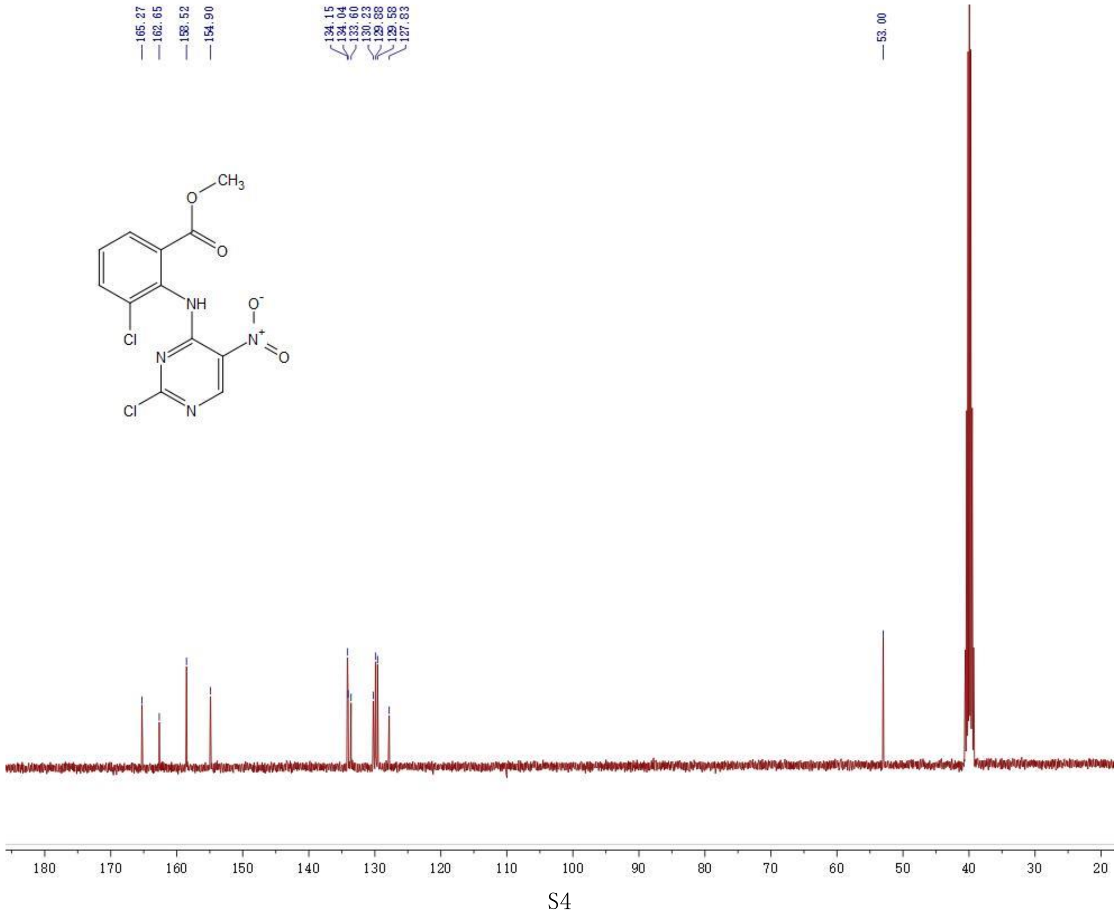

The $^{1}H/^{13}C$ NMR spectrum of intermediate **3-2**

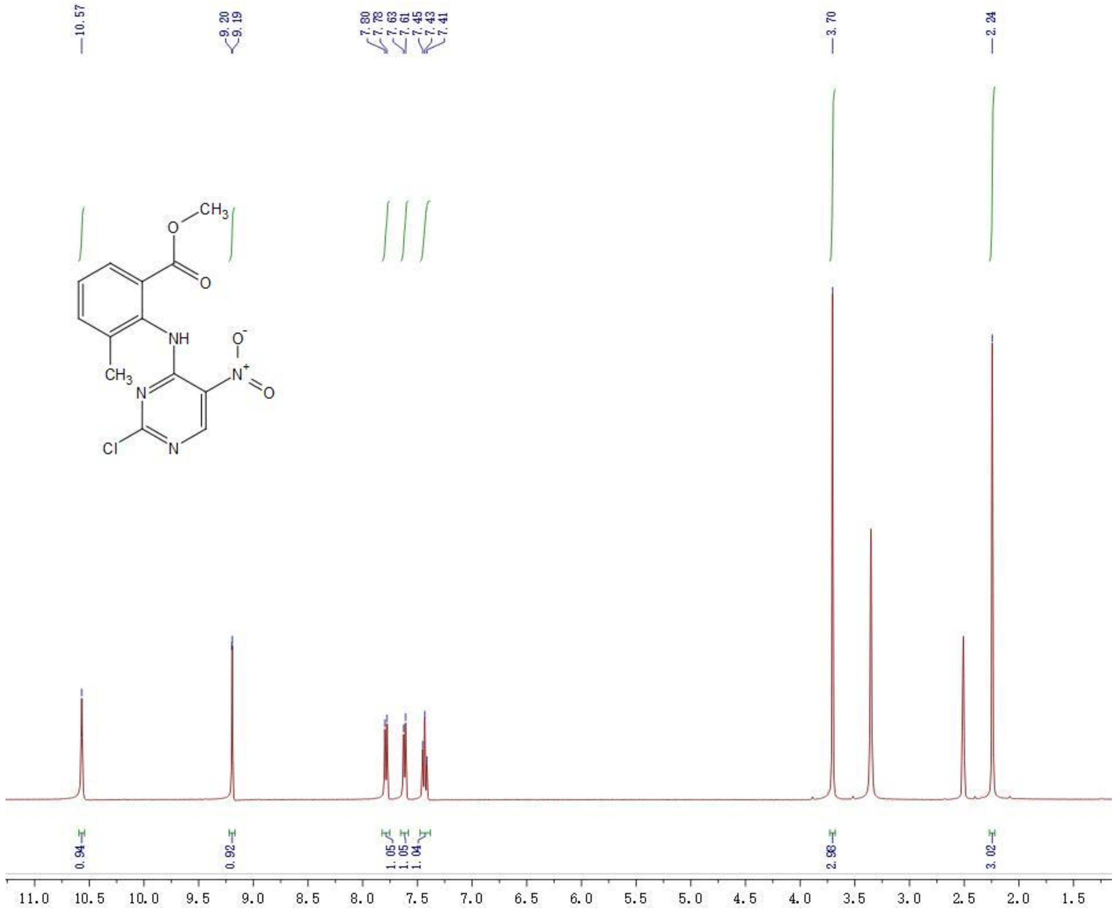

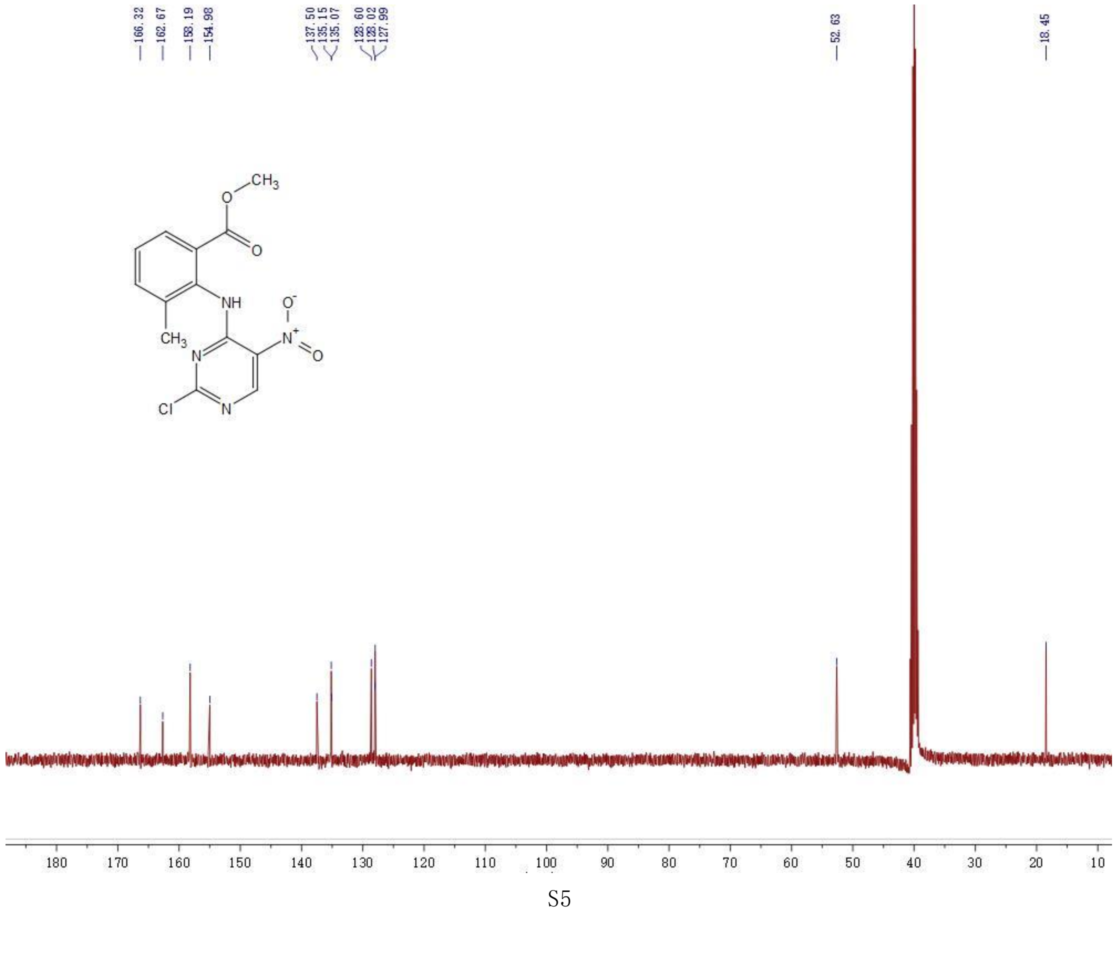

The $^{1}H/^{13}C$ NMR spectrum of intermediate **3-3**

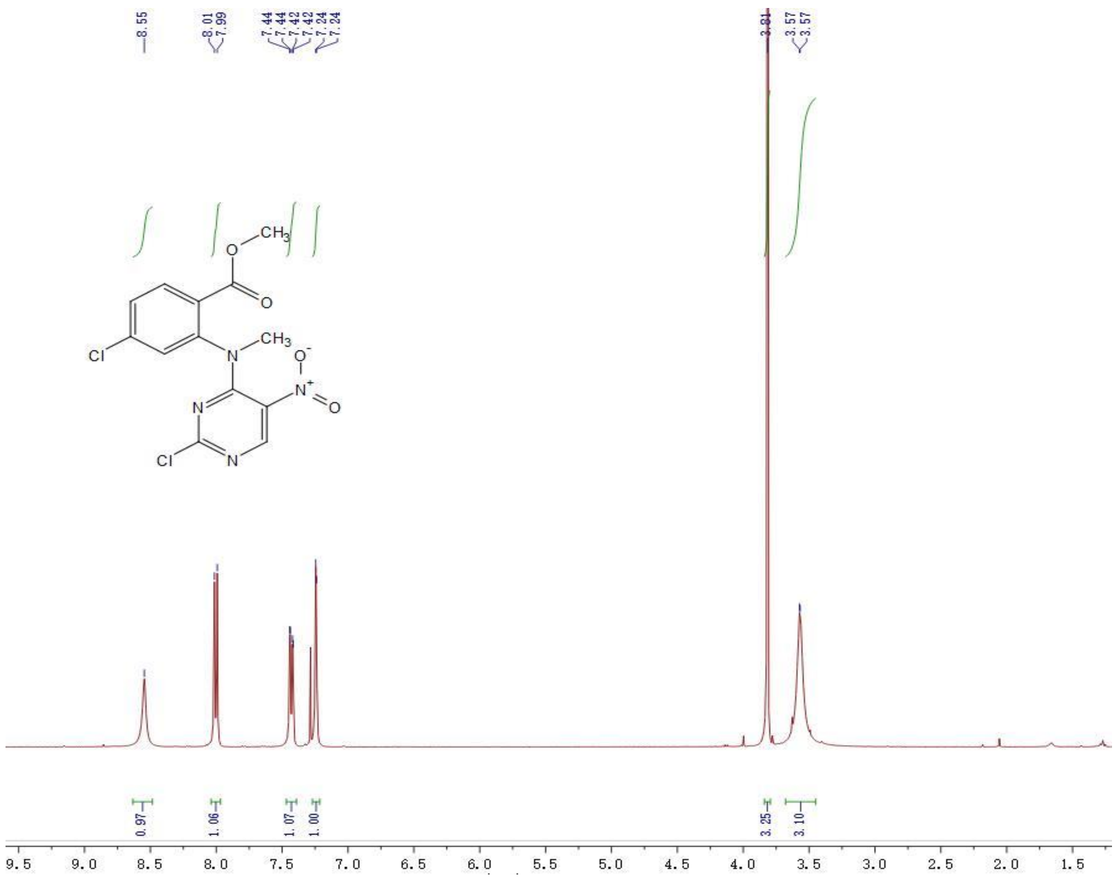

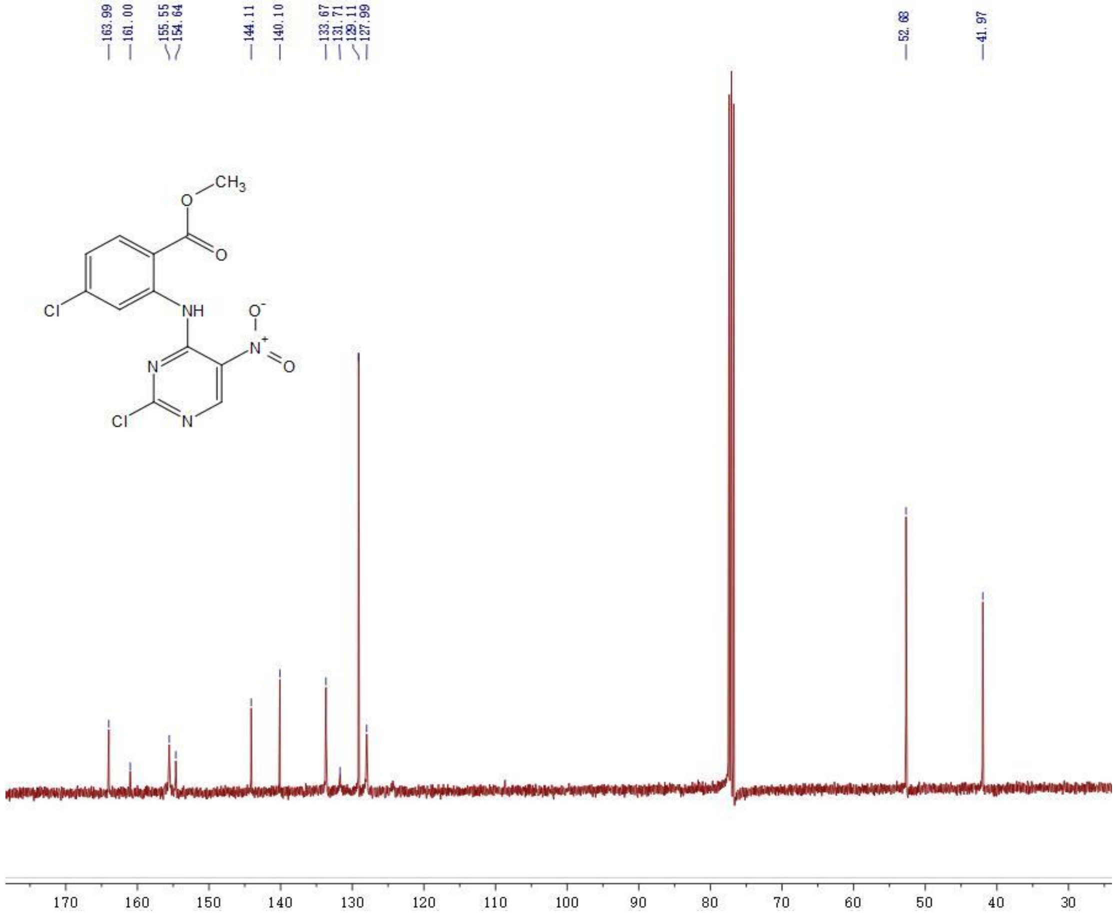

The $^{1}H/^{13}C$ NMR spectrum of intermediate **3-4**

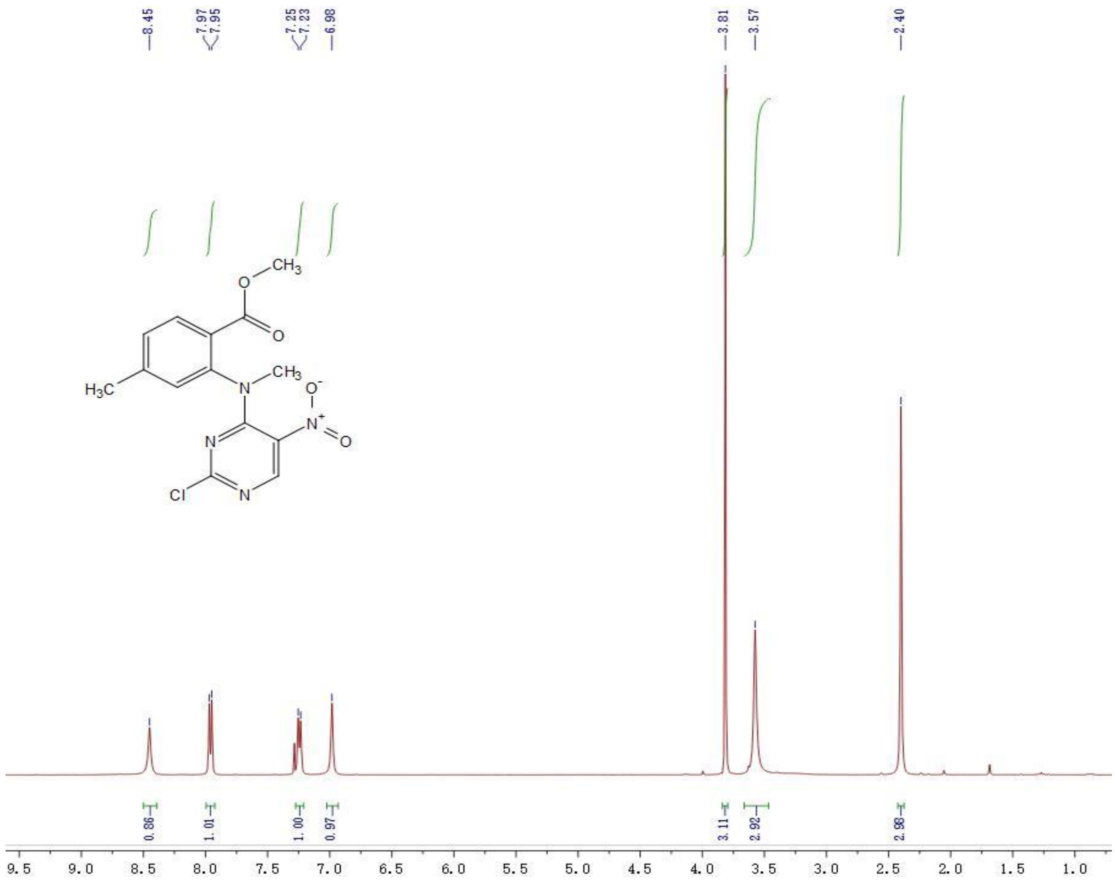

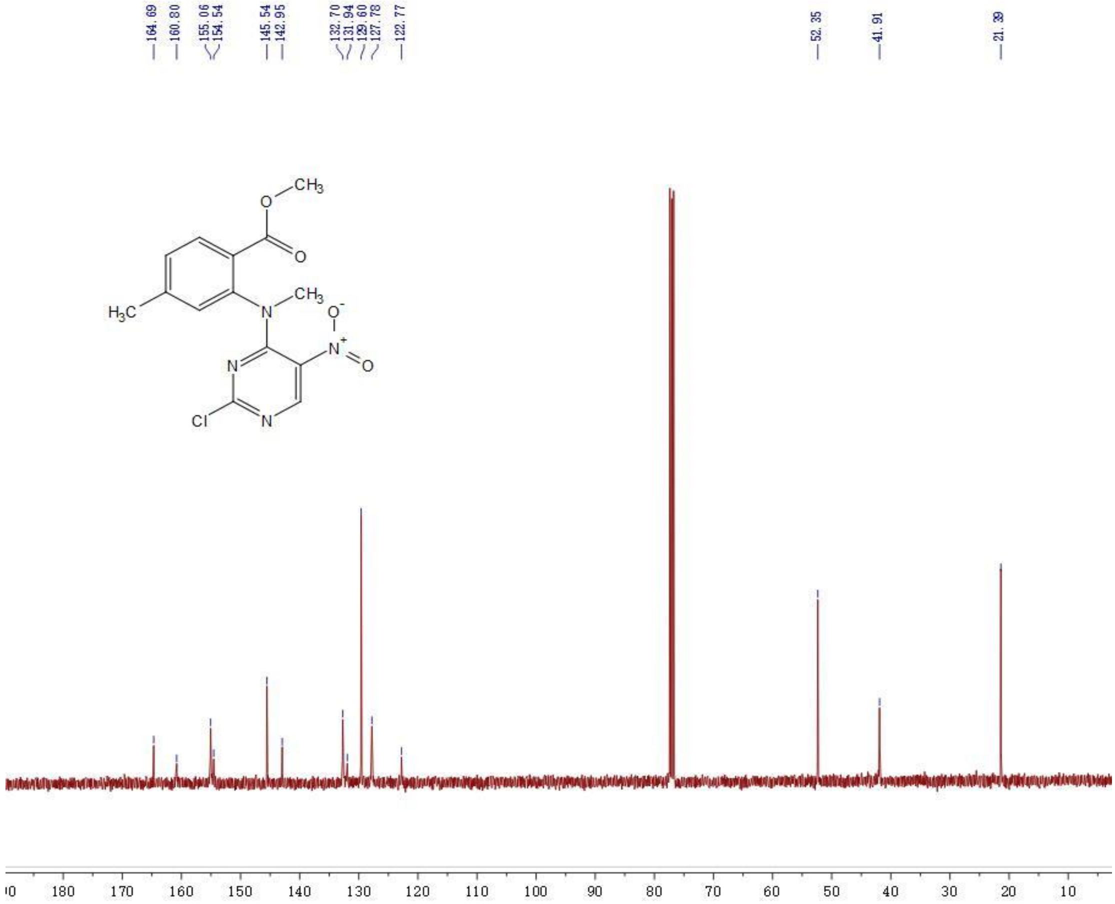

The $^{1}H/^{13}C$ NMR spectrum of intermediate **3-5**

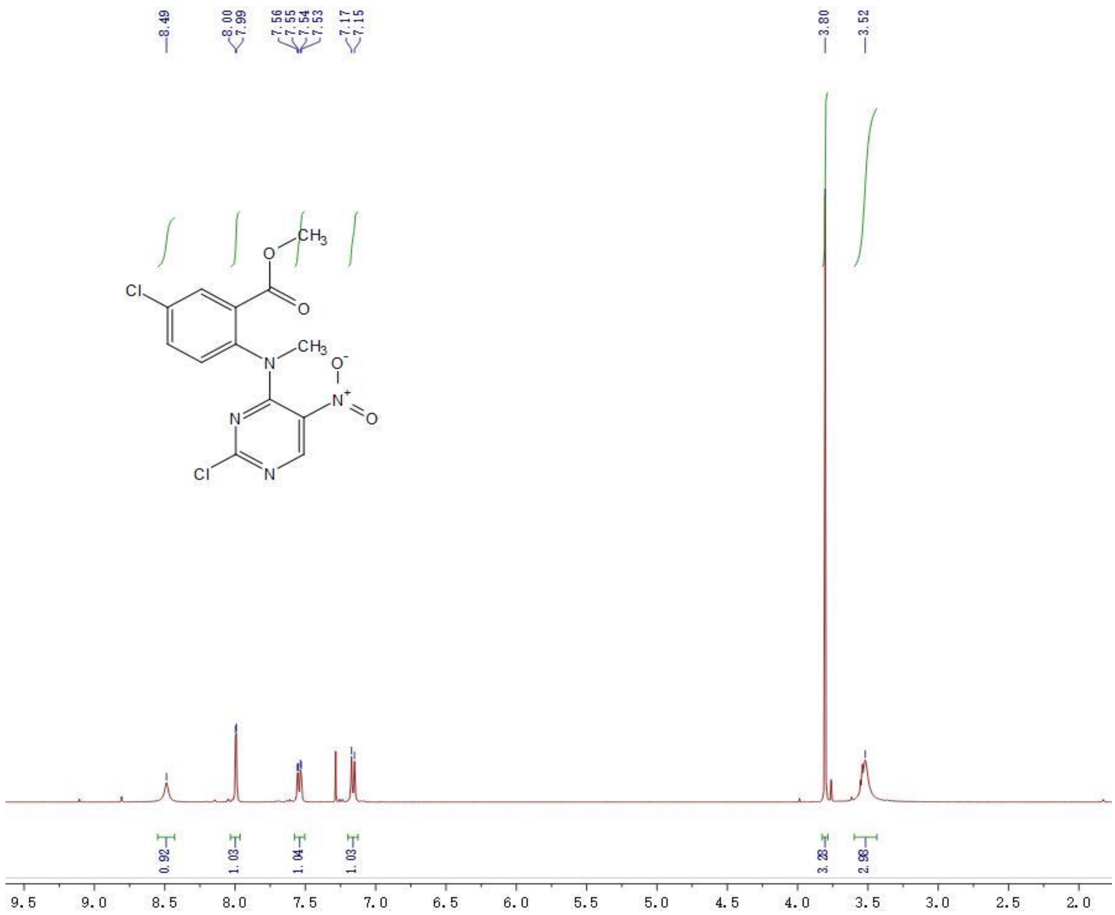

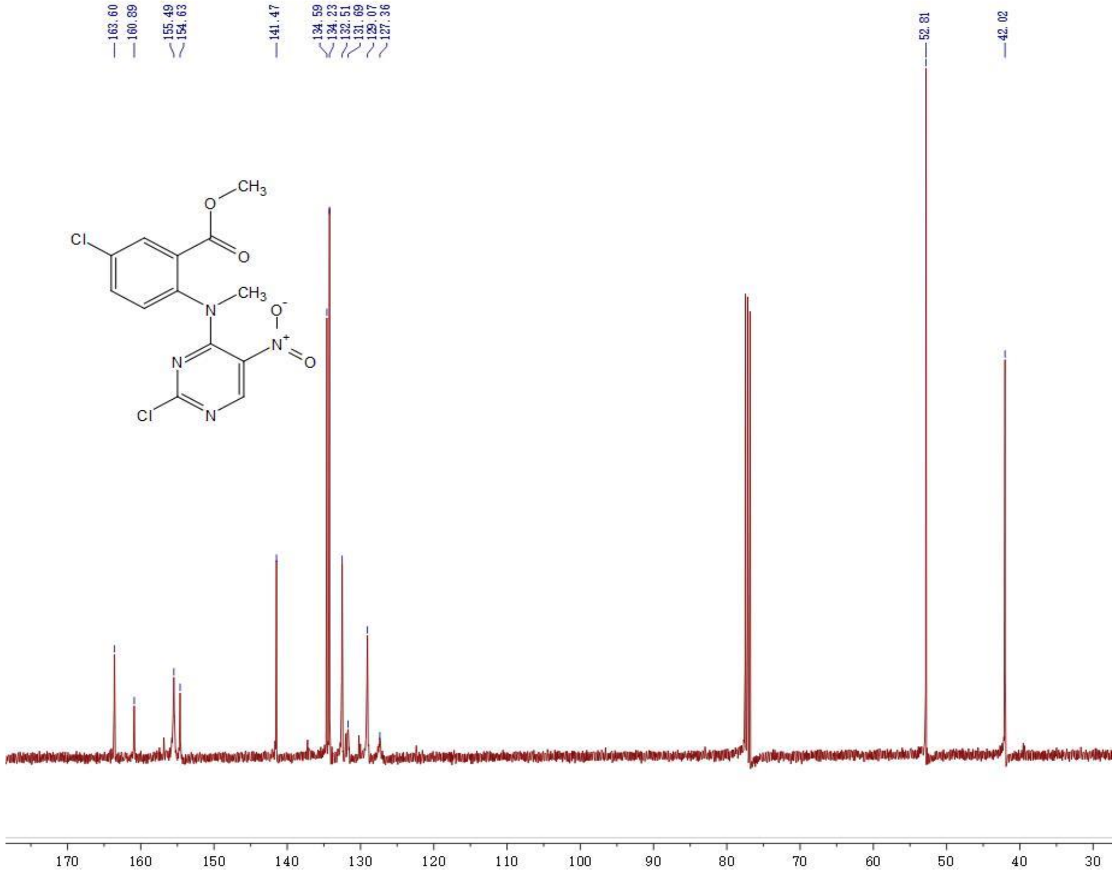

The $^{1}H/^{13}C$ NMR spectrum of intermediate **3-6**

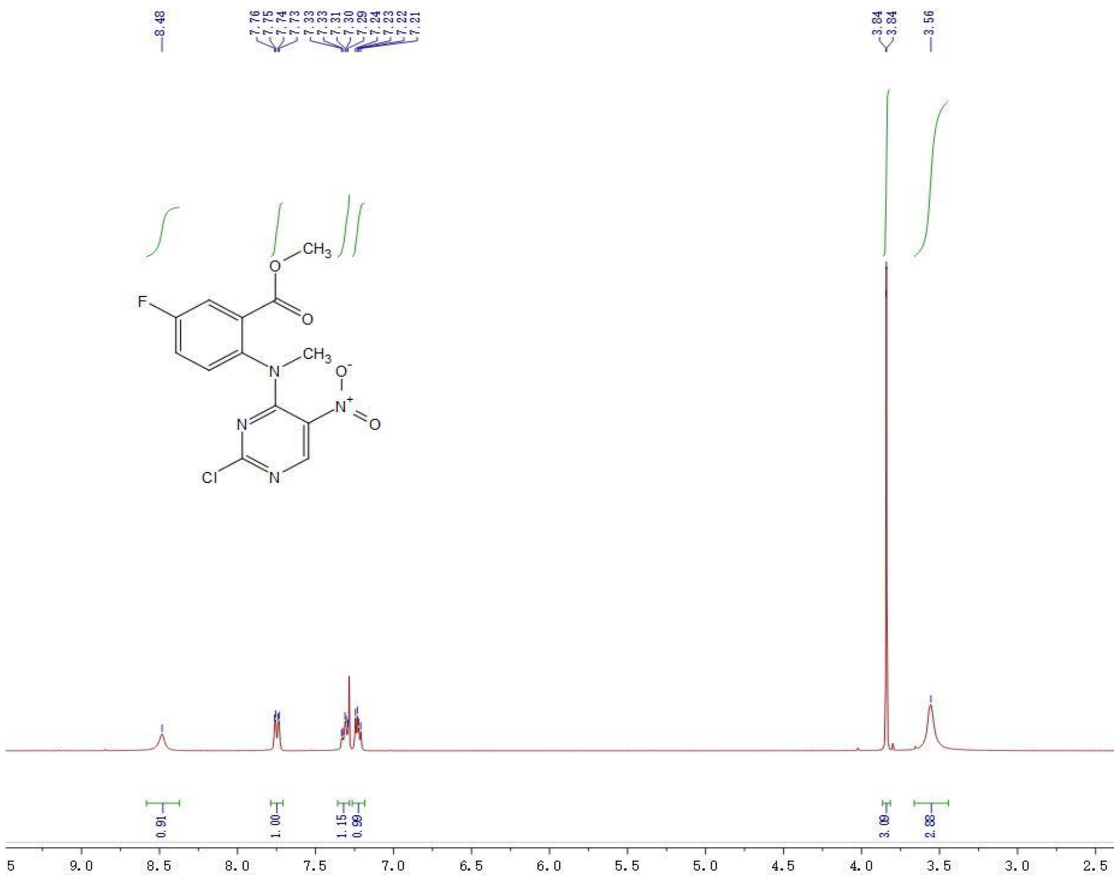

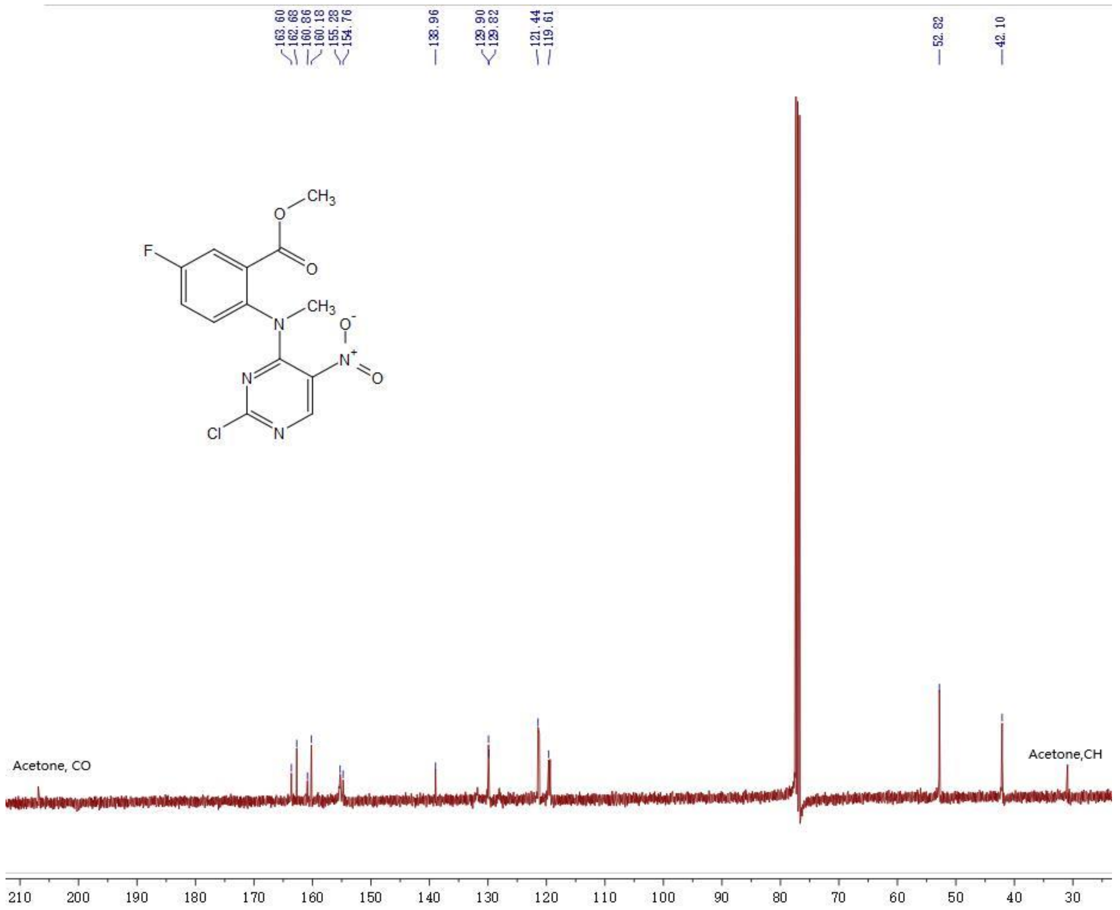

The $^{1}H/^{13}C$ NMR spectrum of intermediate **3-7**

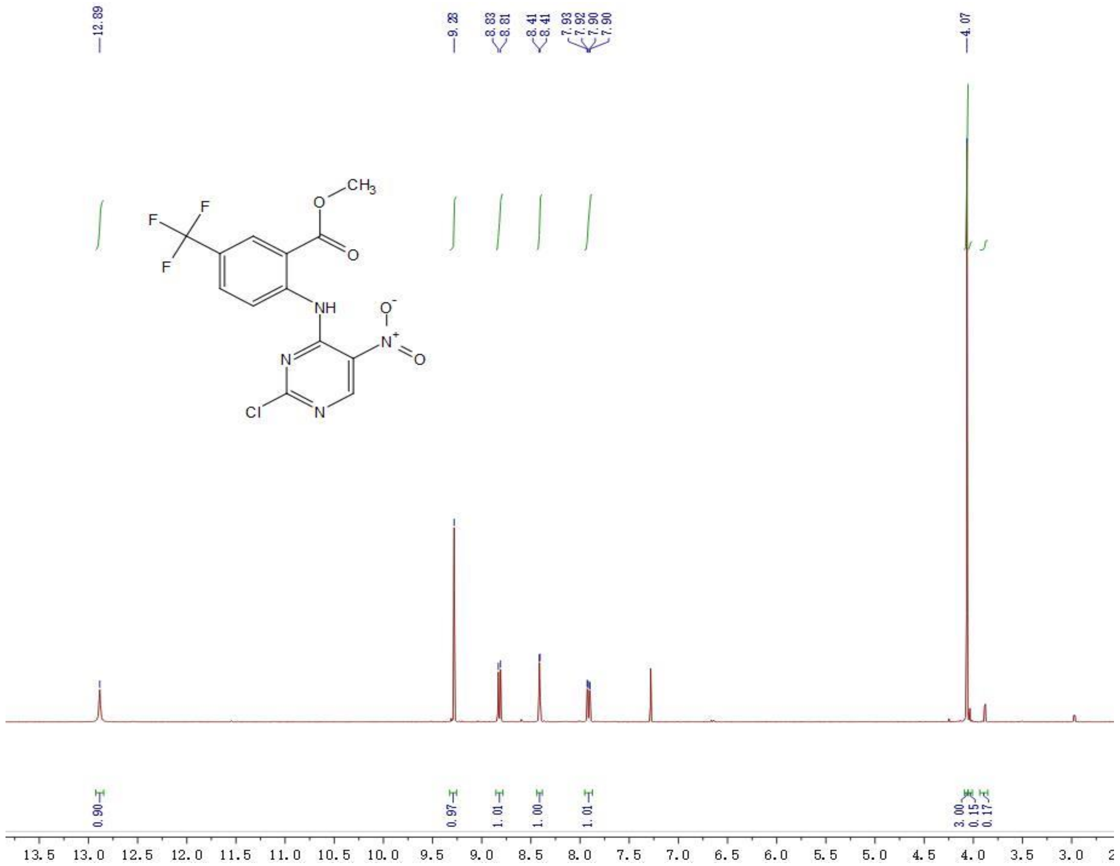

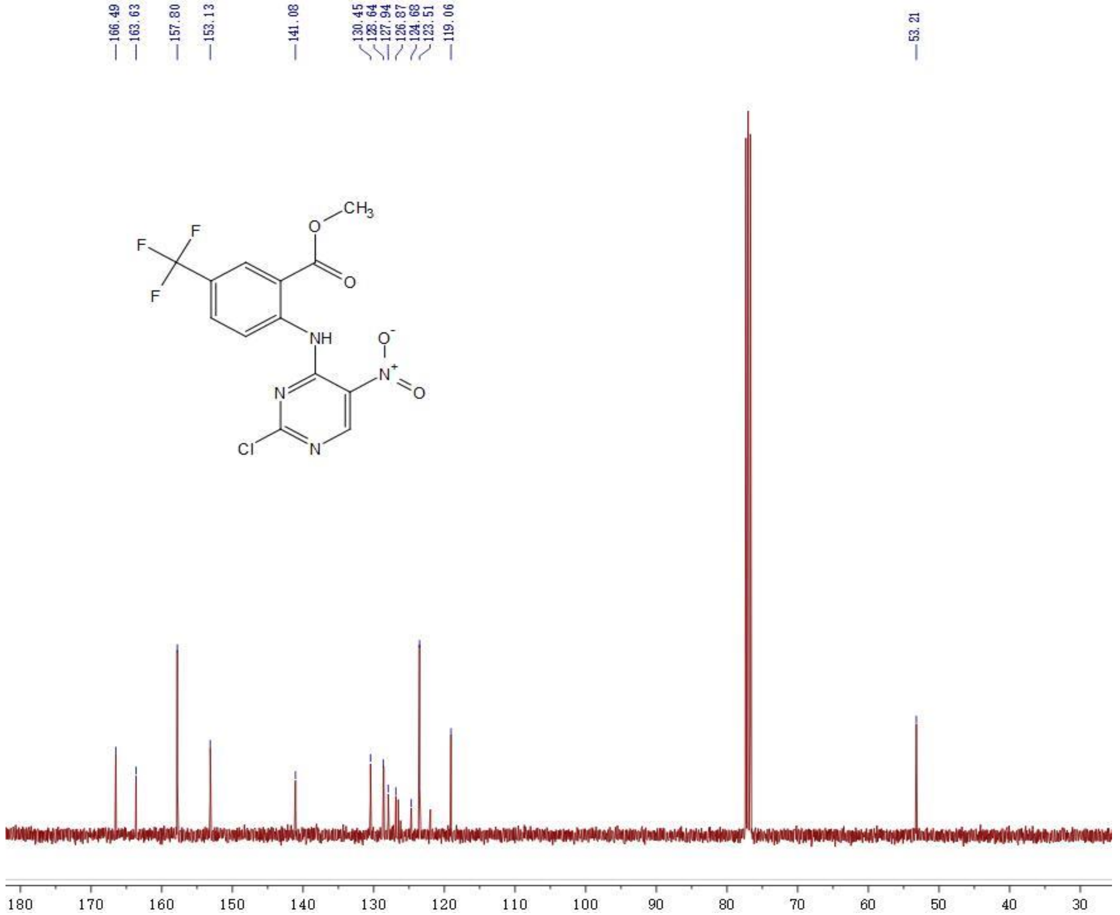

The $^{1}H/^{13}C$ NMR spectrum of intermediate **3-8**

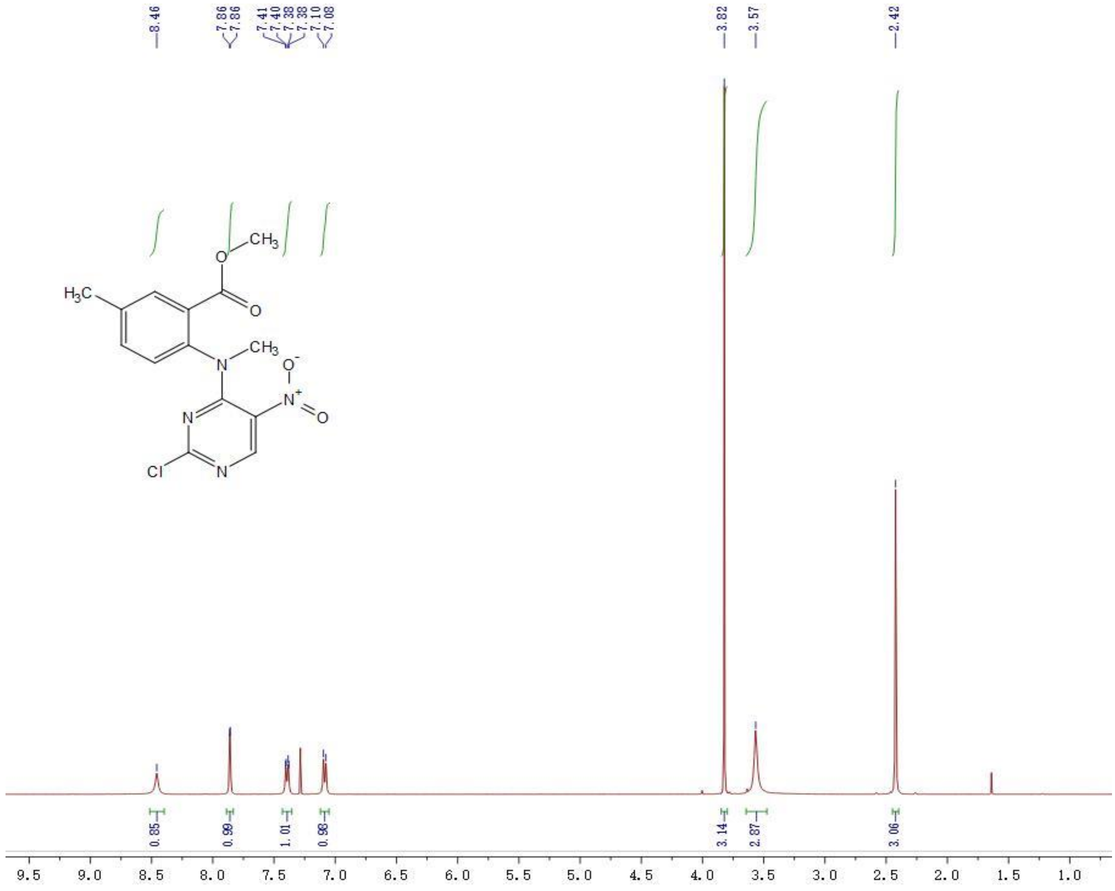

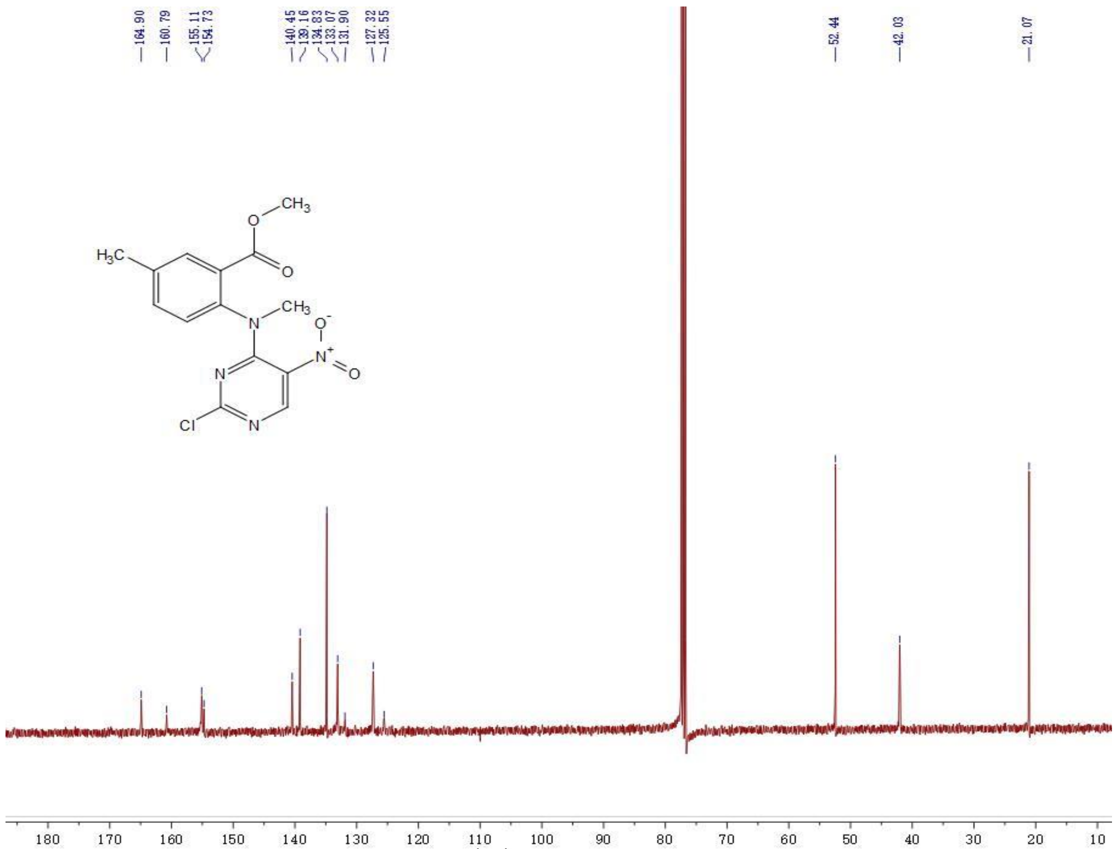

The $^{1}H/^{13}C$ NMR spectrum of intermediate **3-9**

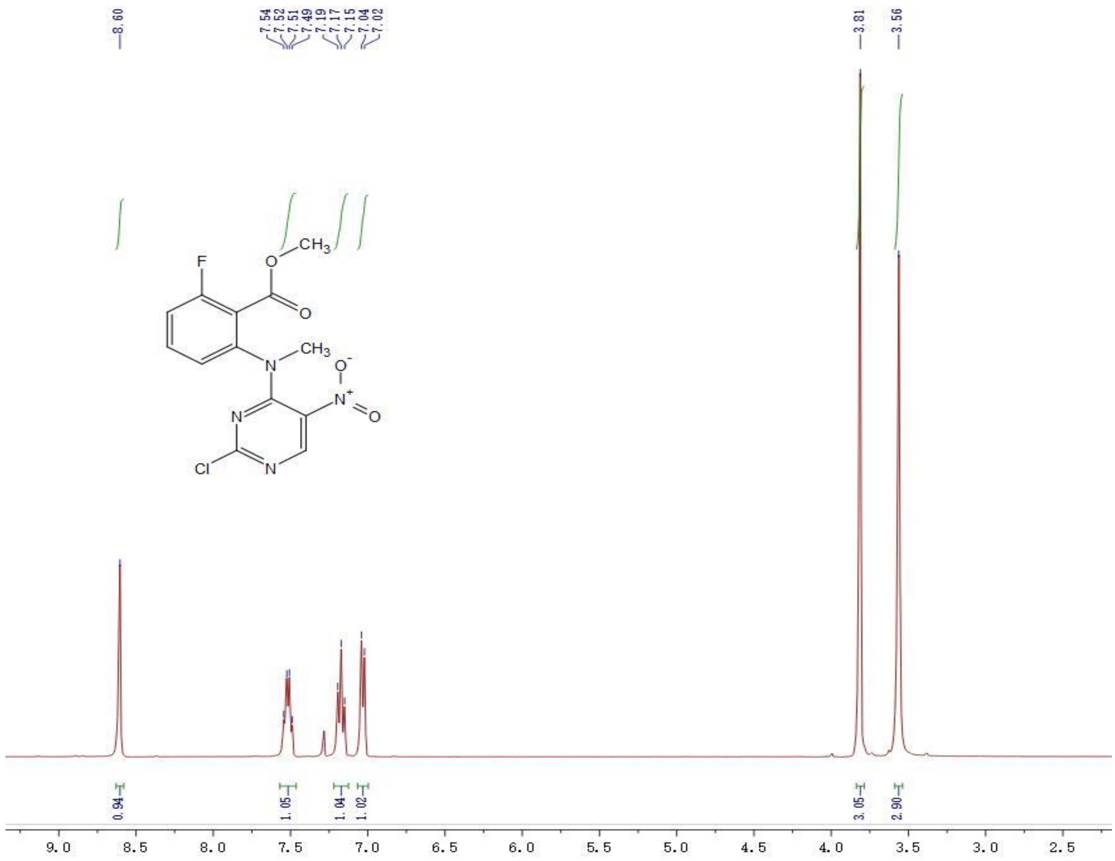

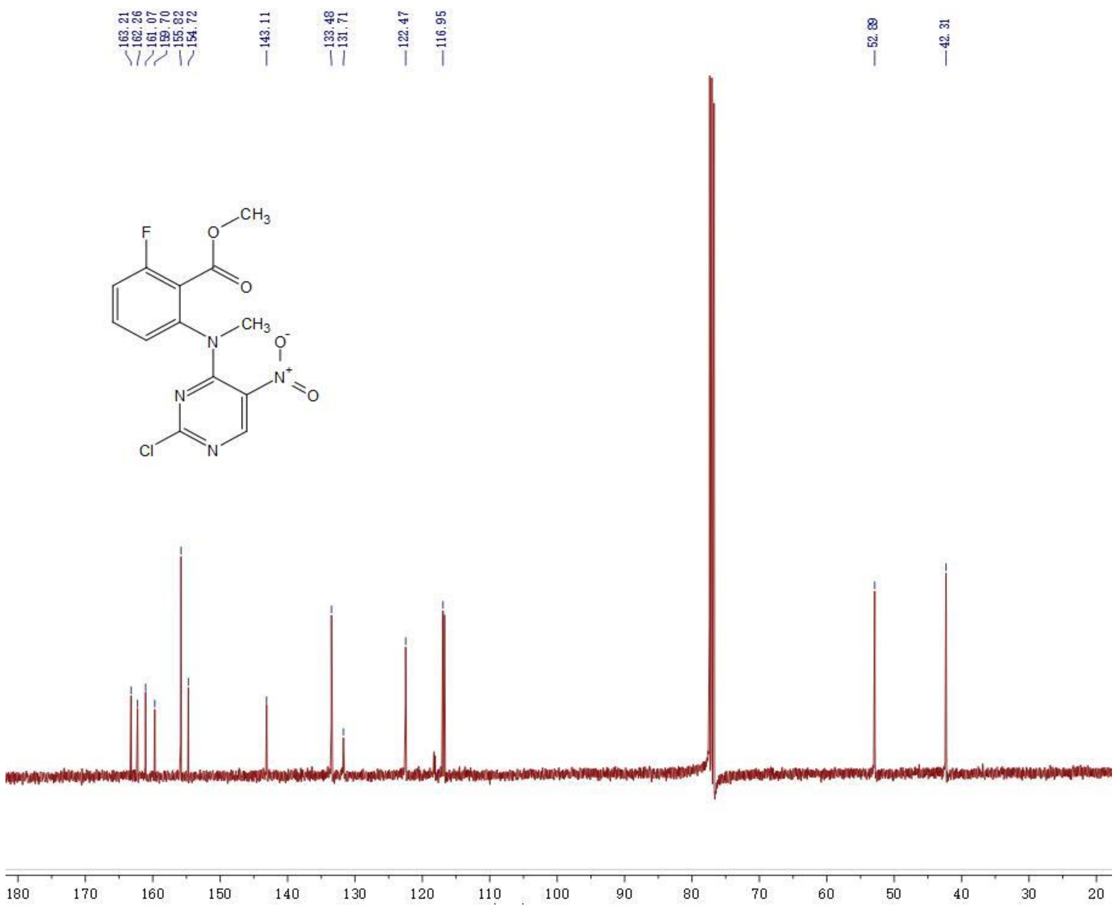

The $^{1}H/^{13}C$ NMR spectrum of intermediate **3-10**

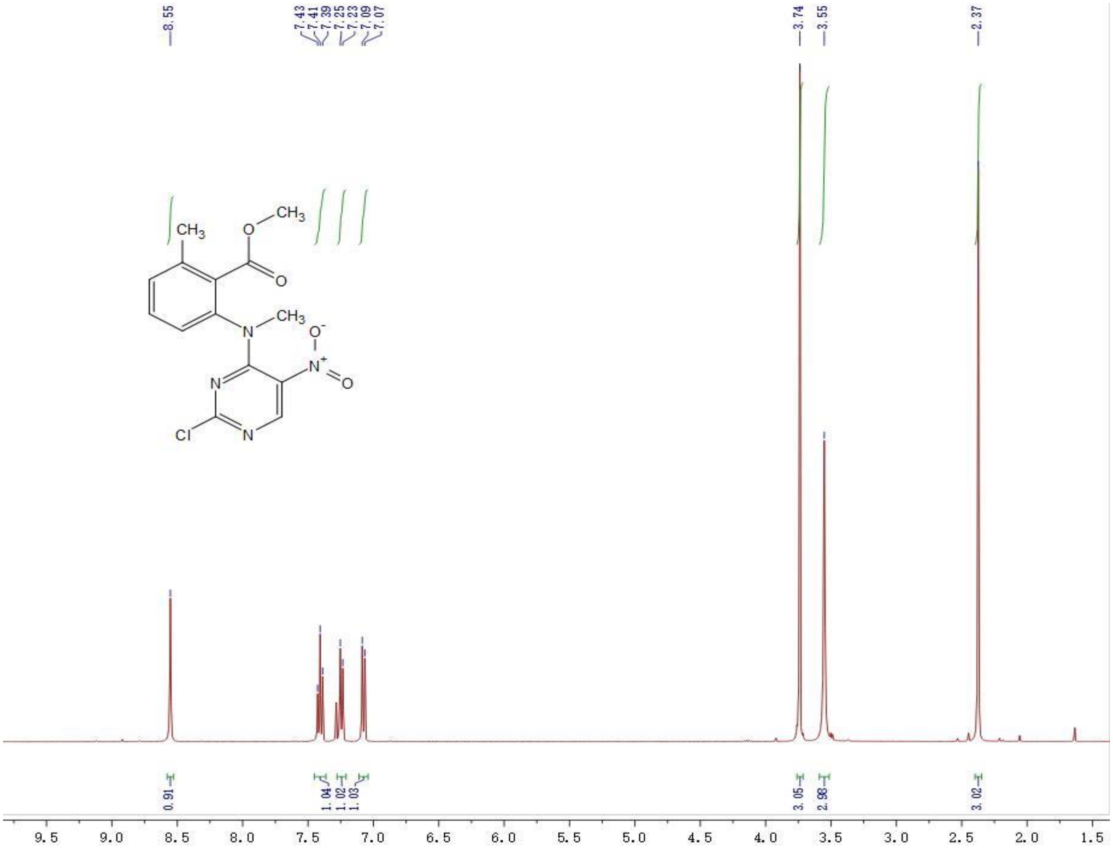

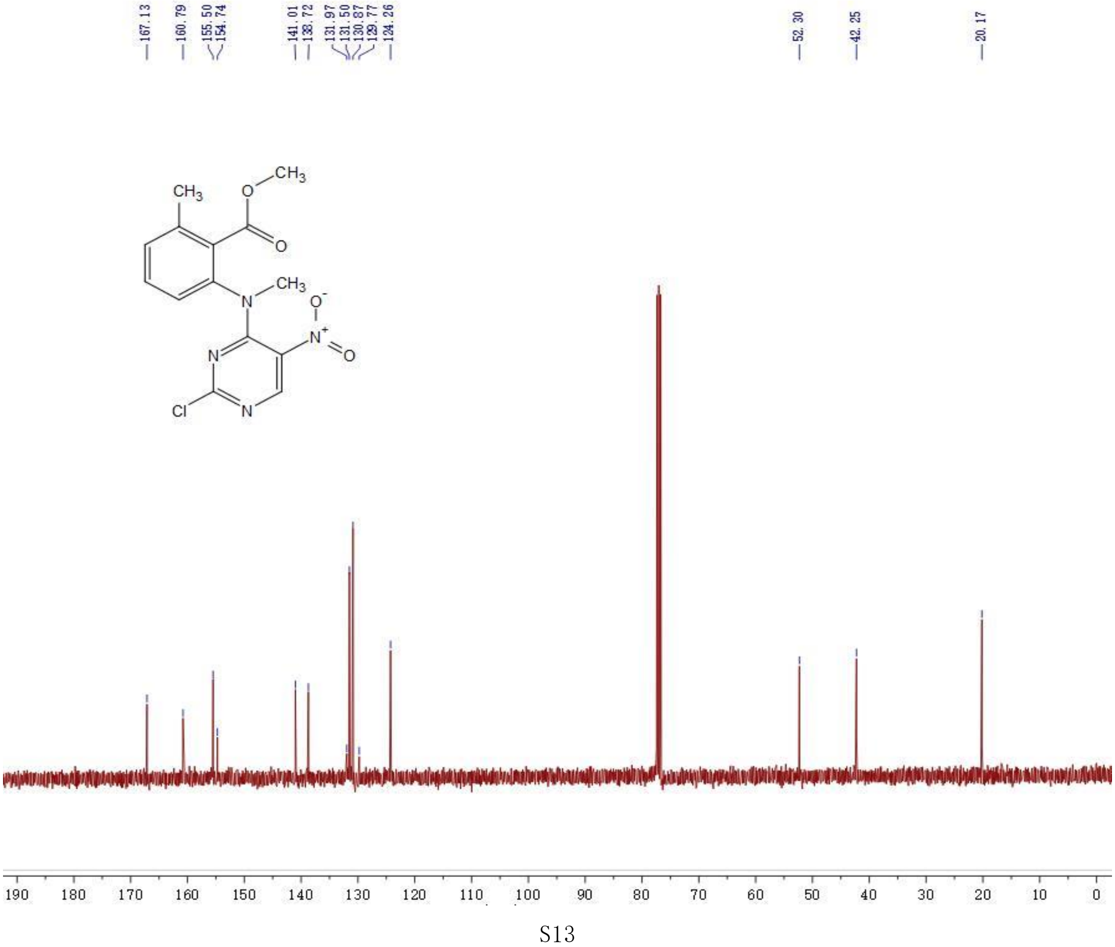

The ^{1}H/^{13}C NMR spectrum of intermediate **4-1**

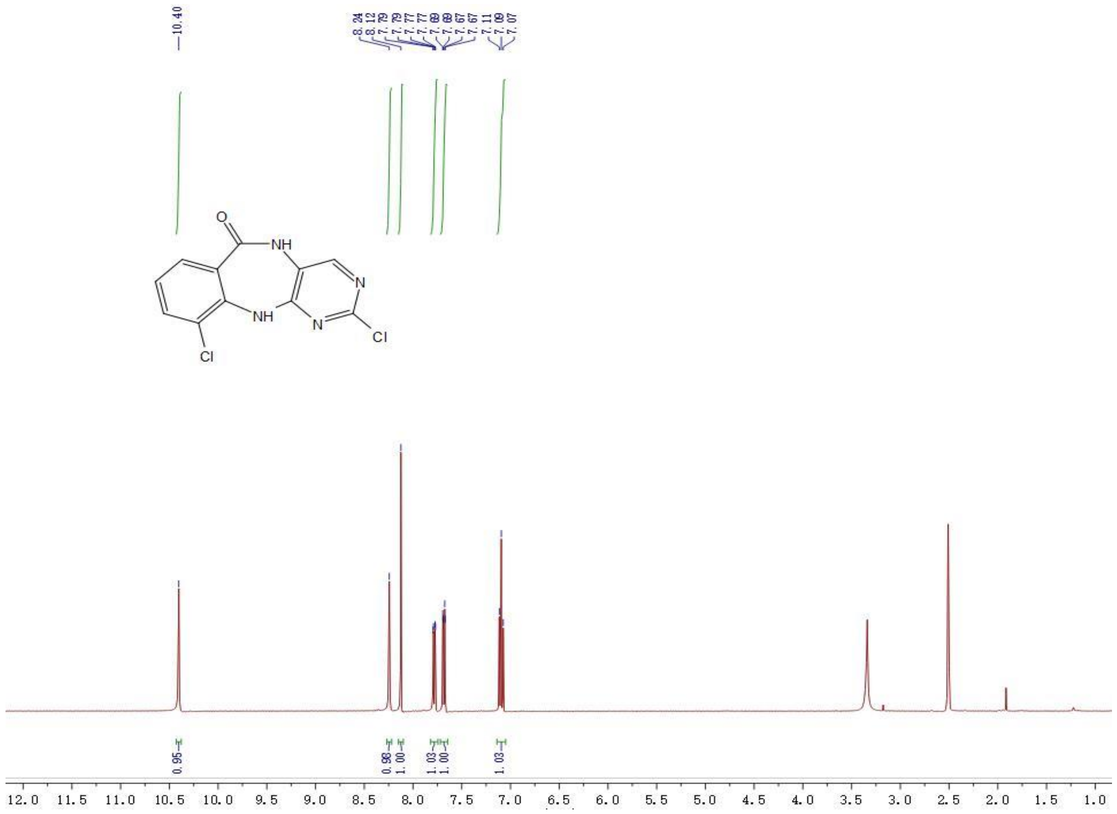

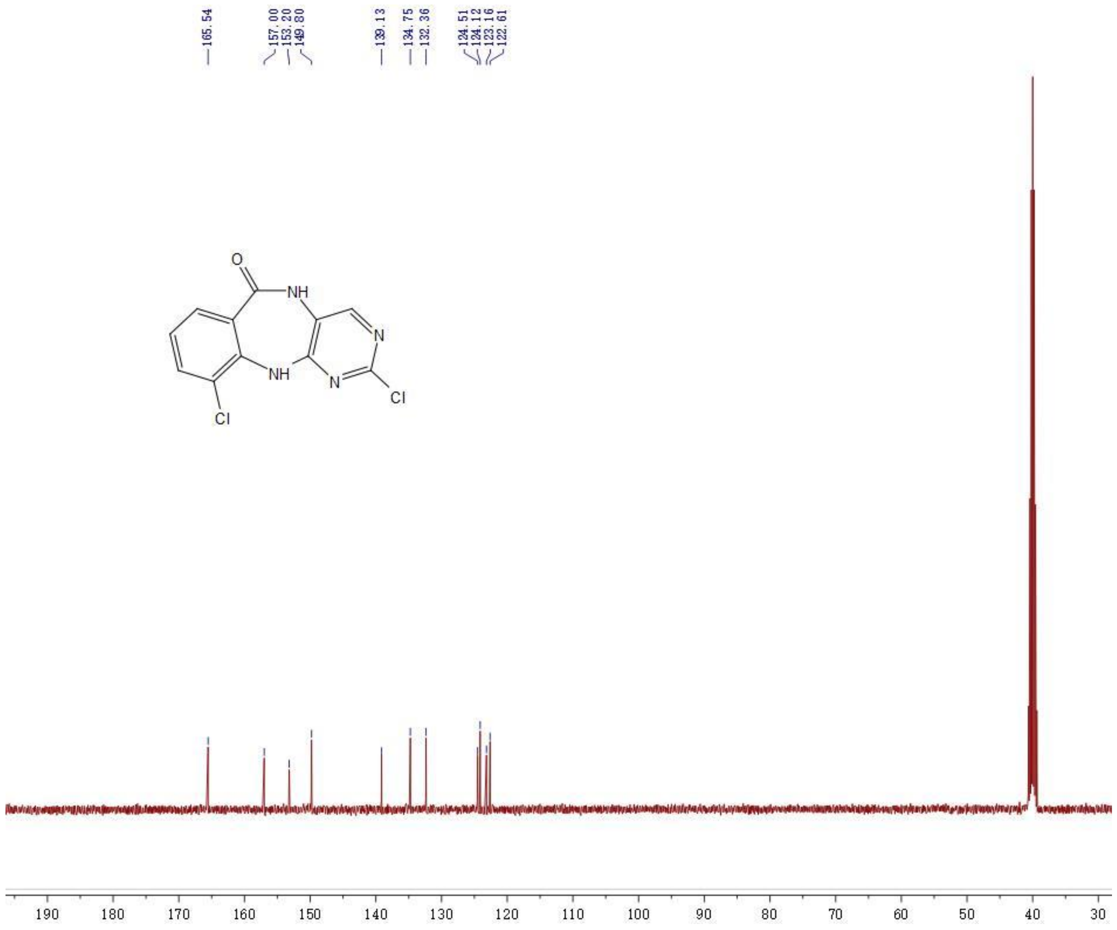

The $^{1}H/^{13}C$ NMR spectrum of intermediate **4-2**

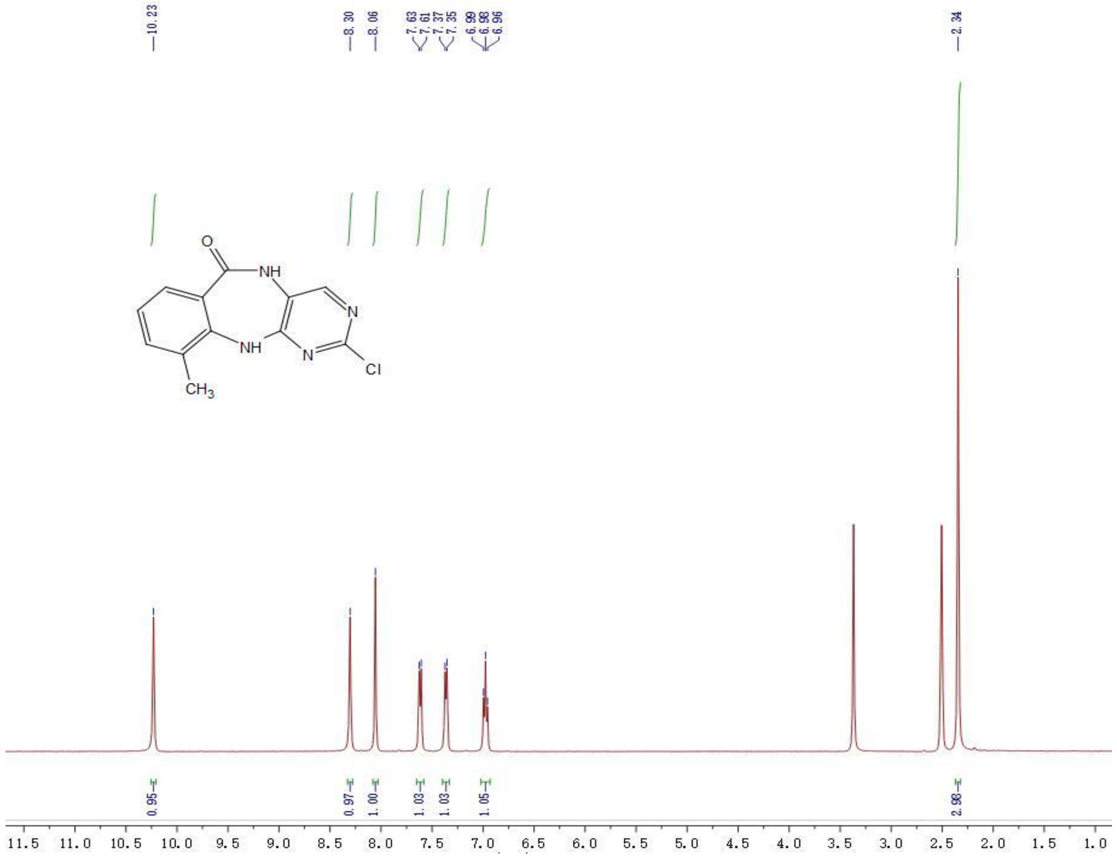

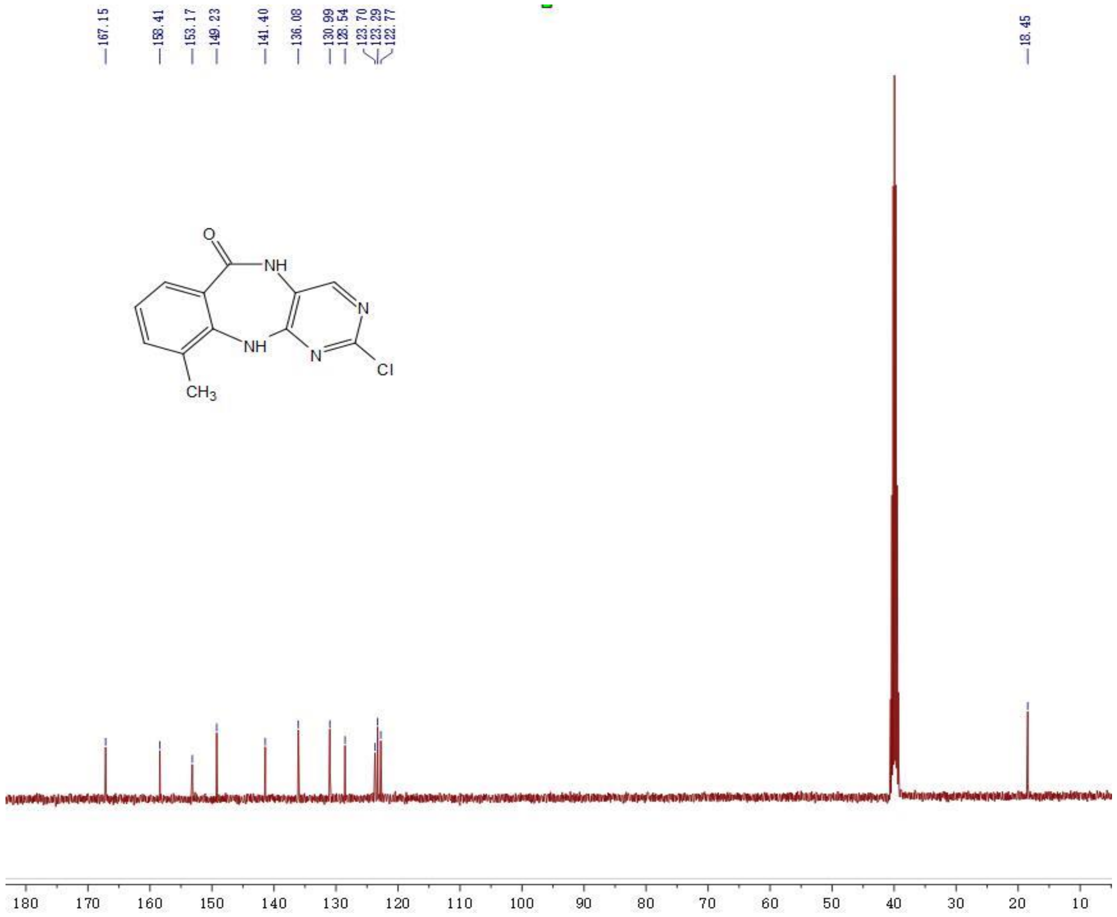

The $^{1}H/^{13}C$ NMR spectrum of intermediate **4-3**

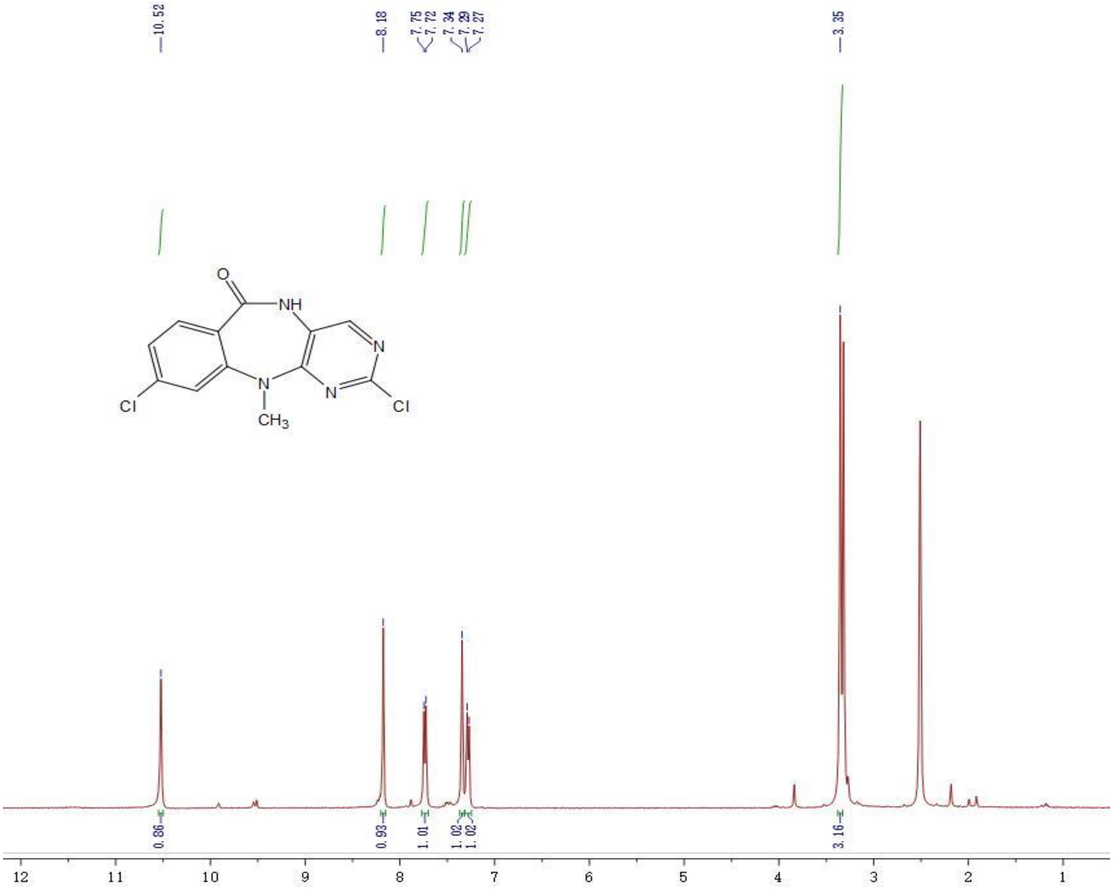

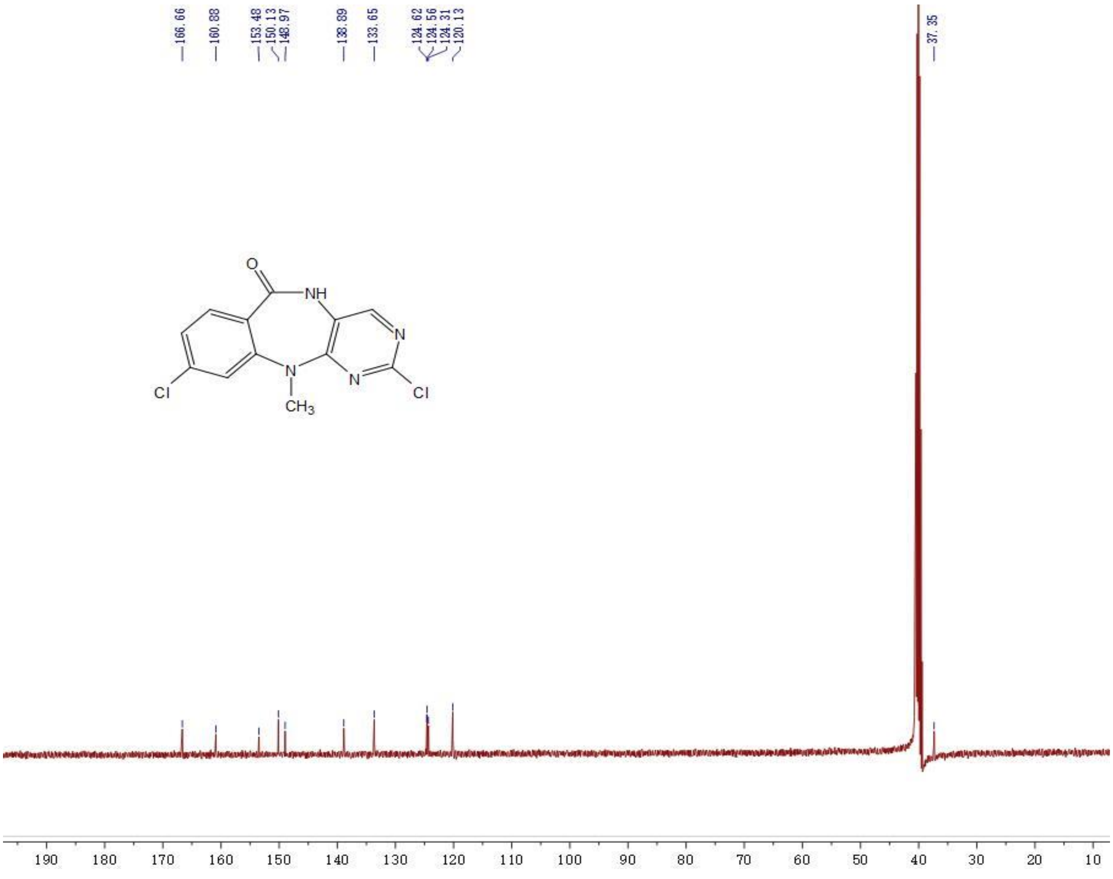

The $^{1}H/^{13}C$ NMR spectrum of intermediate **4-4**

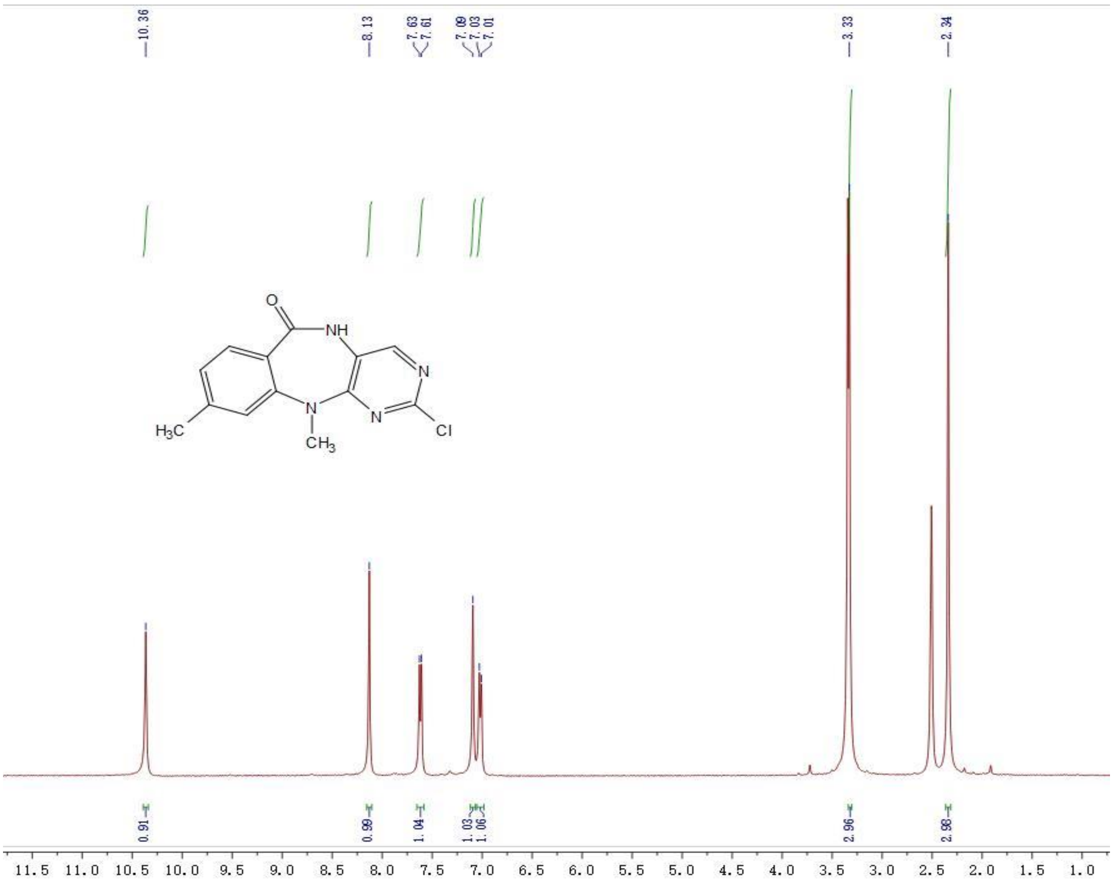

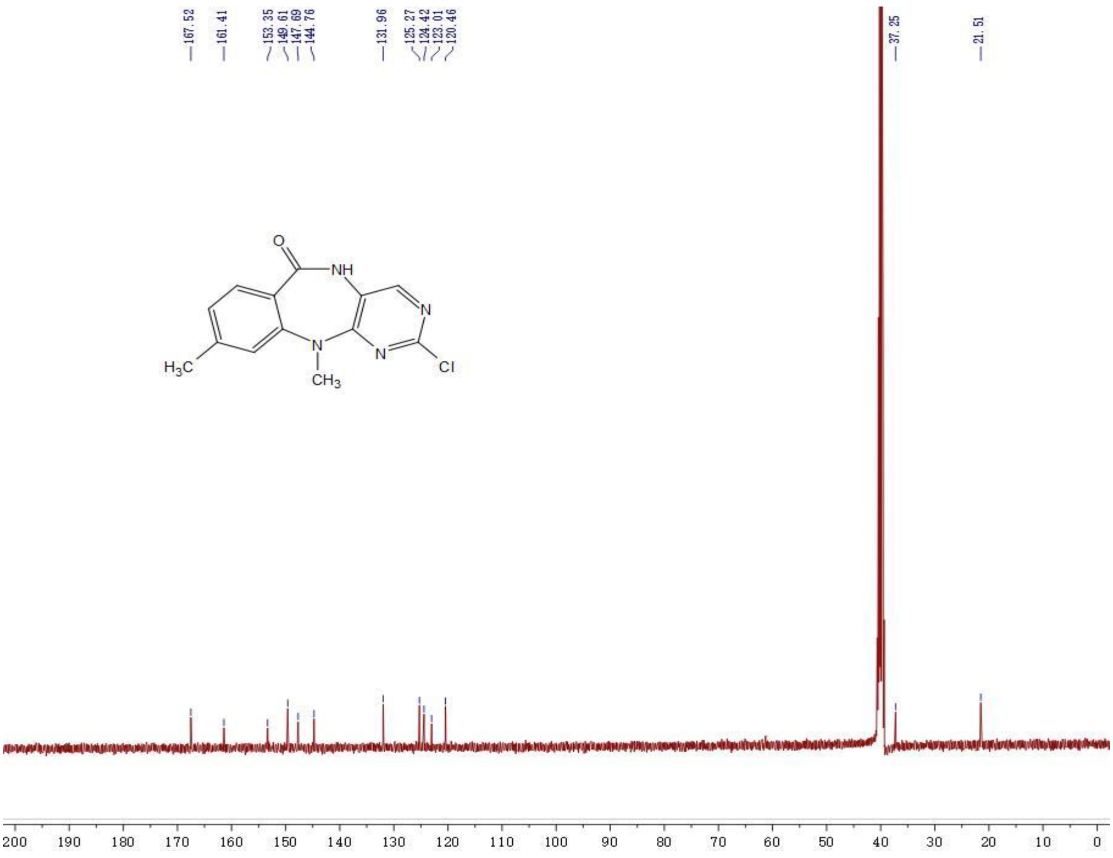

The $^{1}H/^{13}C$ NMR spectrum of intermediate **4-5**

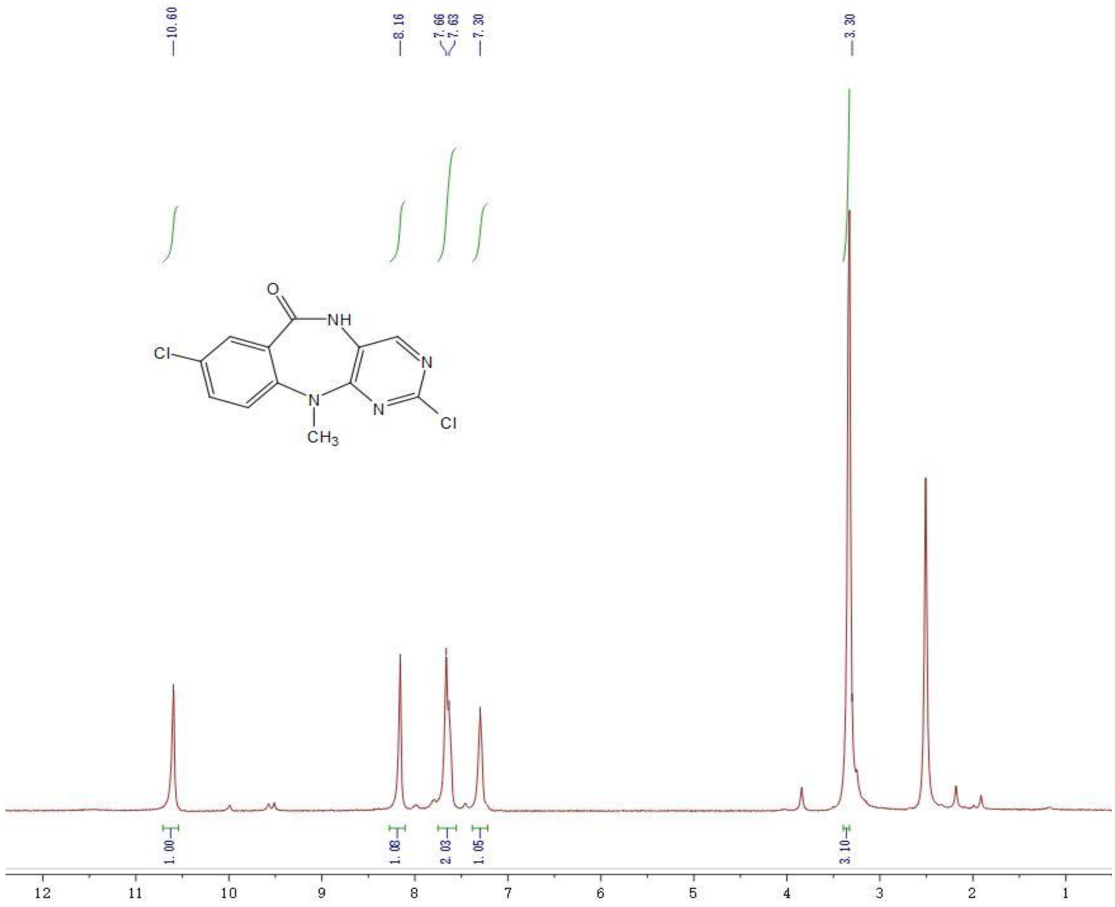

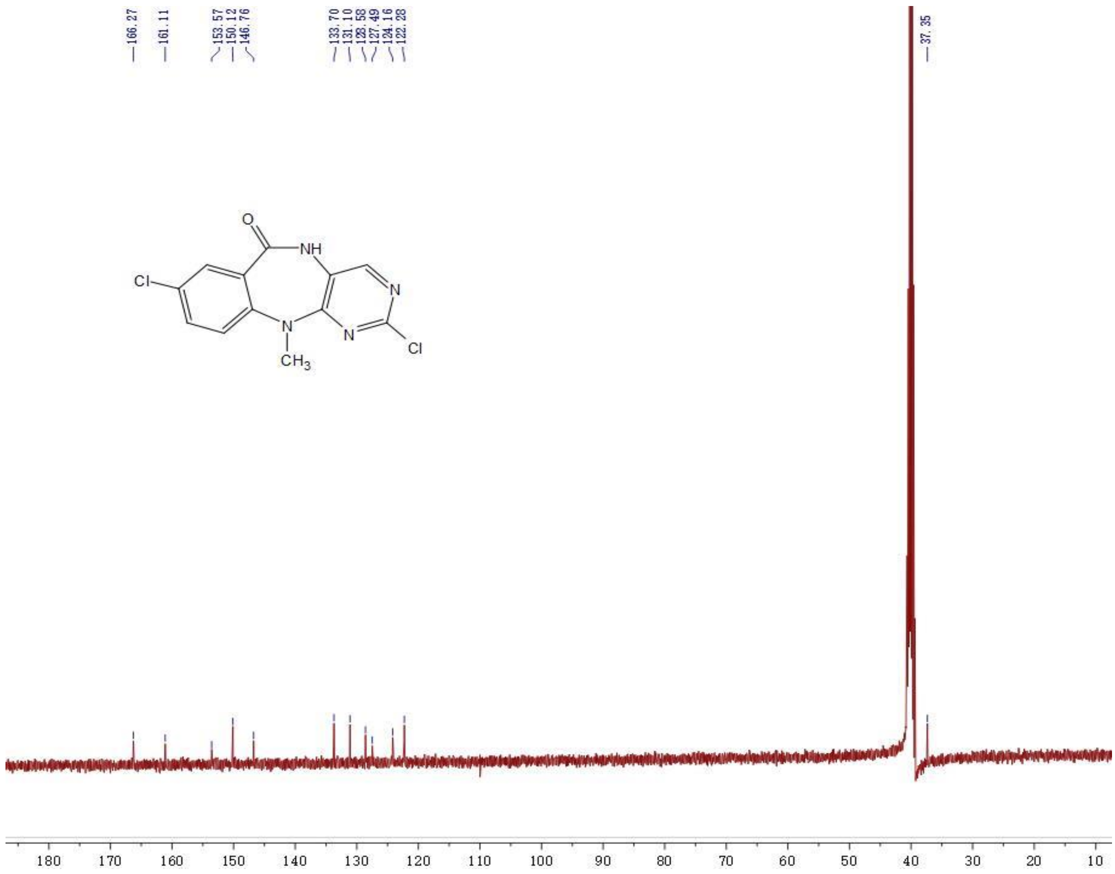

The $^1H/^{13}C$ NMR spectrum of intermediate **4-6**

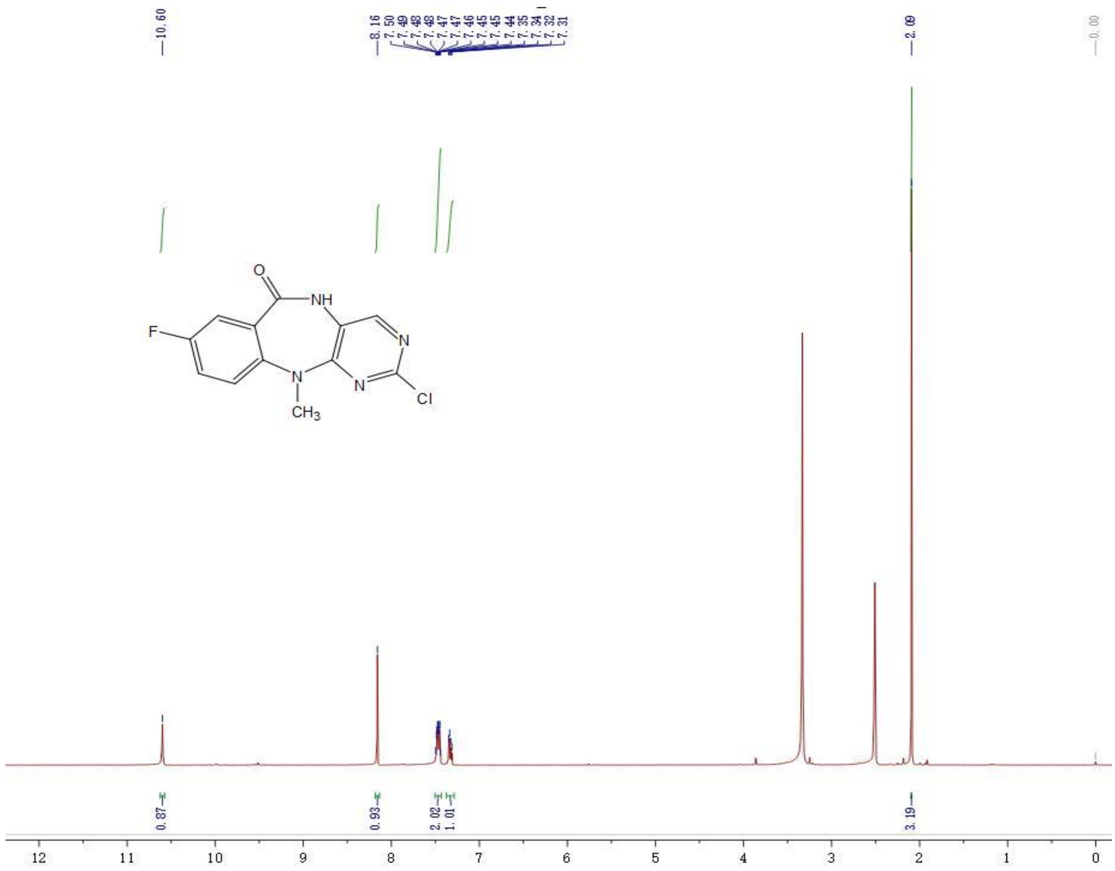

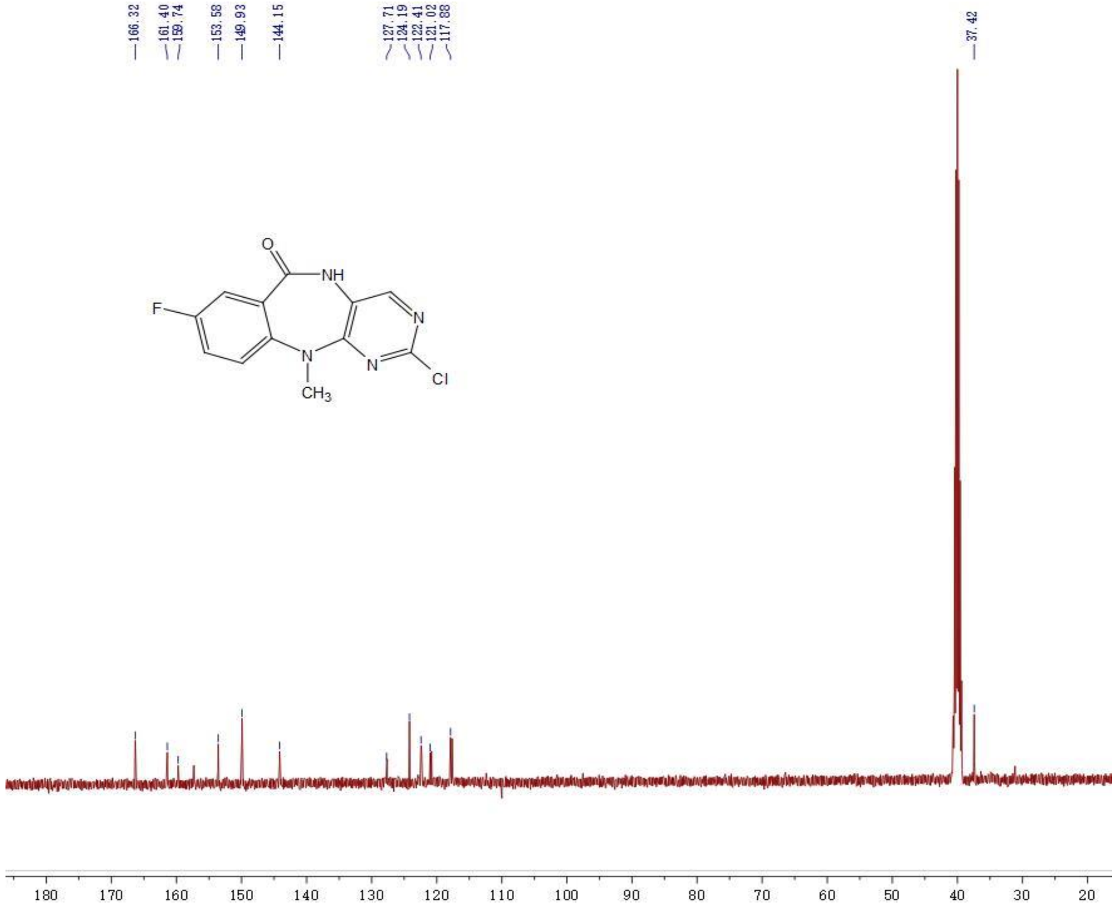

The $^{1}H/^{13}C$ NMR spectrum of intermediate **4-7**

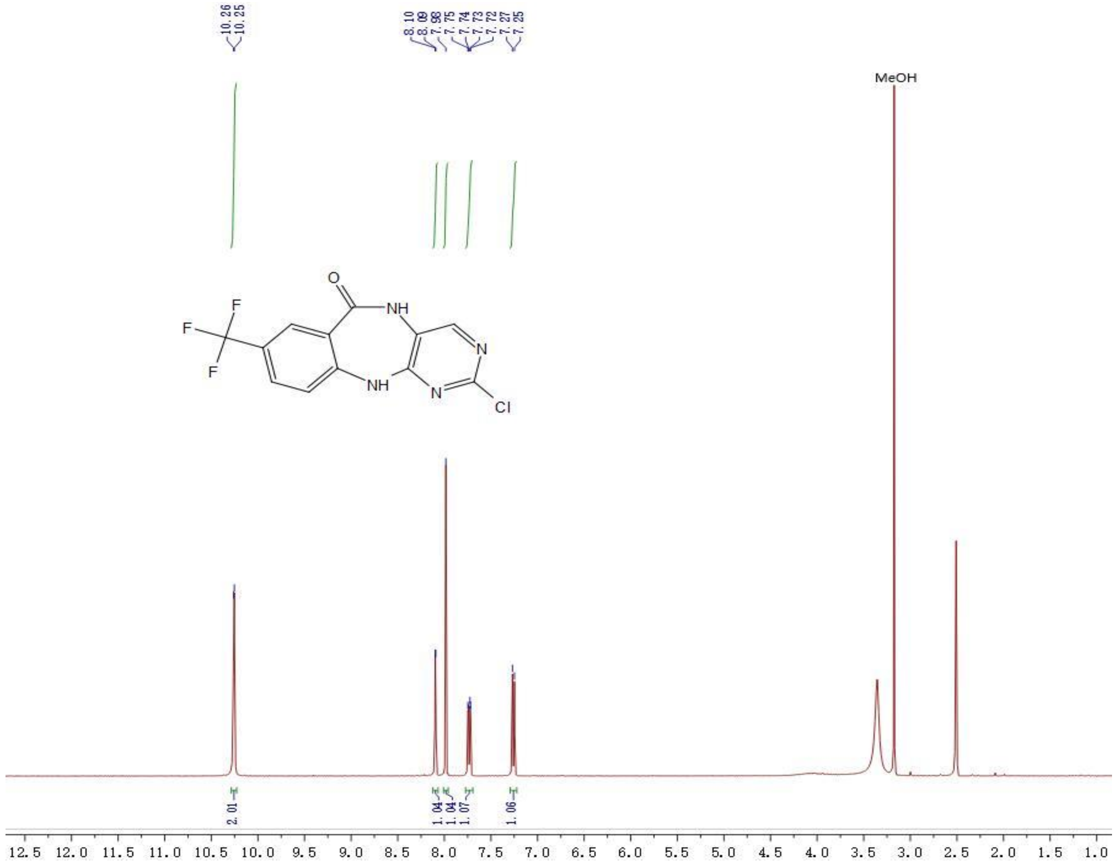

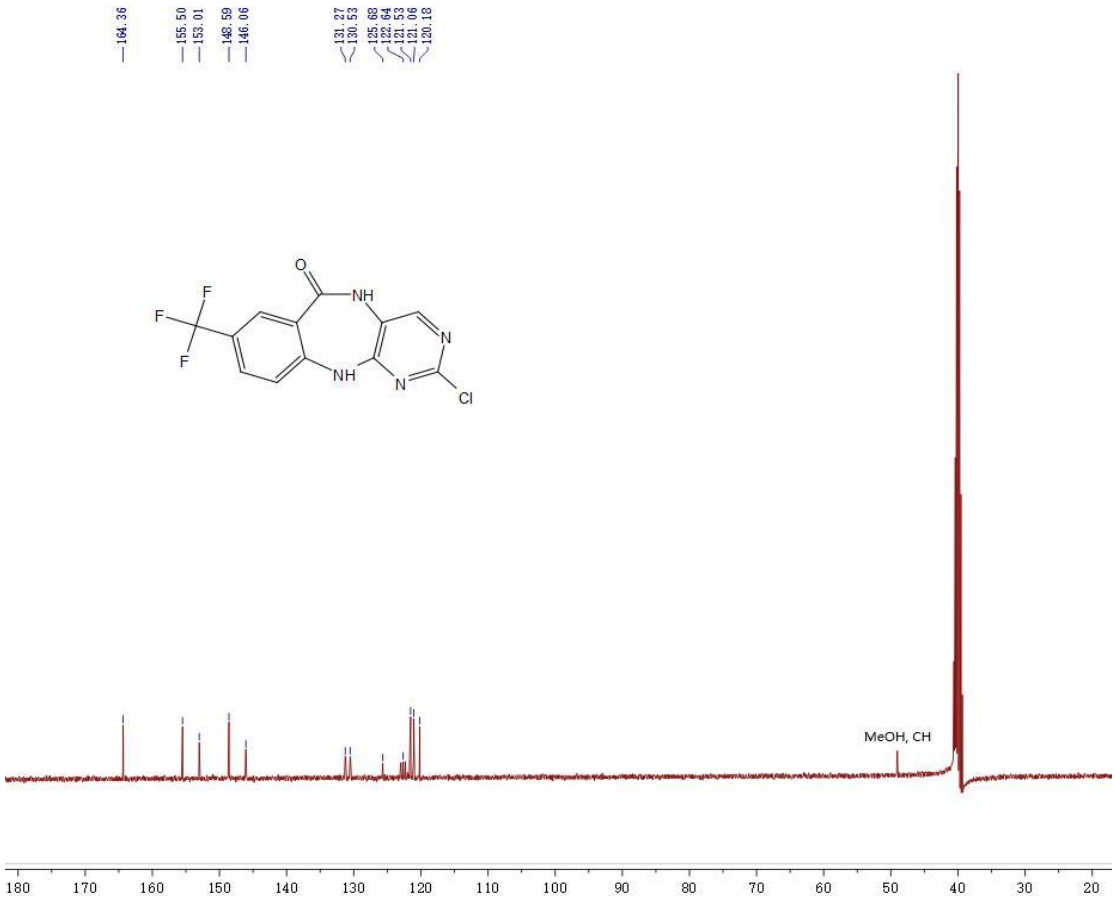

The $^{1}H/^{13}C$ NMR spectrum of intermediate **4-8**

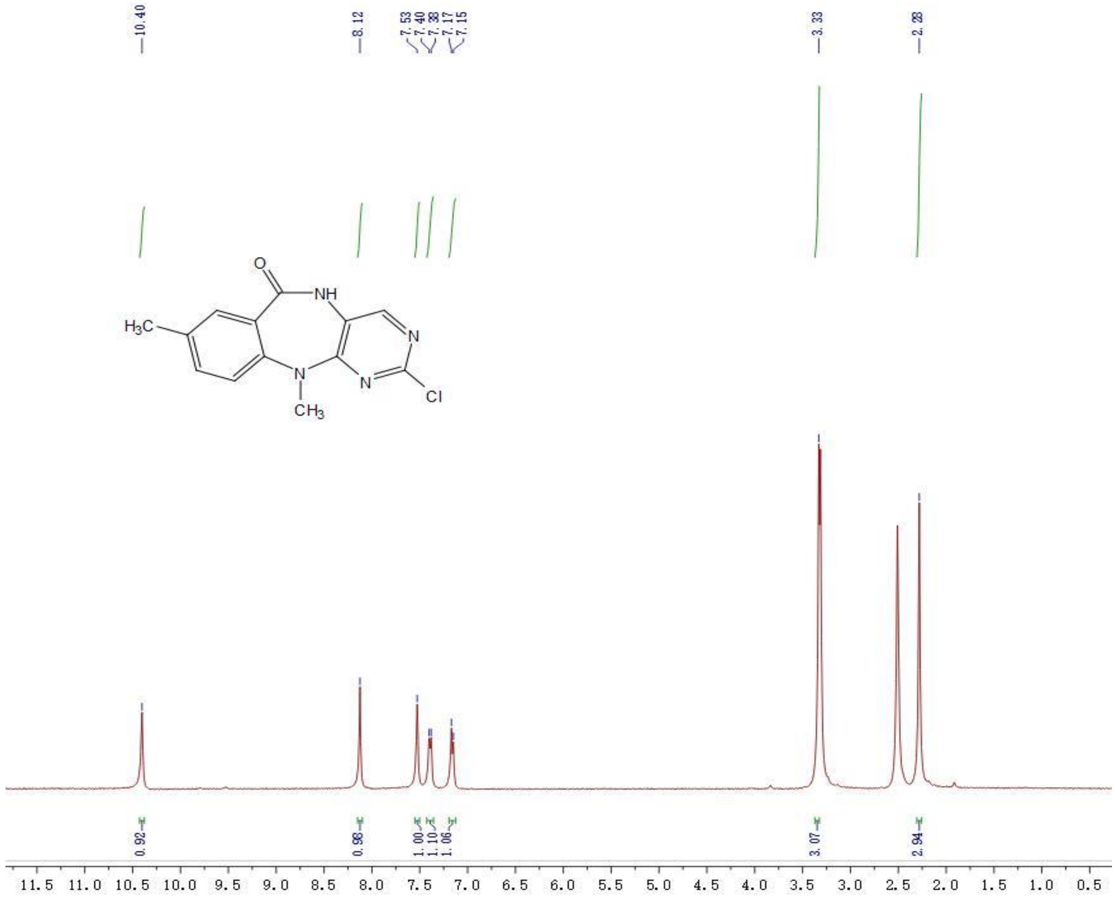

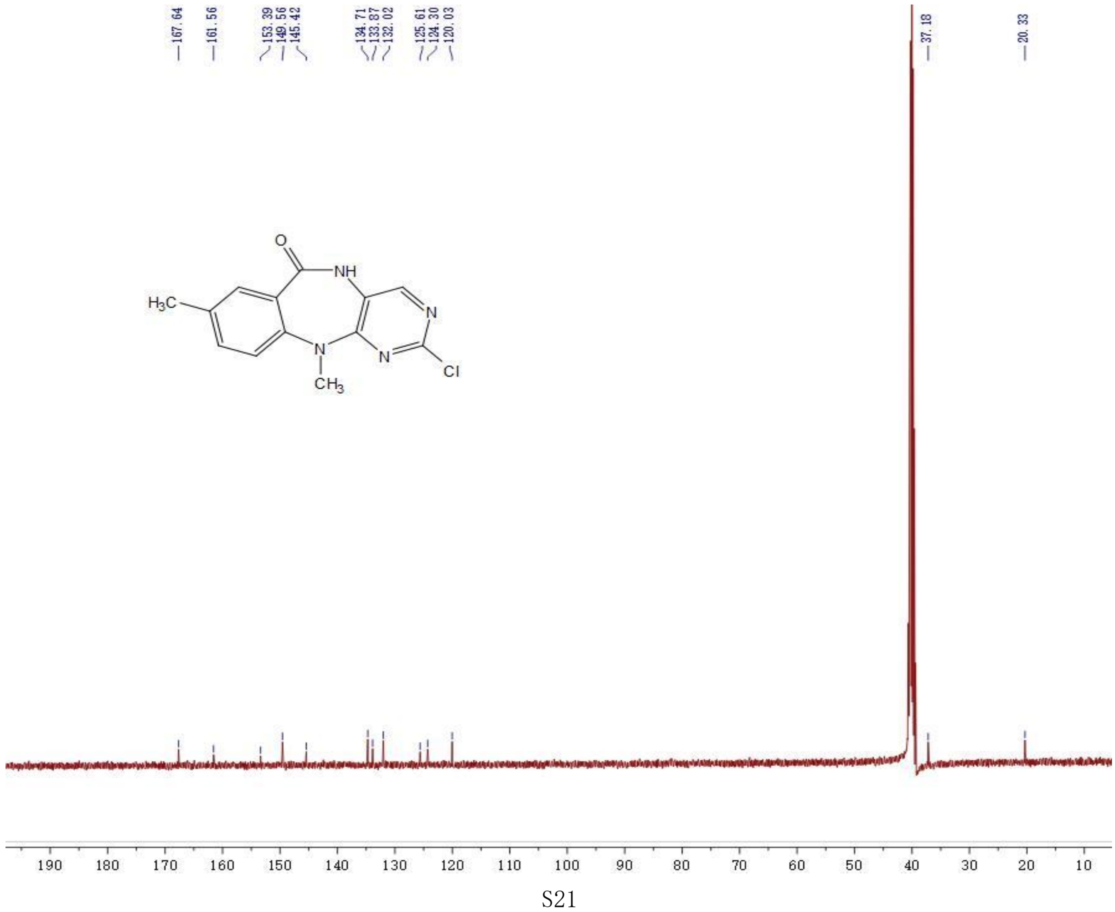

The $^{1}H/^{13}C$ NMR spectrum of intermediate **4-9**

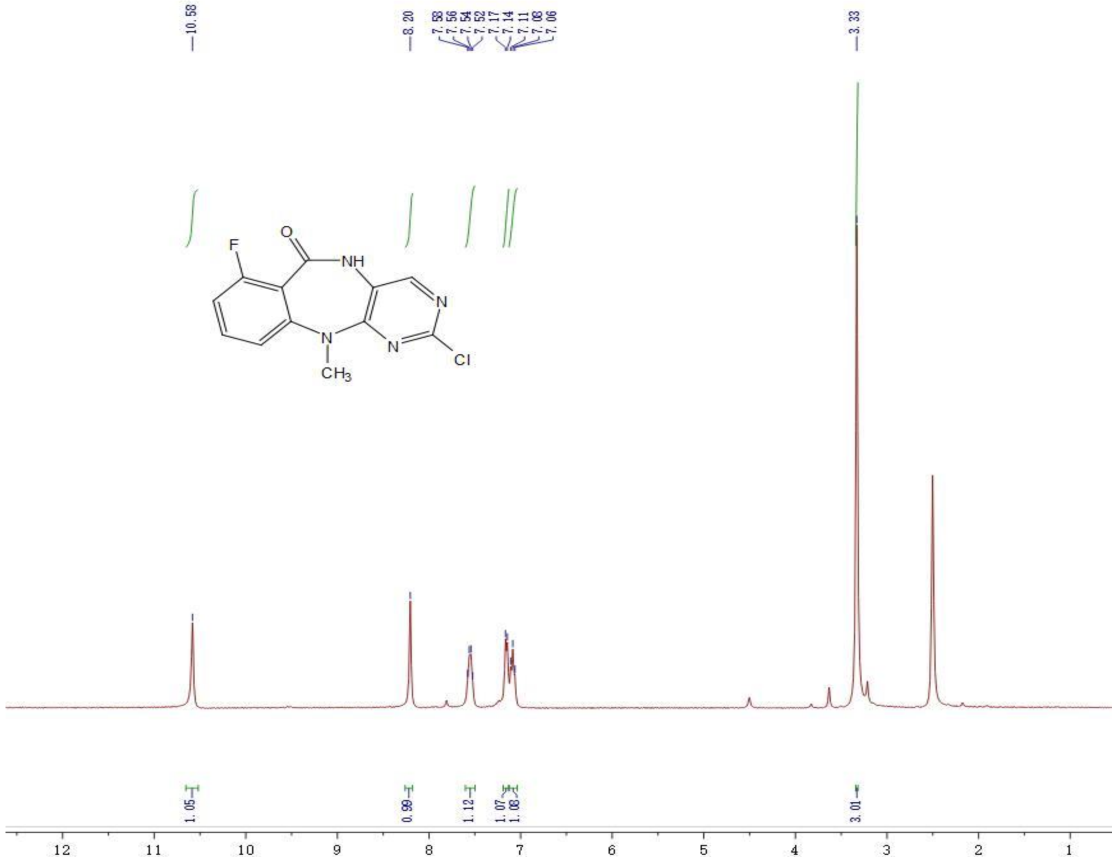

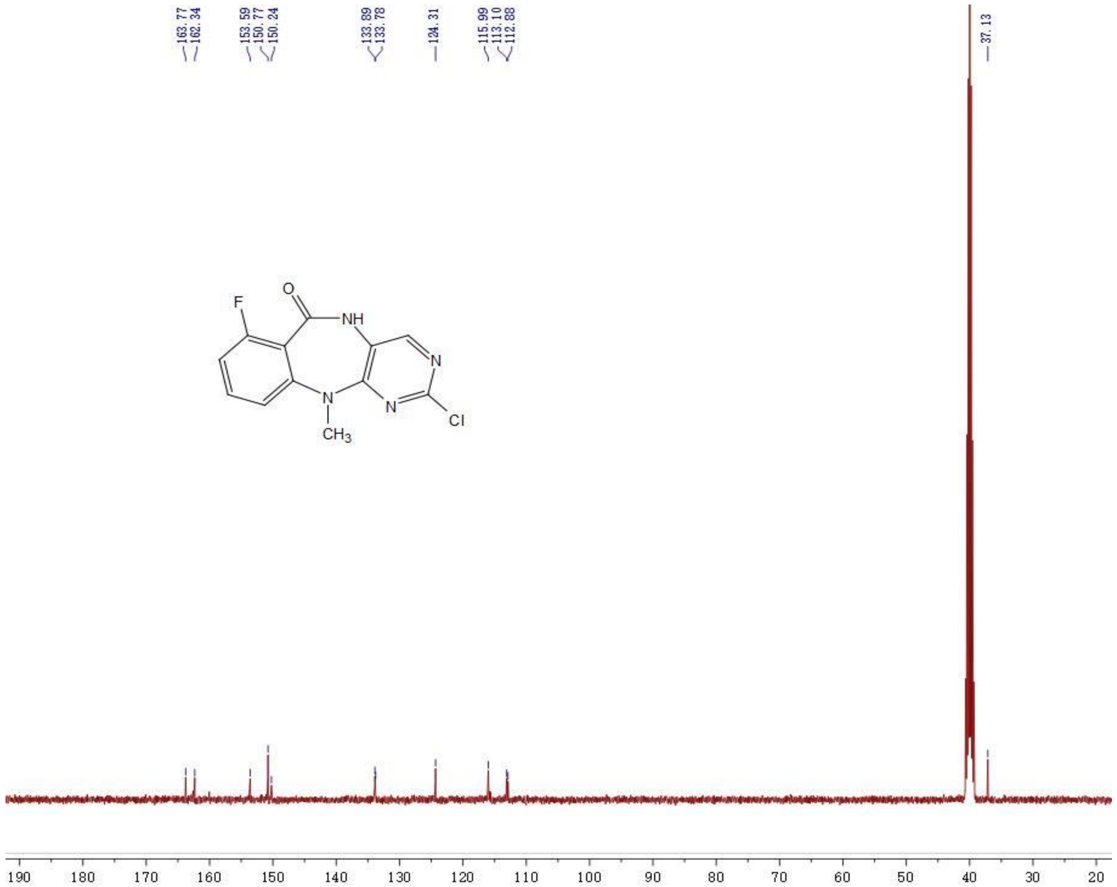

The $^{1}H/^{13}C$ NMR spectrum of intermediate **4-10**

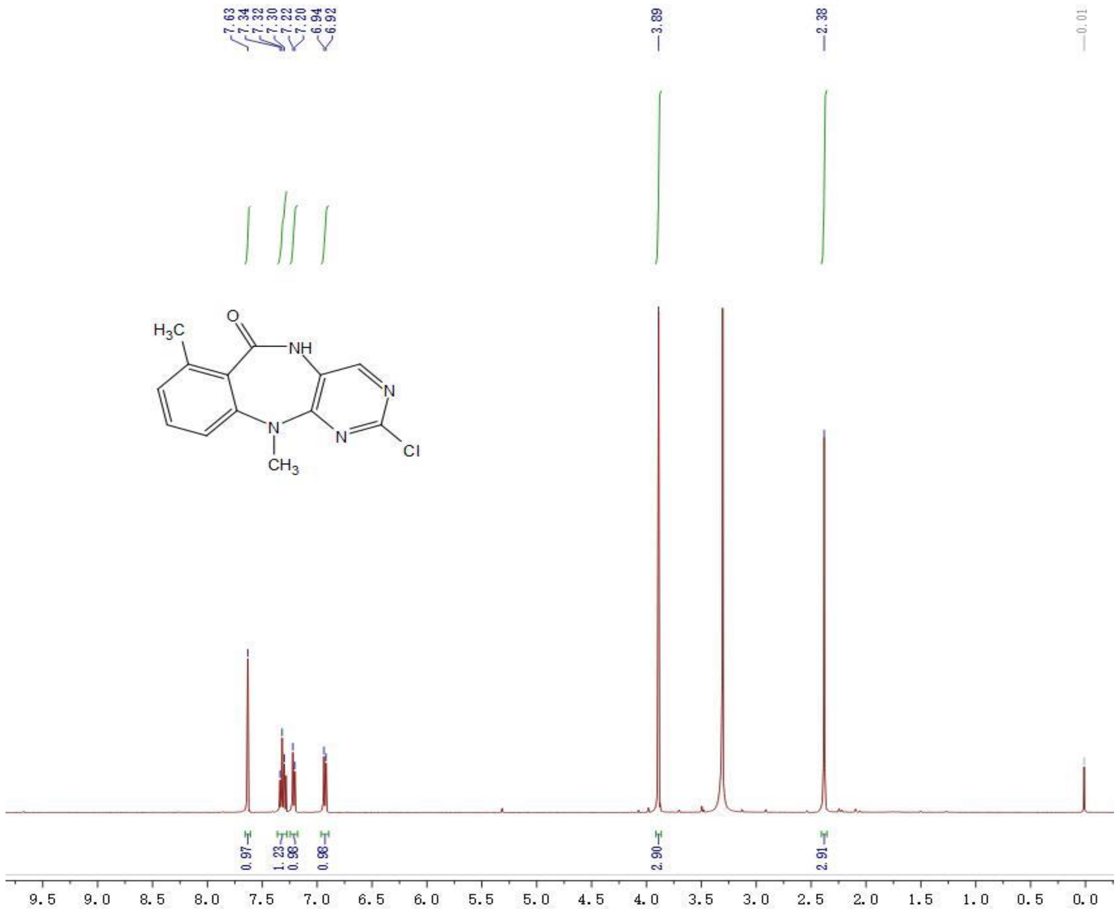

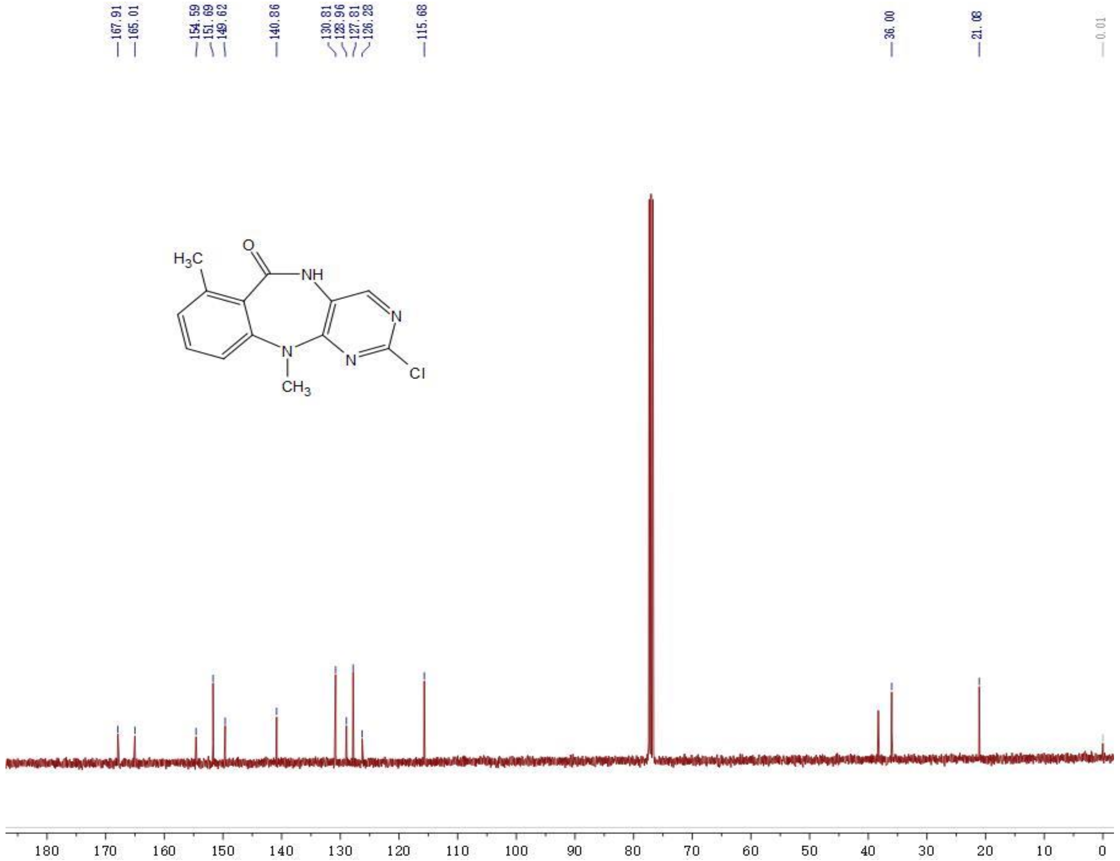

The $^{1}H/^{13}C$ NMR spectrum of intermediate **5-1**

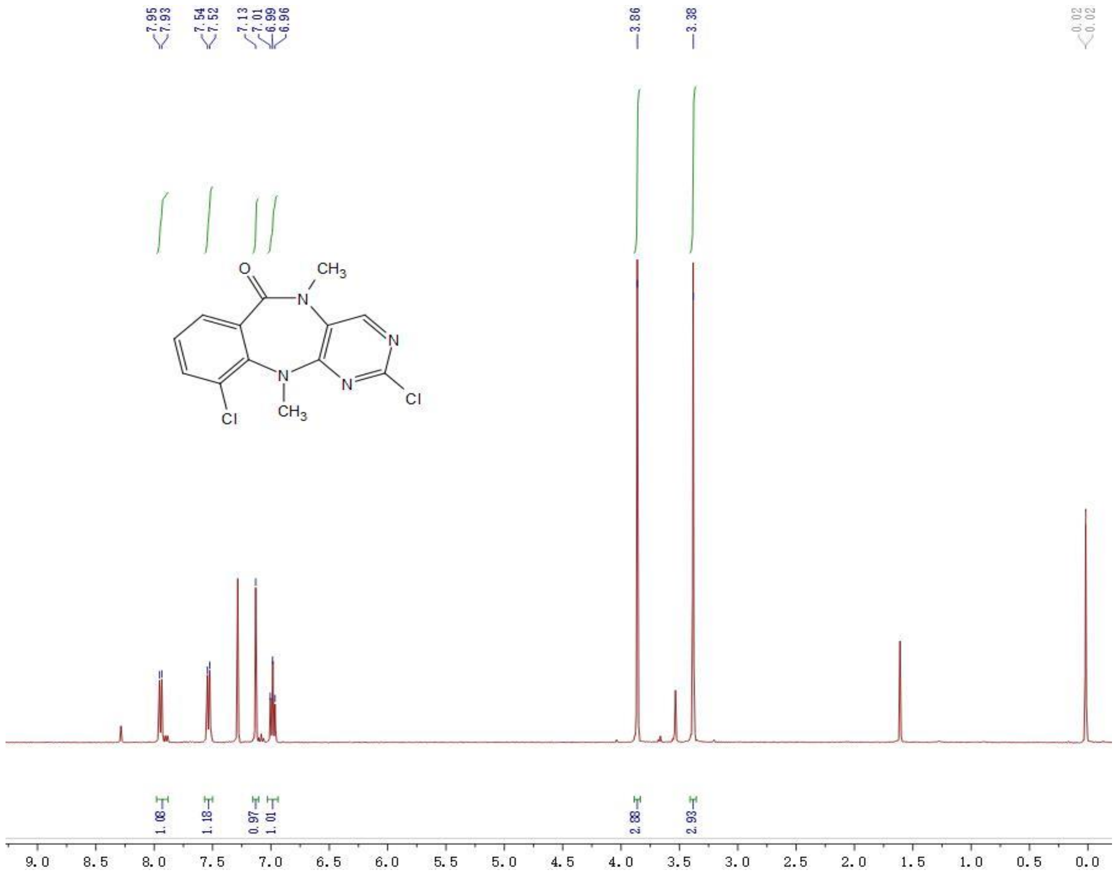

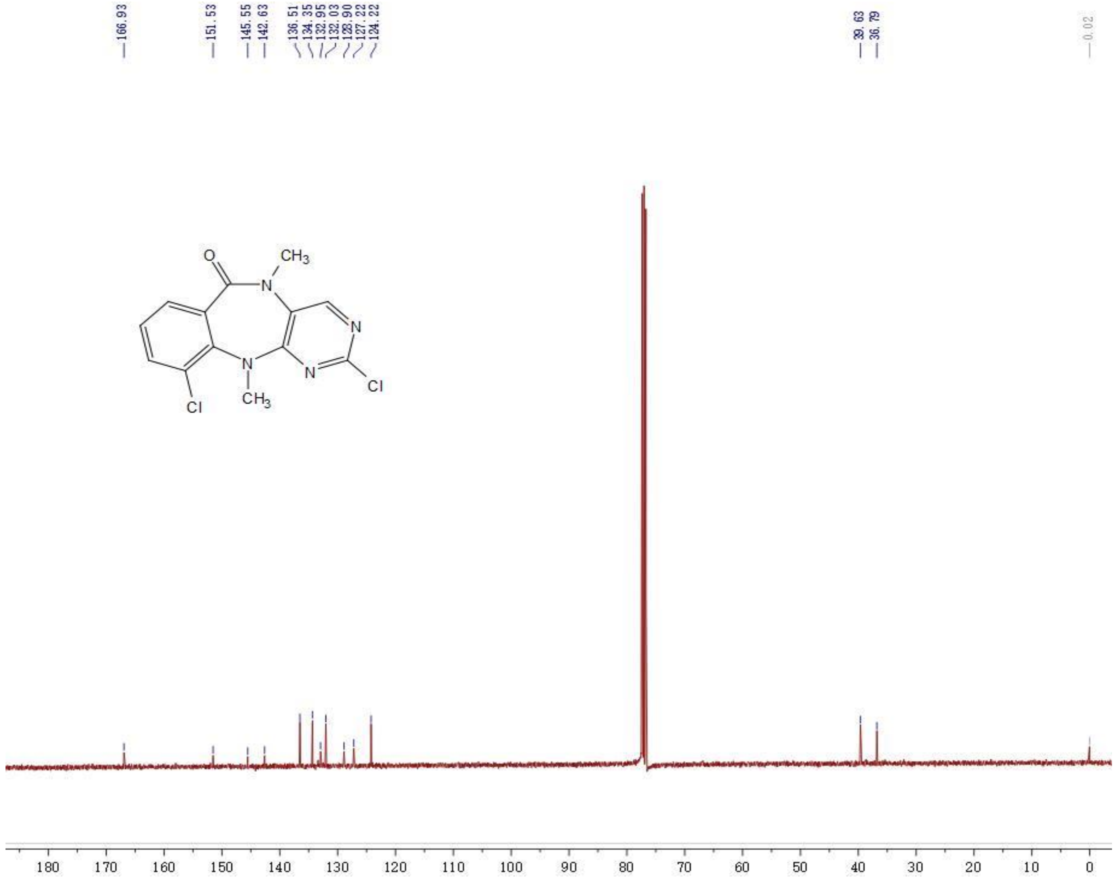

The $^{1}H/^{13}C$ NMR spectrum of intermediate **5-2**

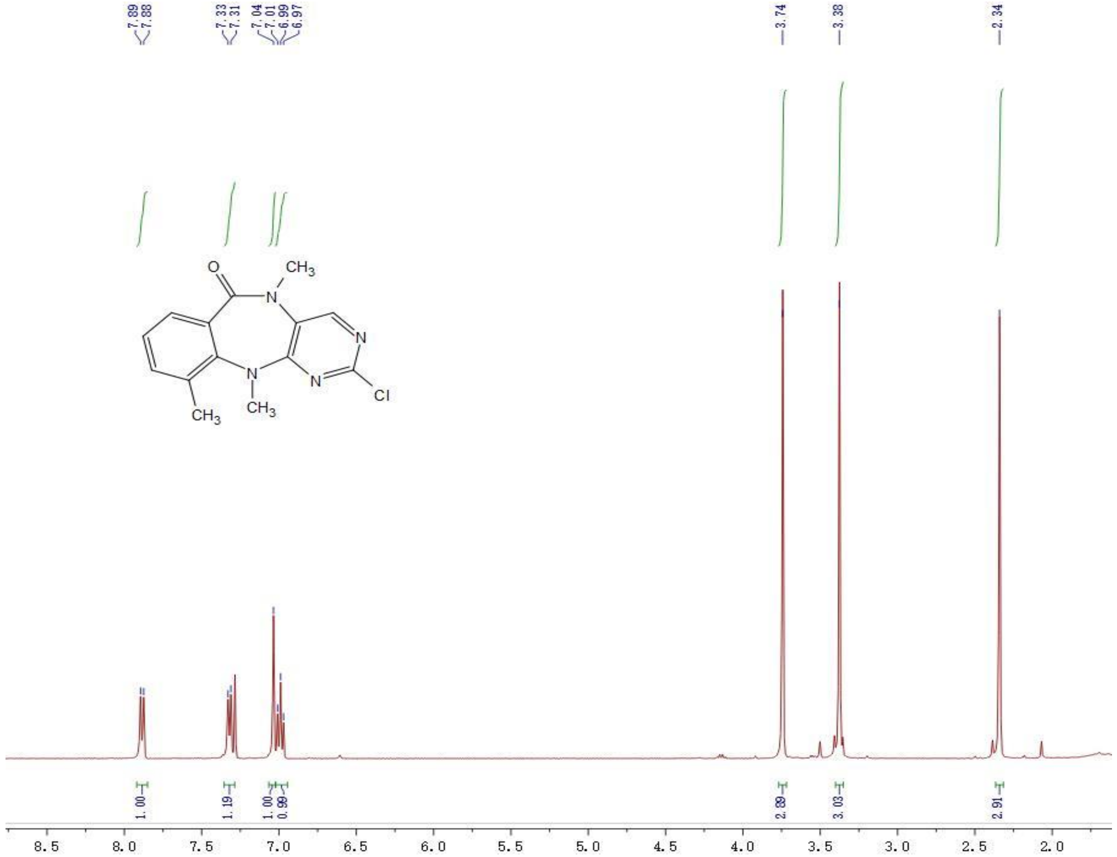

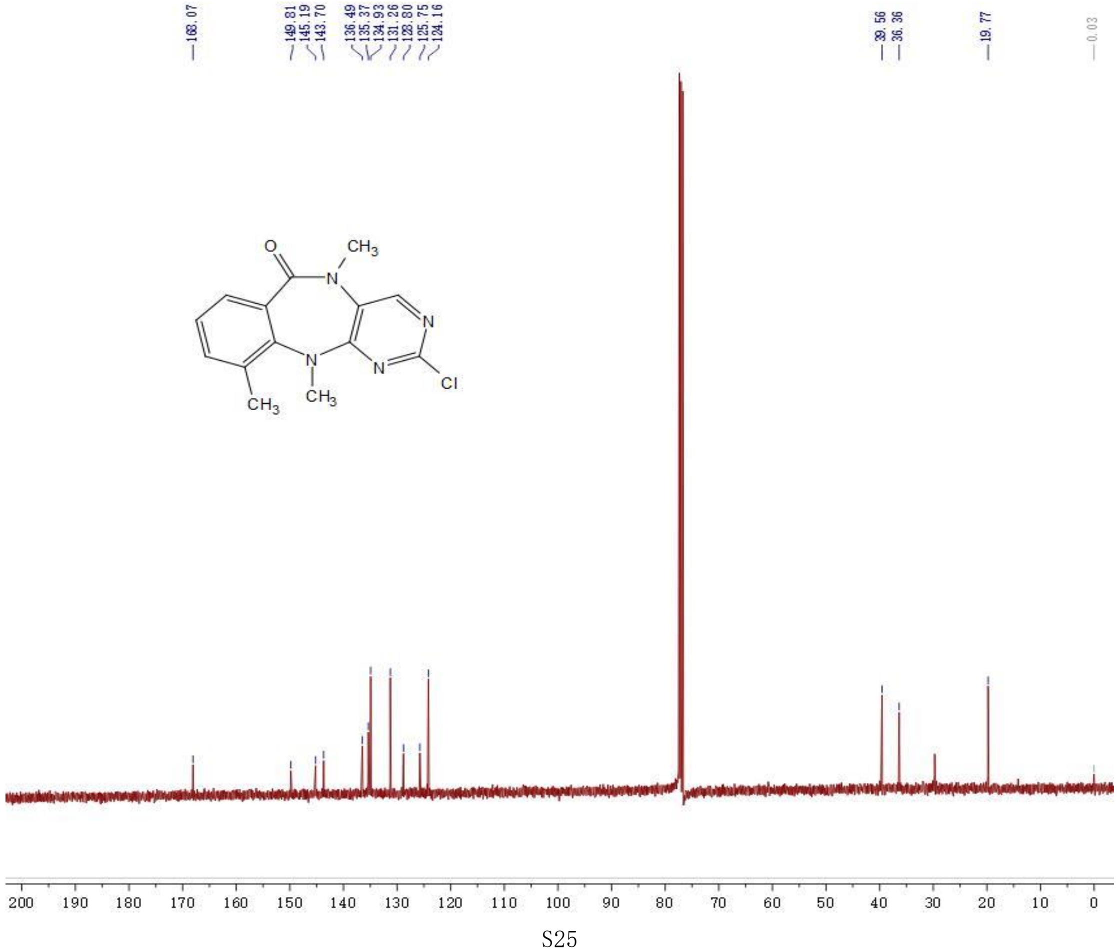

The $^{1}H/^{13}C$ NMR spectrum of intermediate **5-3**

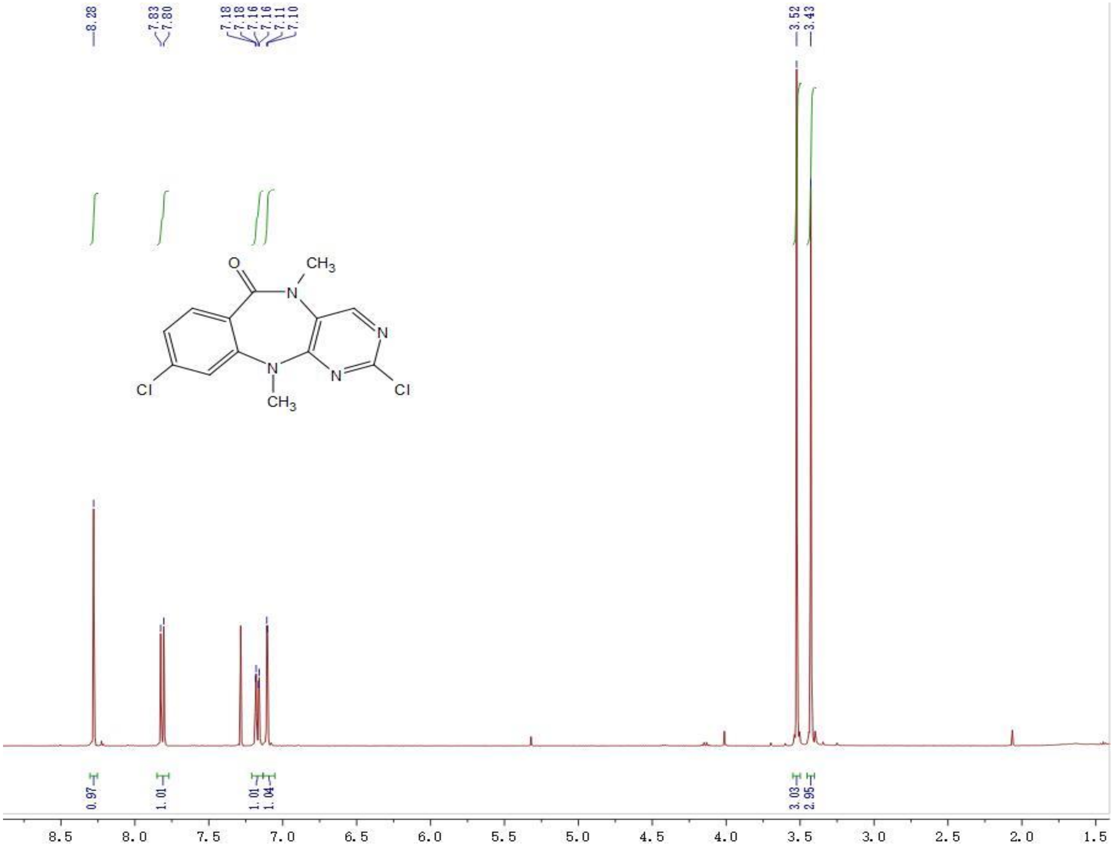

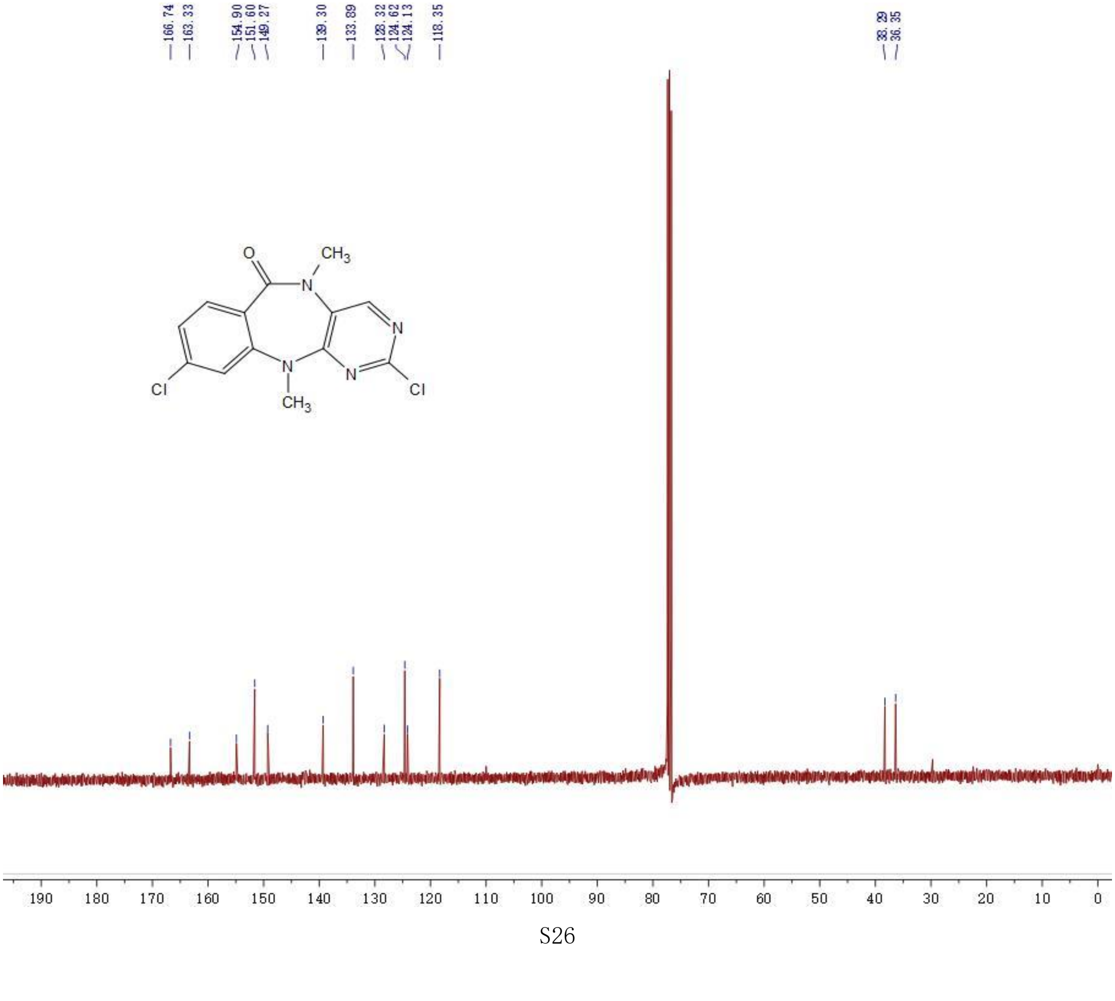

The $^{1}H/^{13}C$ NMR spectrum of intermediate **5-4**

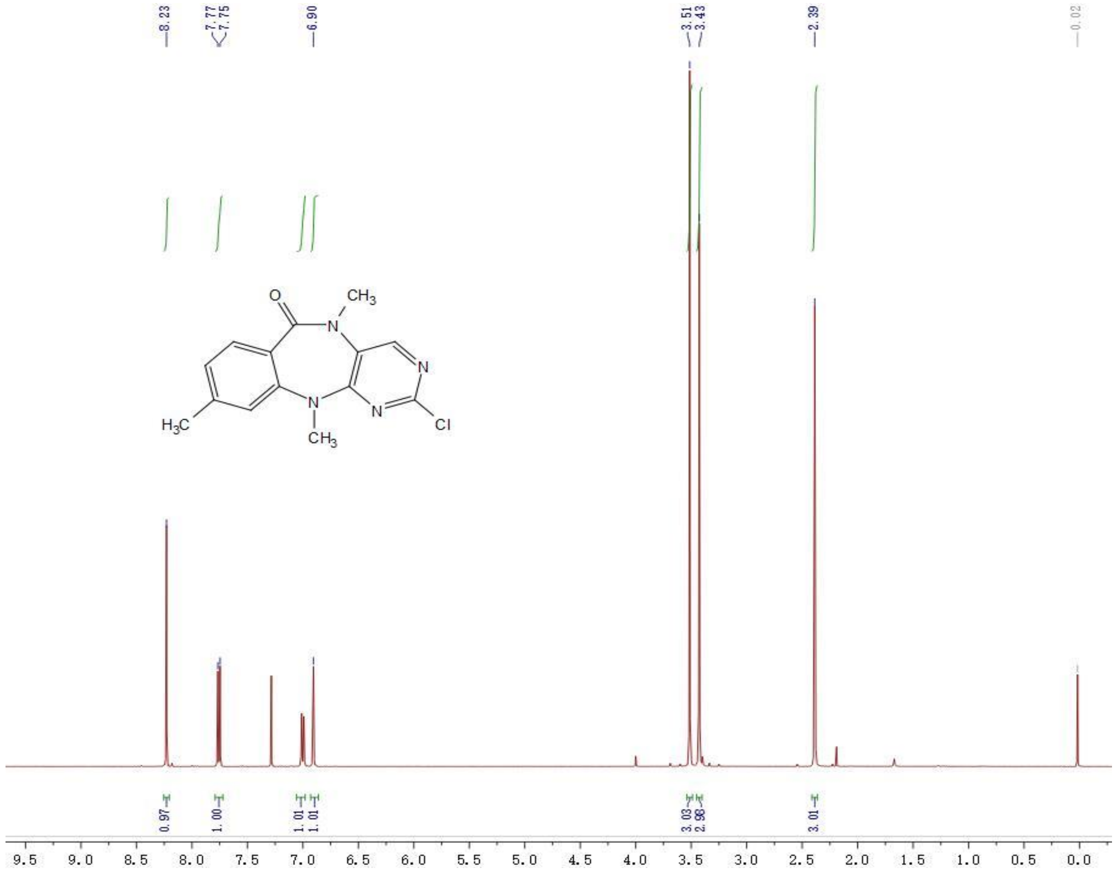

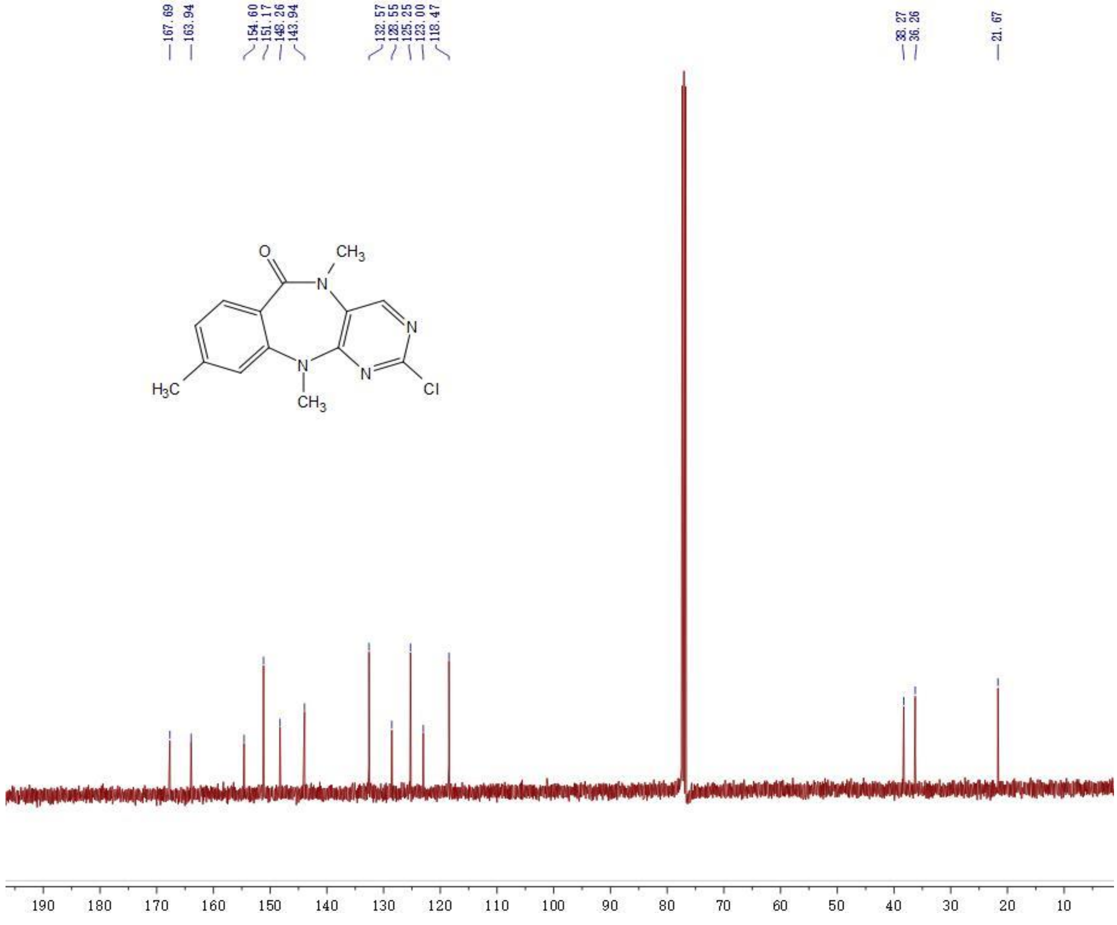

The $^{1}H/^{13}C$ NMR spectrum of intermediate **5-5**

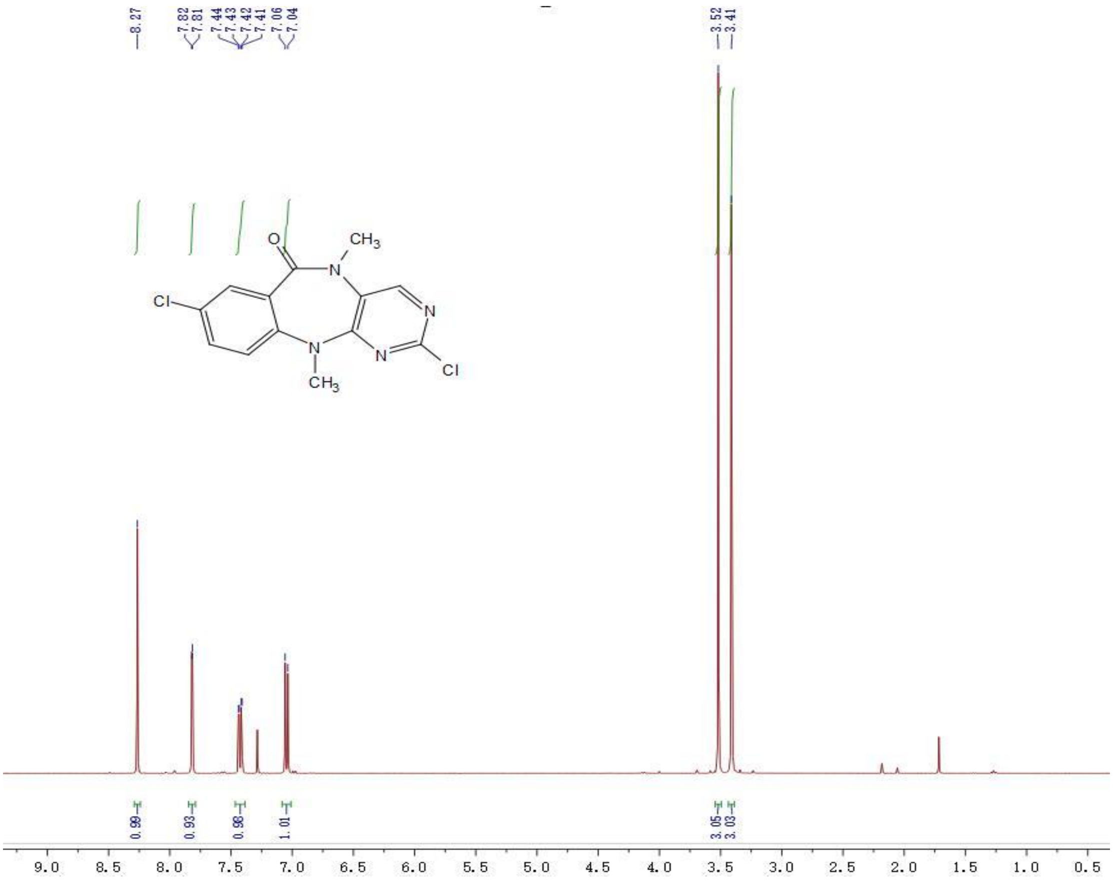

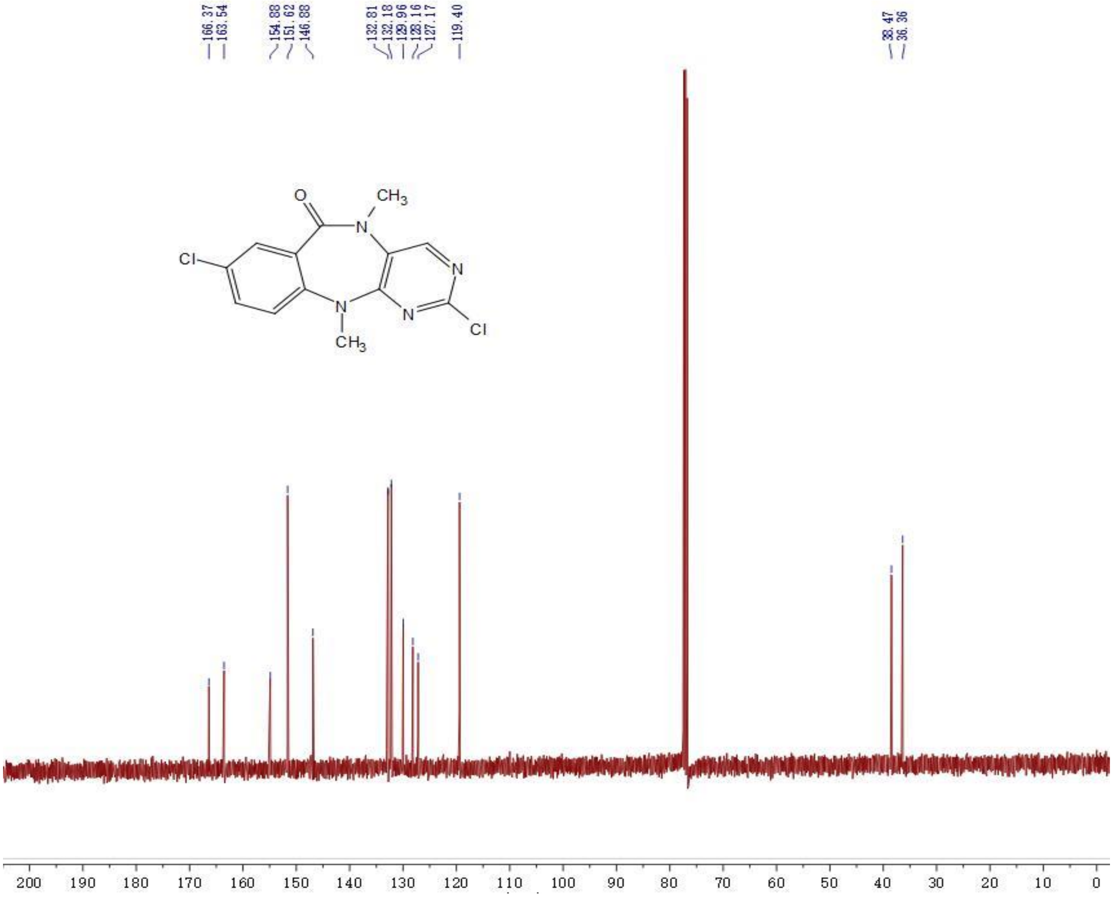

The $^{1}H/^{13}C$ NMR spectrum of intermediate **5-6**

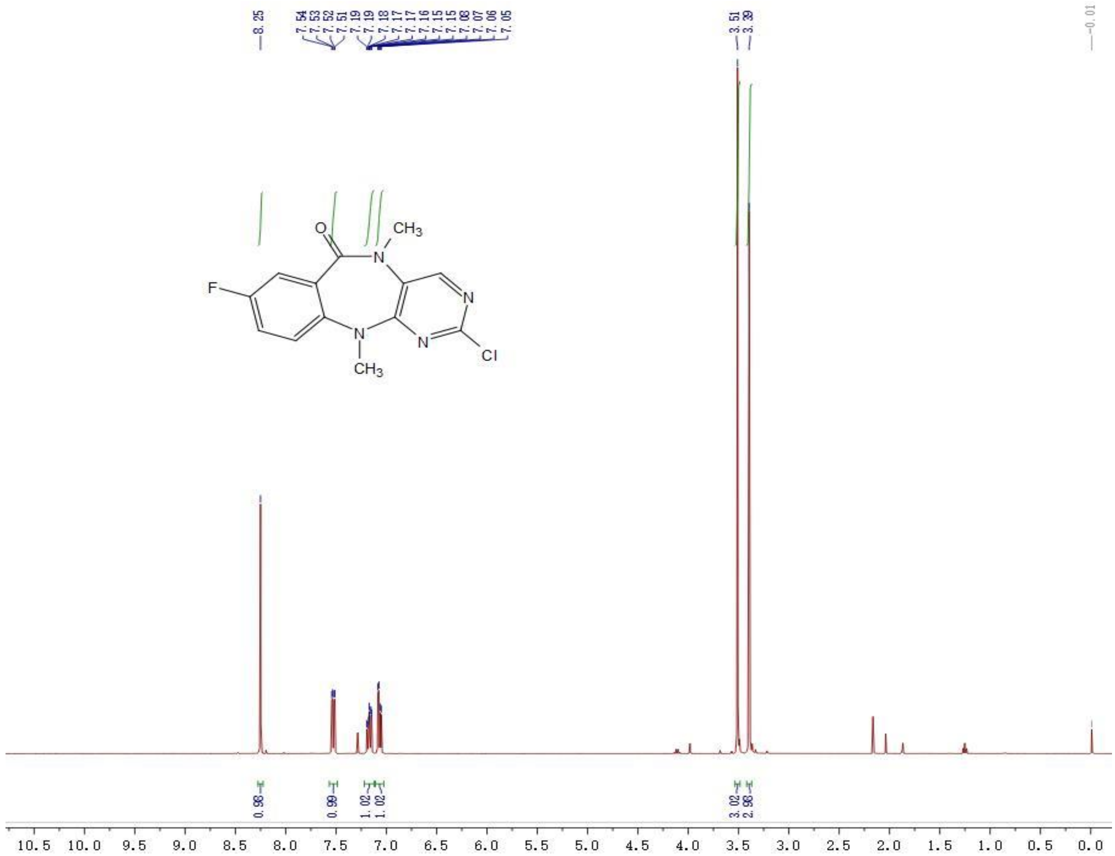

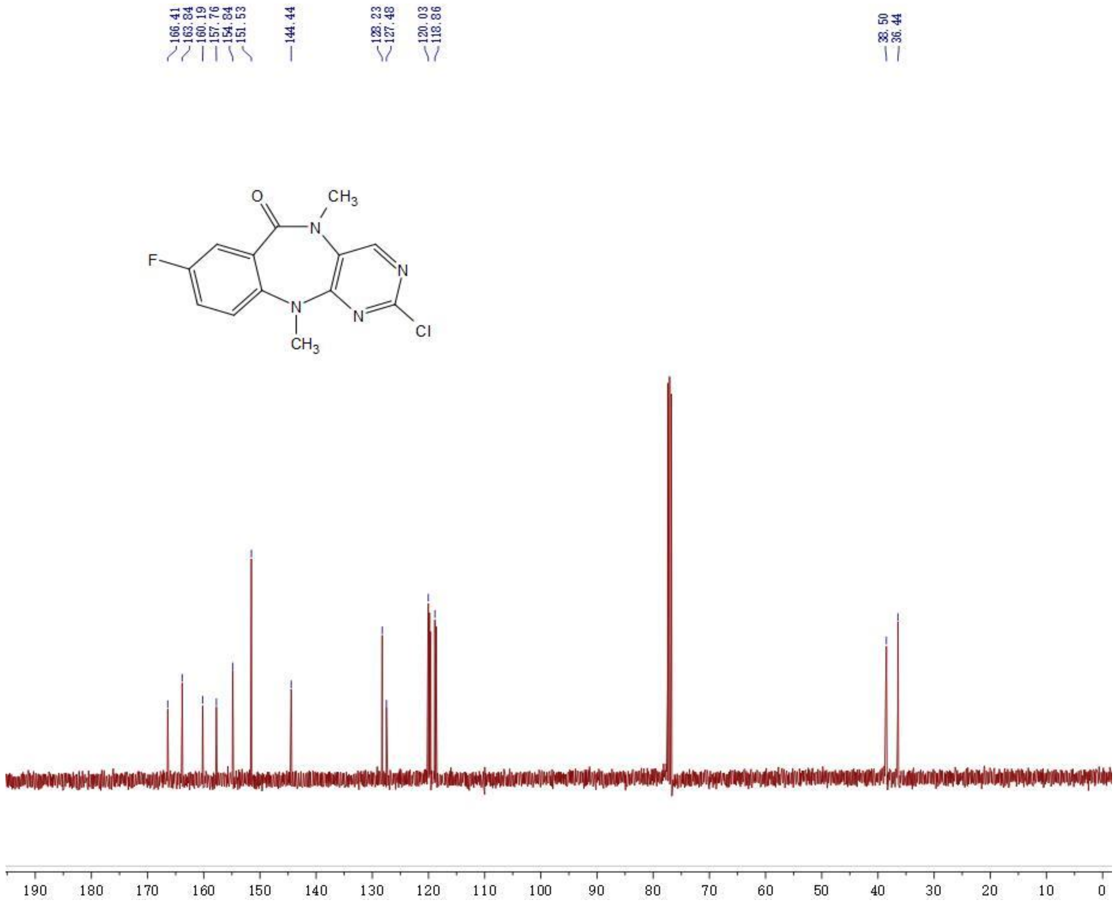

The $^{1}H/^{13}C$ NMR spectrum of intermediate **5-7**

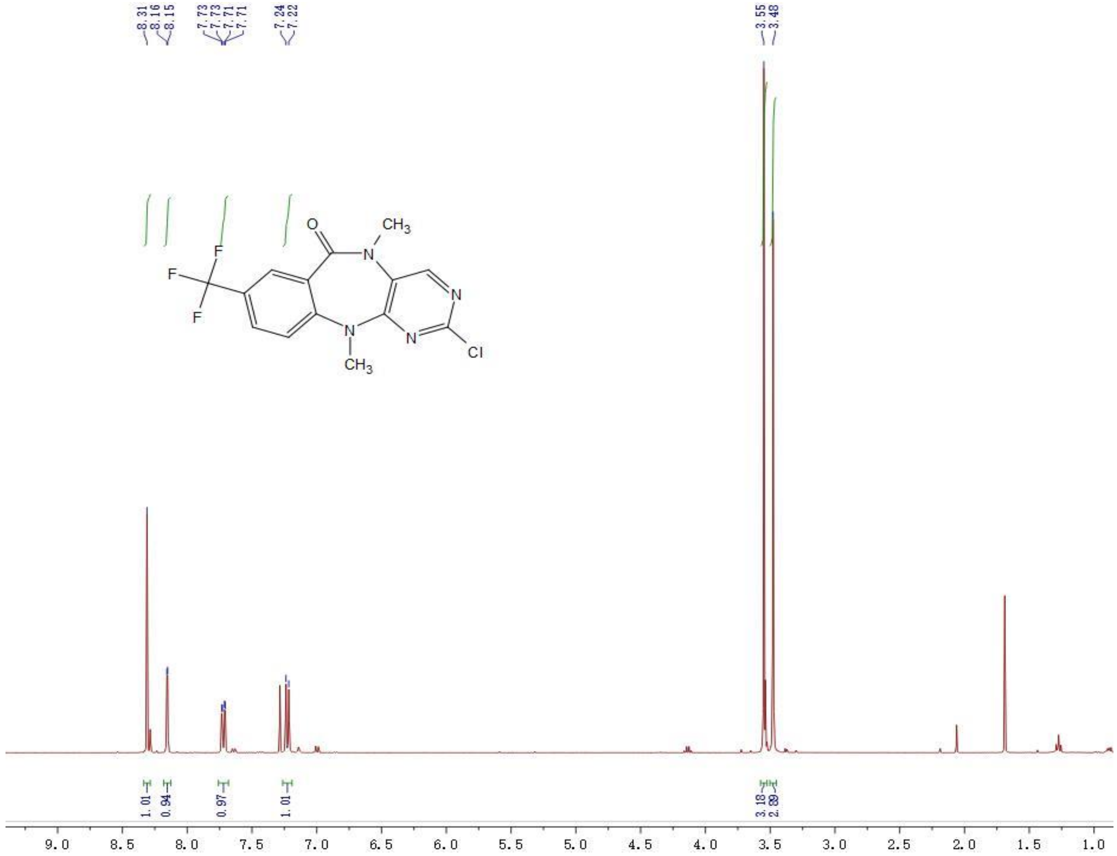

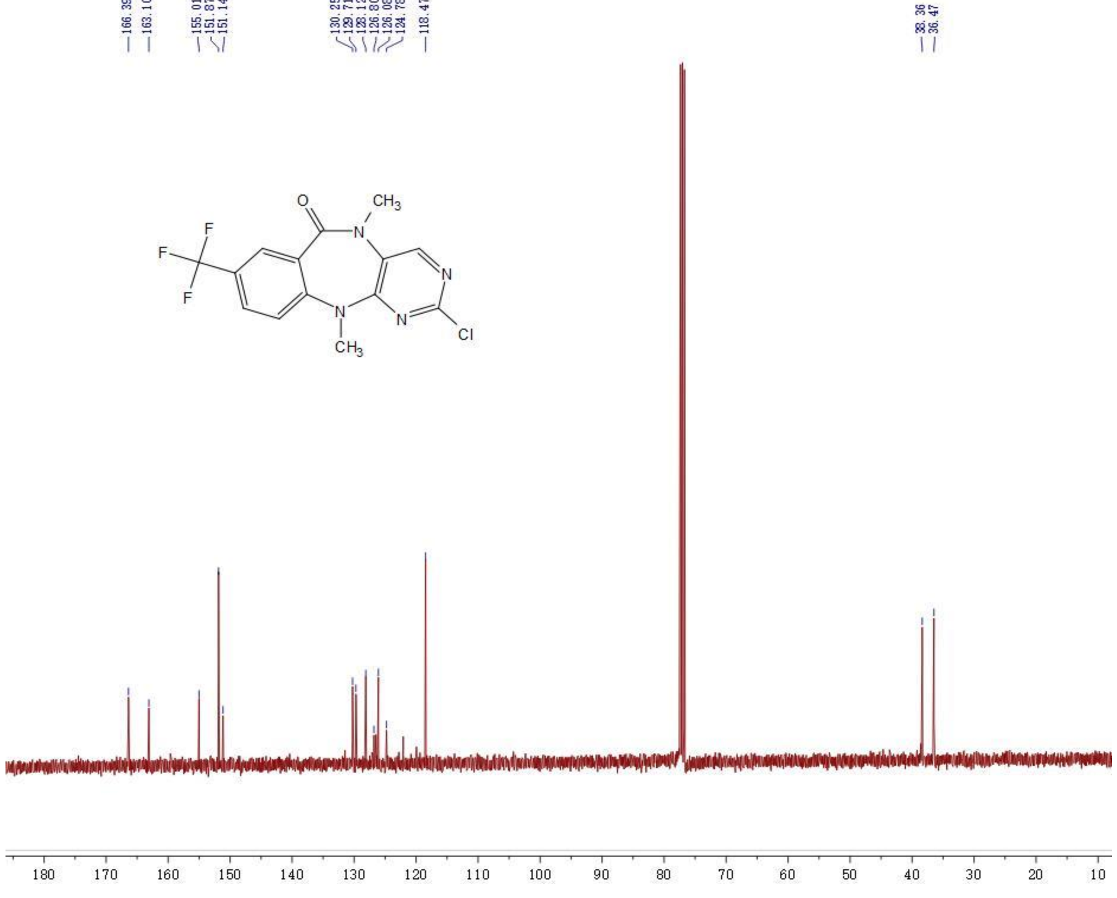

The $^{1}H/^{13}C$ NMR spectrum of intermediate **5-8**

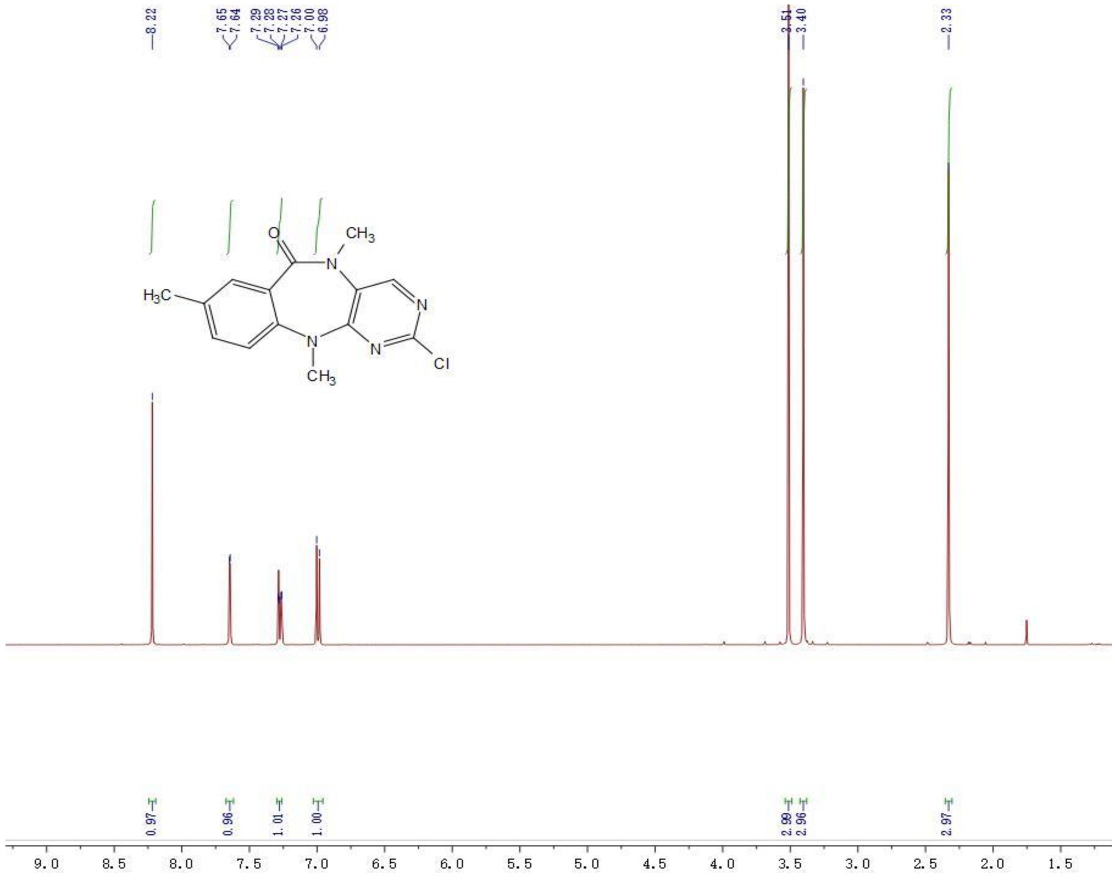

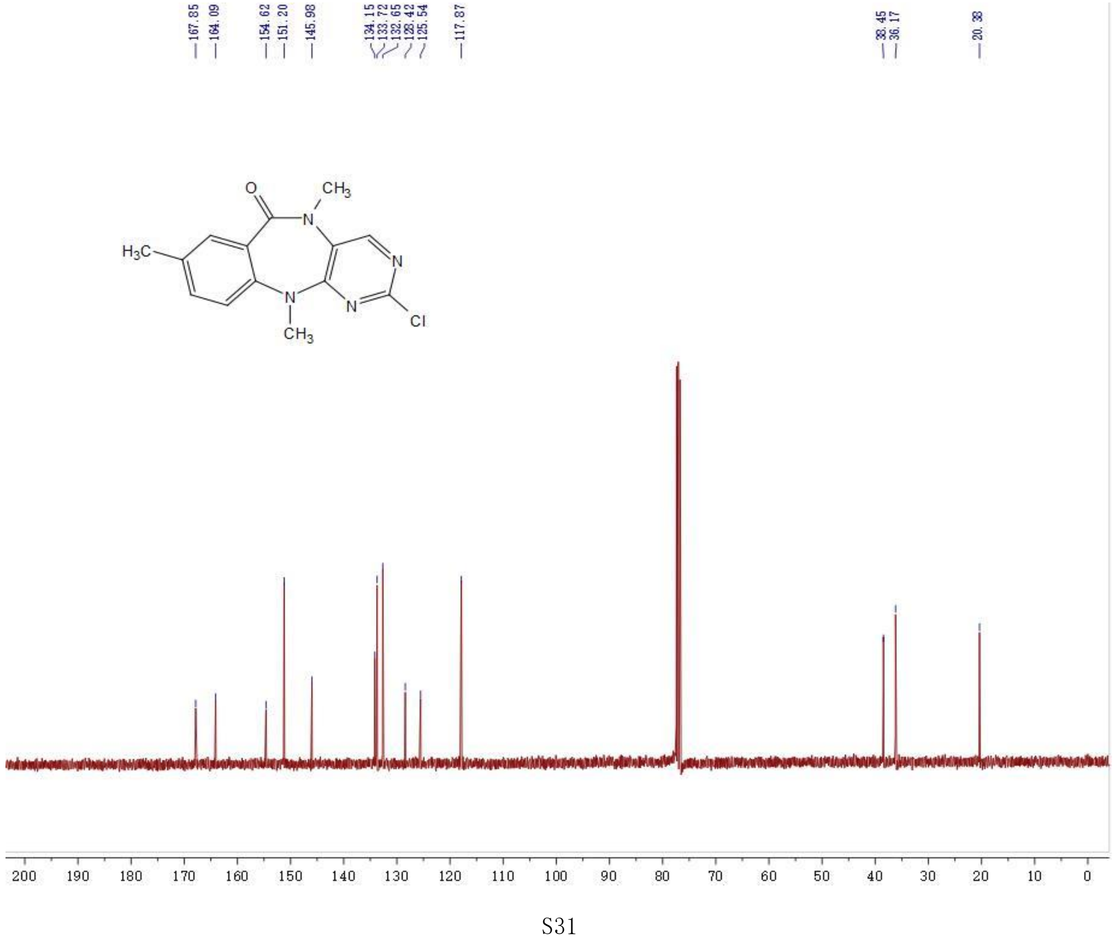

The $^{1}H/^{13}C$ NMR spectrum of intermediate **5-9**

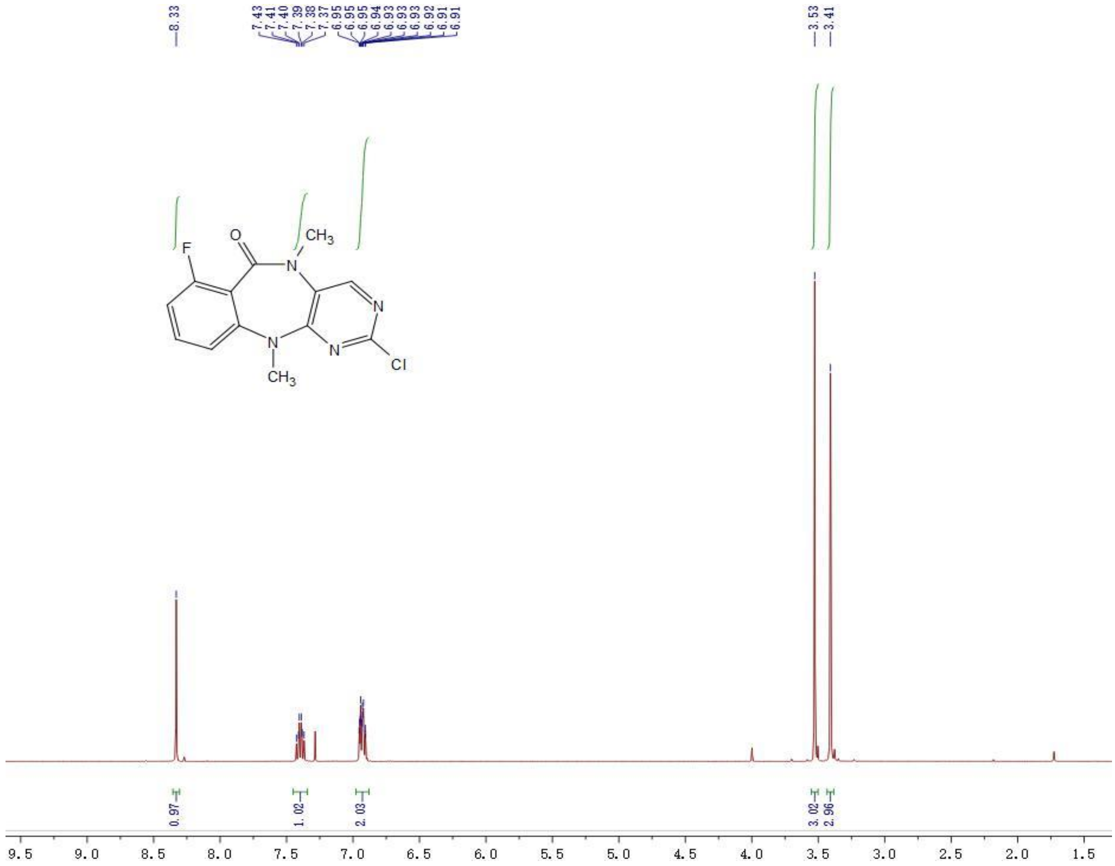

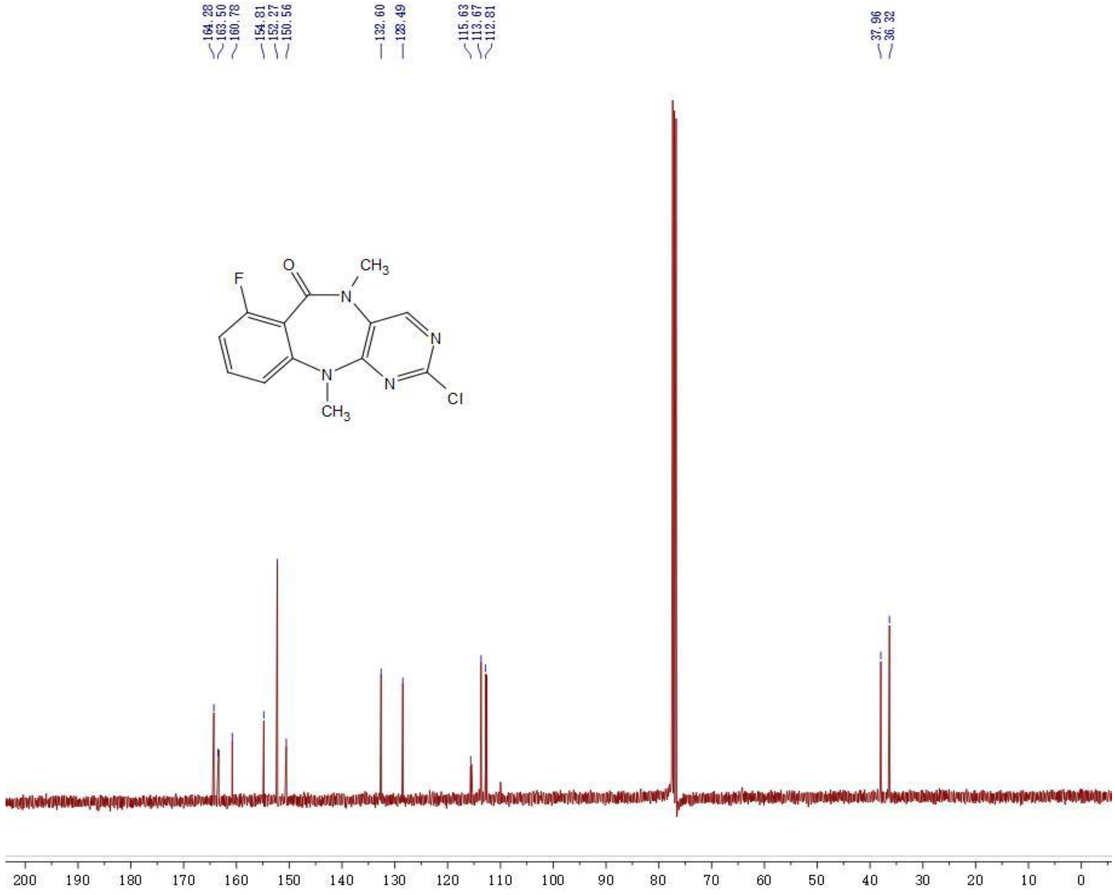

The $^{1}H/^{13}C$ NMR spectrum of intermediate **5-10**

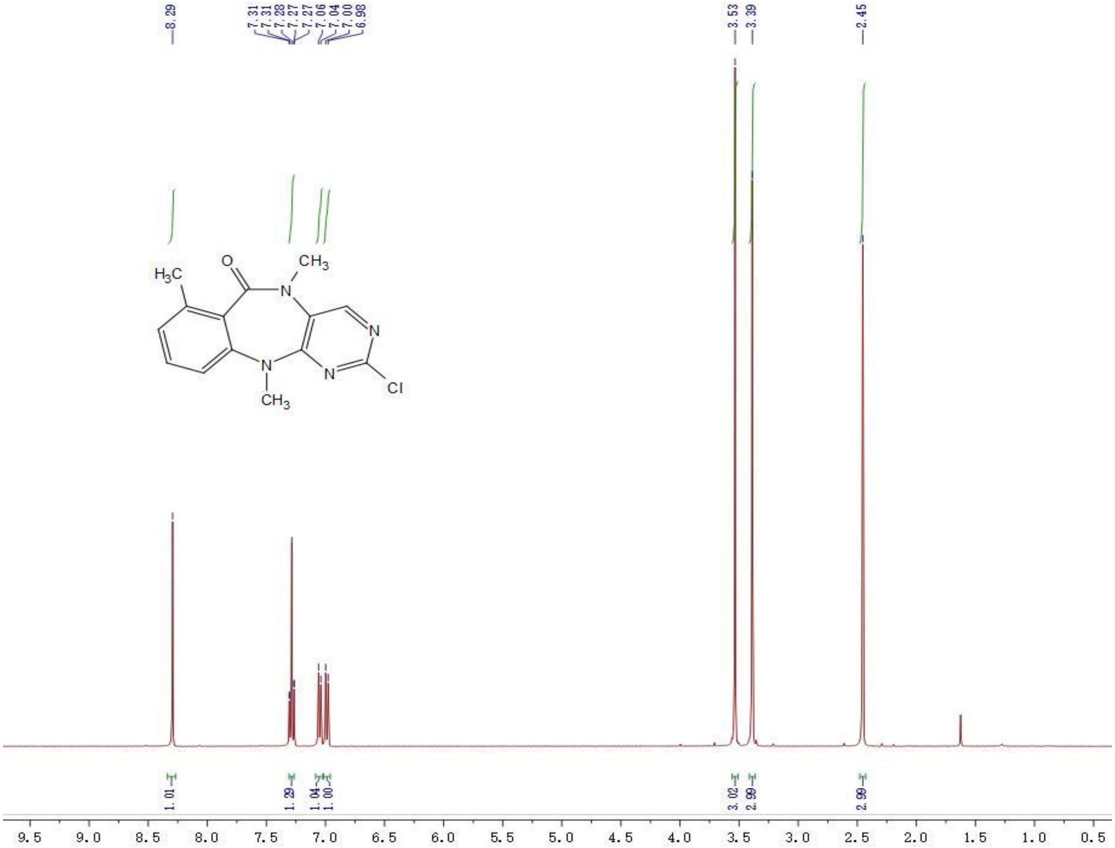

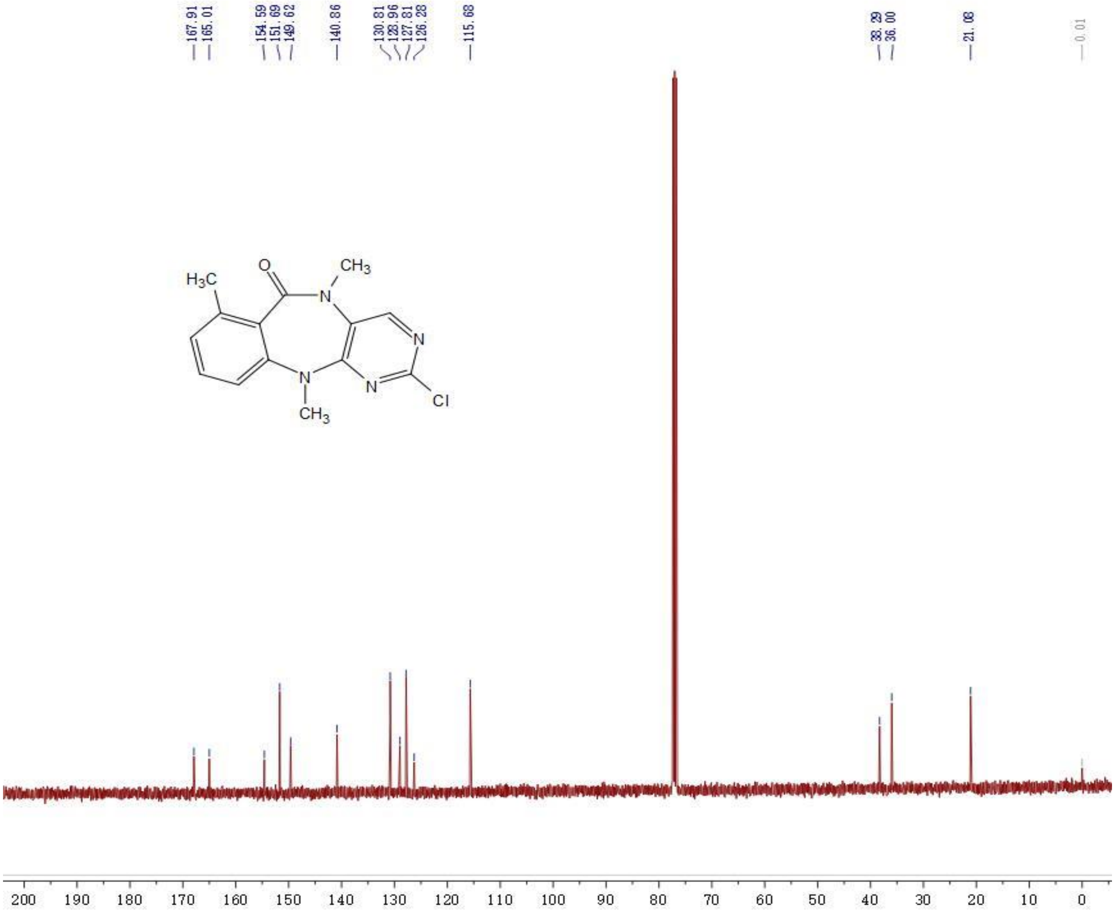

The $^{1}H/^{13}C$ NMR spectrum of Compound **A1**

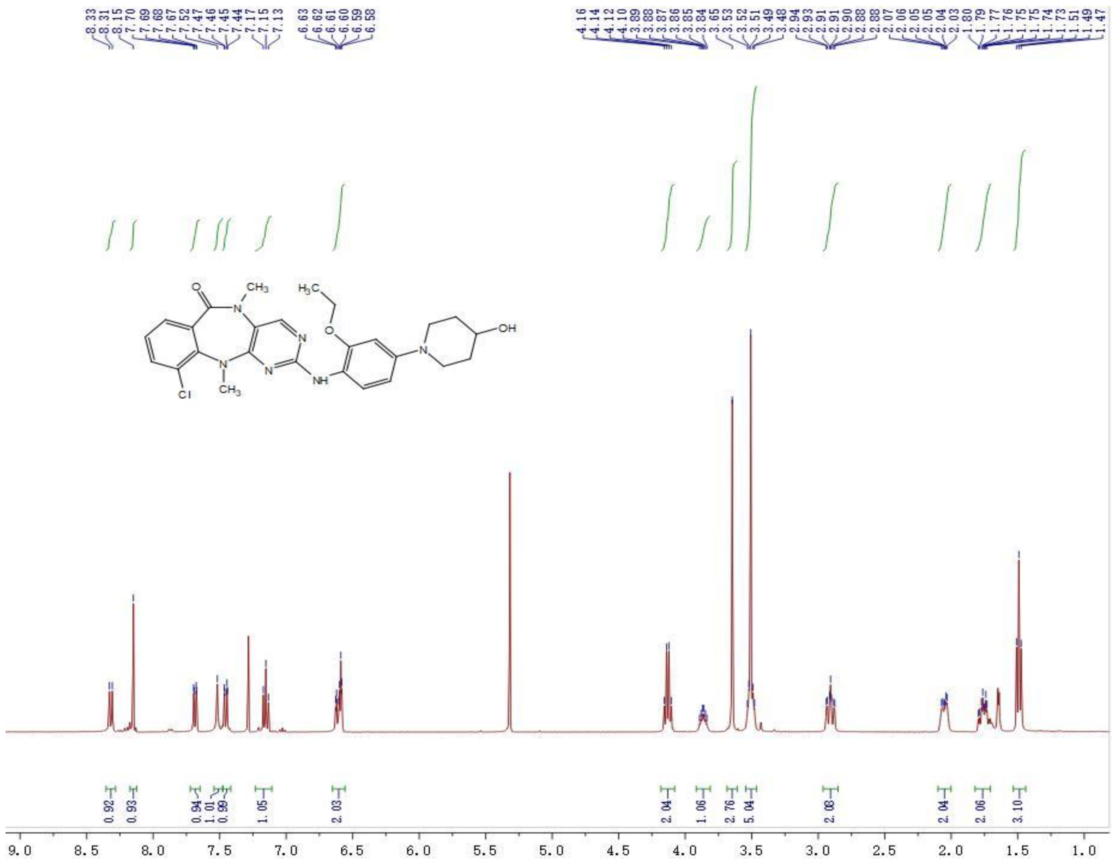

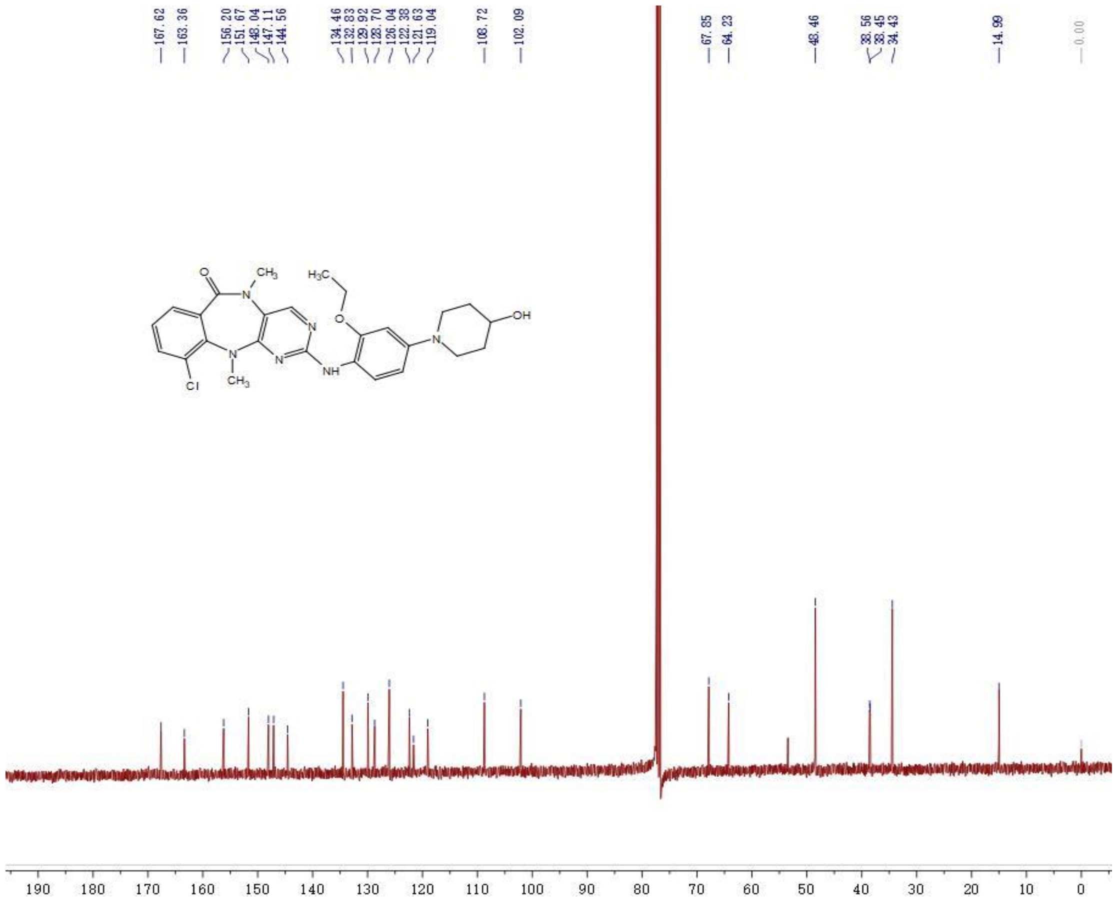

The $^{1}H/^{13}C$ NMR spectrum of Compound **A2**

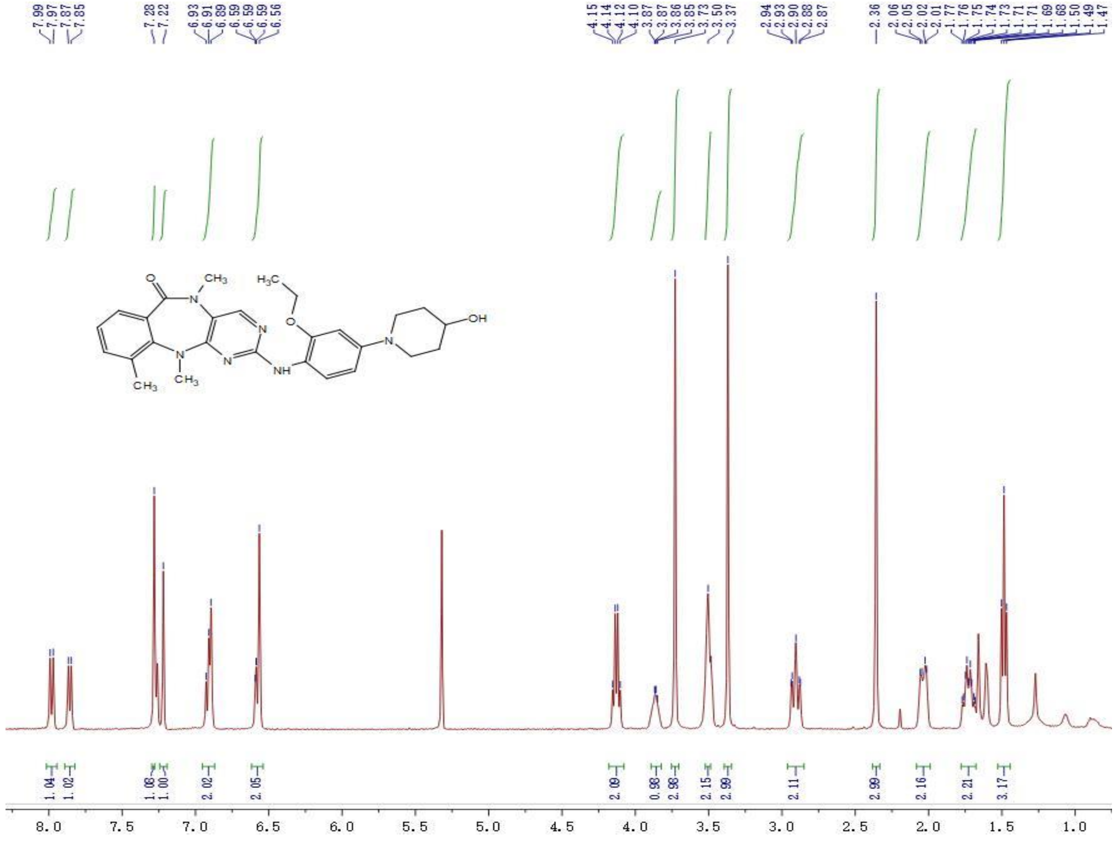

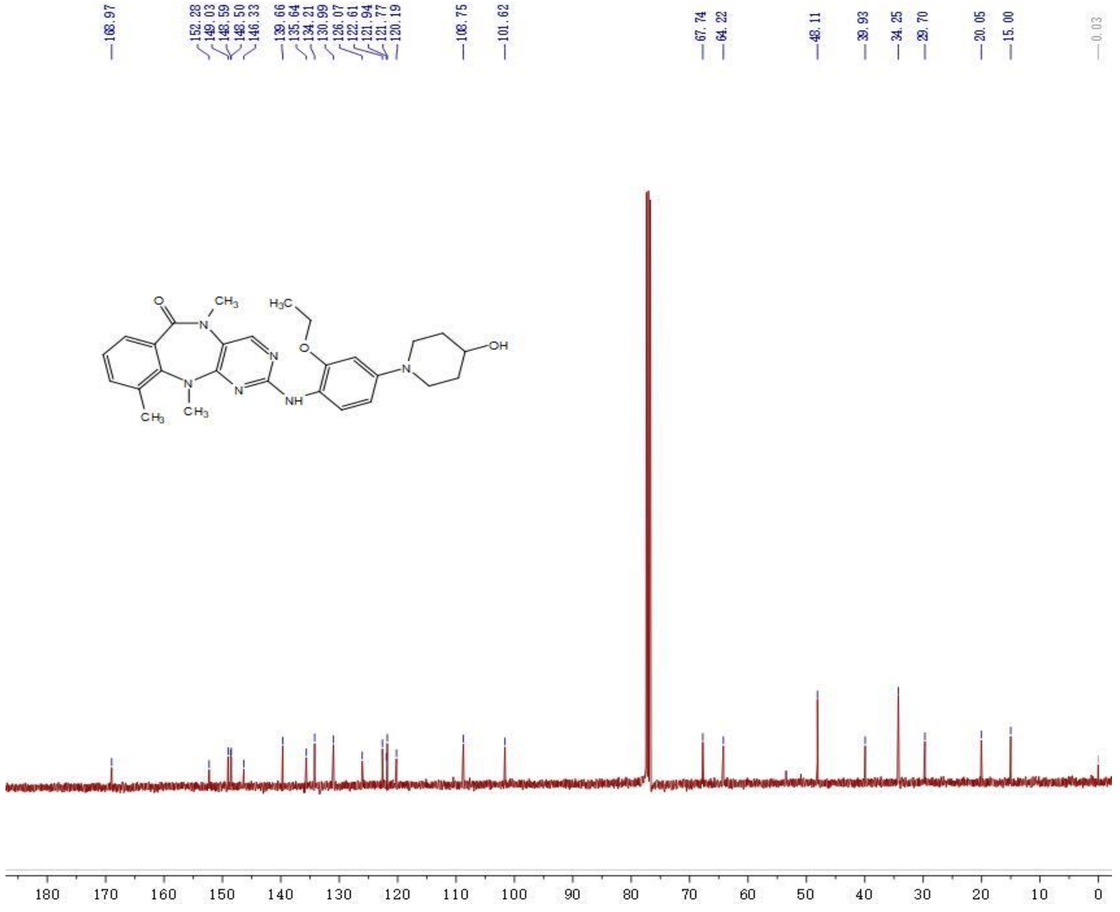

The ^{1}H/^{13}C NMR spectrum of Compound **A3**

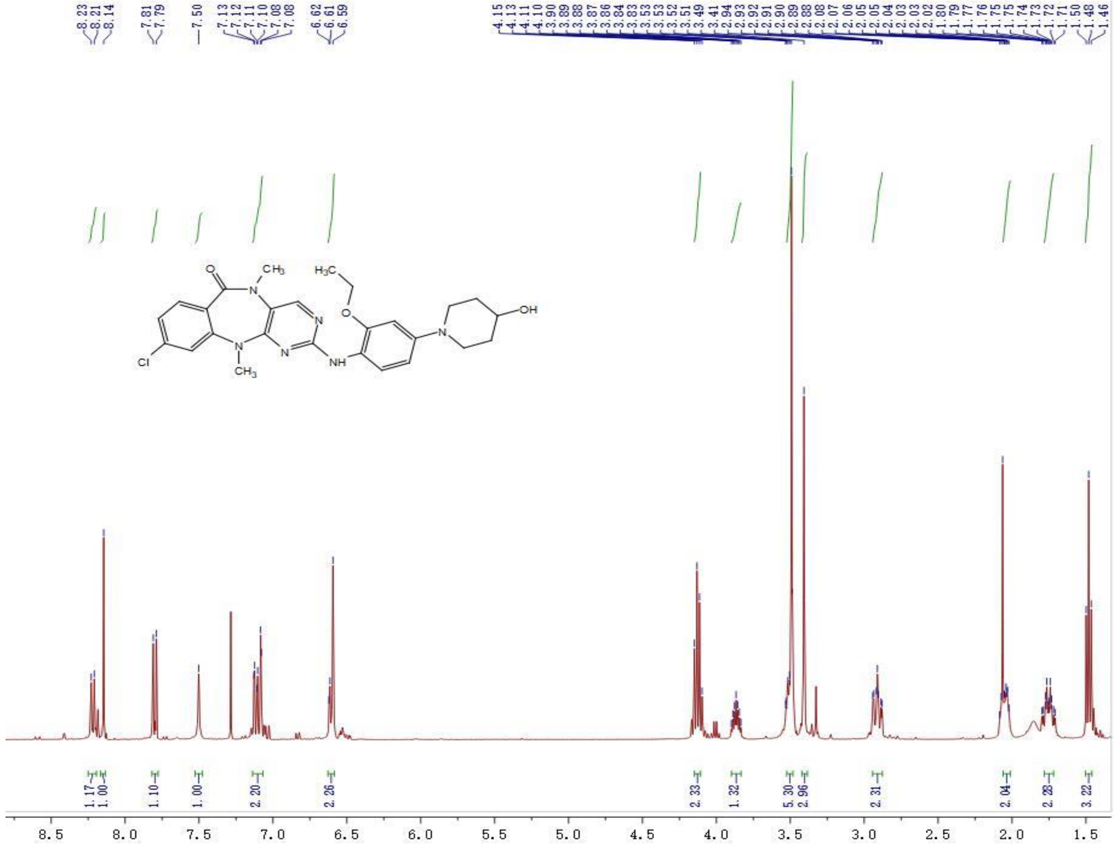

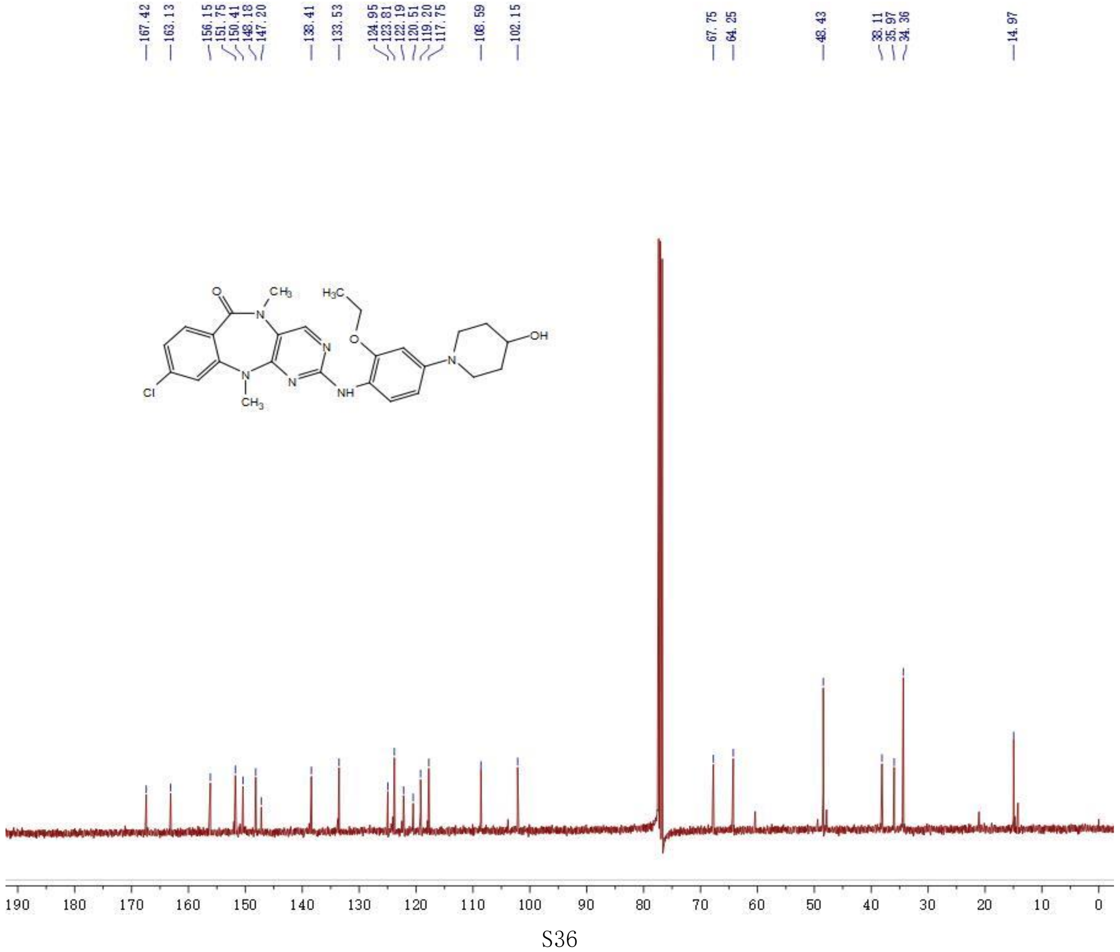

The $^{1}H/^{13}C$ NMR spectrum of Compound **A4**

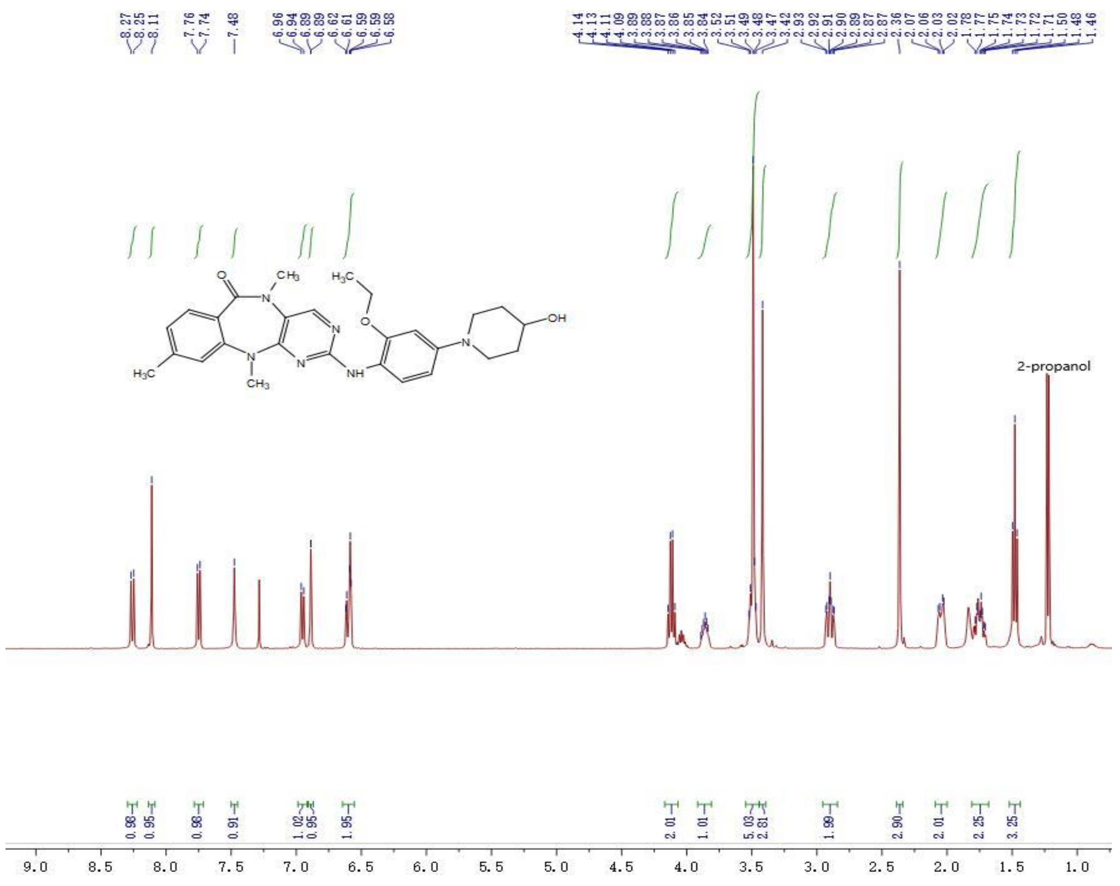

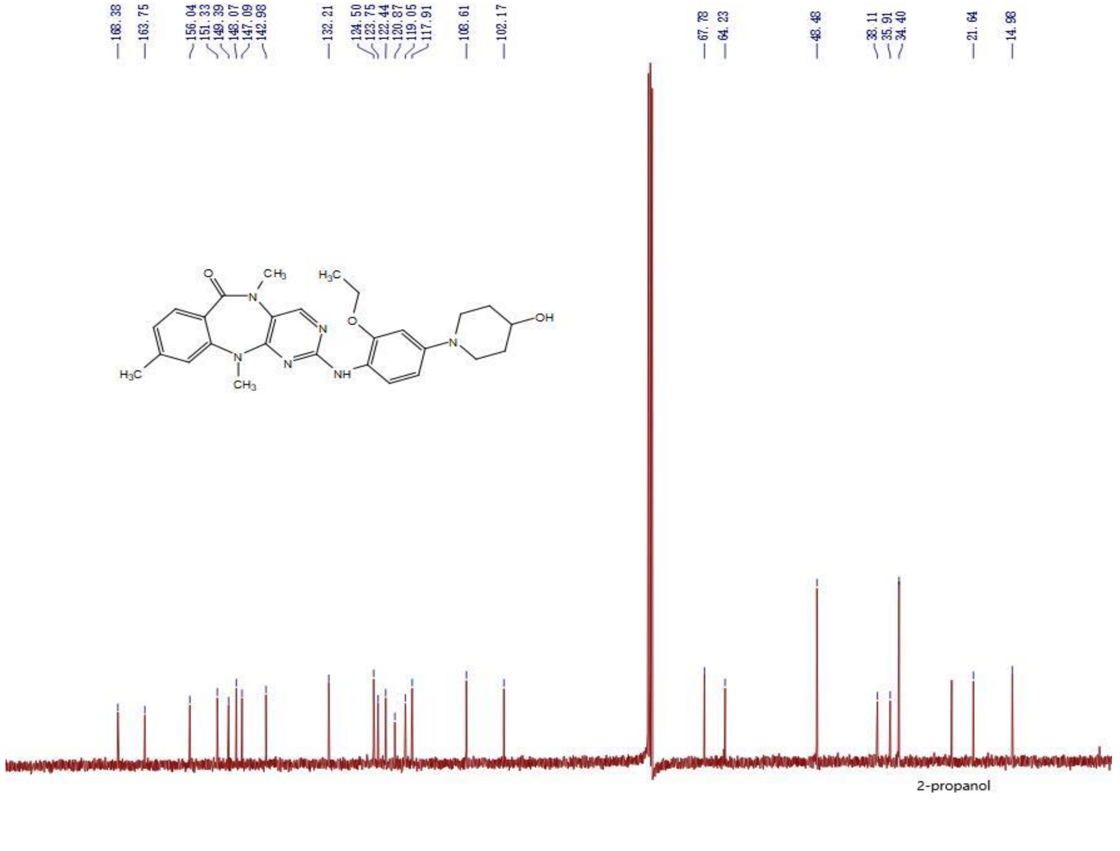

The $^{1}H/^{13}C$ NMR spectrum of Compound **A5**

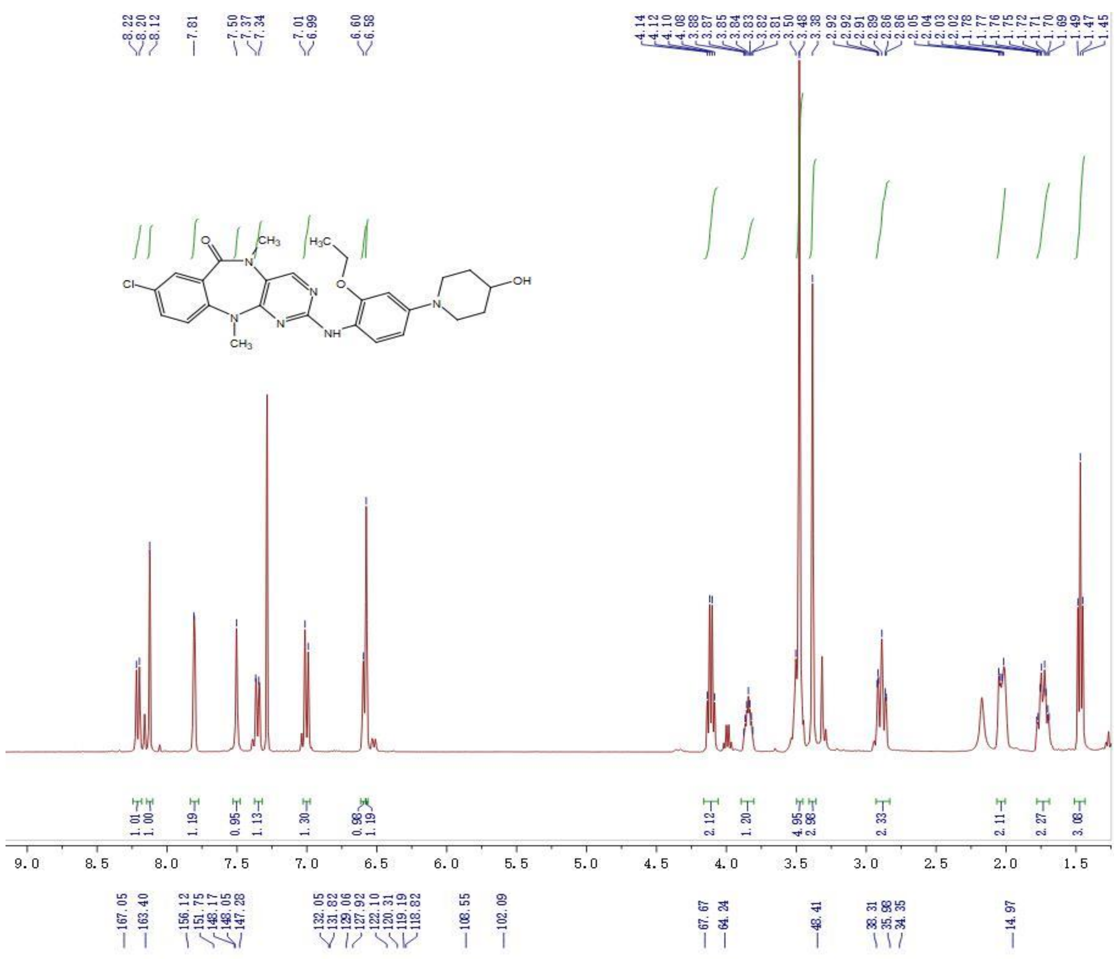

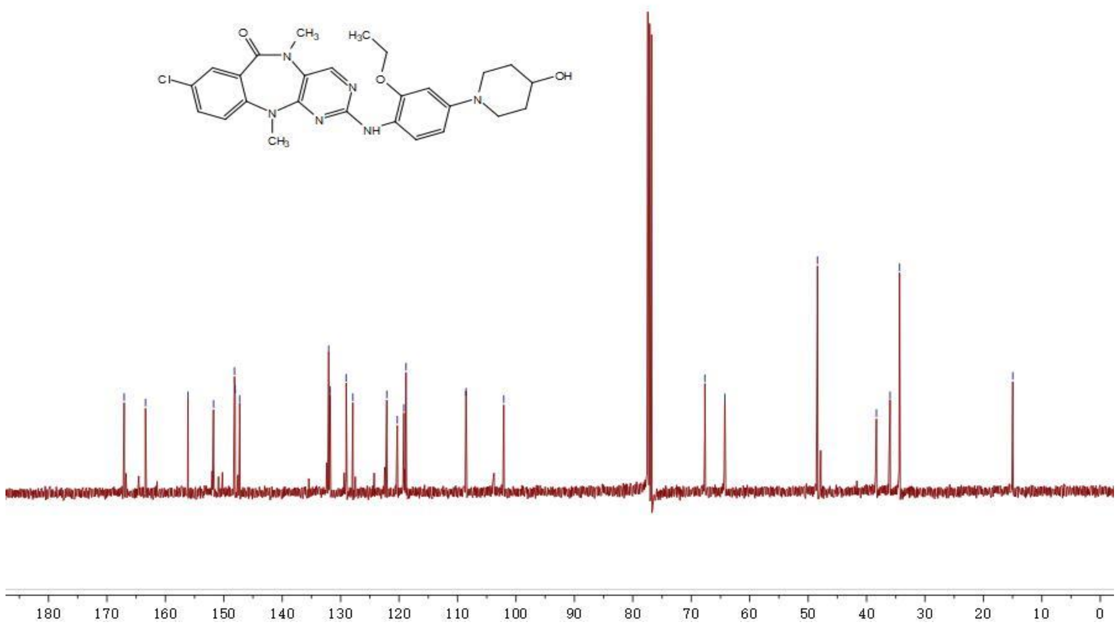

The $^{1}H/^{13}C$ NMR spectrum of Compound **A6**

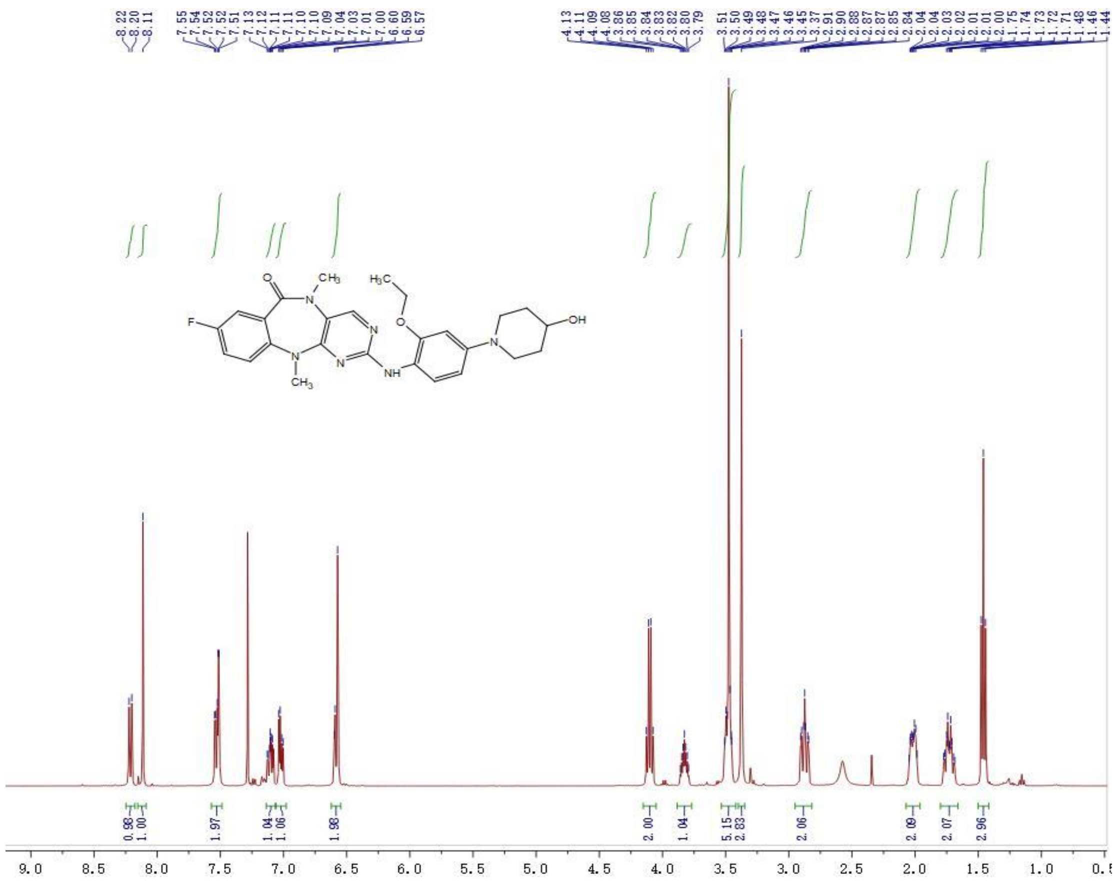

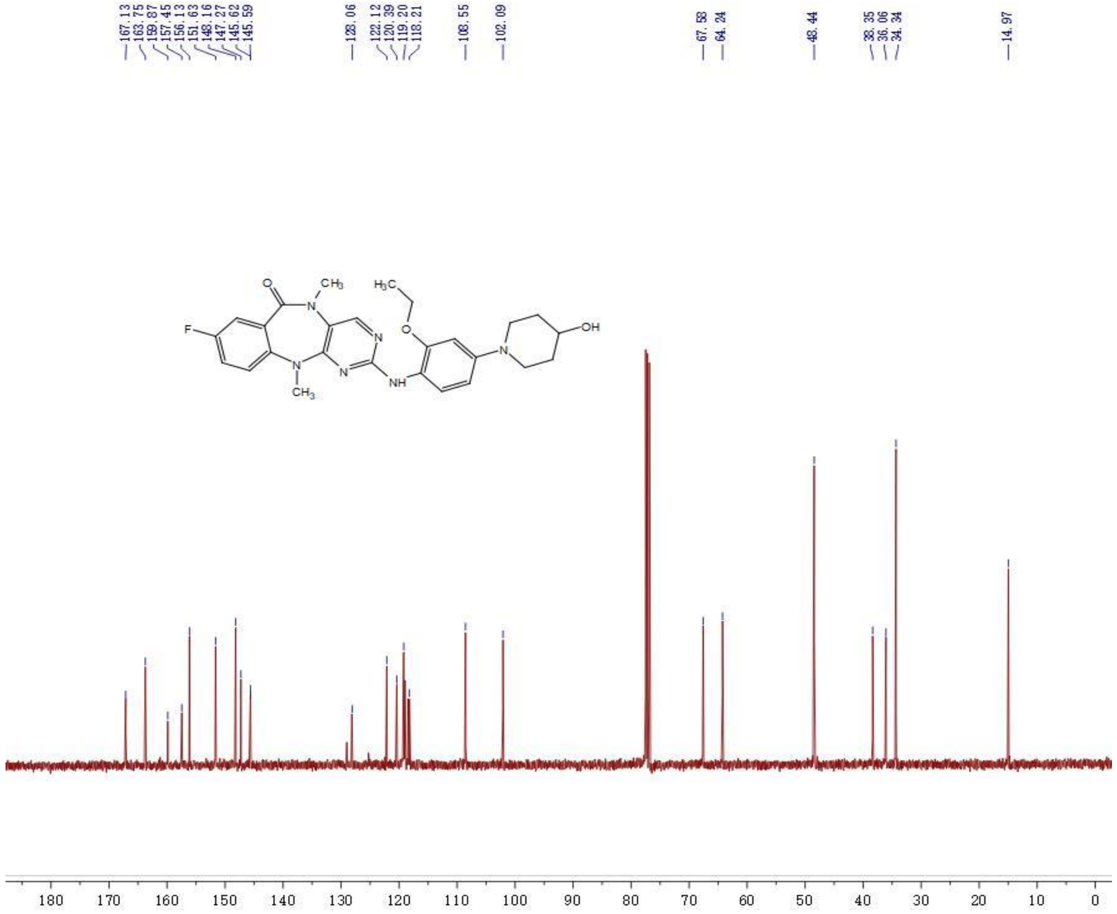

The $^{1}H/^{13}C$ NMR spectrum of Compound **A7**

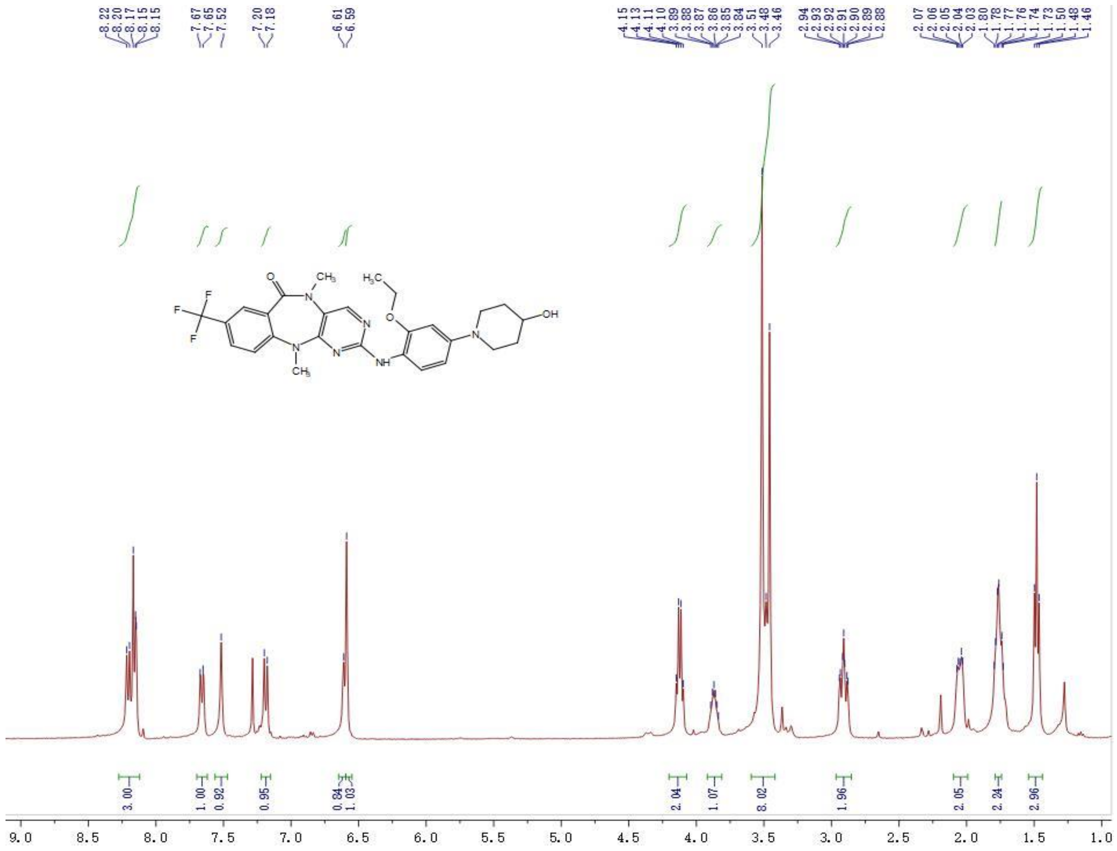

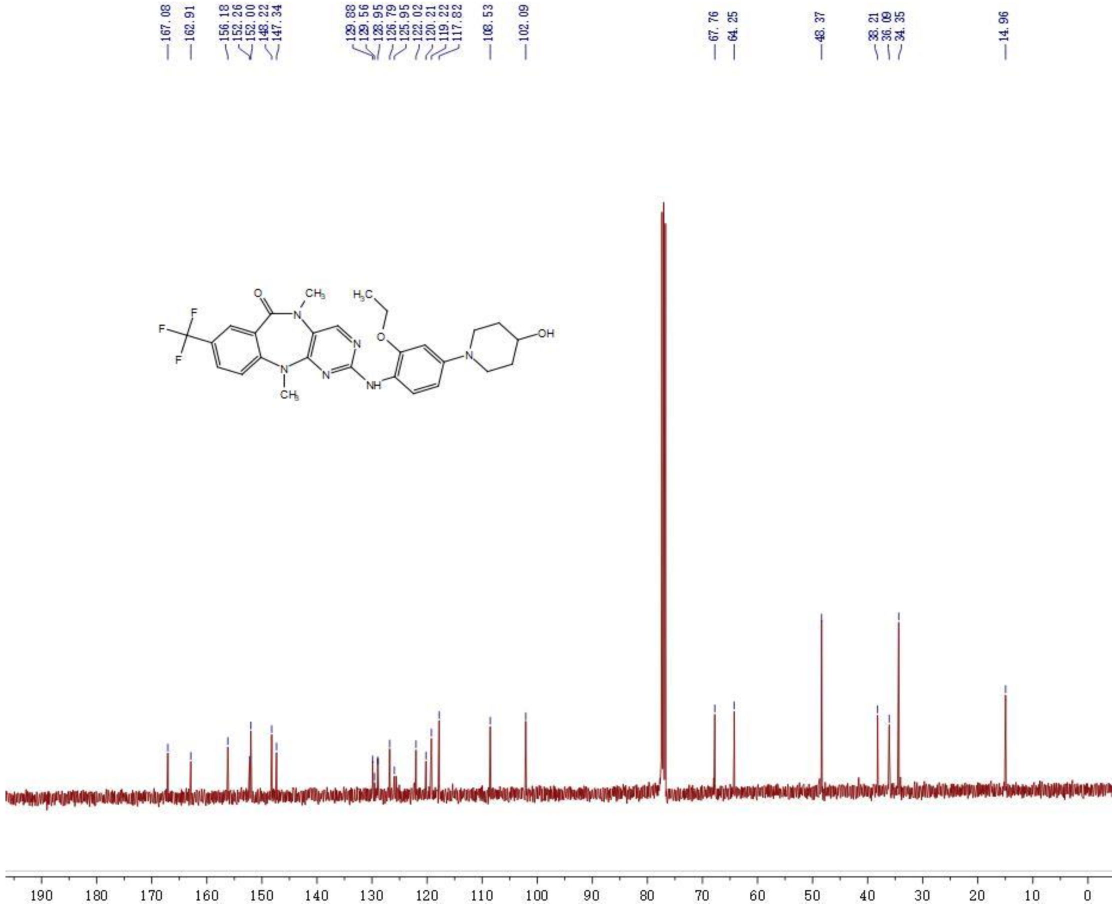

The $^{1}H/^{13}C$ NMR spectrum of Compound **A8**

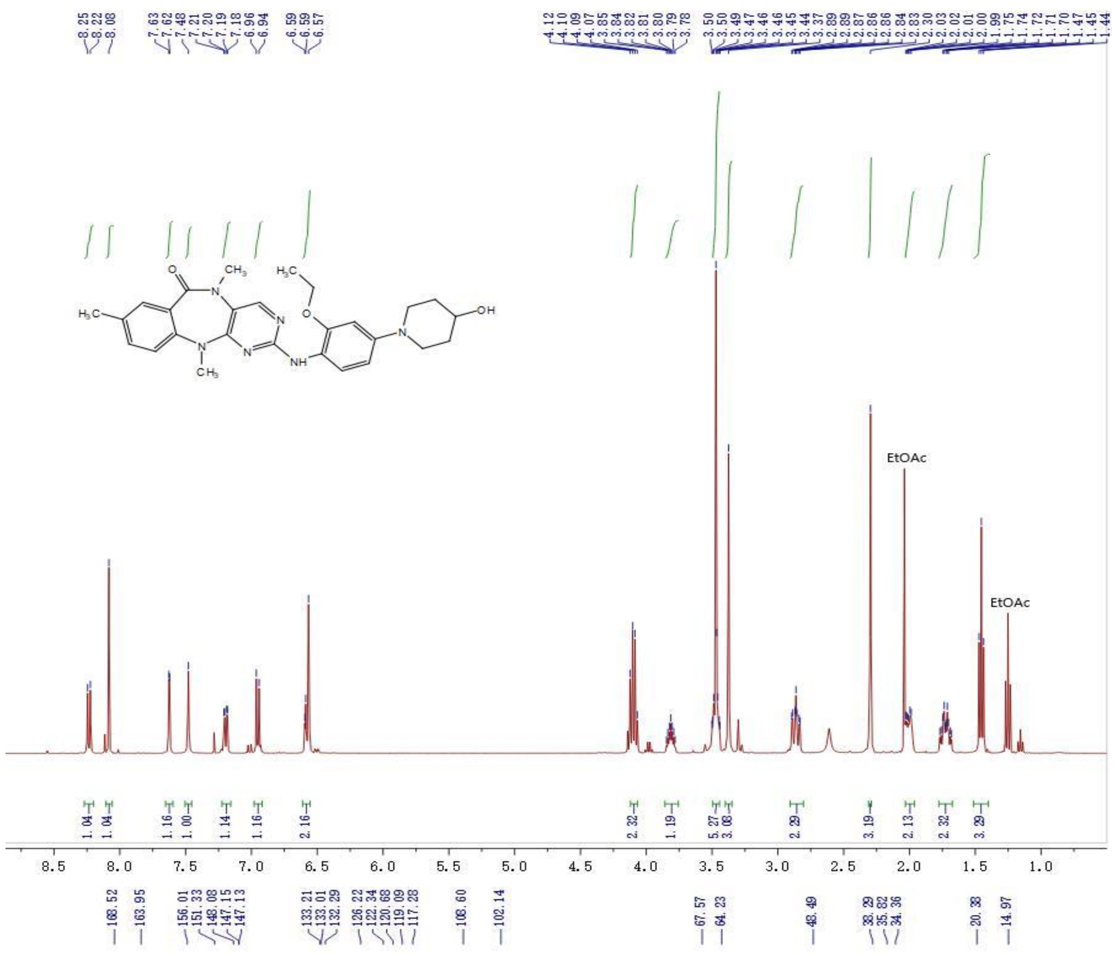

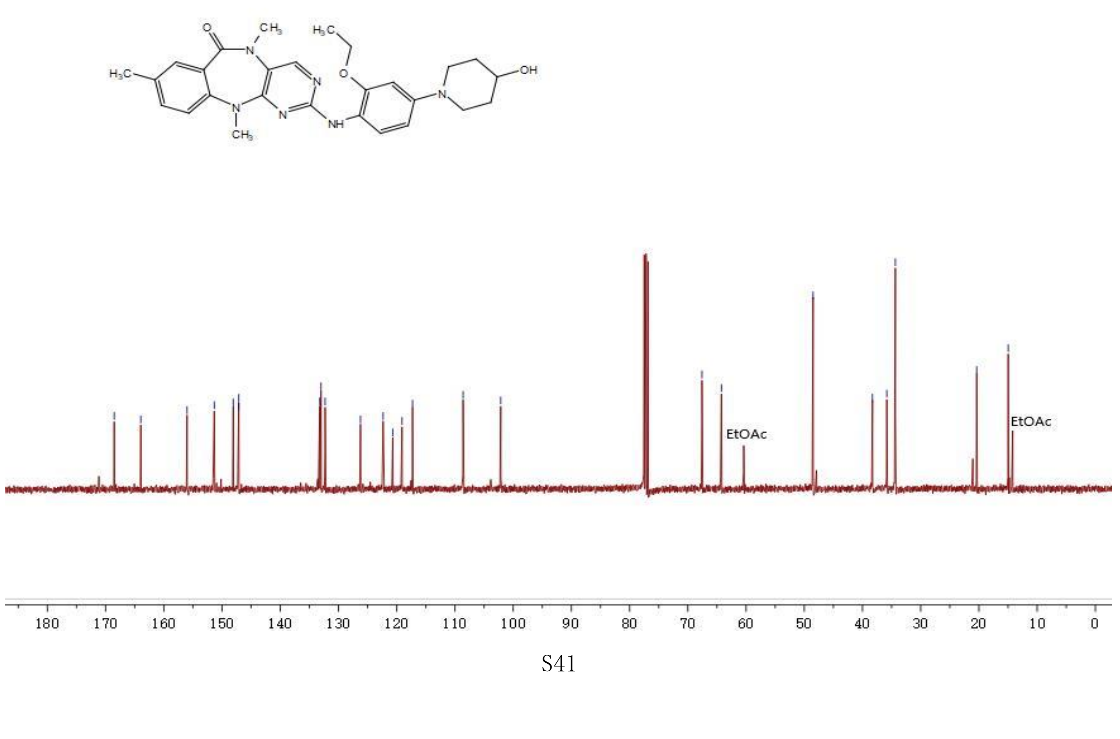

The $^{1}H/^{13}C$ NMR spectrum of Compound **A9**

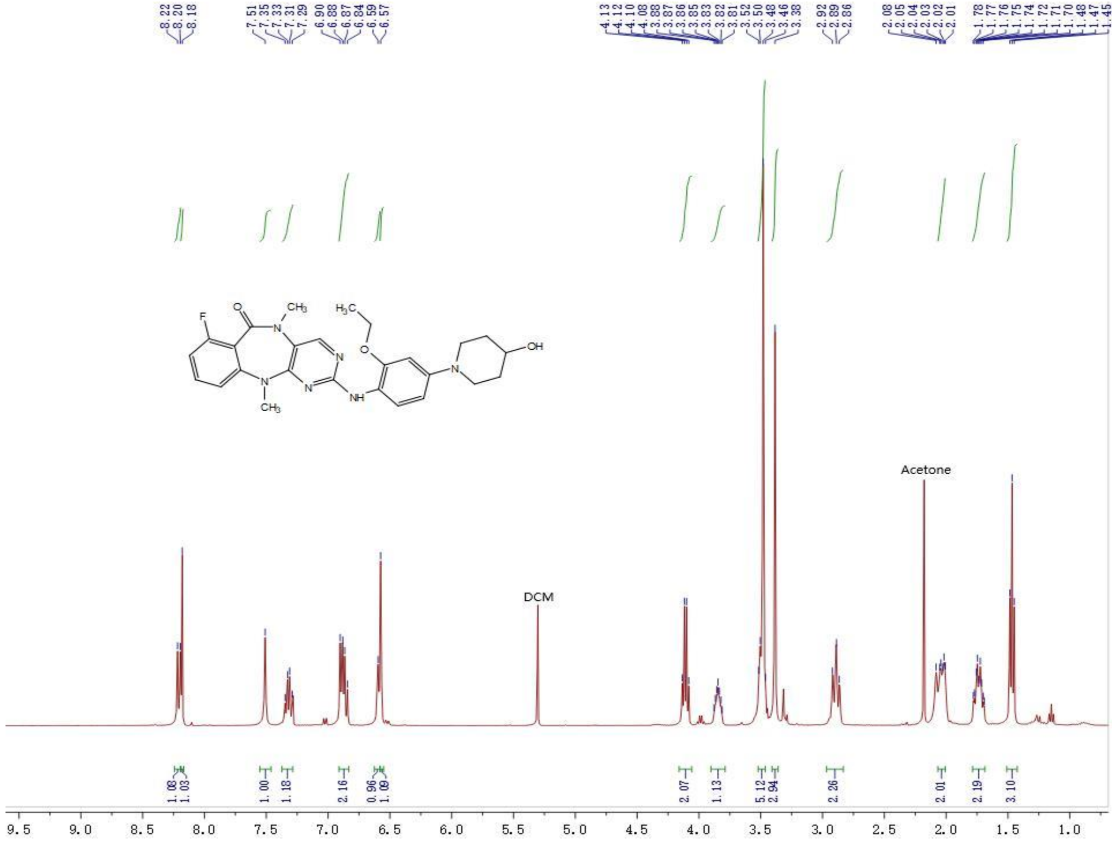

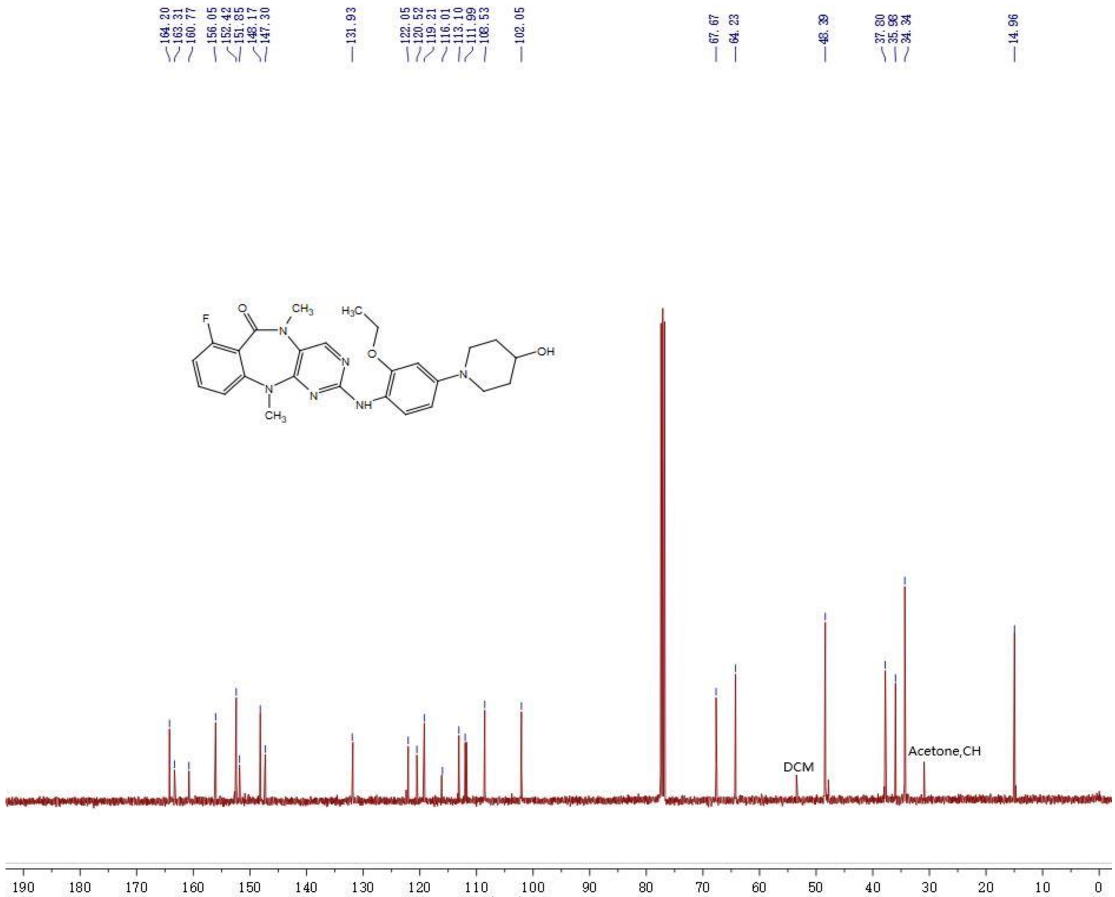

The $^{1}H/^{13}C$ NMR spectrum of Compound **A10**

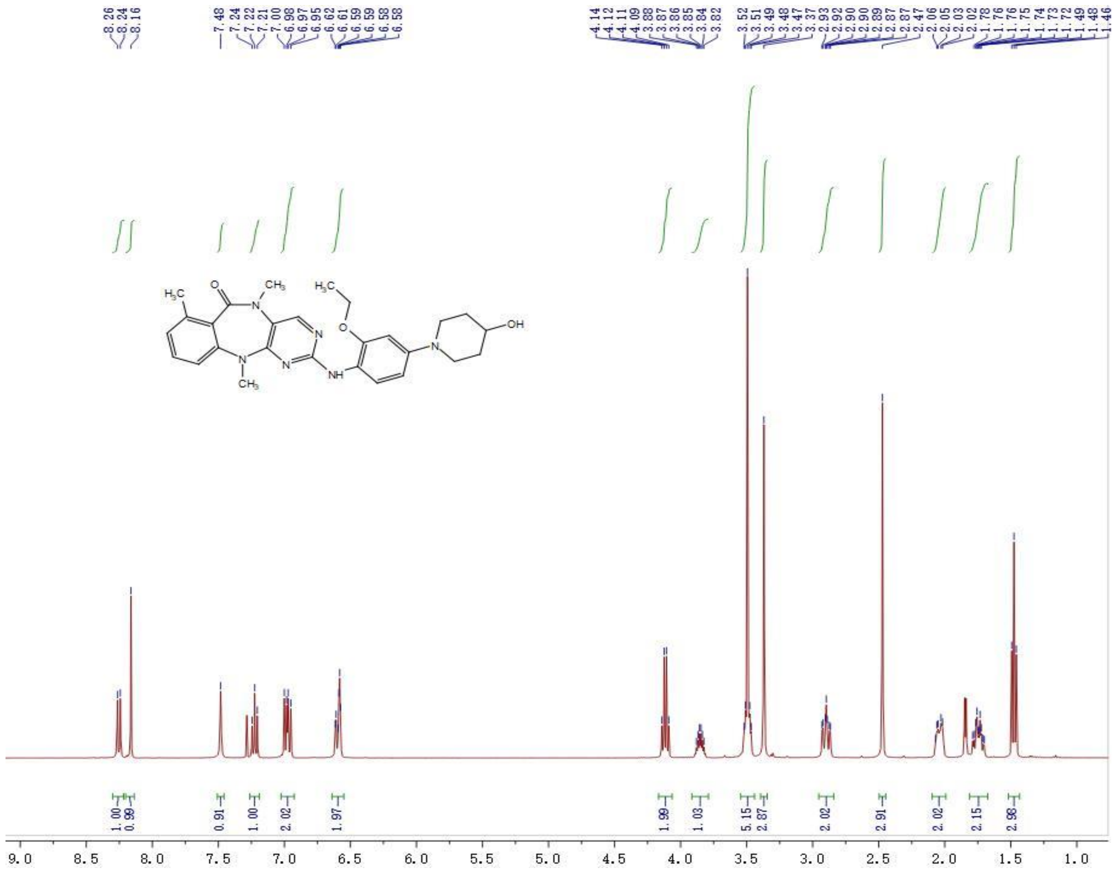

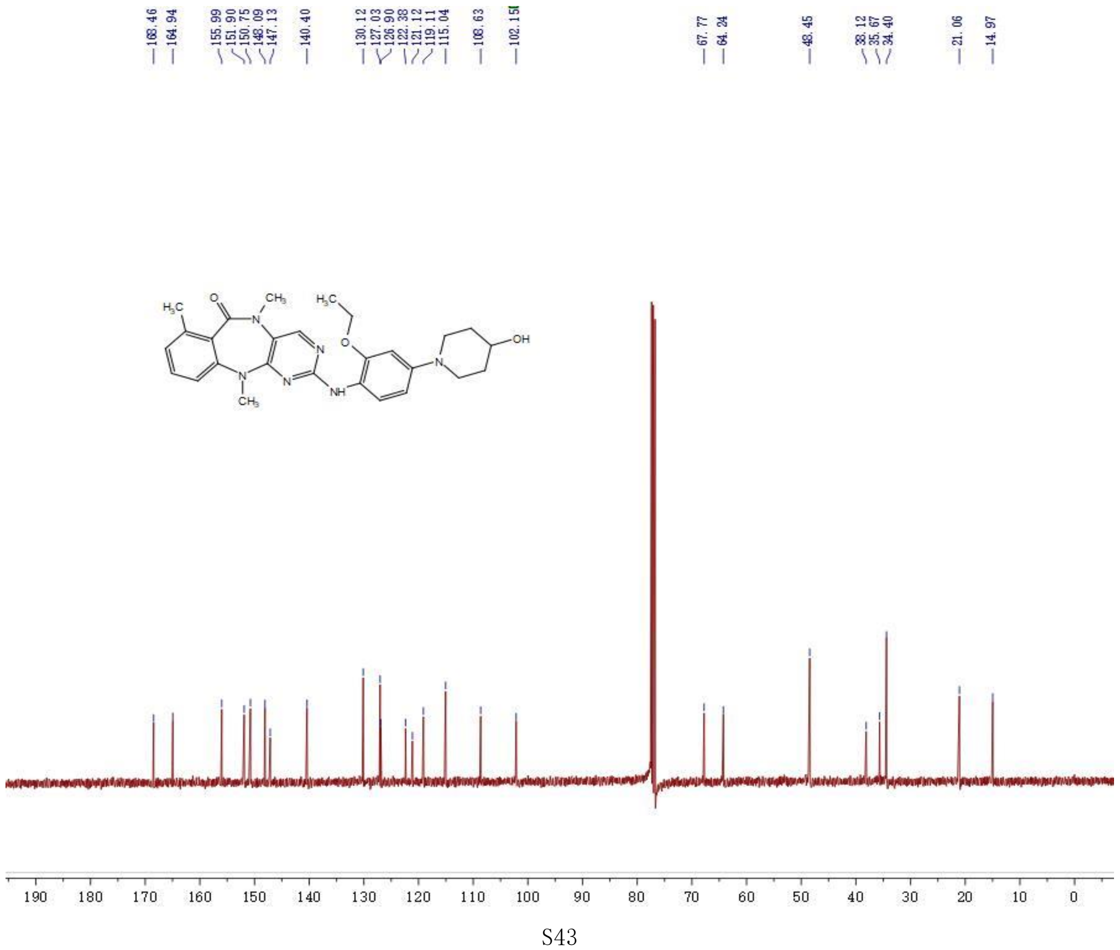

The $^1H/^{13}C$ NMR spectrum of Compound **B1**

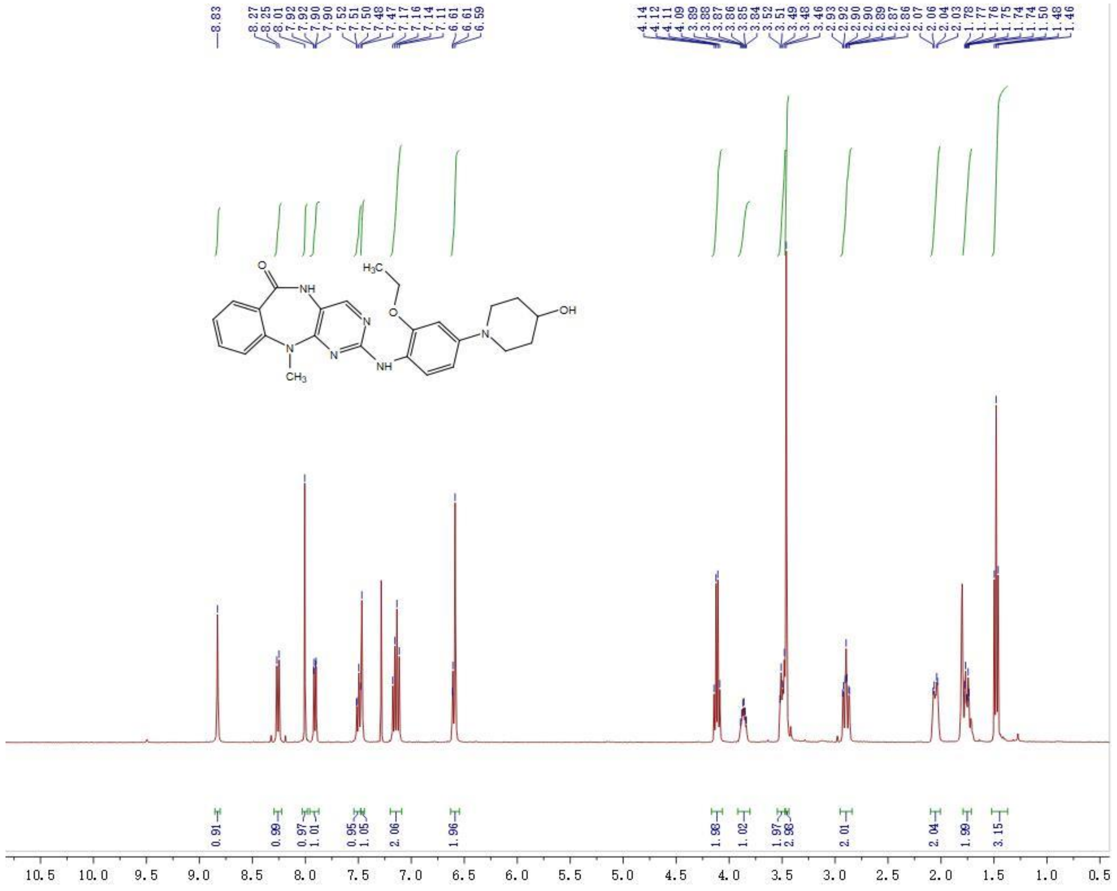

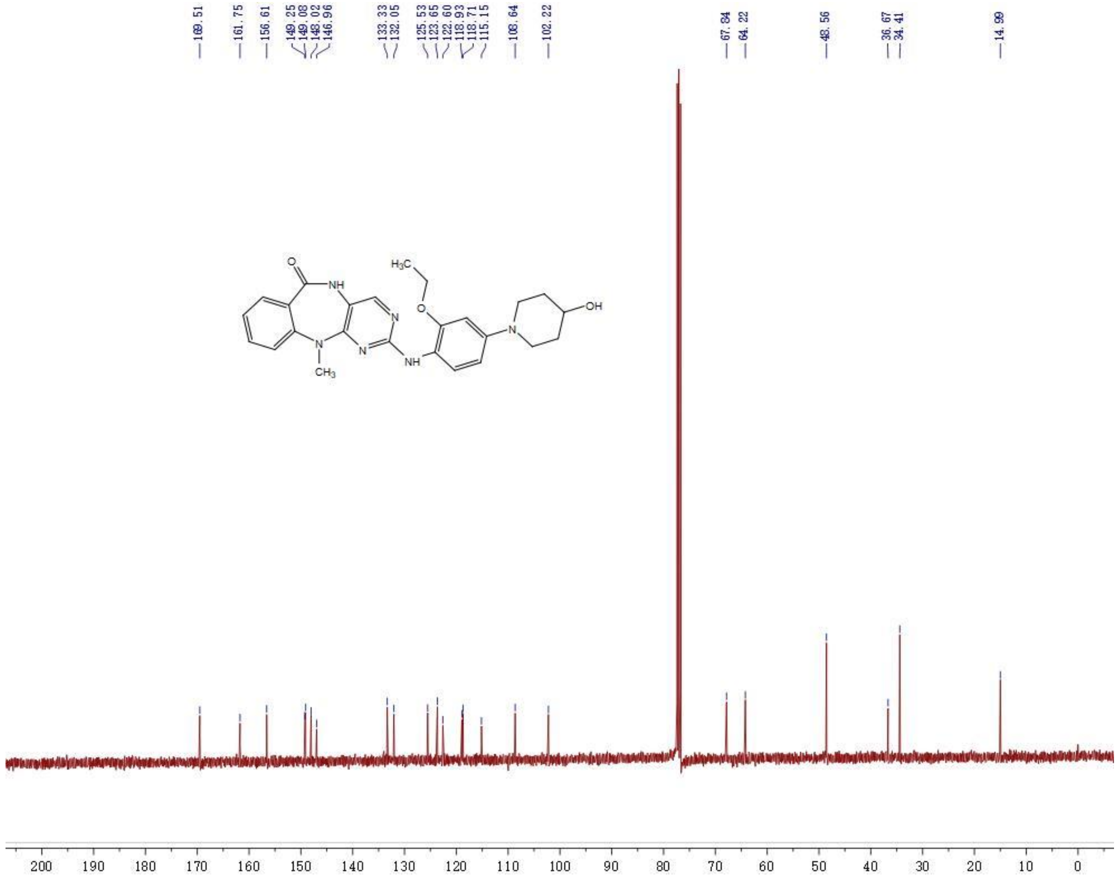

The ^{1}H/^{13}C NMR spectrum of Compound **B2**

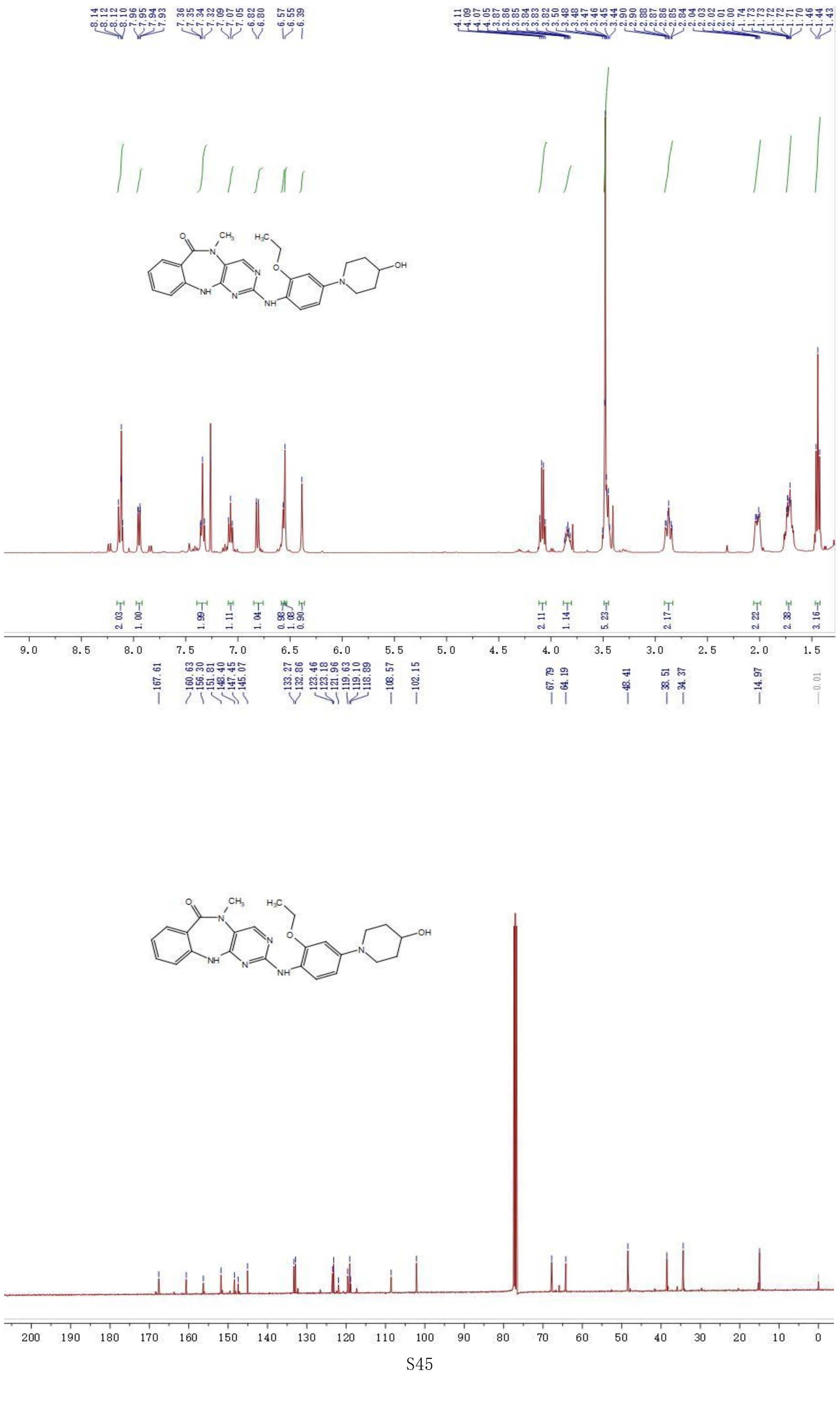

The $^{1}H/^{13}C$ NMR spectrum of Compound **B3**

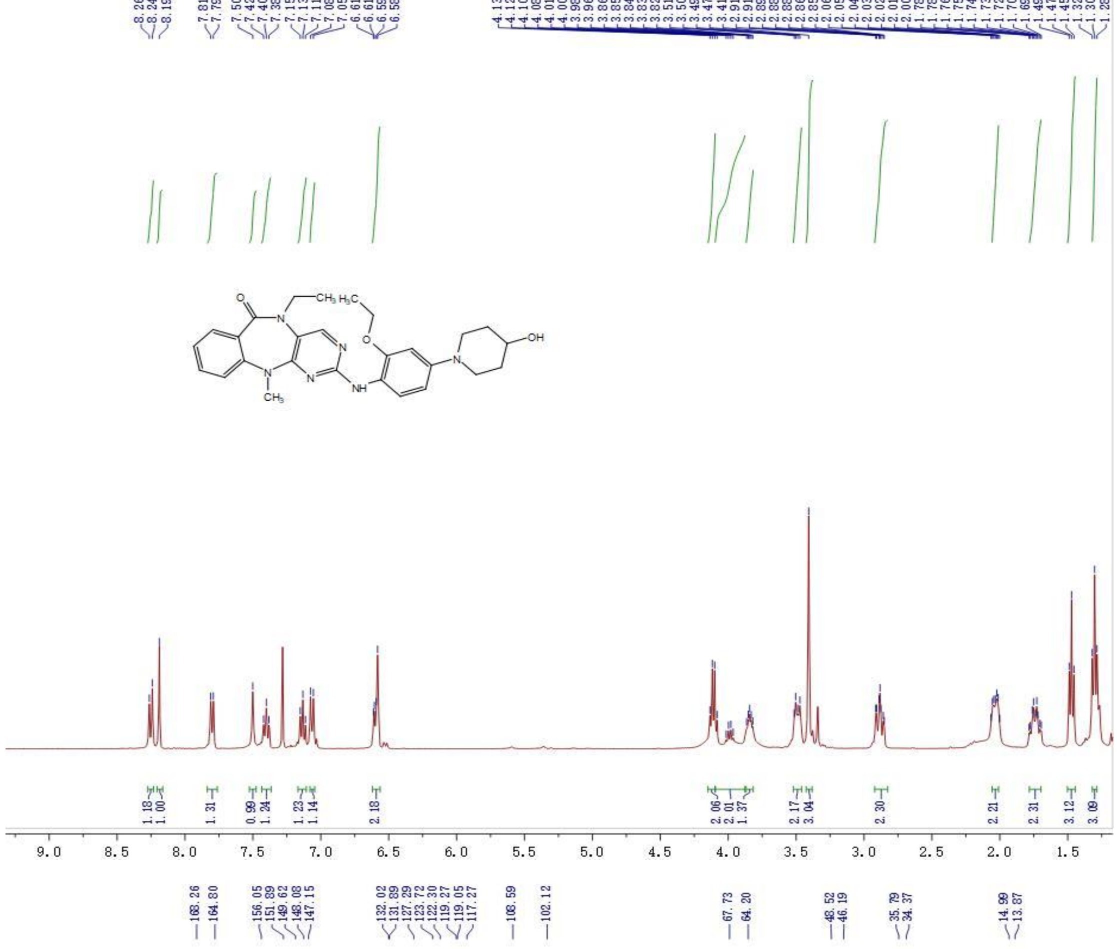

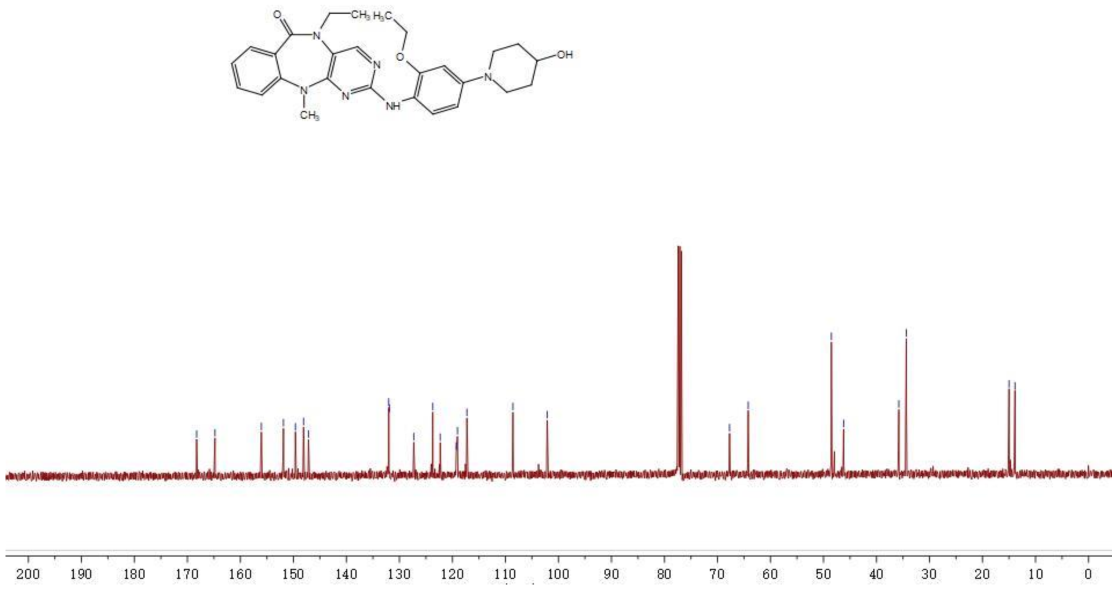

The $^{1}H/^{13}C$ NMR spectrum of Compound **C1**

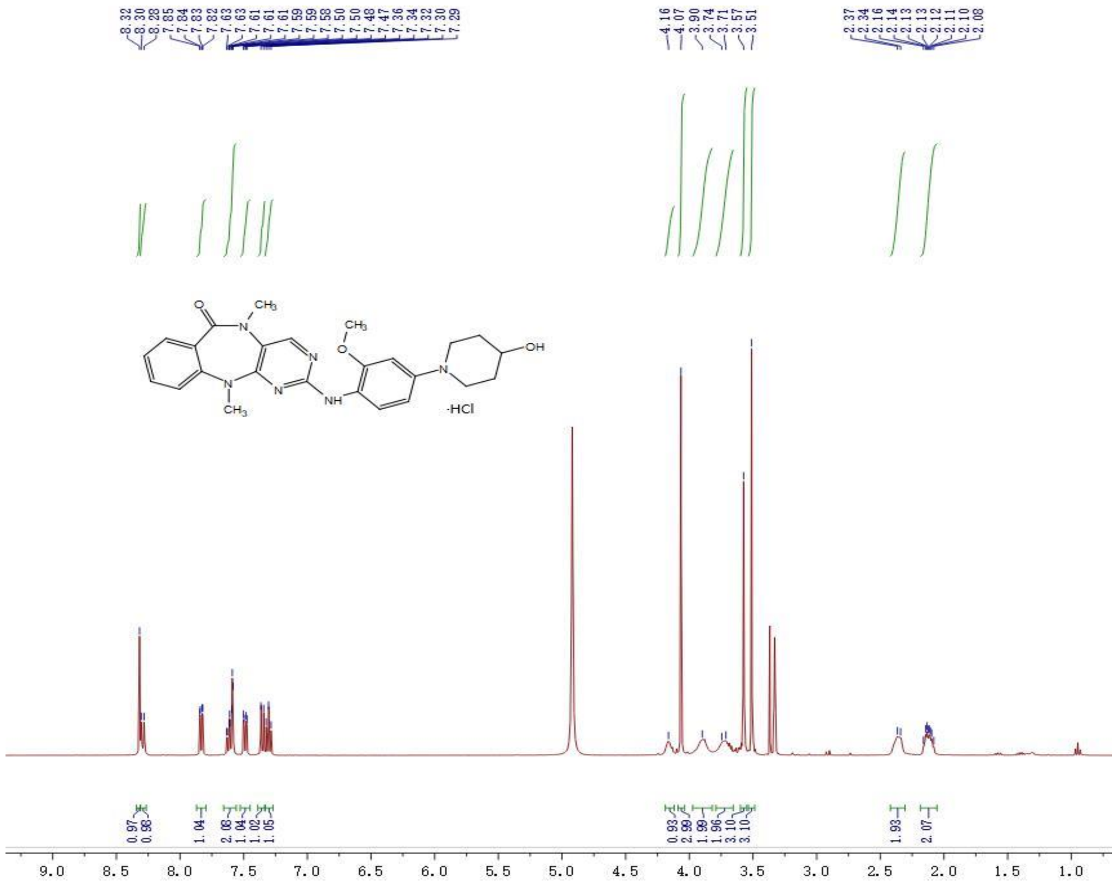

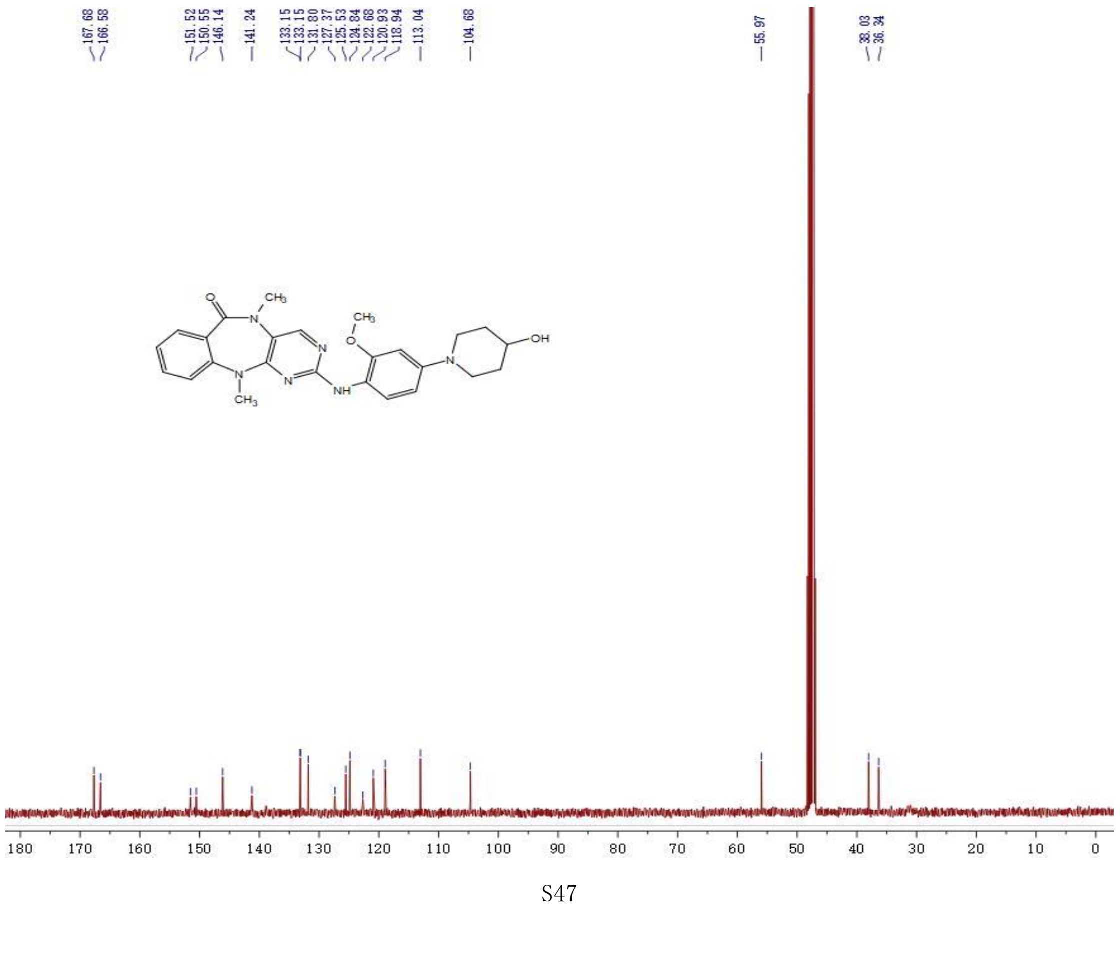

The $^{1}H/^{13}C$ NMR spectrum of Compound **C2**

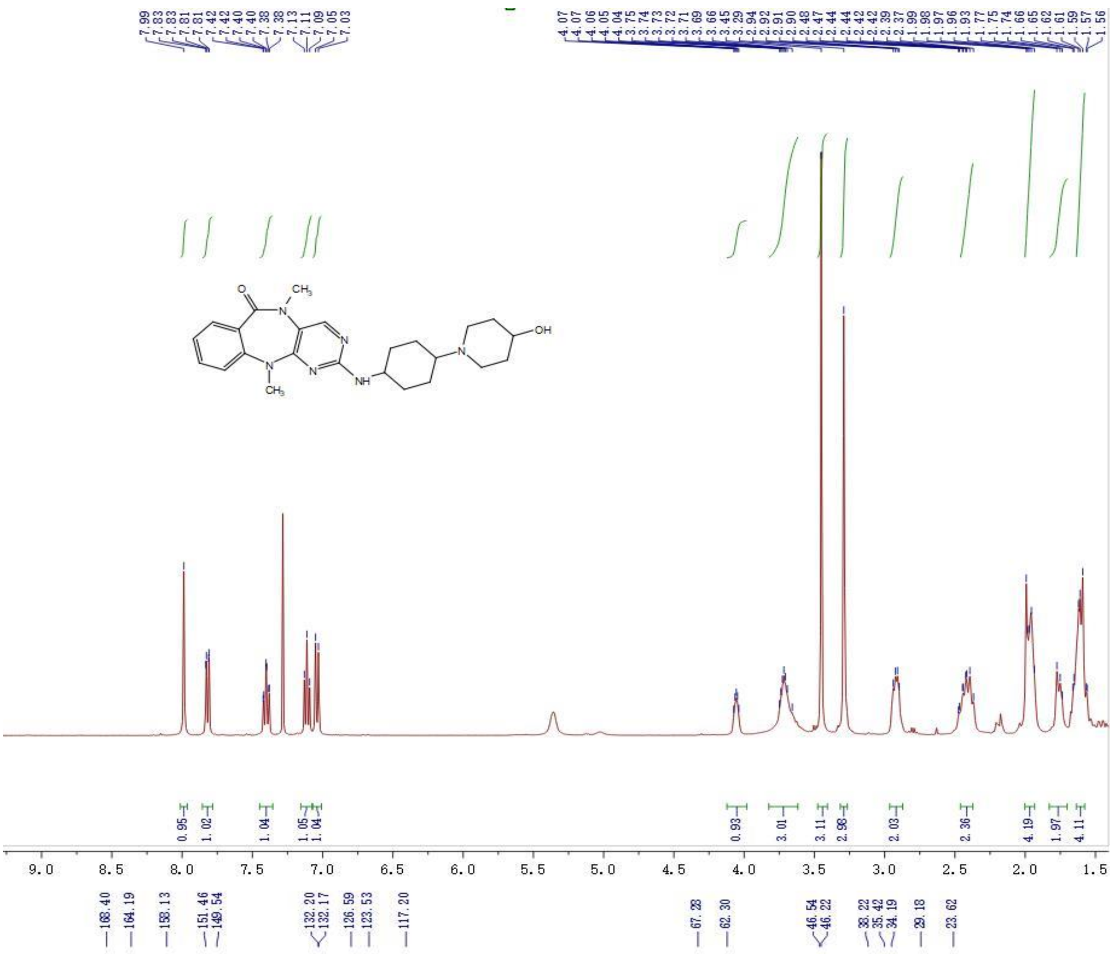

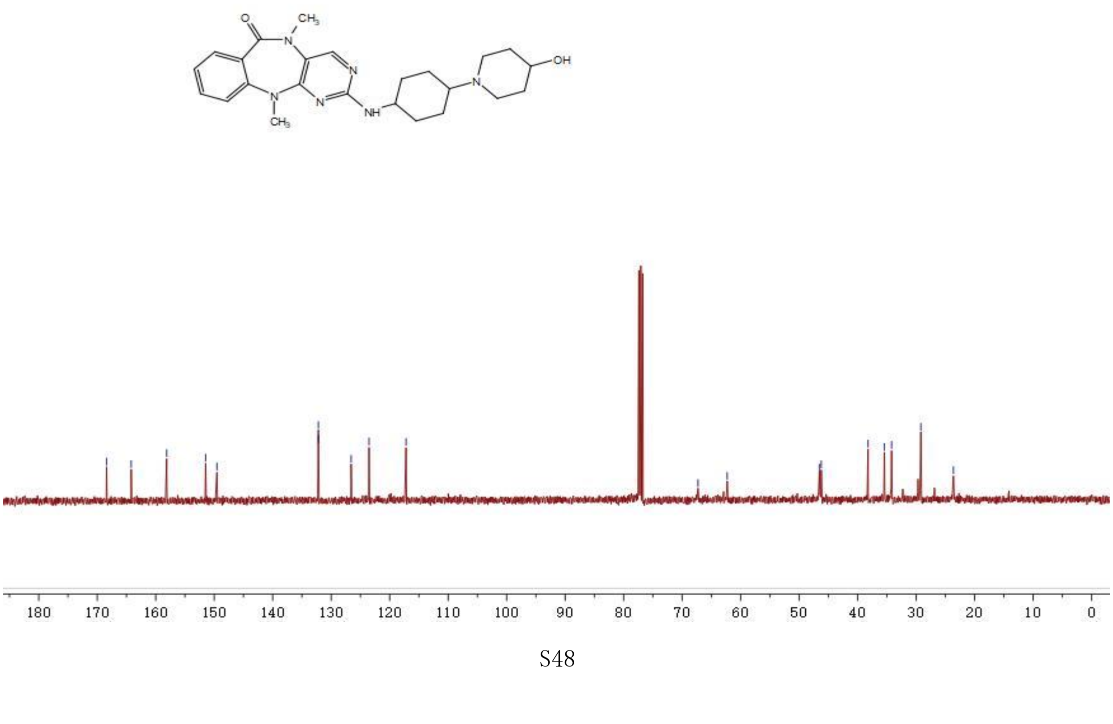

The $^1H/^{13}C$ NMR spectrum of Compound **C3**

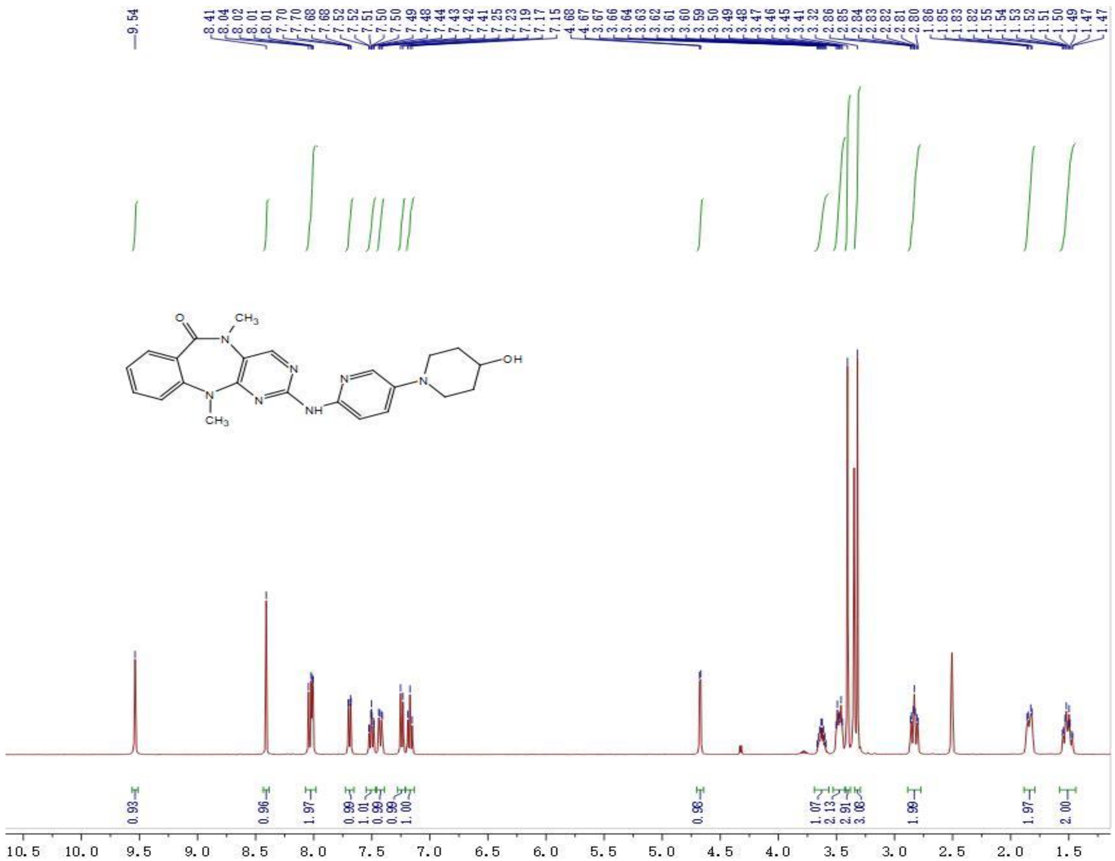

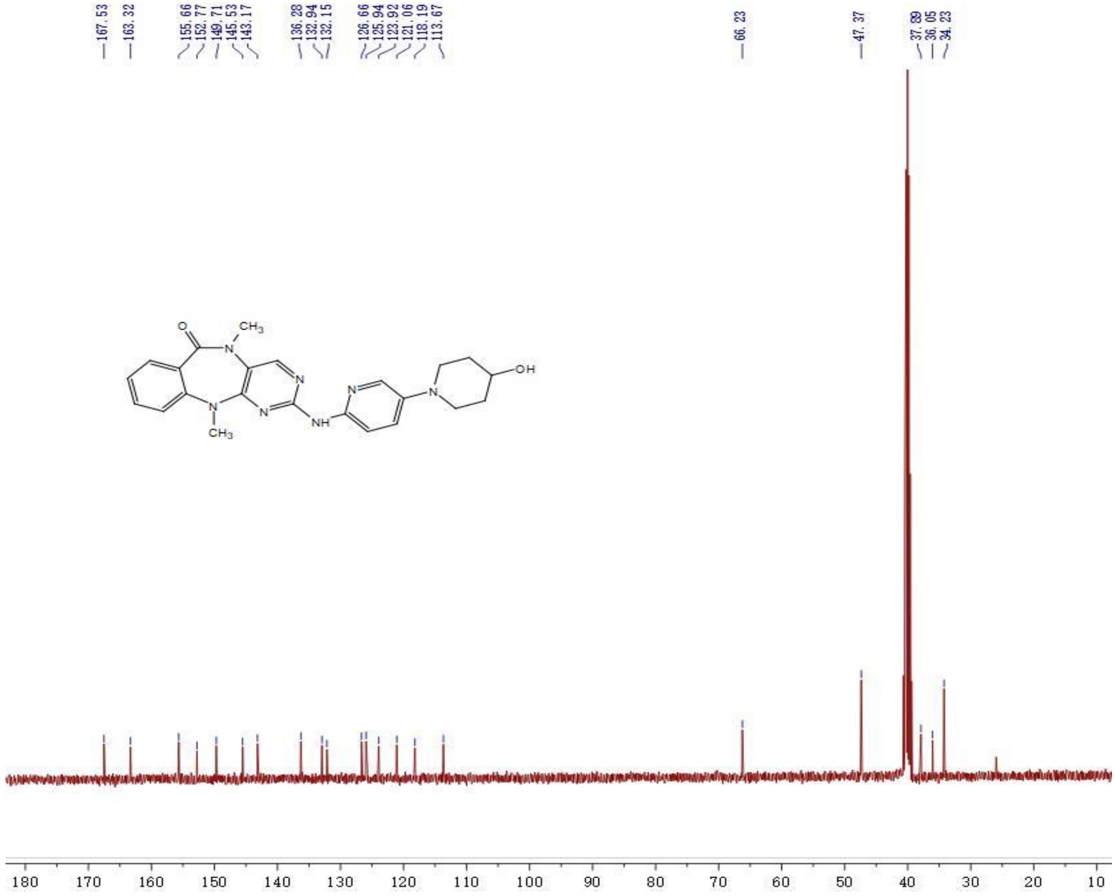

The $^{1}H/^{13}C$ NMR spectrum of Compound **C4**

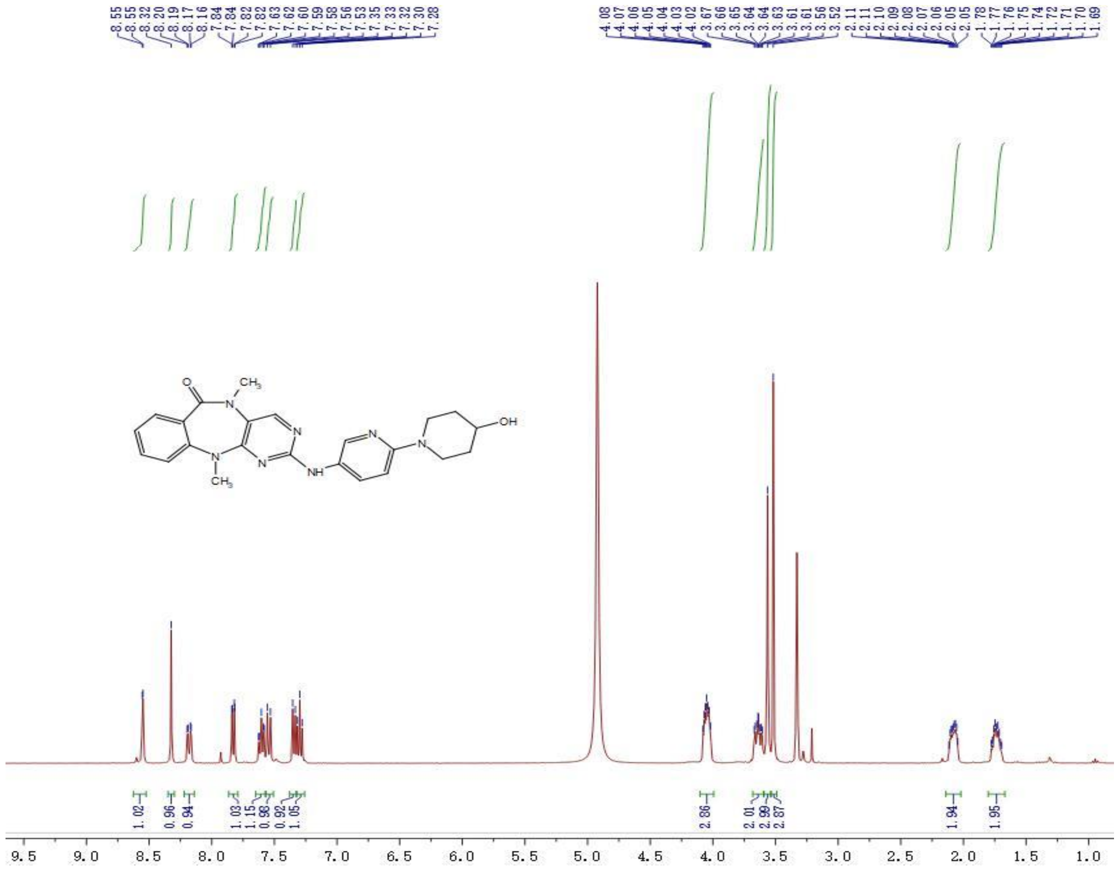

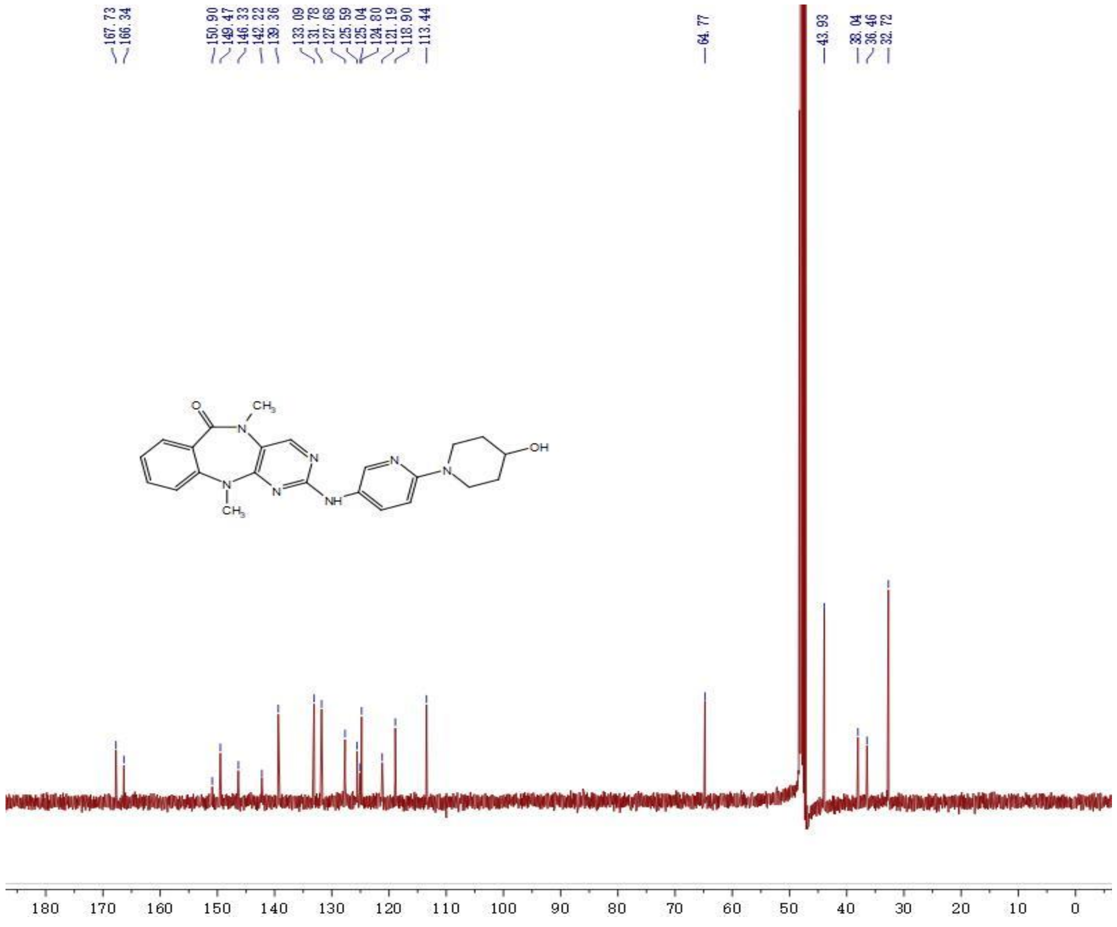

The $^{1}H/^{13}C$ NMR spectrum of intermediate **14**

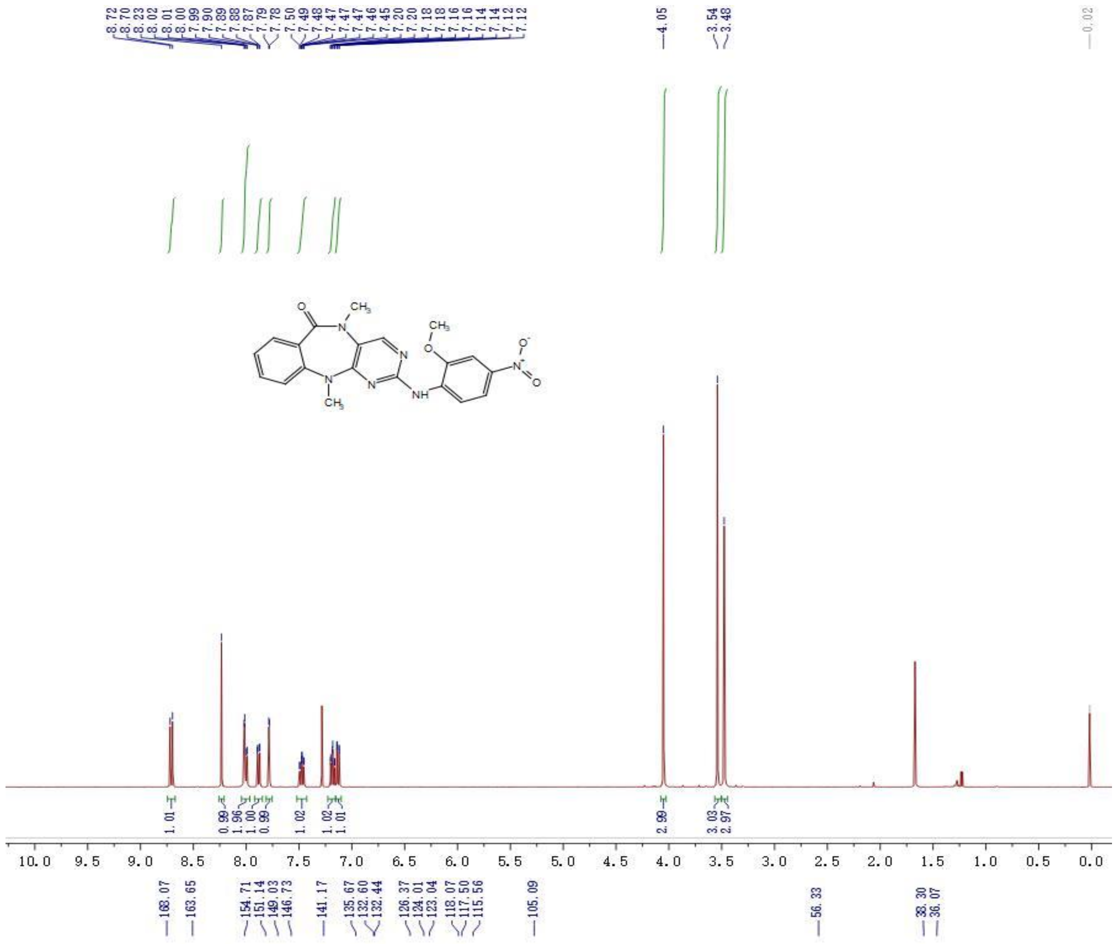

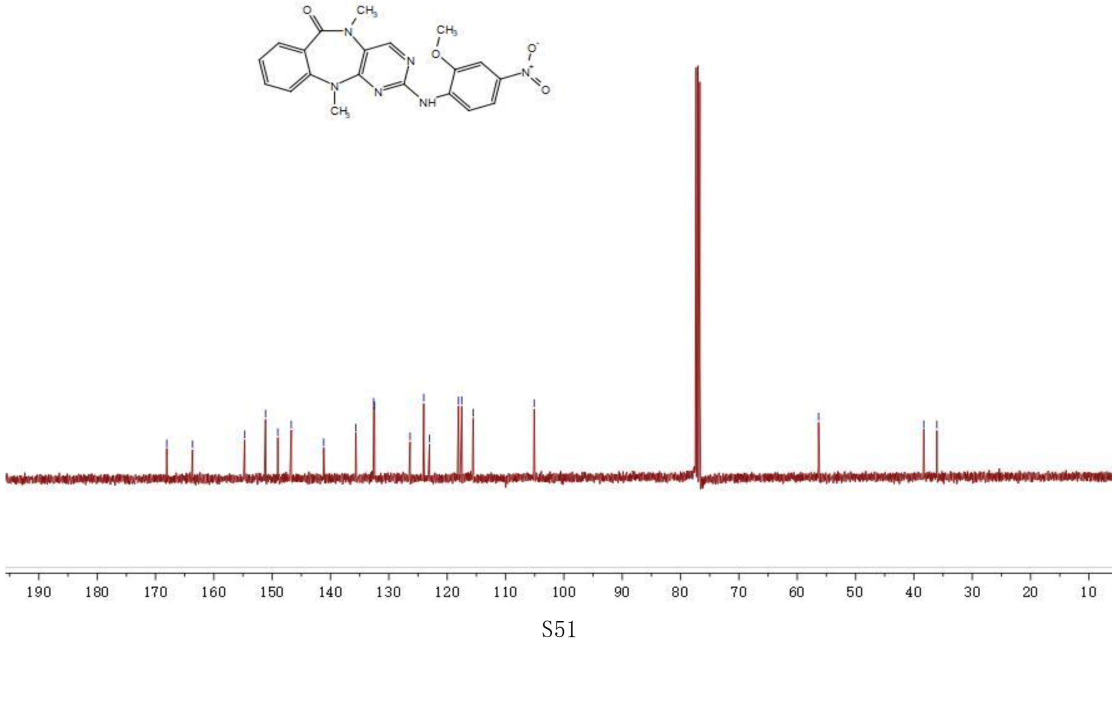

The $^{1}H/^{13}C$ NMR spectrum of intermediate **15**

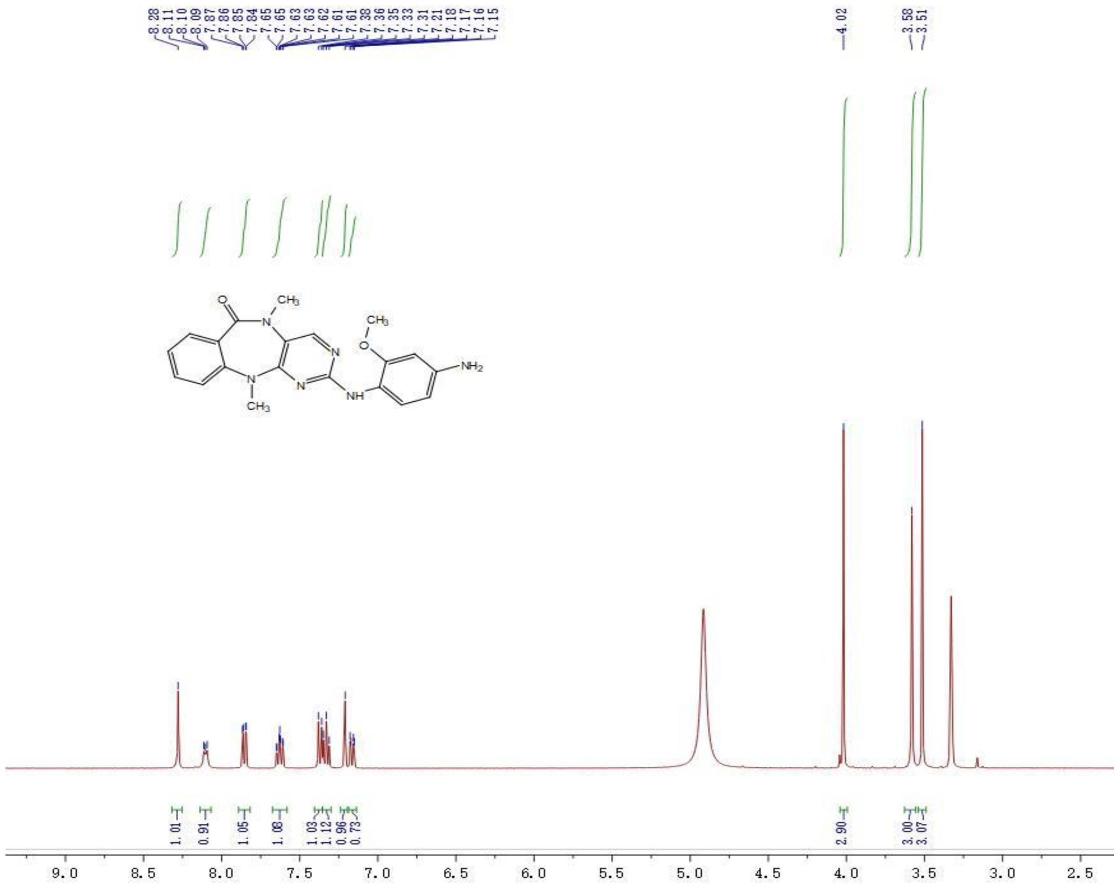

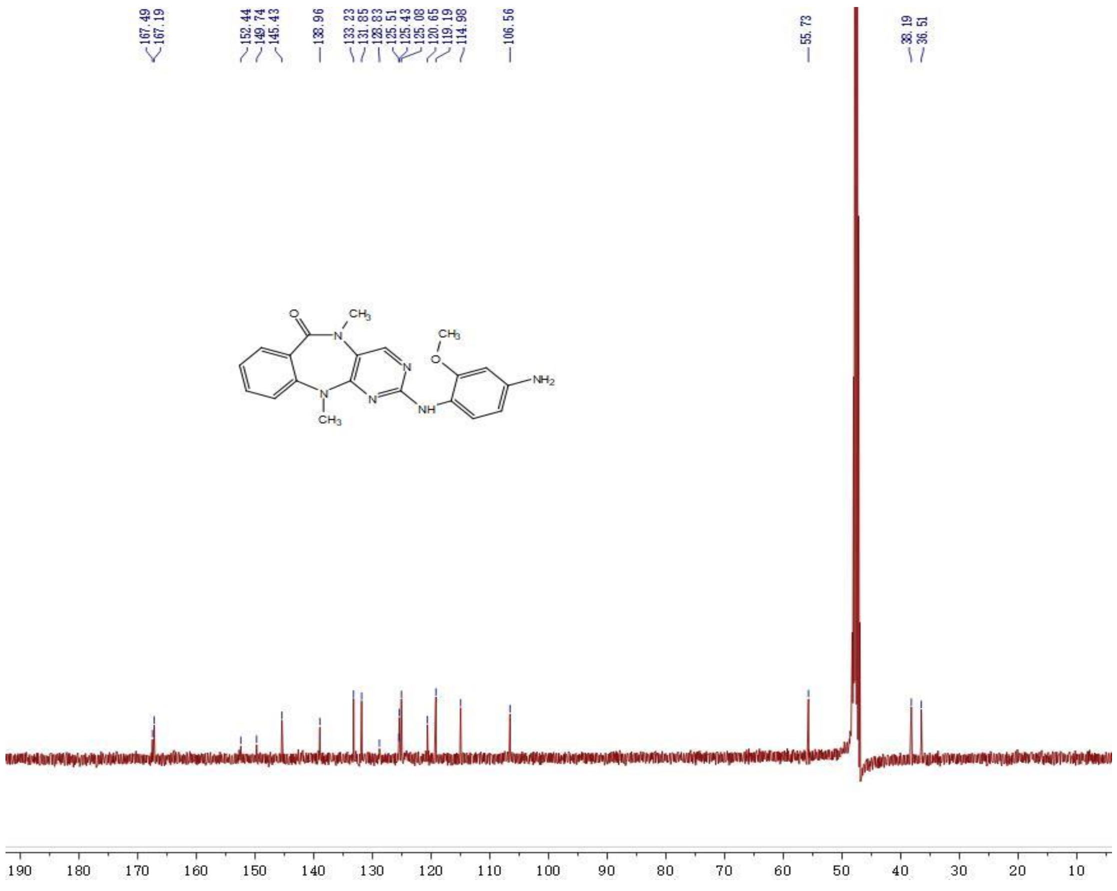

The $^{1}H/^{13}C$ NMR spectrum of Compound **D1**

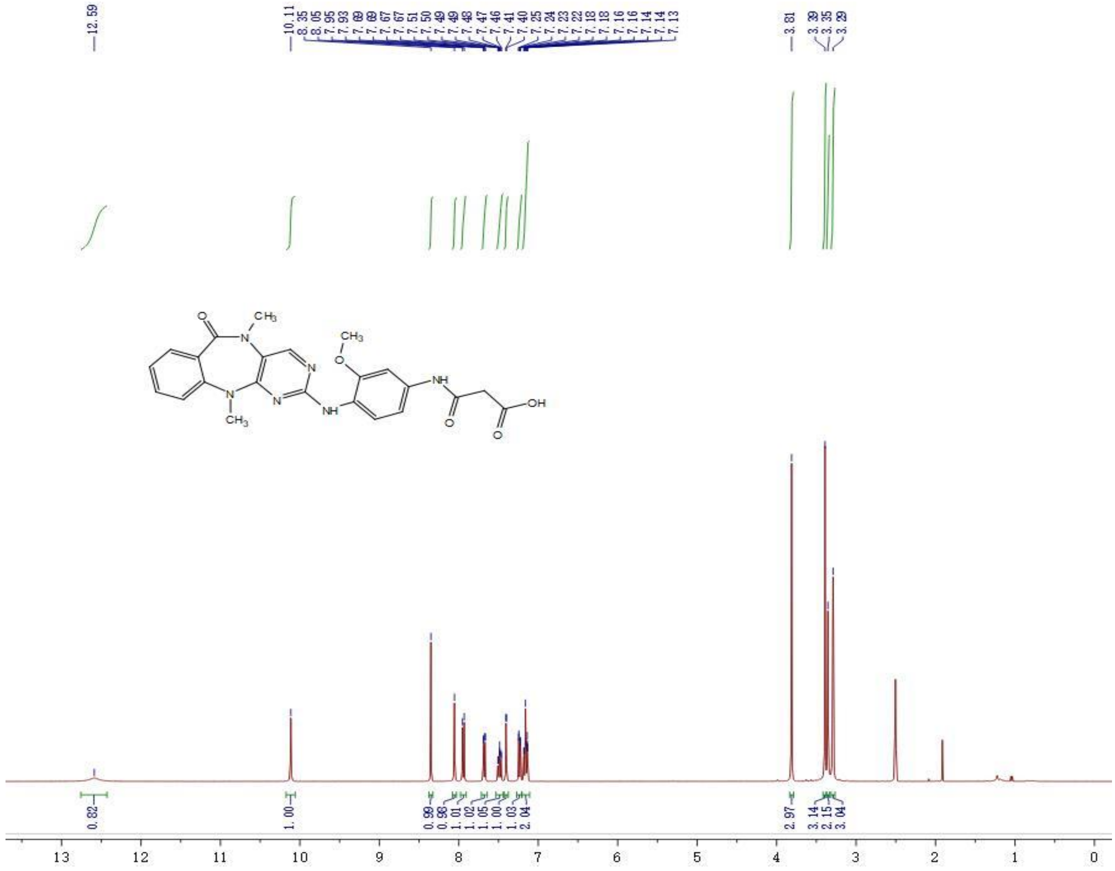

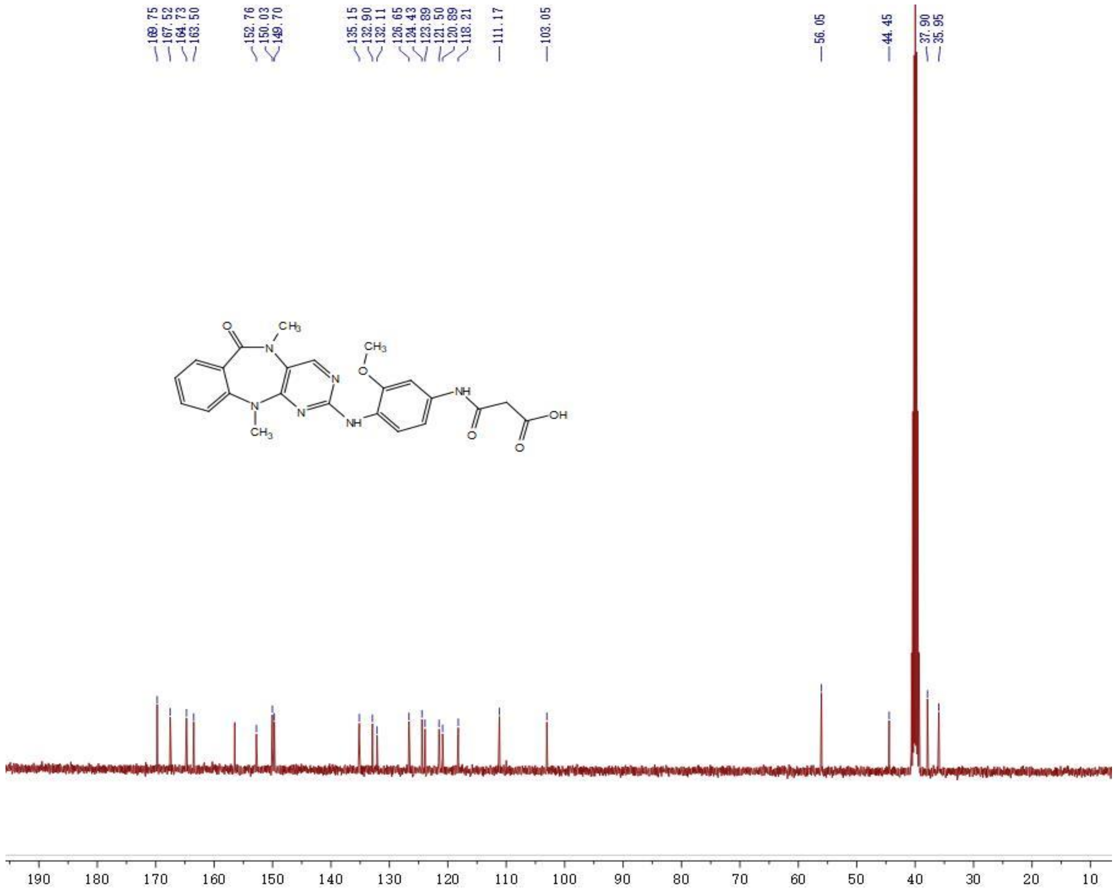

The $^{1}H/^{13}C$ NMR spectrum of Compound **D2**

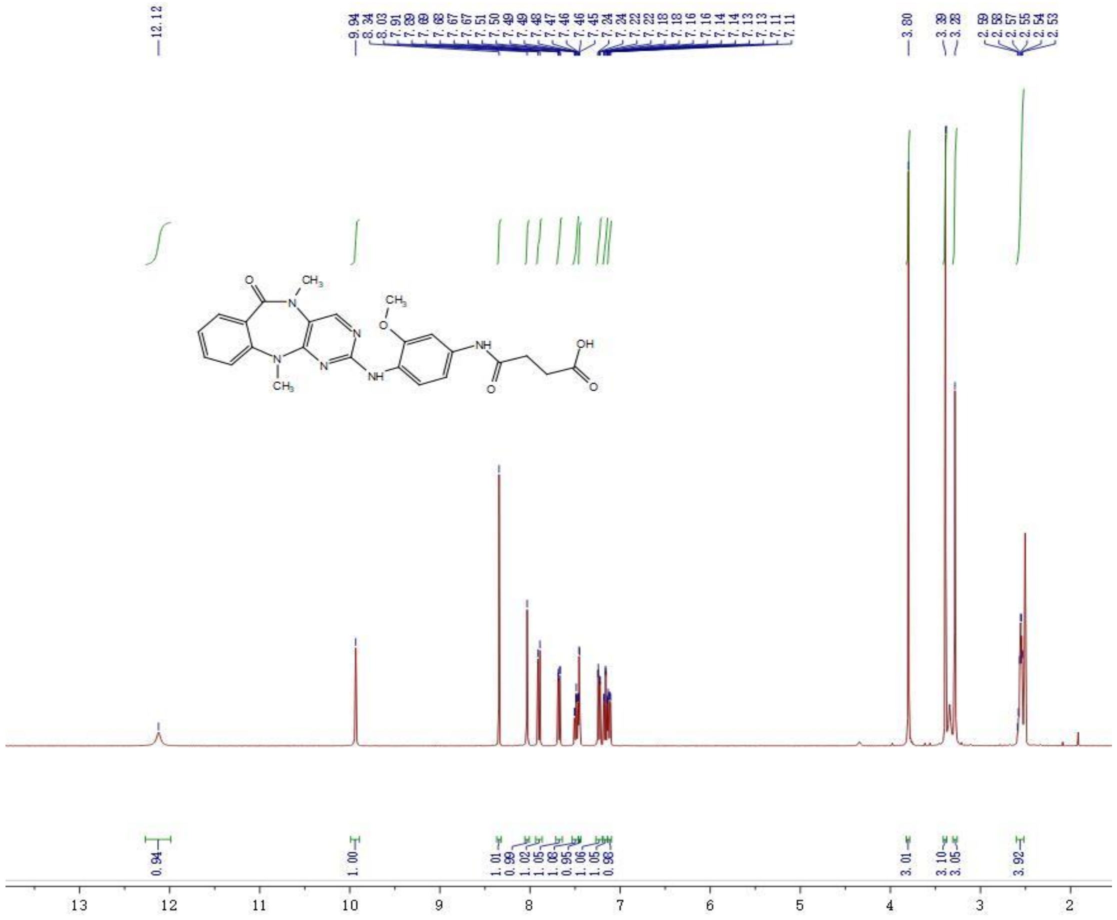

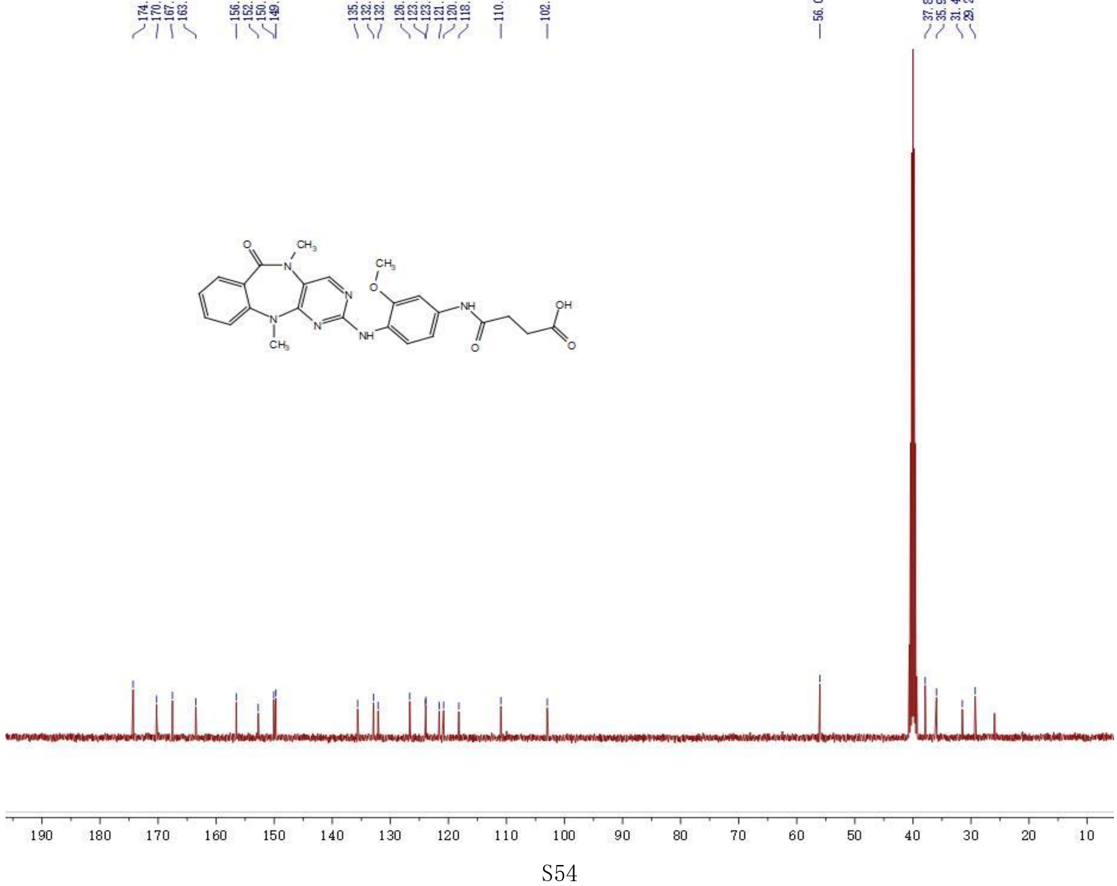

The $^{1}H/^{13}C$ NMR spectrum of intermediate **16**

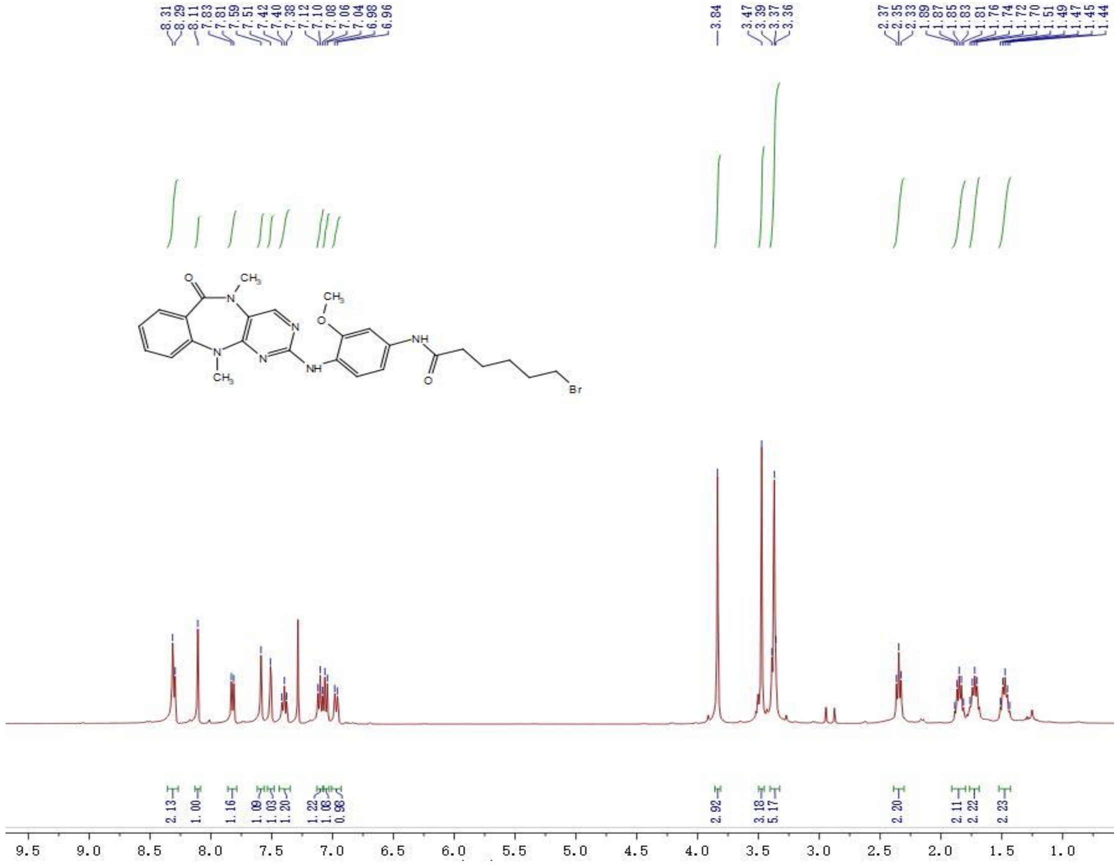

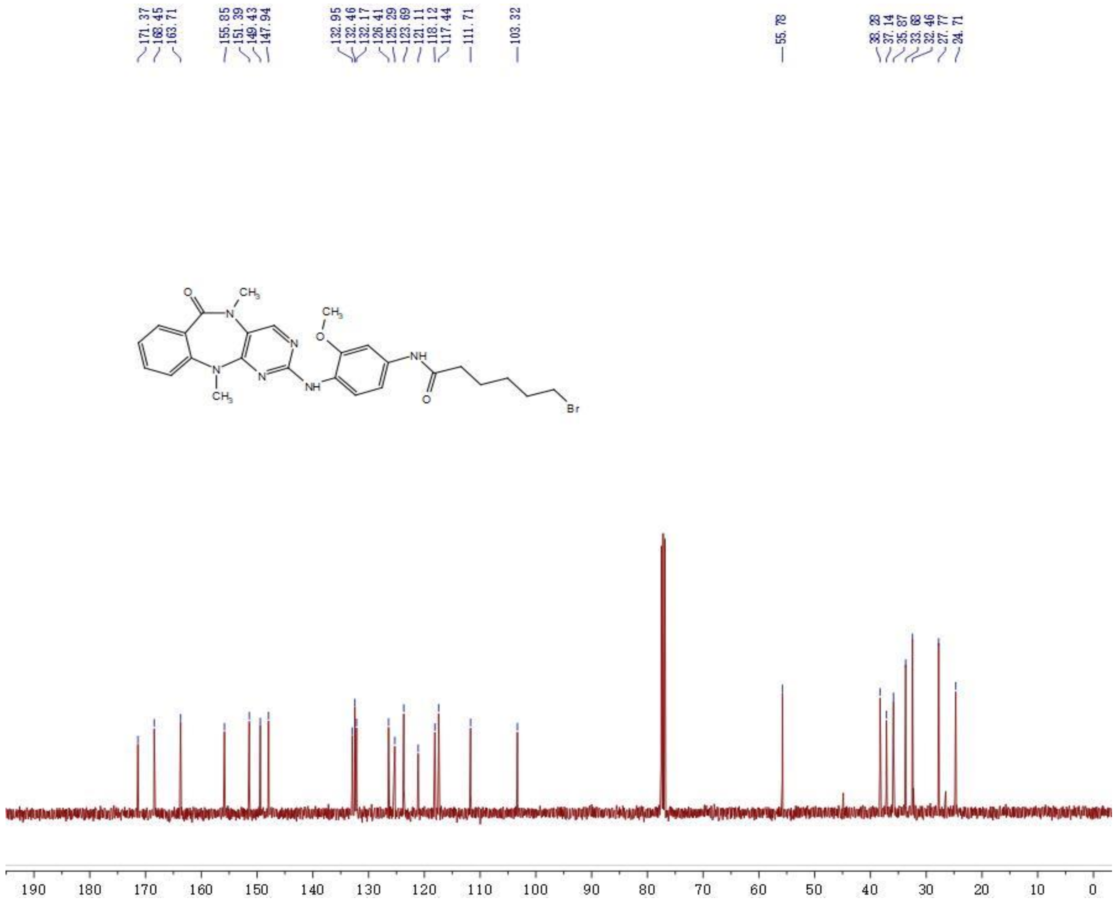

The $^{1}H/^{13}C$ NMR spectrum of Compound **D8**

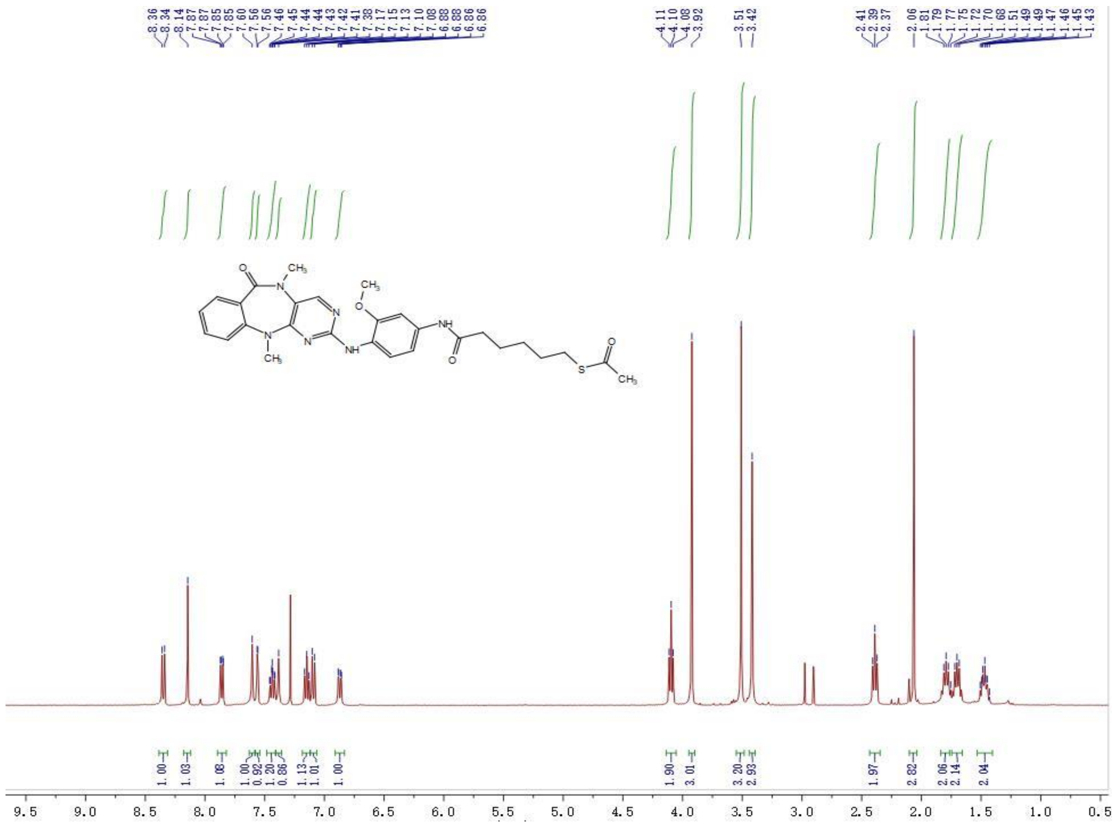

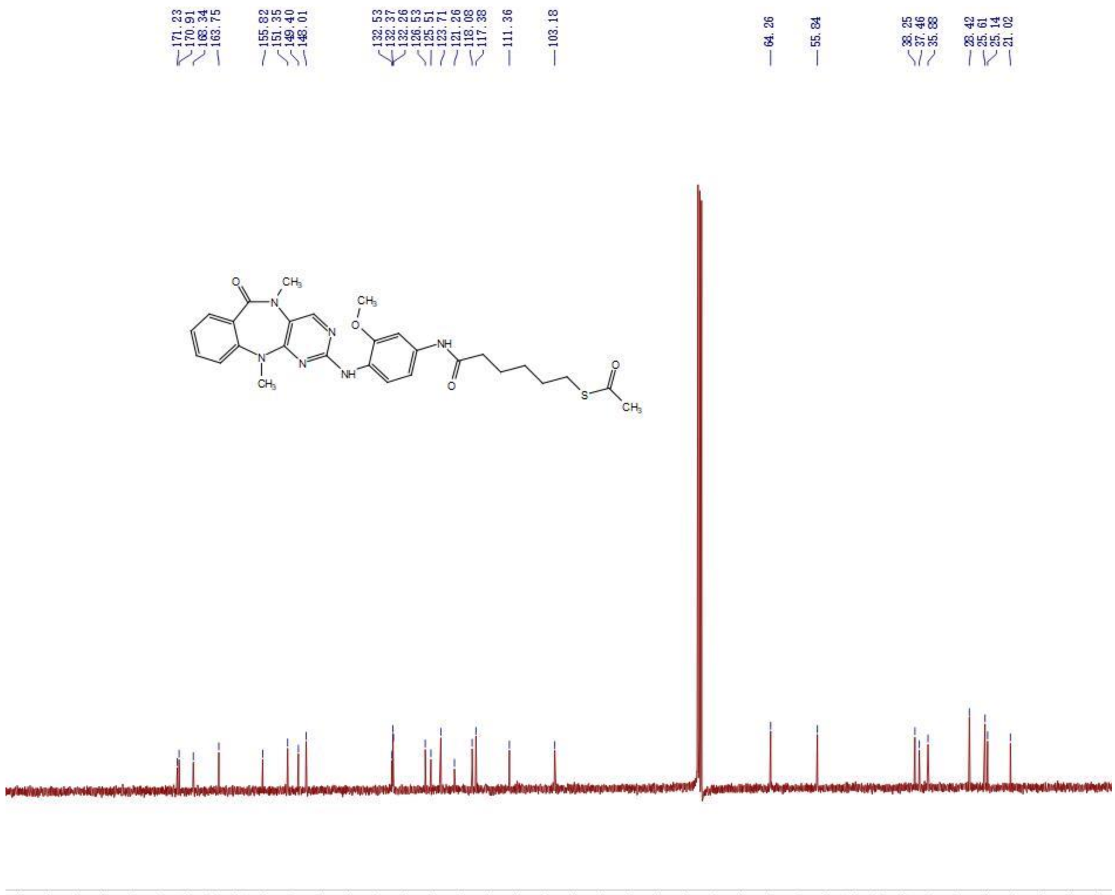

The $^{1}H/^{13}C$ NMR spectrum of intermediate **18-4**

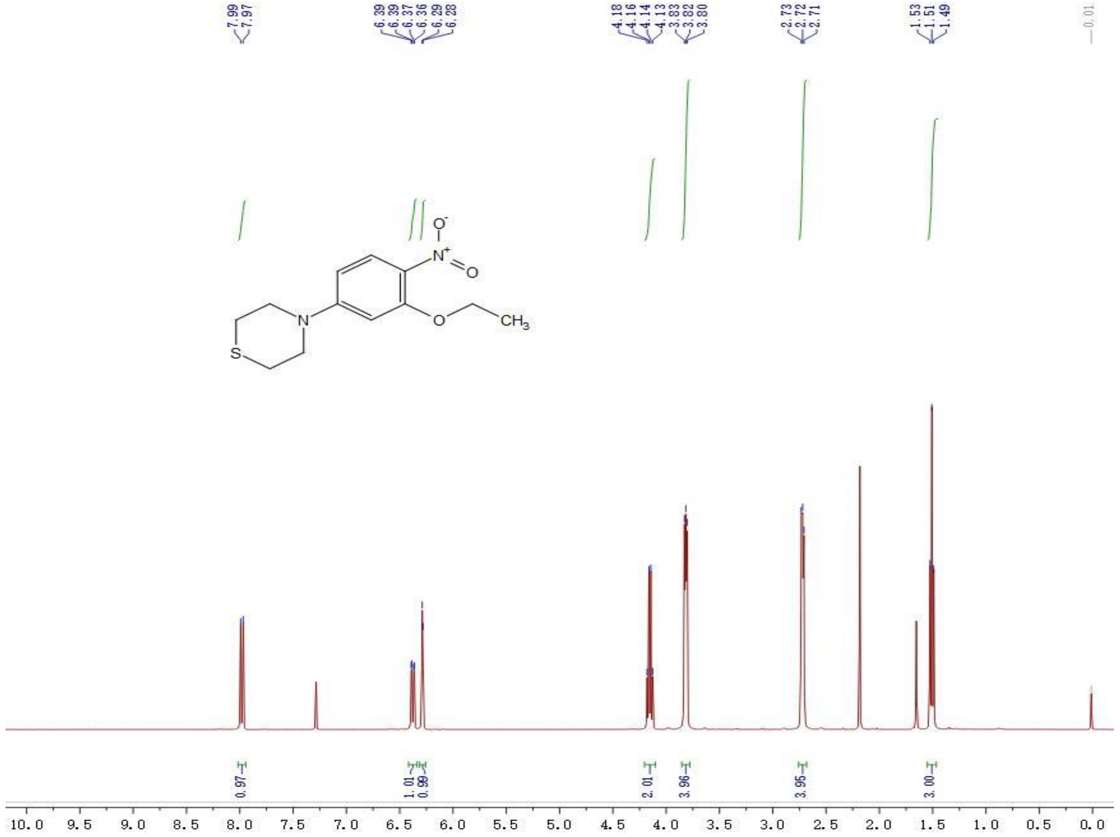

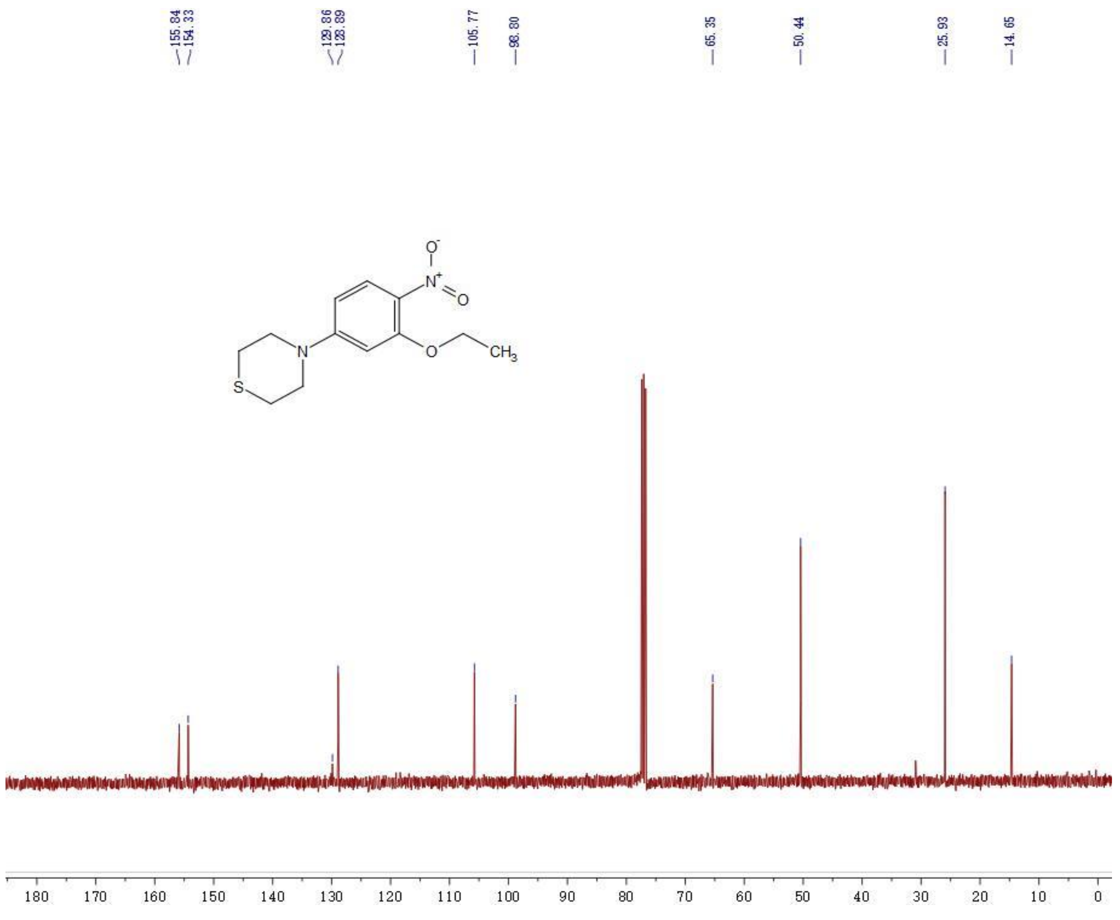

The $^{1}H/^{13}C$ NMR spectrum of intermediate **19-3**

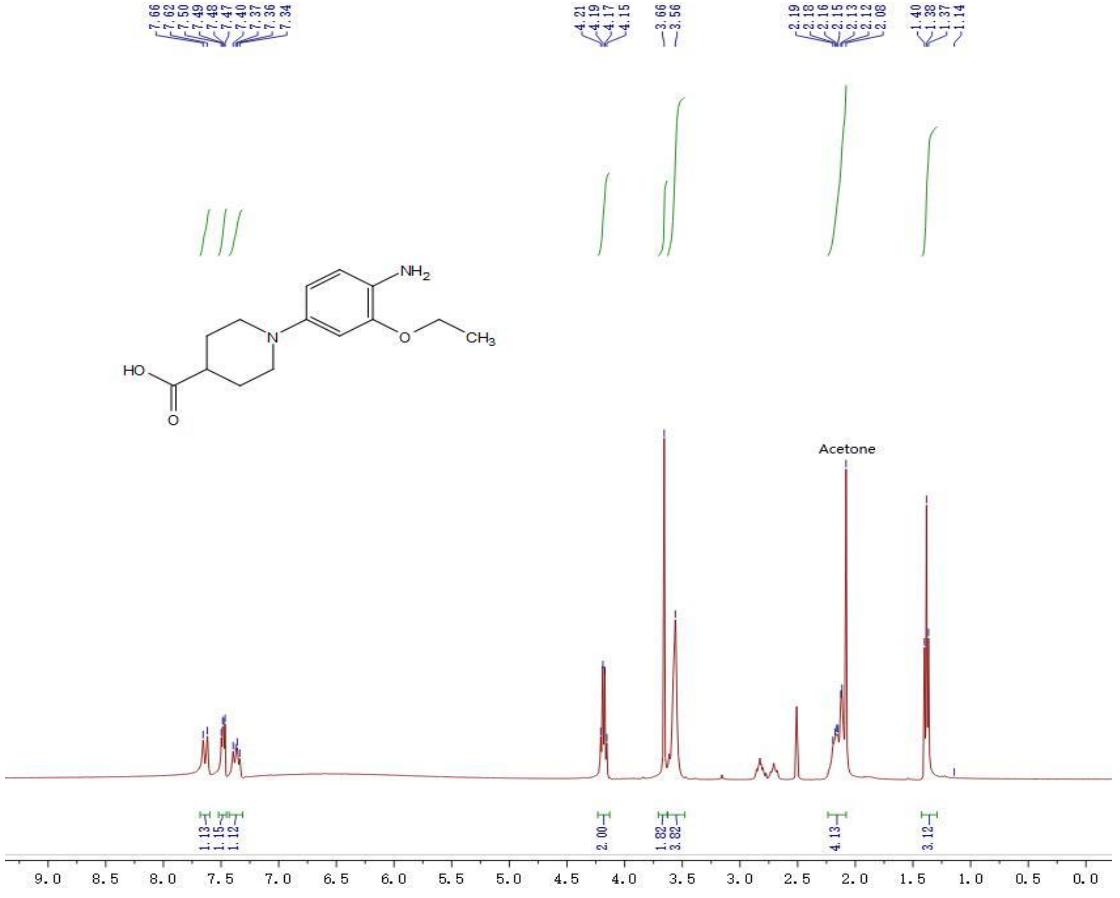

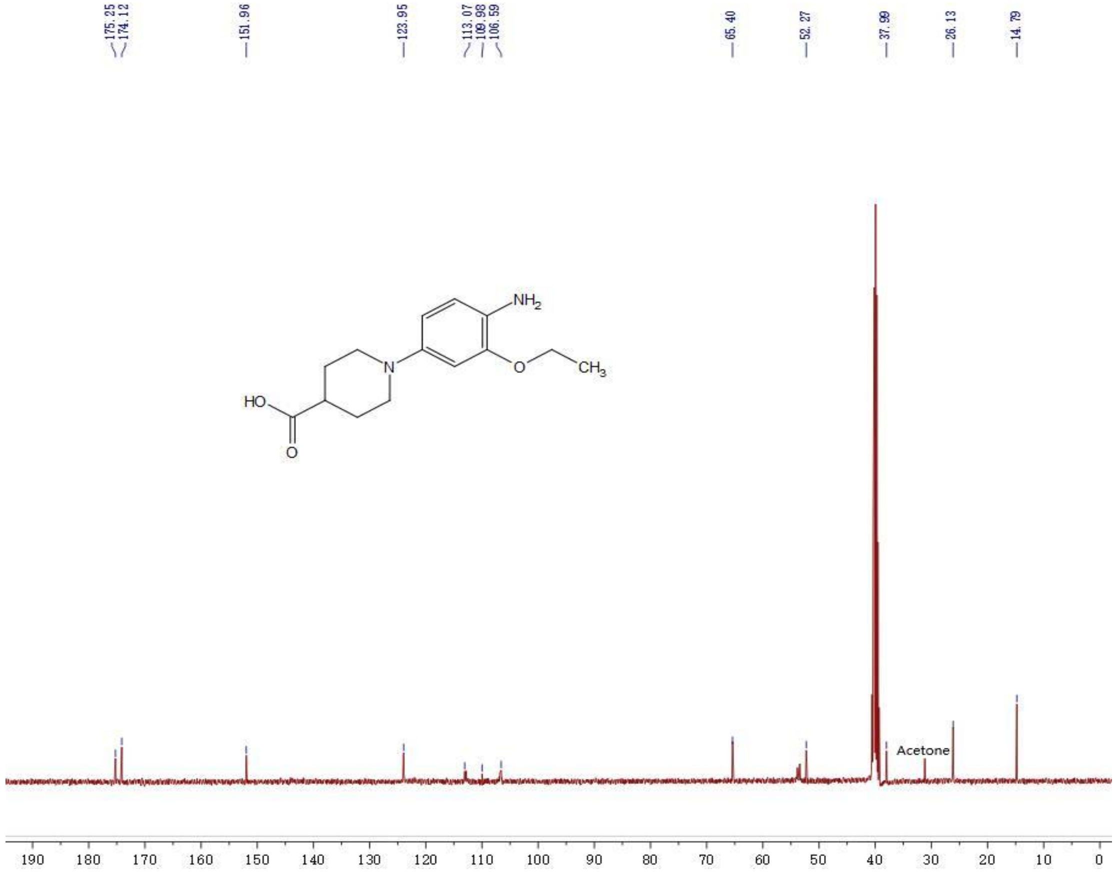

The $^{1}H/^{13}C$ NMR spectrum of Compound **D3**

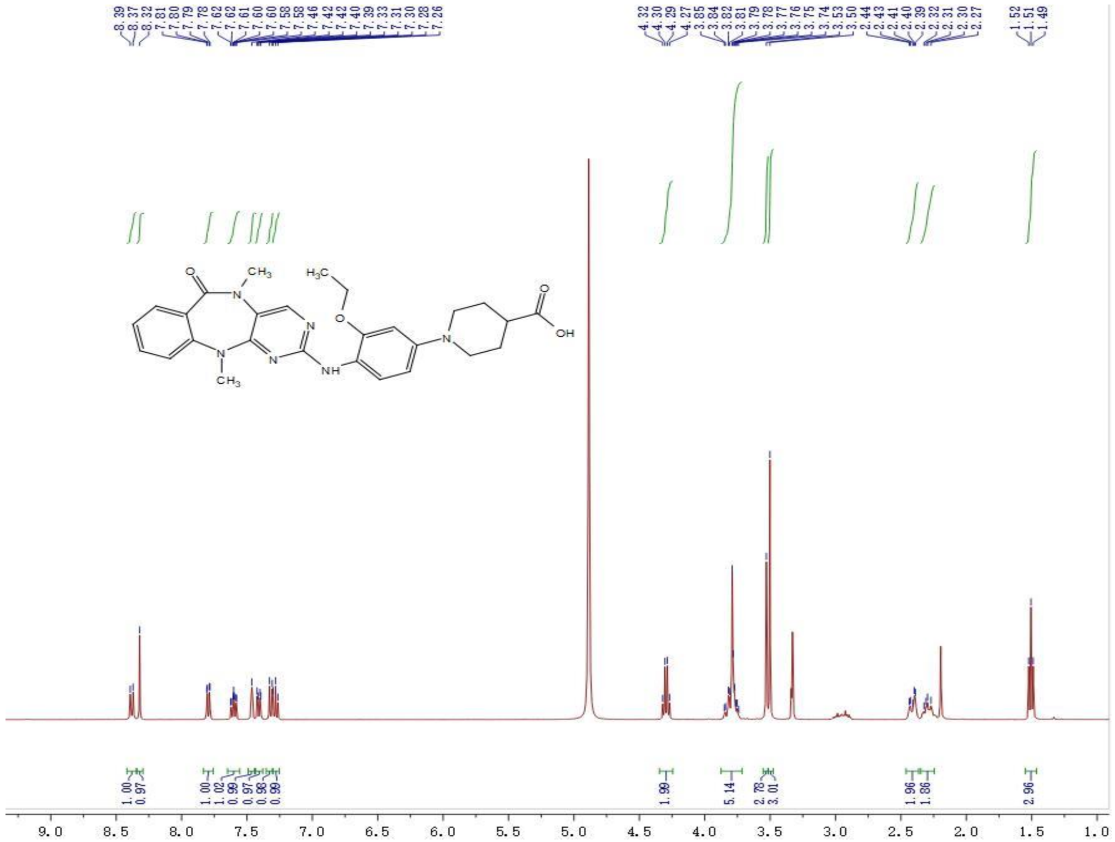

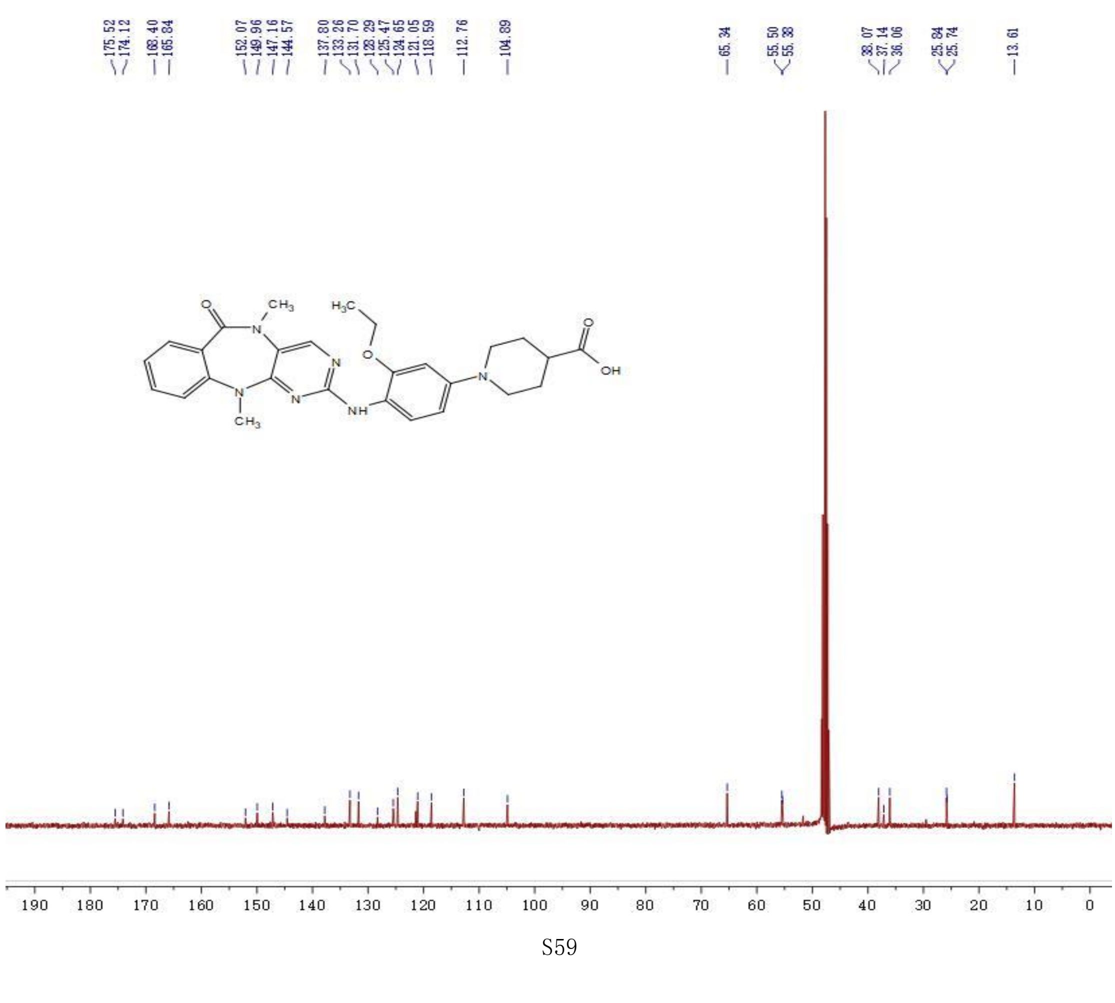

The $^1H/^{13}C$ NMR spectrum of Compound **D4**

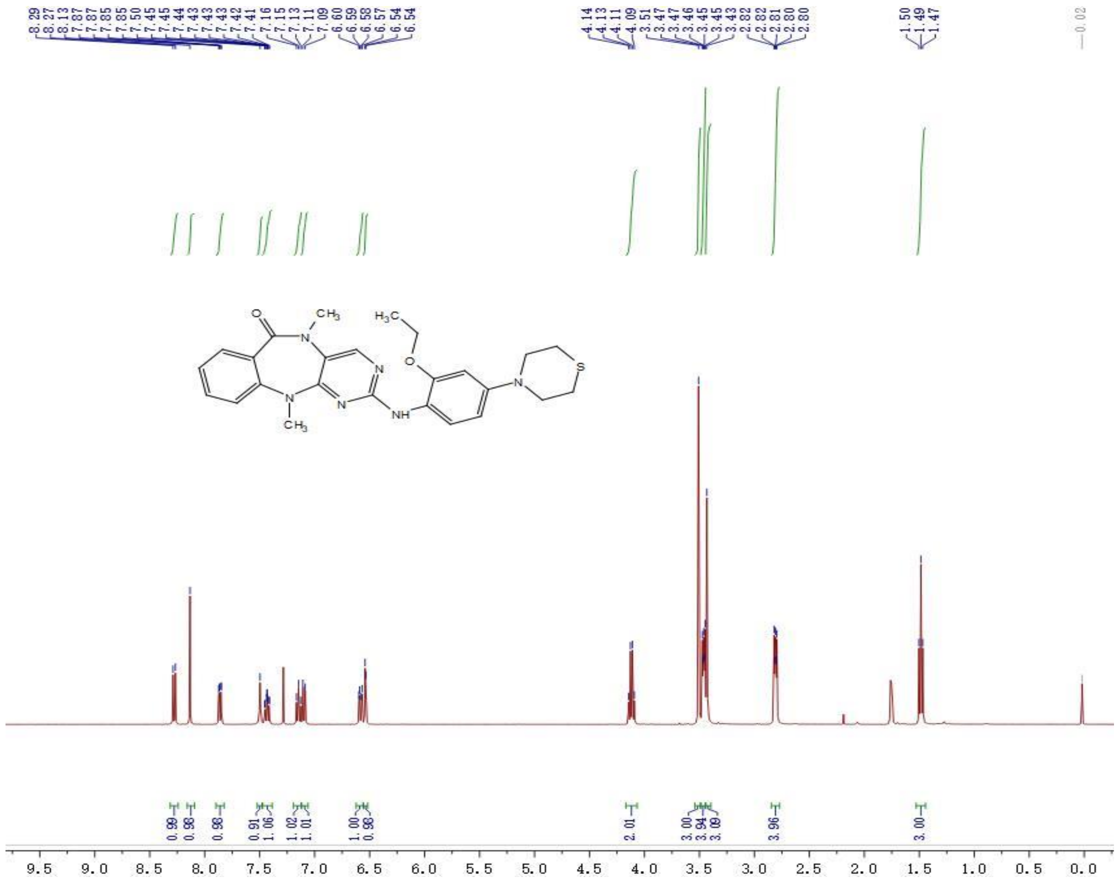

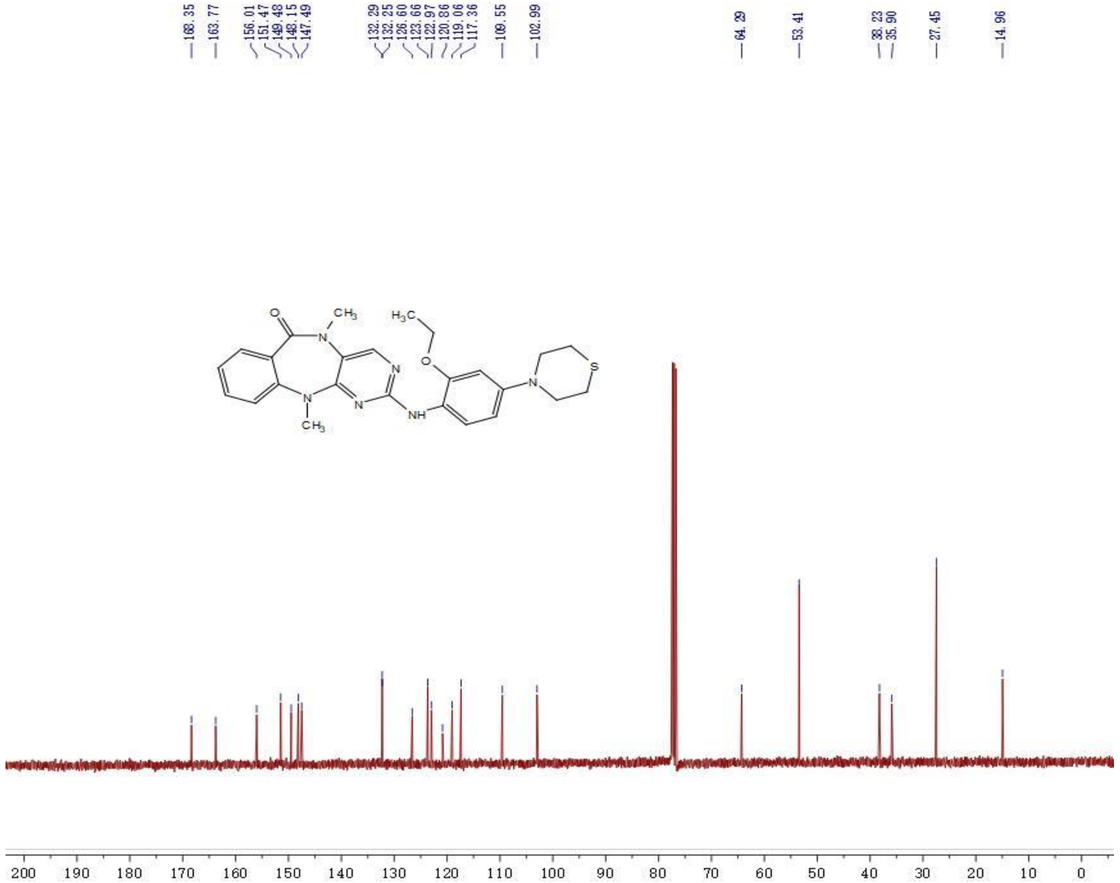

The $^{1}H/^{13}C$ NMR spectrum of Compound **D6**

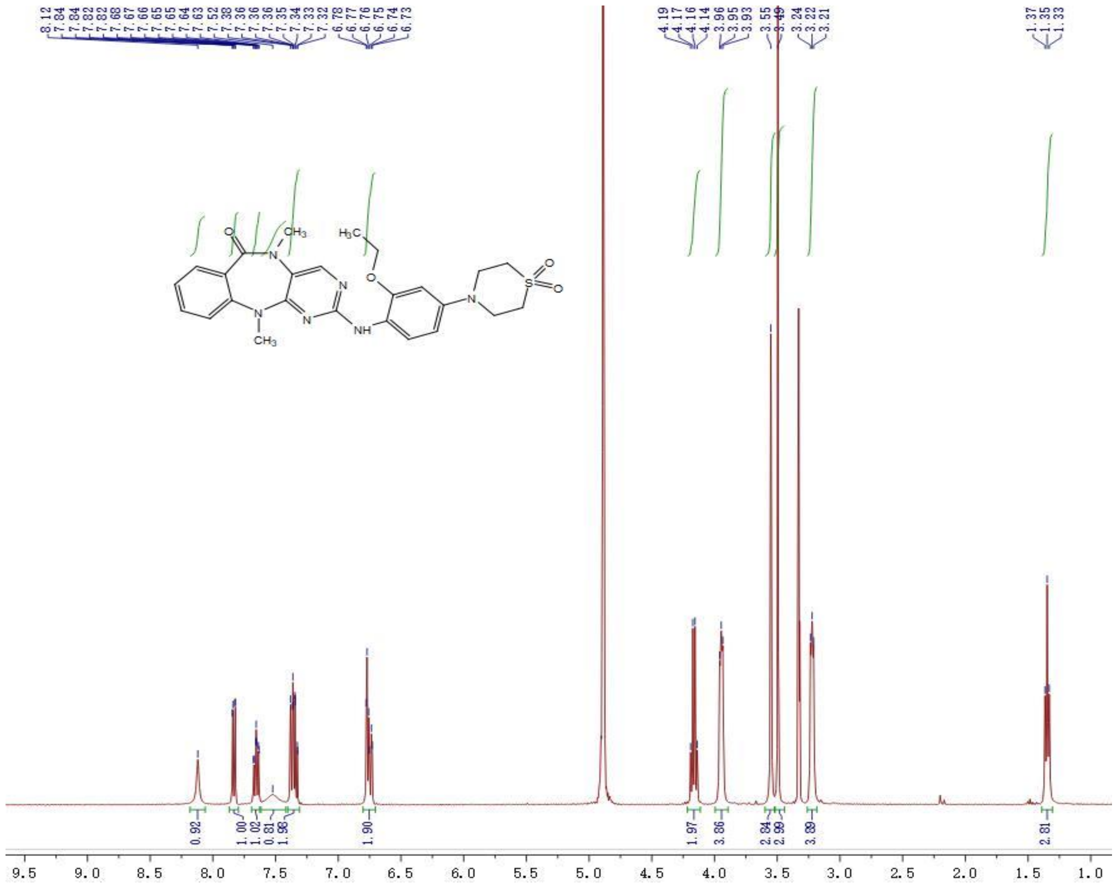

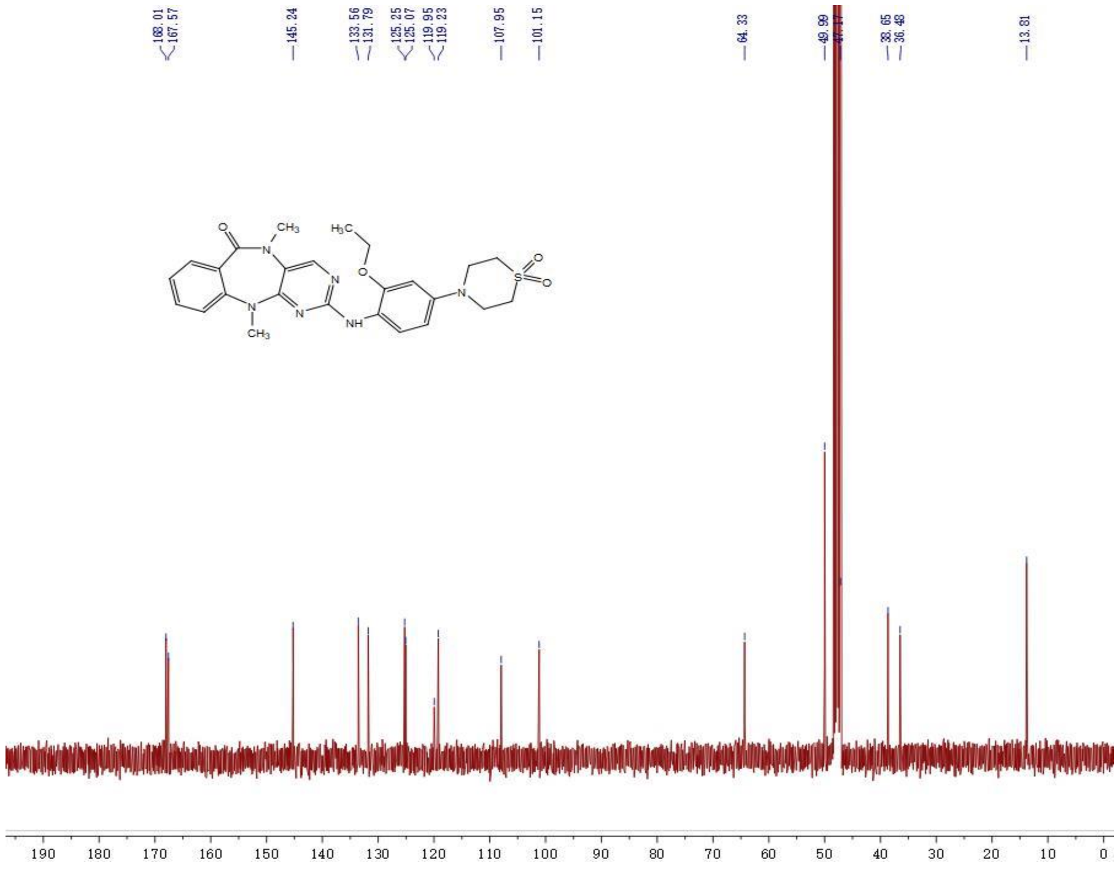

The $^1H/^{13}C$ NMR spectrum of Compound **D9**

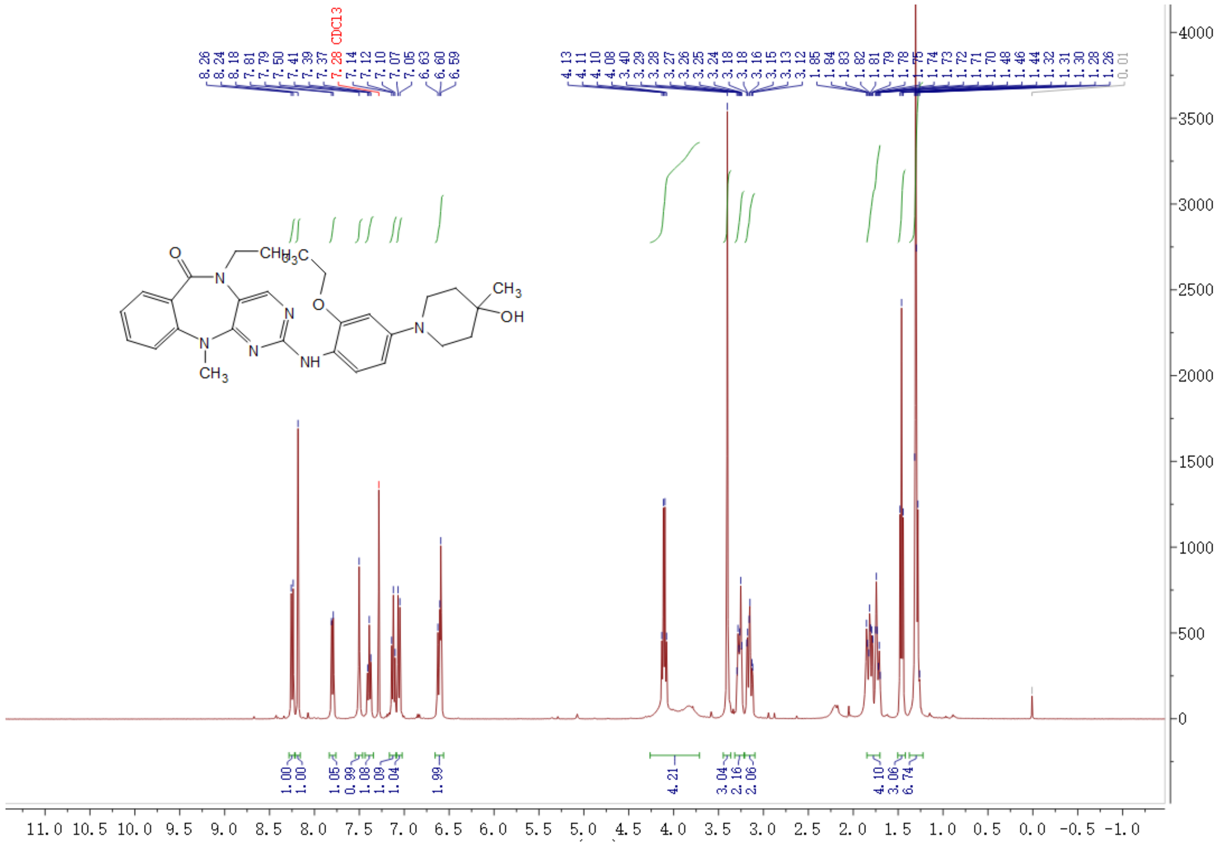

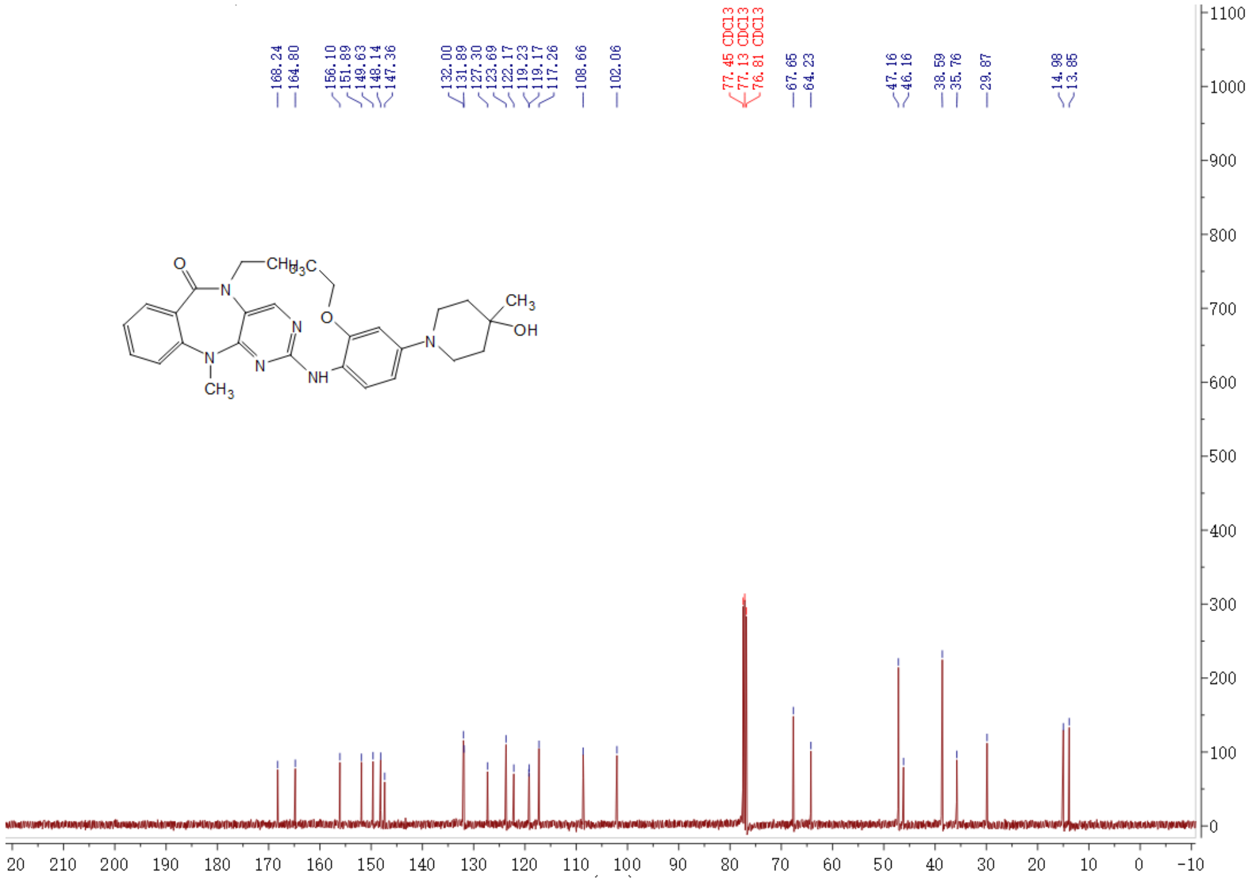

The ^{1}H/^{13}C NMR spectrum of Compound **D10**

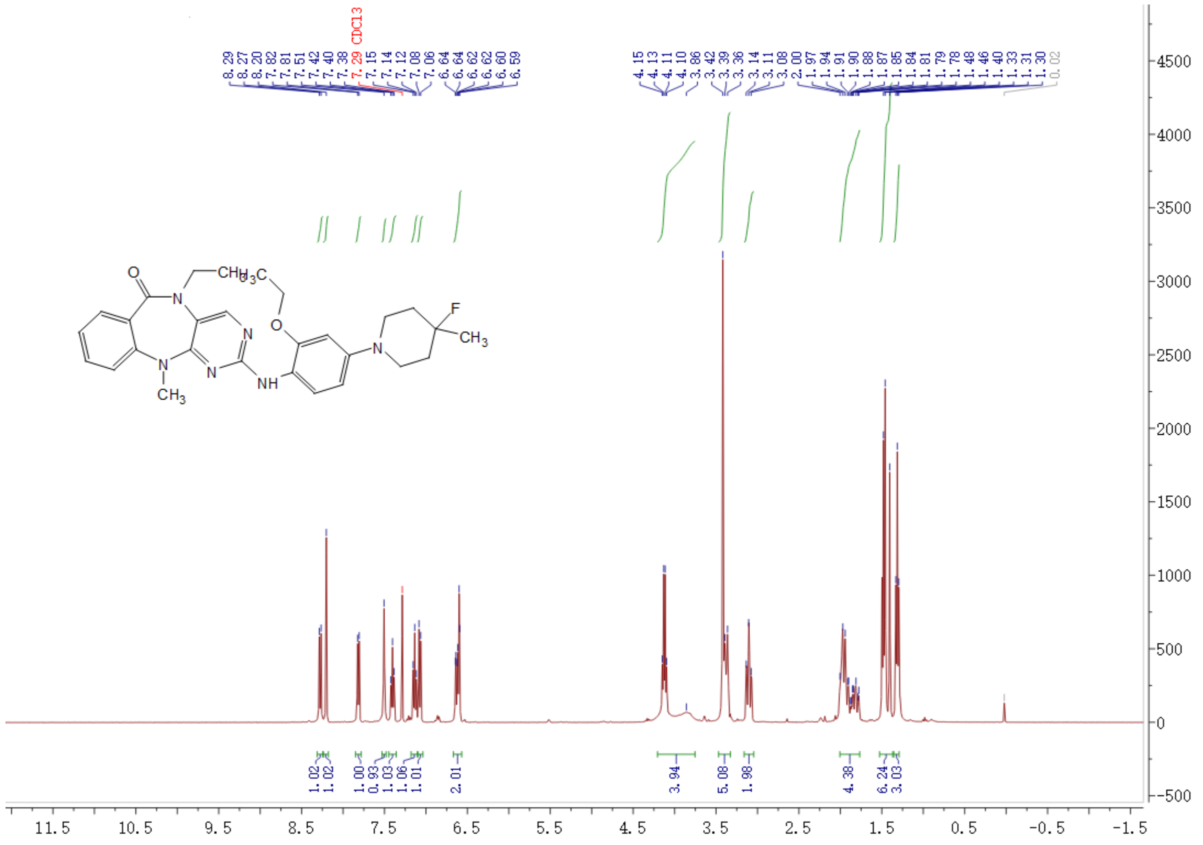

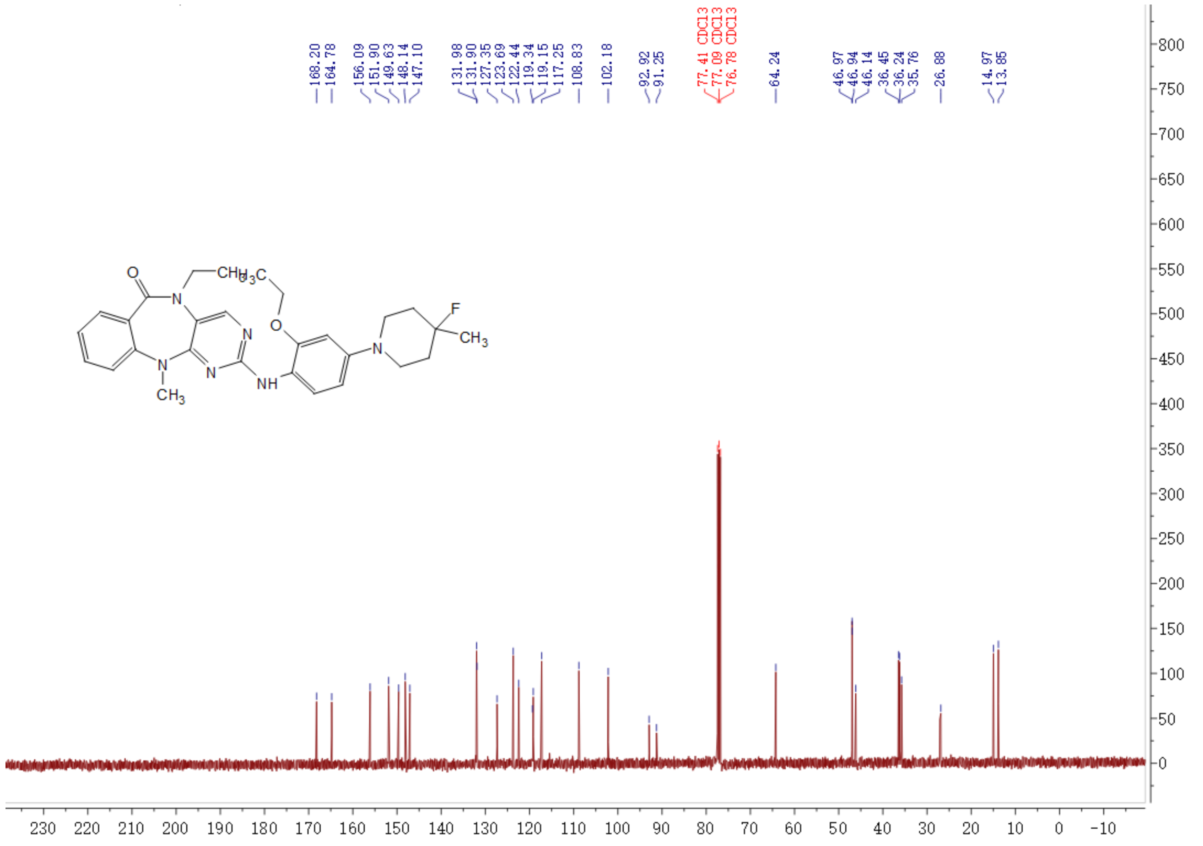

The $^{1}H/^{13}C$ NMR spectrum of Compound **D11**

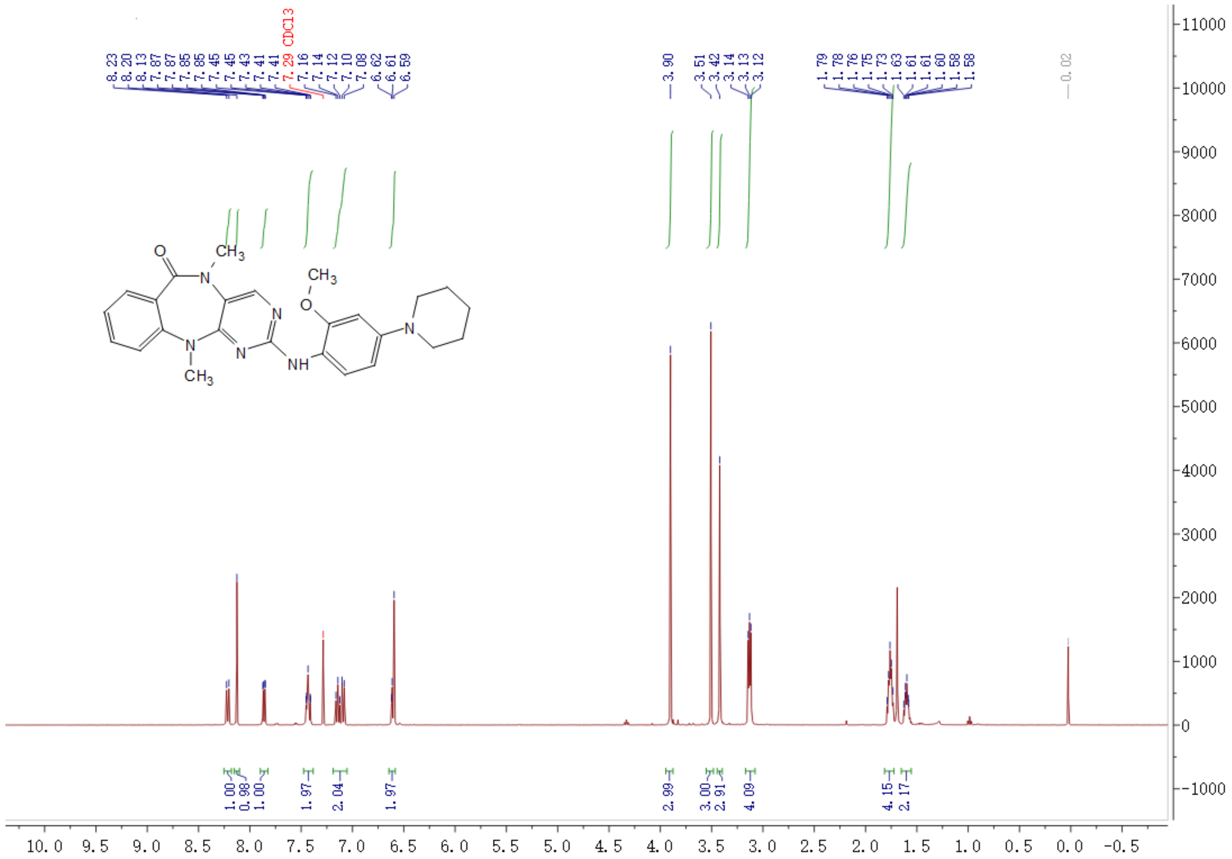

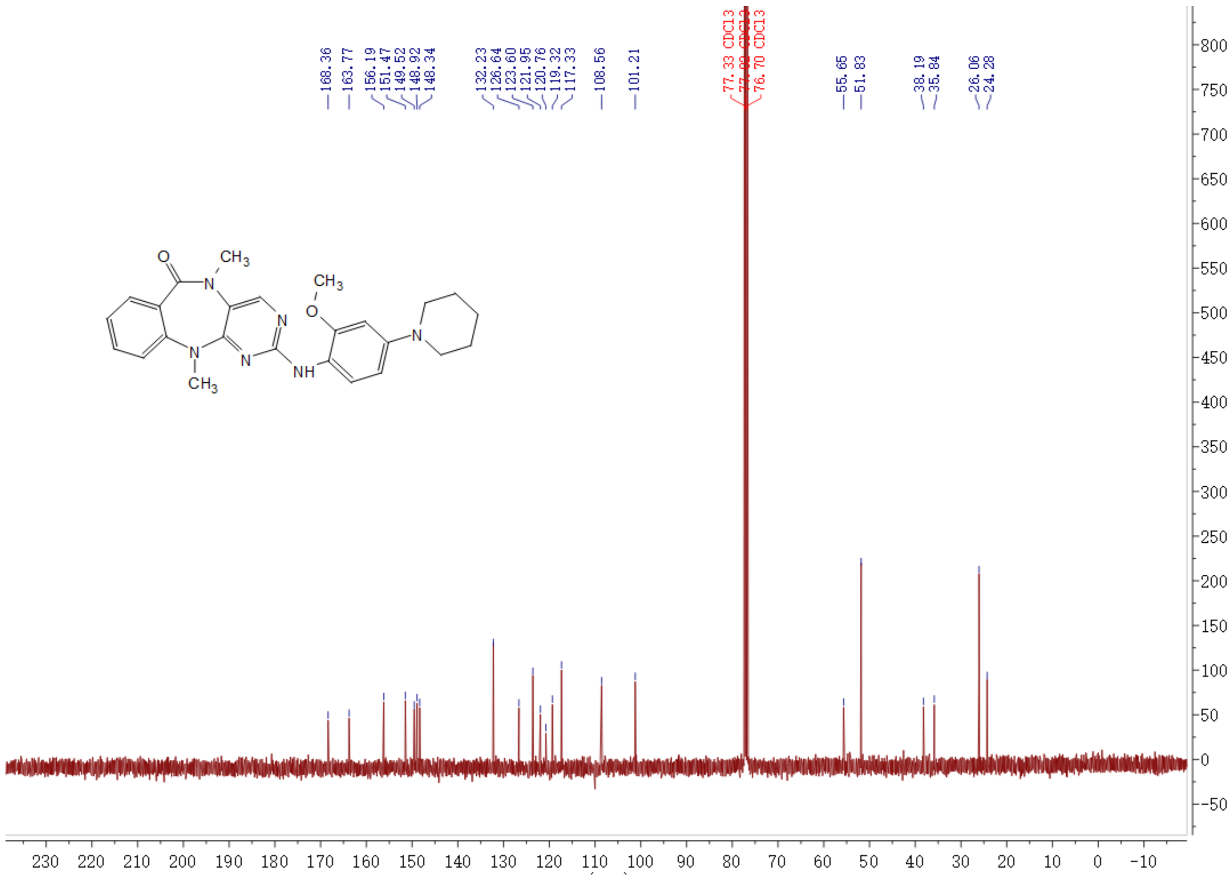

The $^{1}H/^{13}C$ NMR spectrum of Compound **D12**

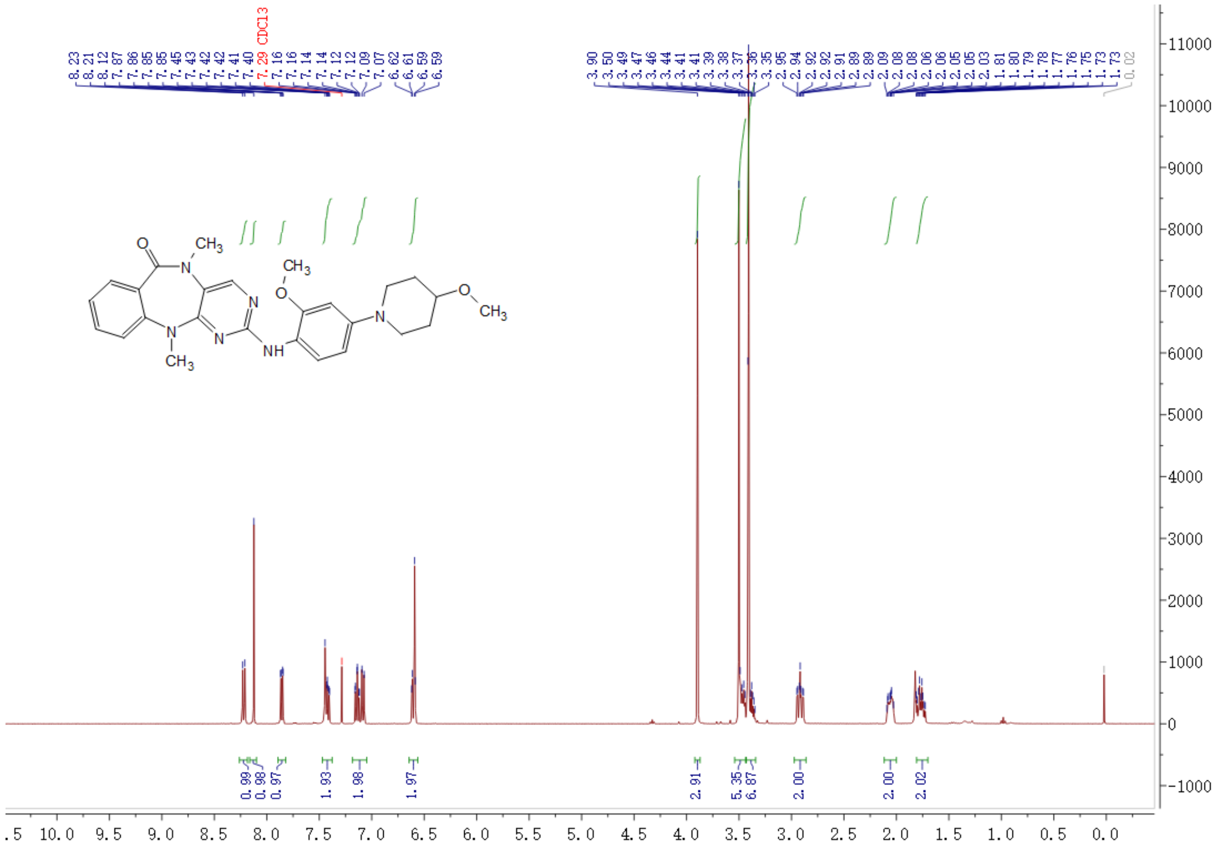

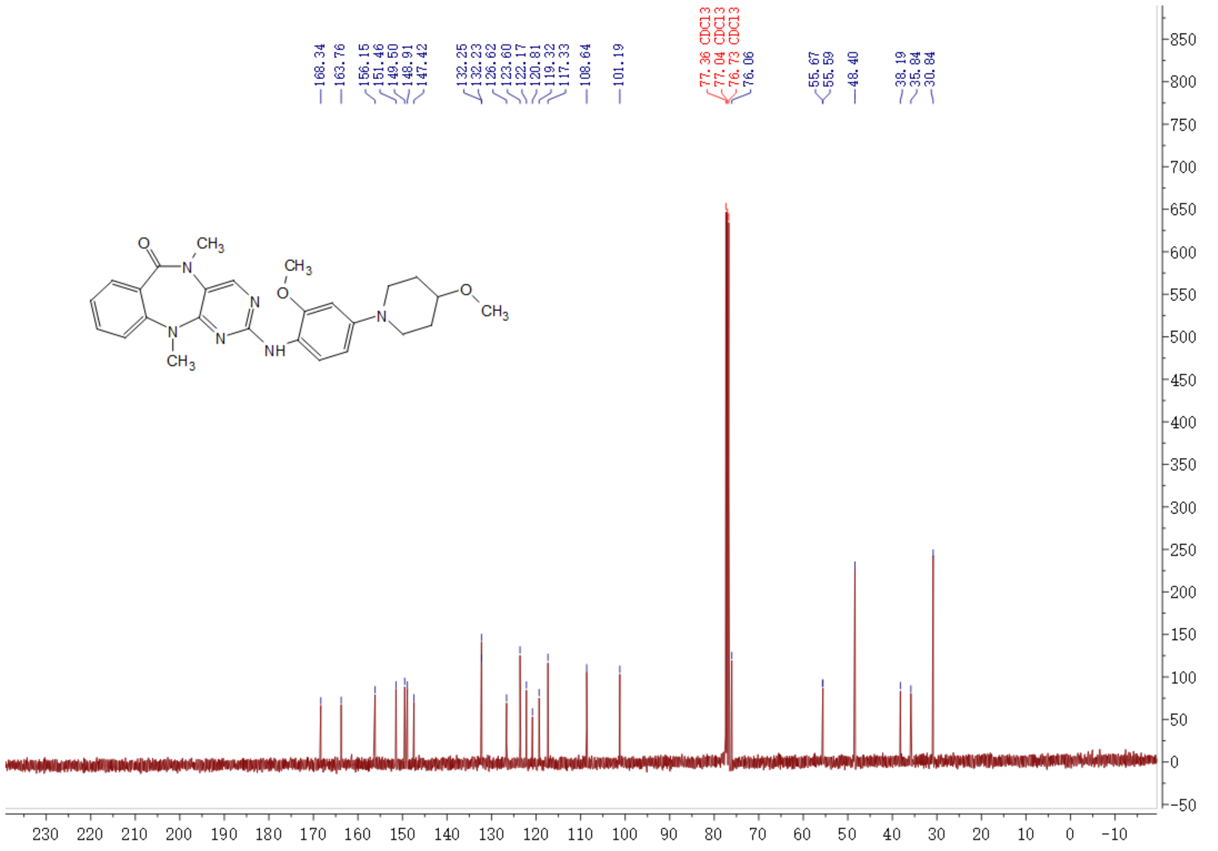

The $^1H/^{13}C$ NMR spectrum of Compound **D13**

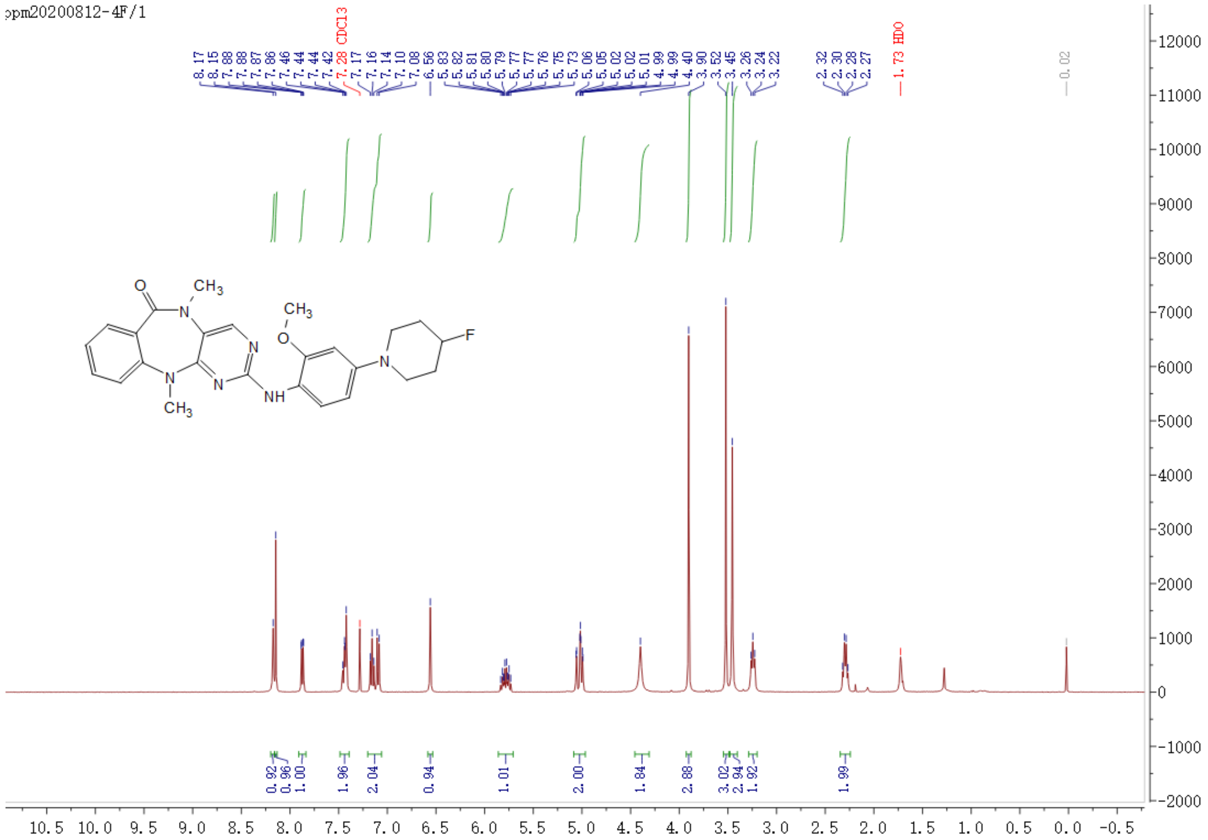

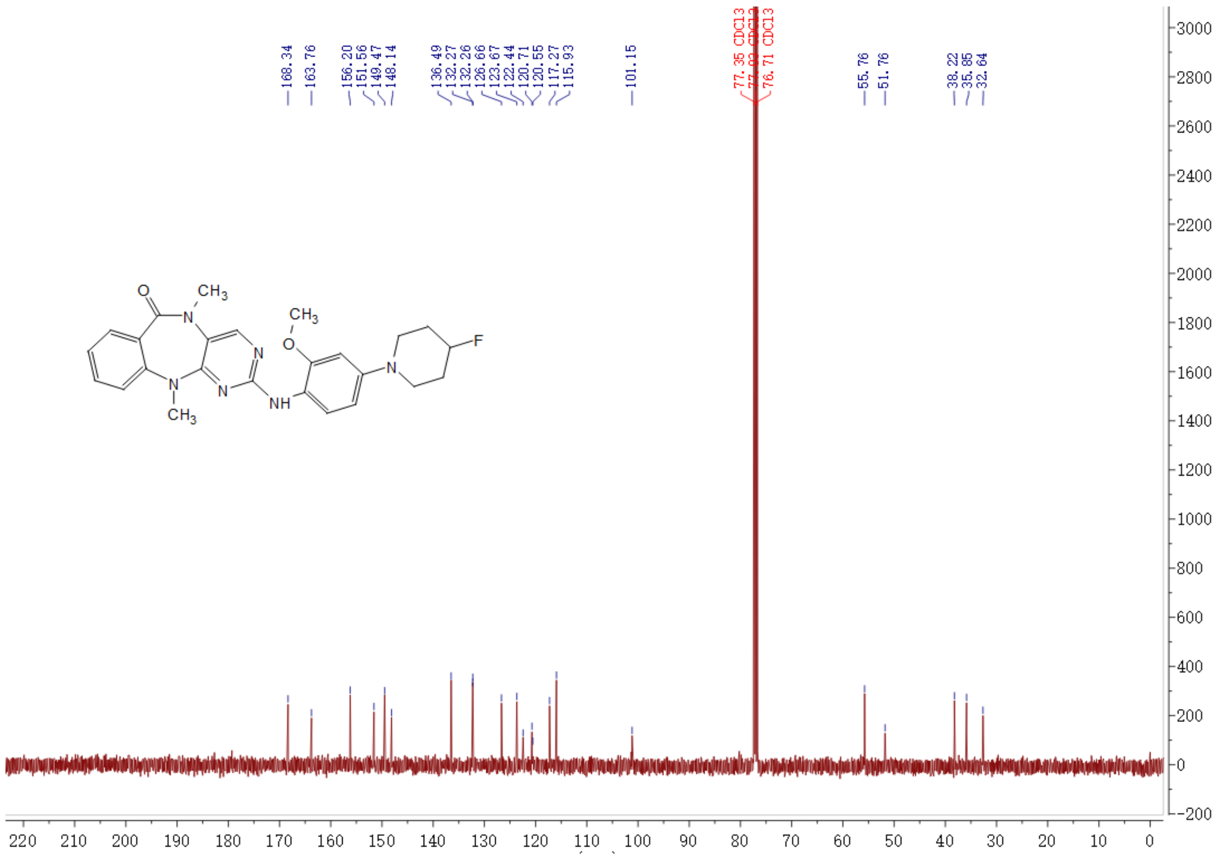

The $^{1}H/^{13}C$ NMR spectrum of Compound **D14**

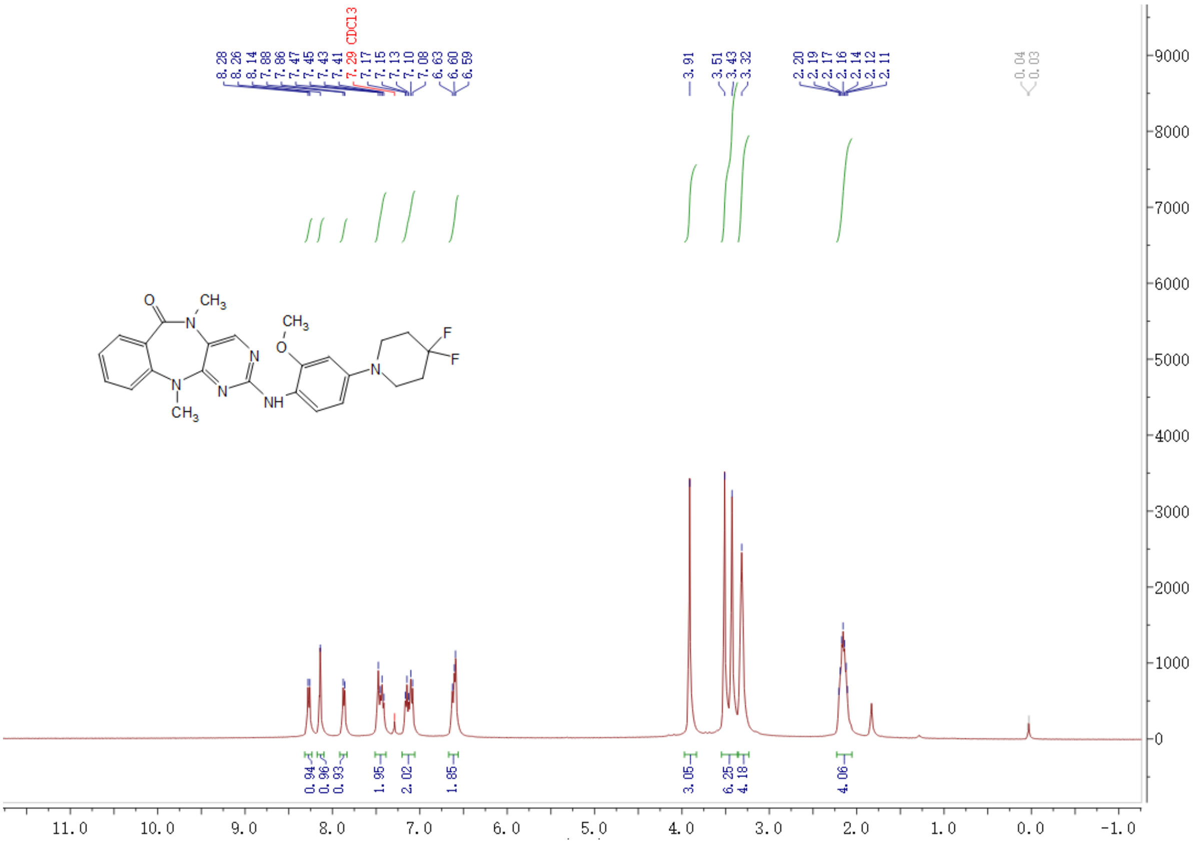

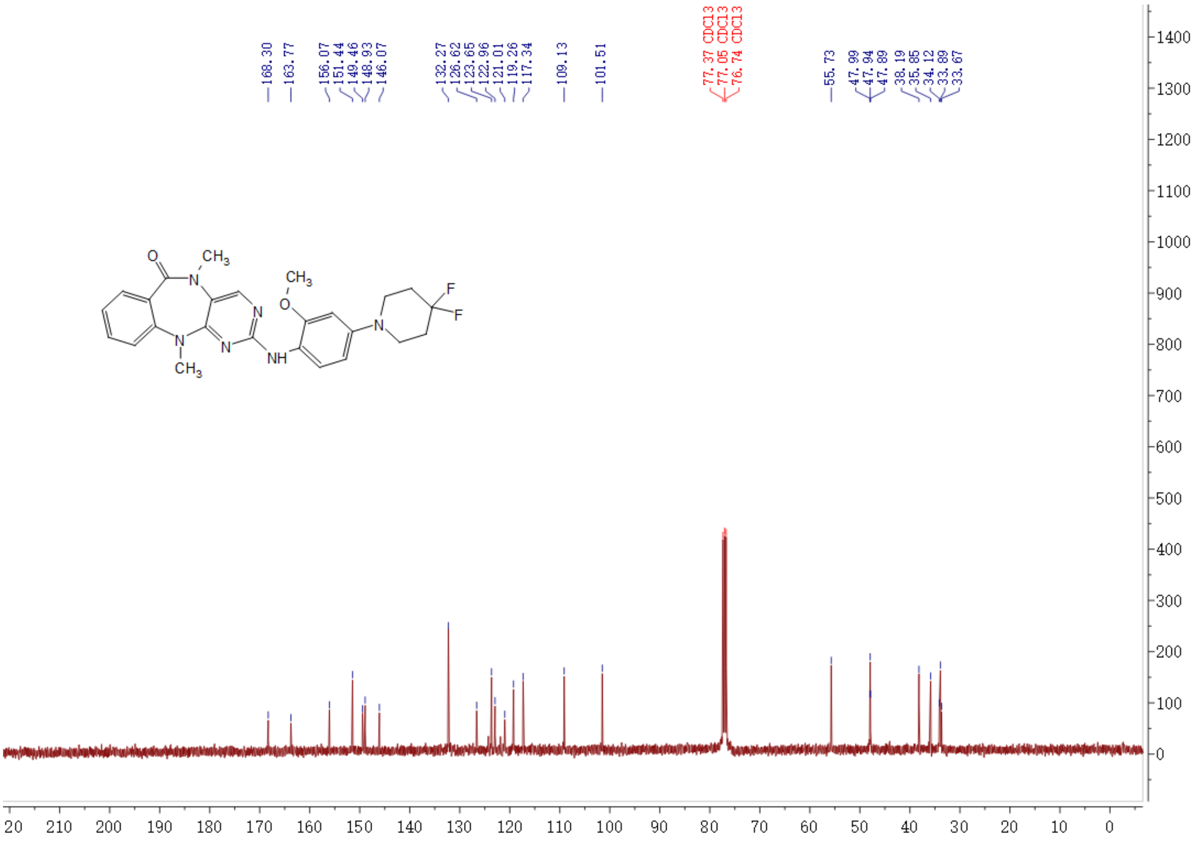

The ^{1}H/^{13}C NMR spectrum of Compound **D15**

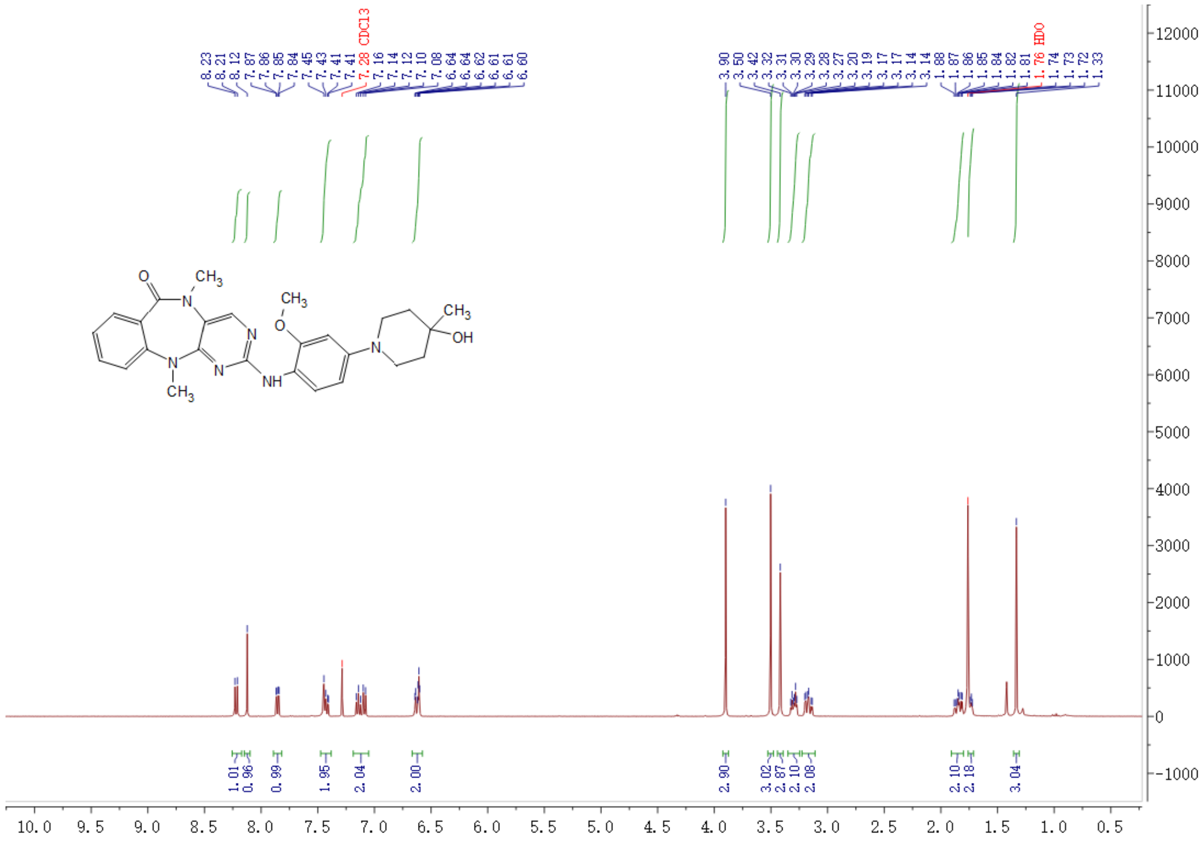

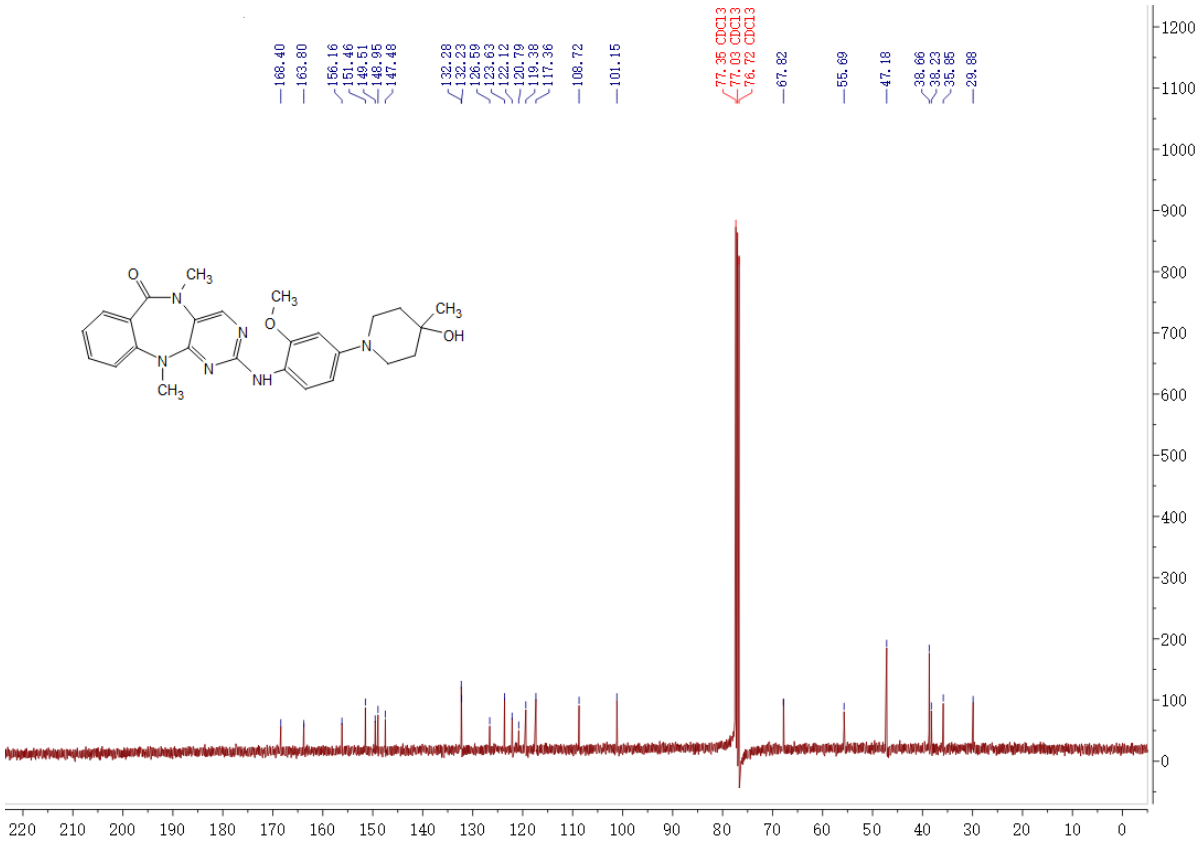

The $^{1}H/^{13}C$ NMR spectrum of Compound **D16**

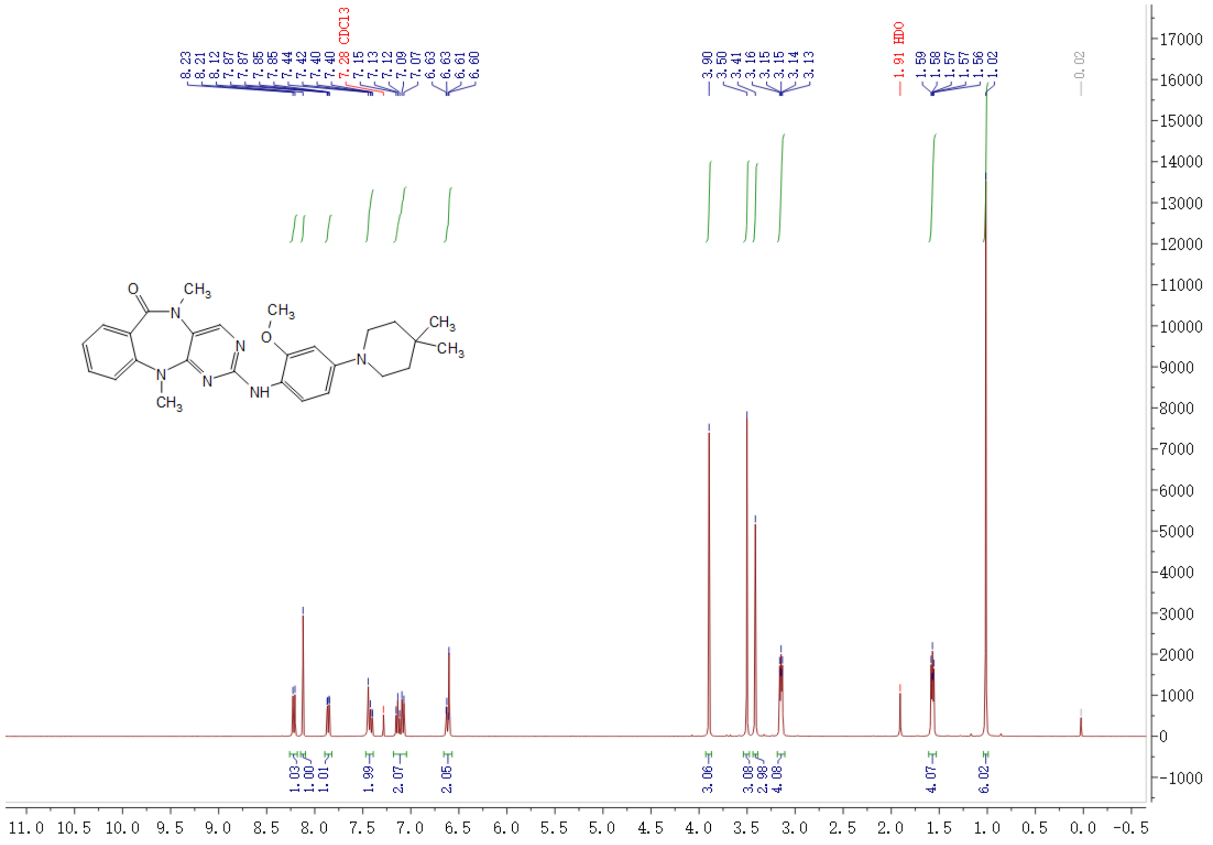

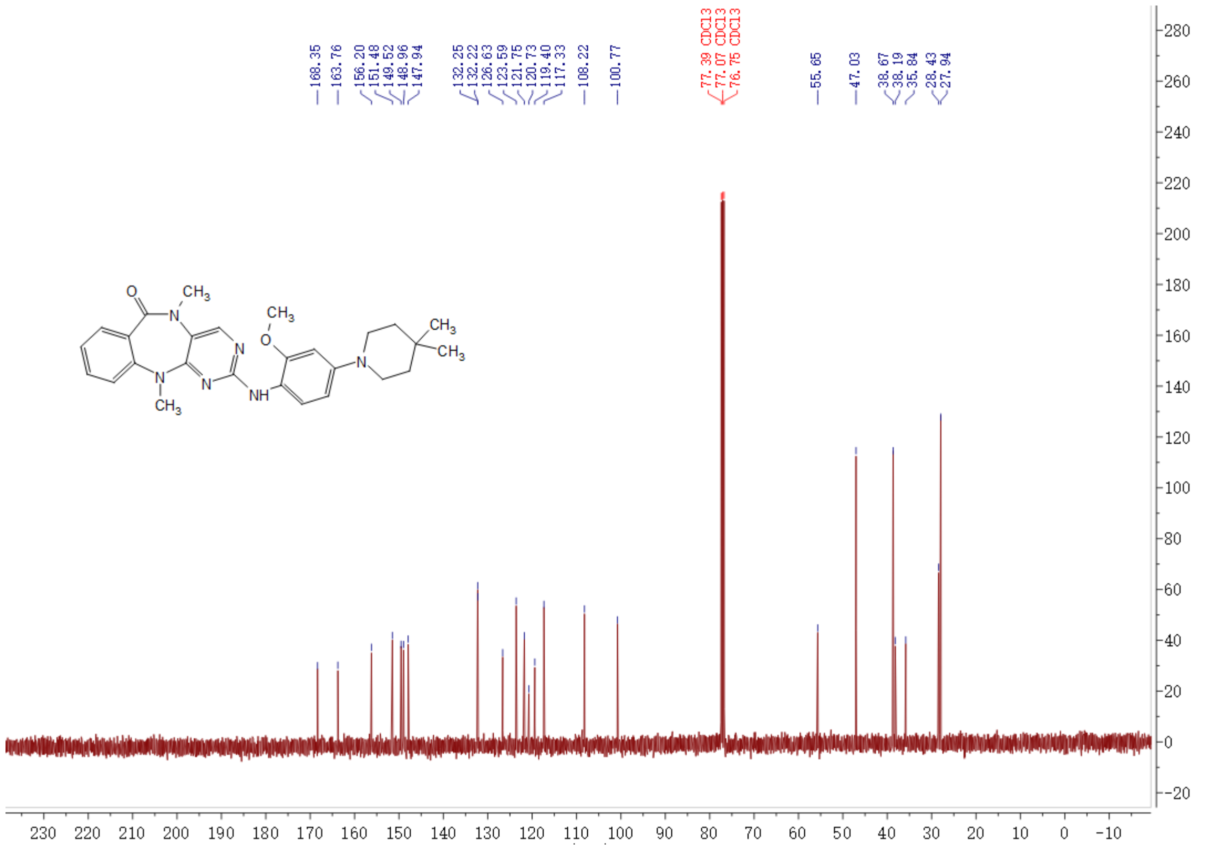

The $^{1}H/^{13}C$ NMR spectrum of Compound **D5**

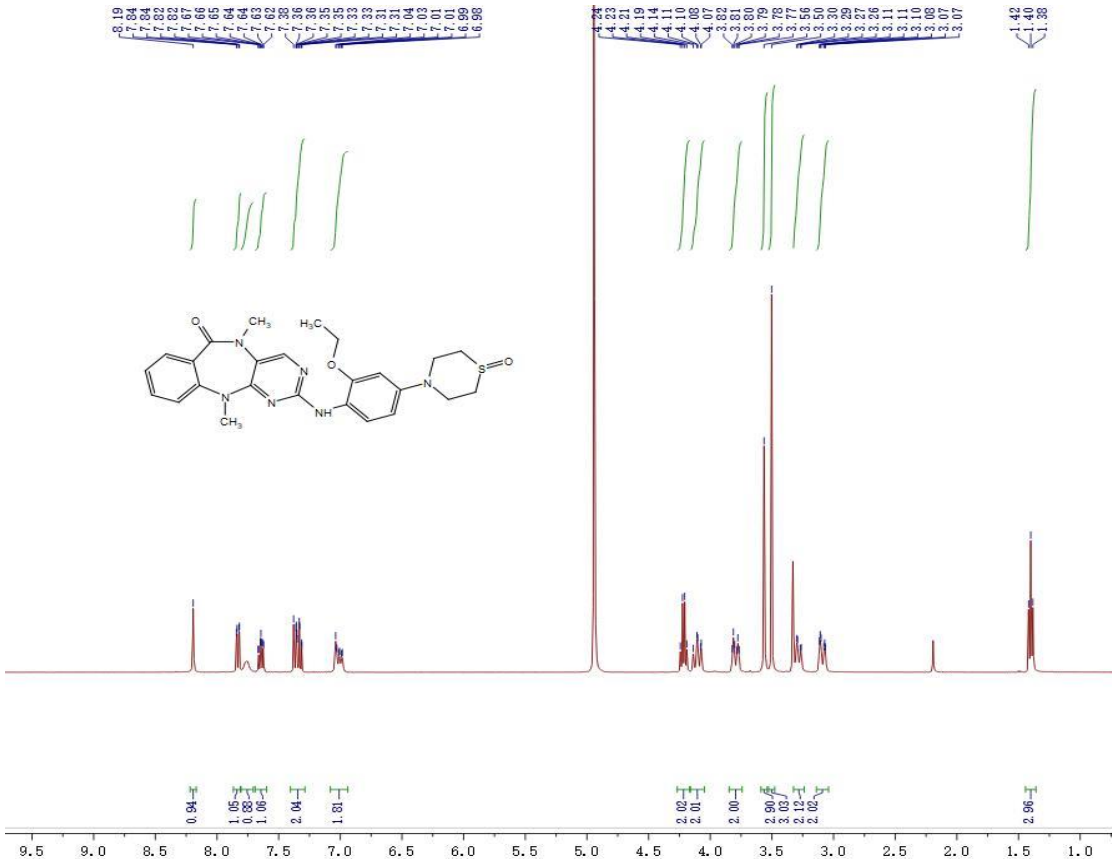

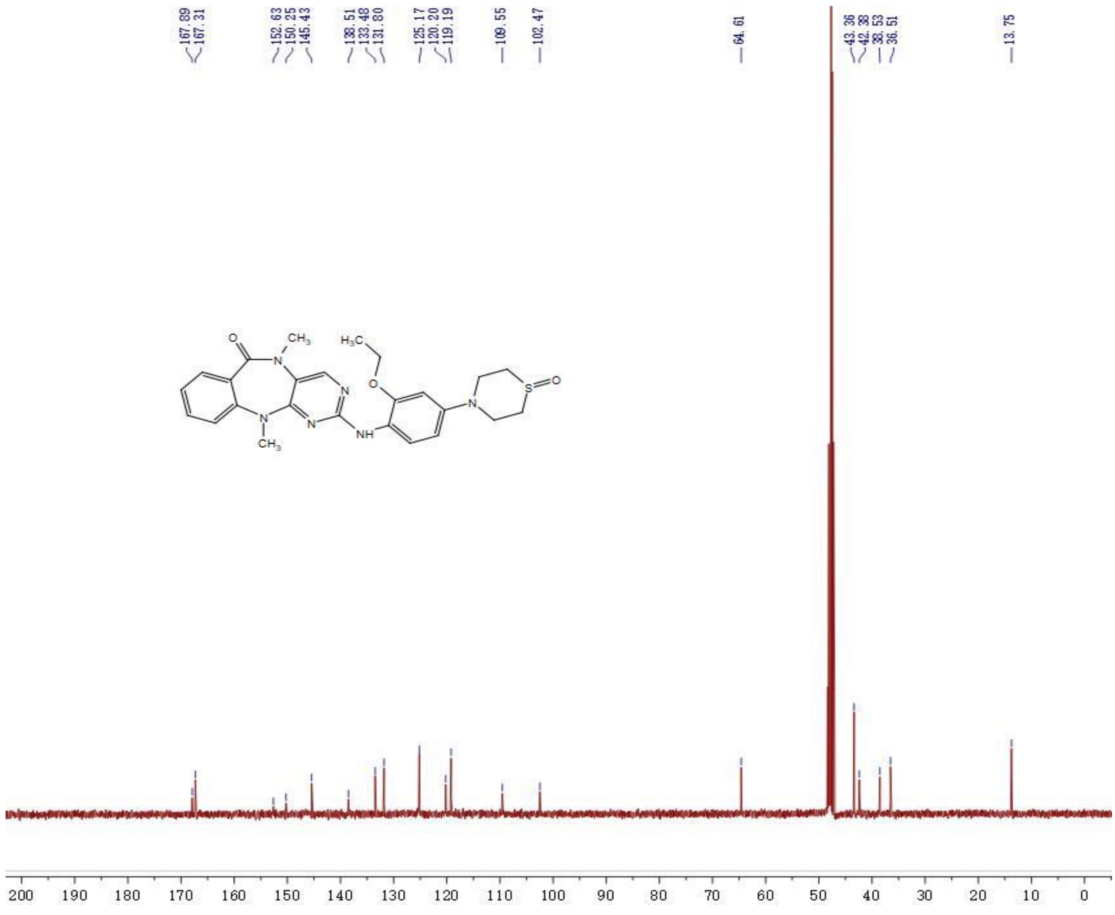

The $^{1}H/^{13}C$ NMR spectrum of intermediate **20**

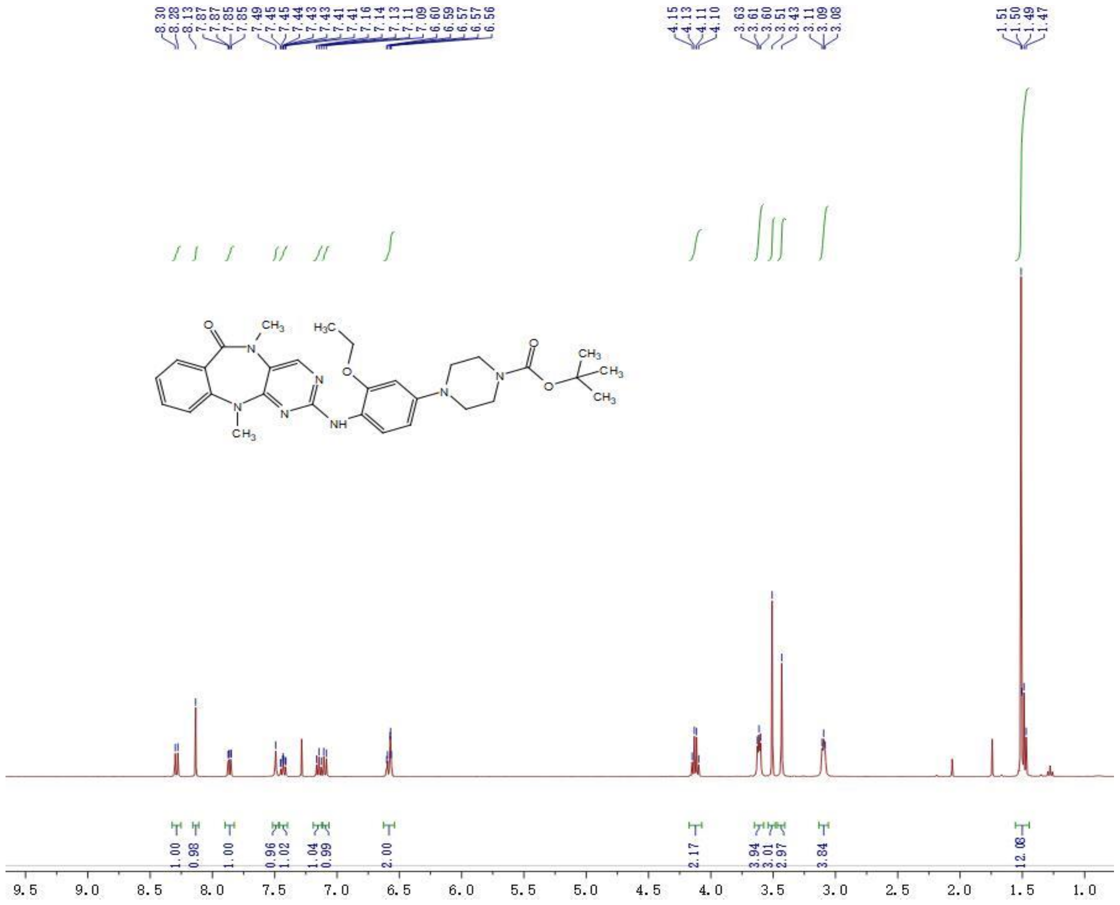

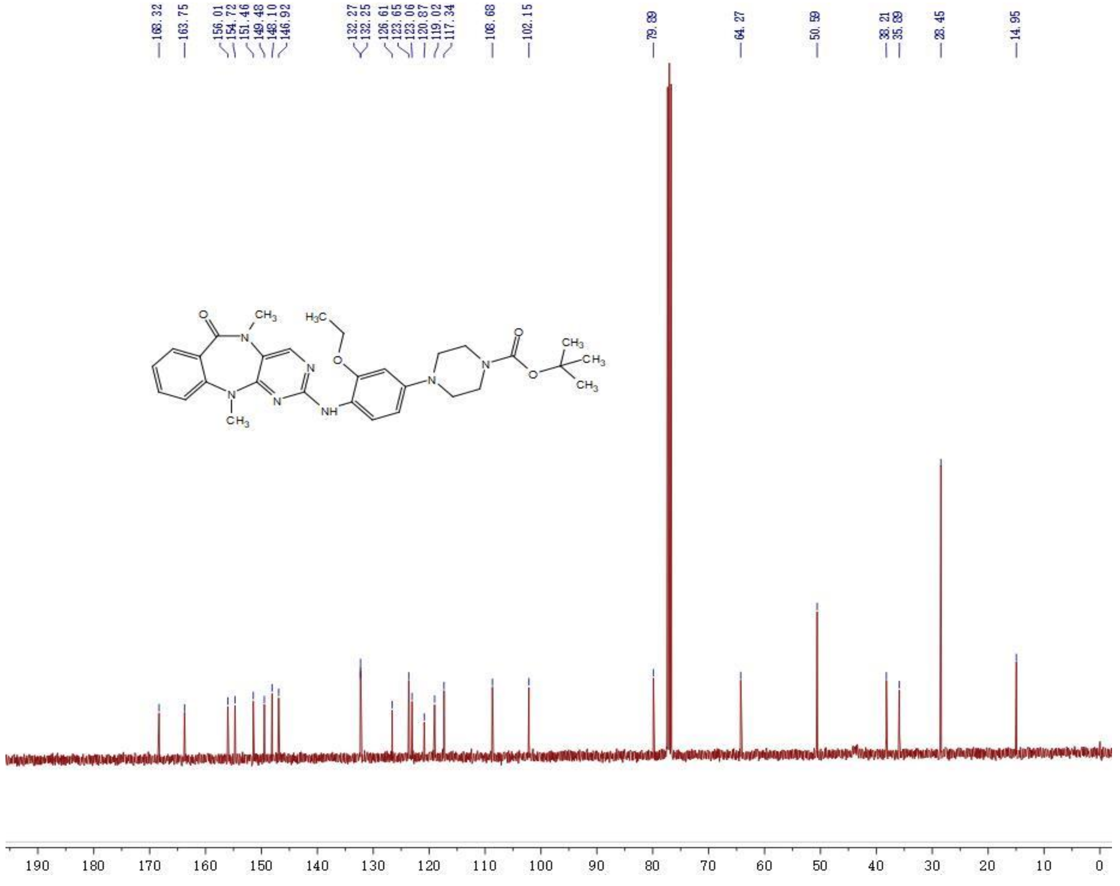

The $^{1}H/^{13}C$ NMR spectrum of intermediate **21**

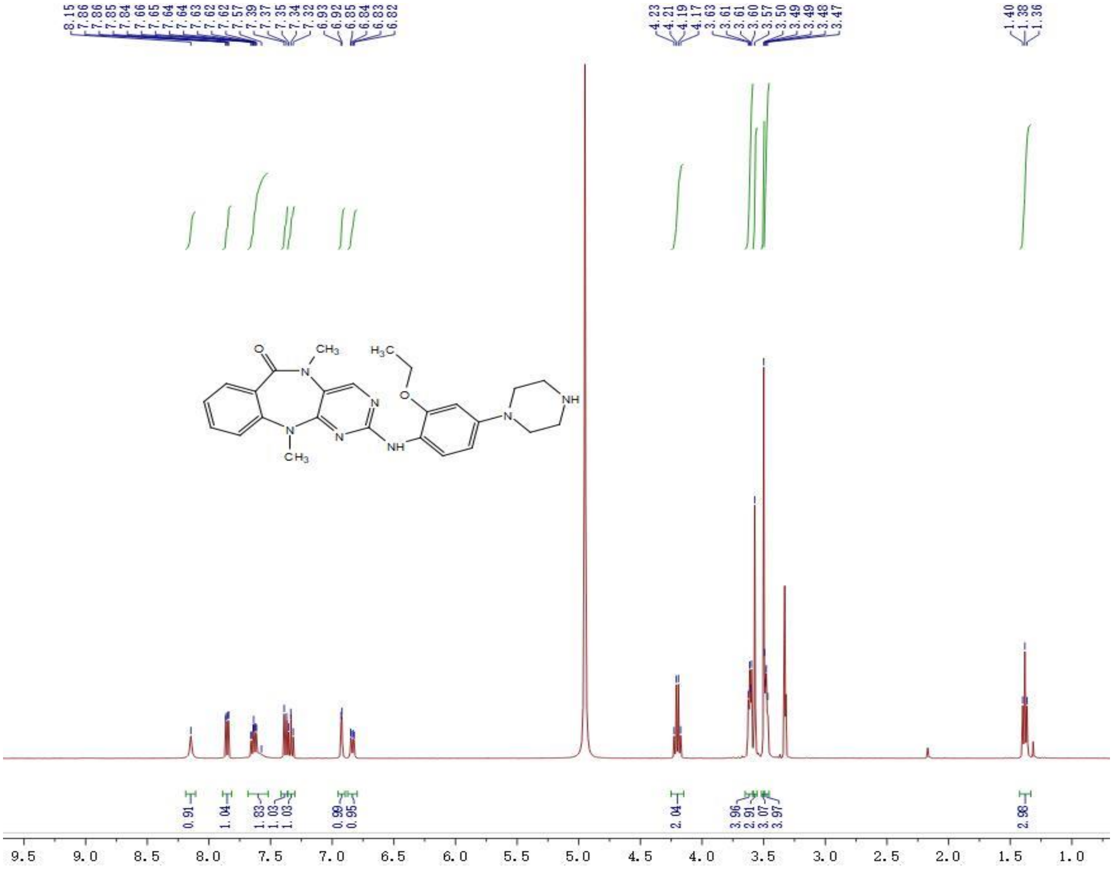

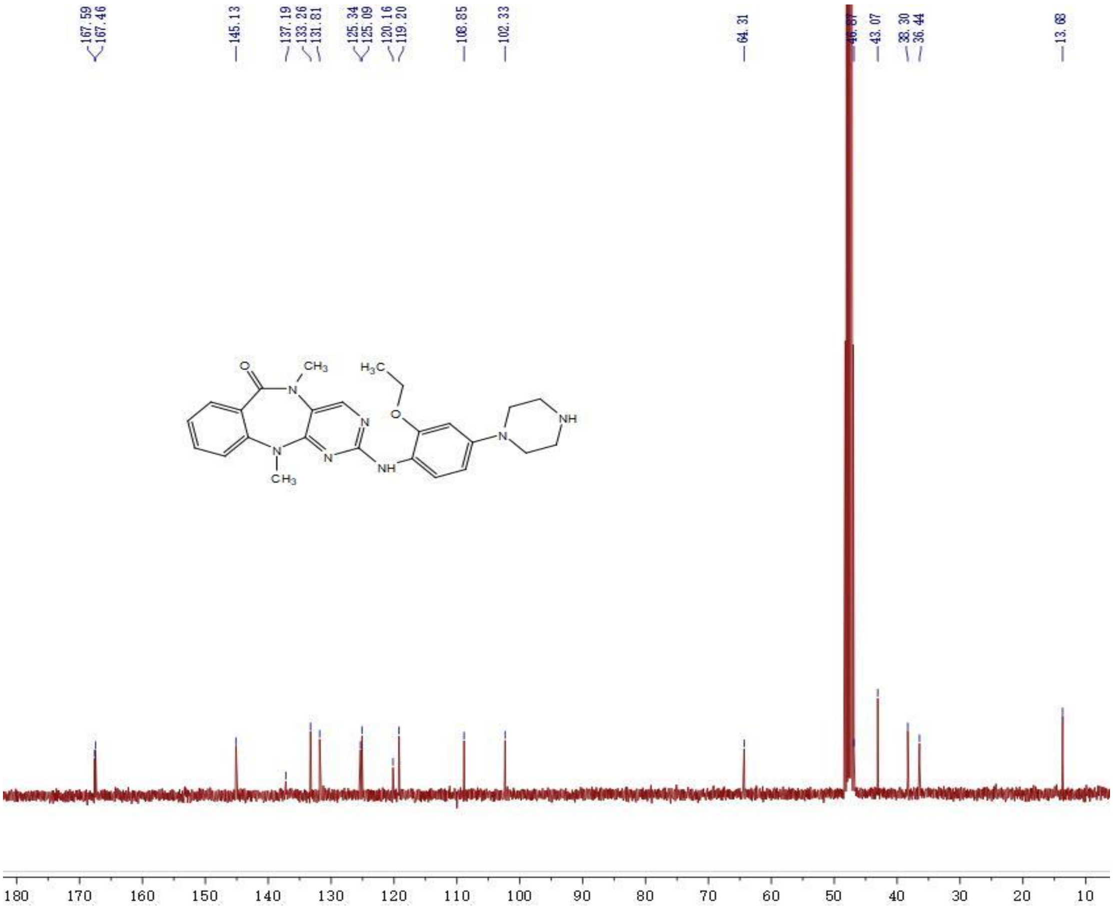

The $^{1}H/^{13}C$ NMR spectrum of Compound **D7**

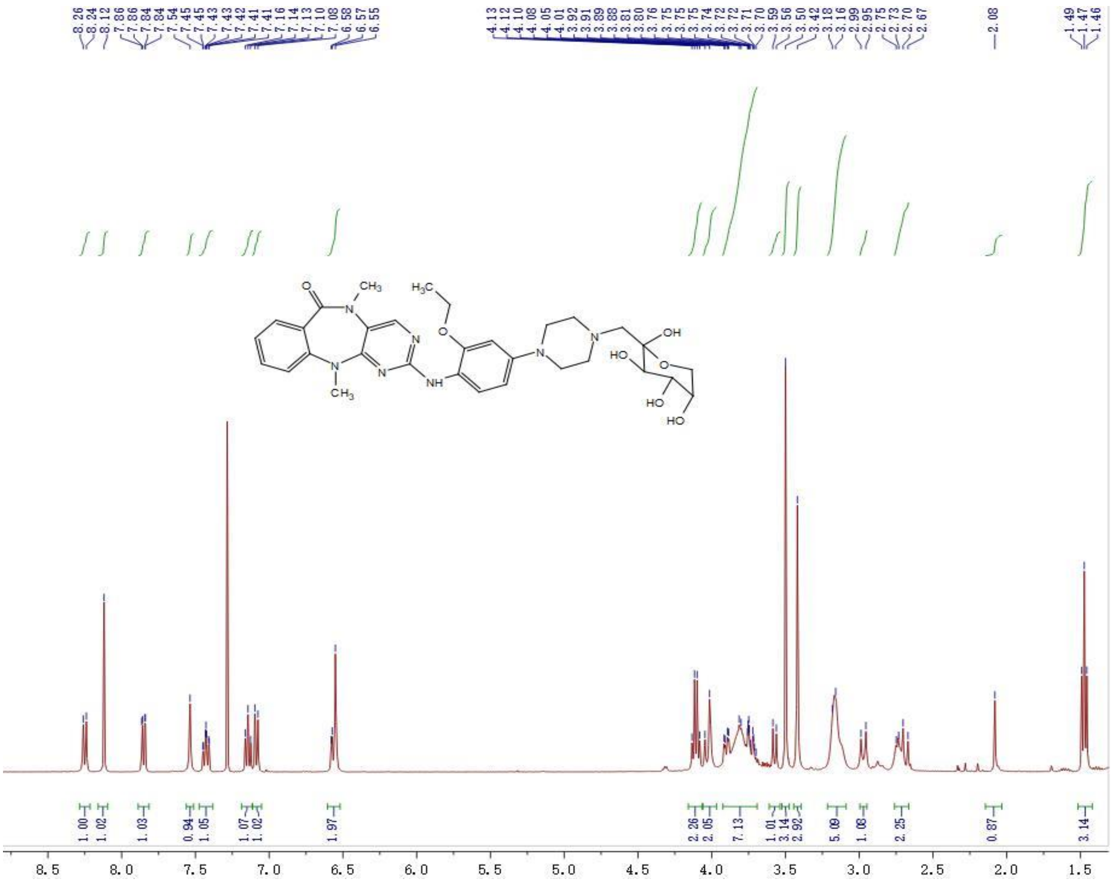

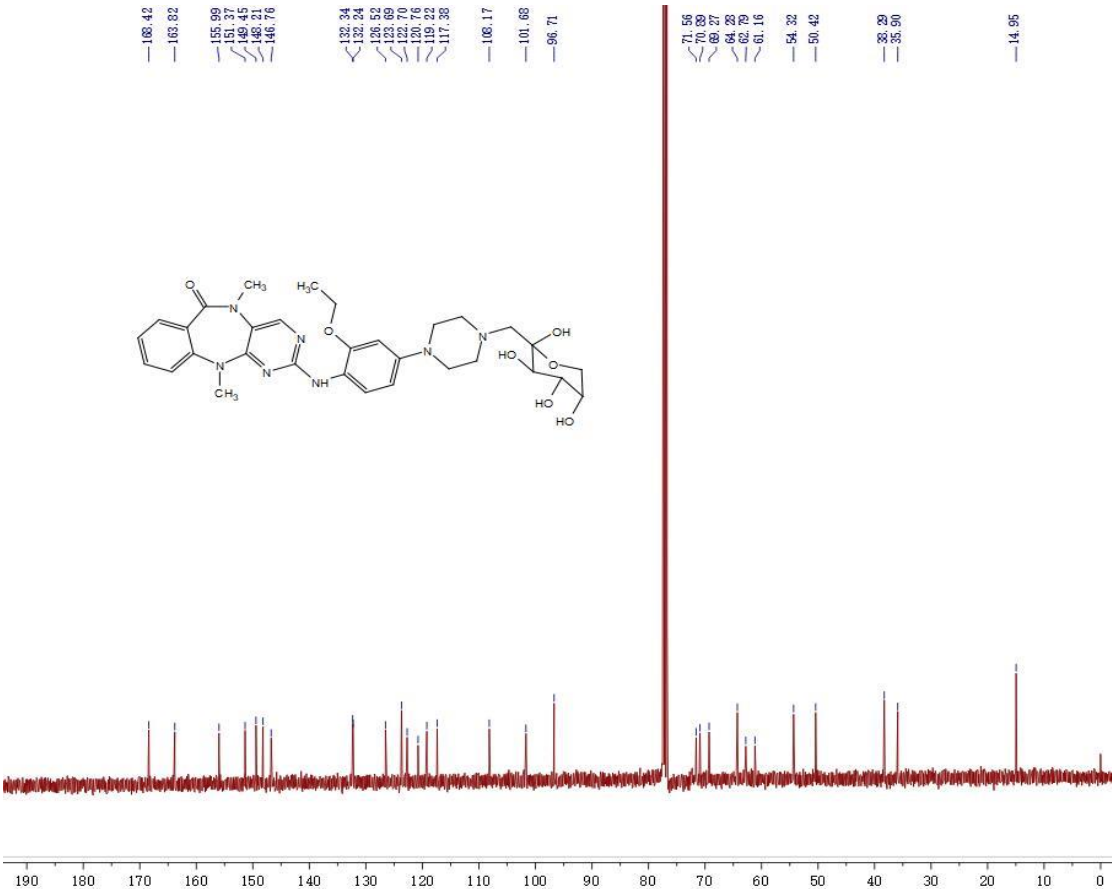

Supplement: Supplemental Material [file IENZ_A_2287990_SM0486.pdf]
